# Asymmetric higher-order [10 + *n*] cycloadditions of palladium-containing 10π-cycloaddends†

**DOI:** 10.1039/d2sc02985e

**Published:** 2022-07-15

**Authors:** Ao Li, Yang Gao, Jian-Bin Lu, Zhi-Chao Chen, Wei Du, Ying-Chun Chen

**Affiliations:** Key Laboratory of Drug-Targeting and Drug Delivery System of the Education Ministry and Sichuan Province, Sichuan Research Center for Drug Precision Industrial Technology, West China School of Pharmacy, Sichuan University Chengdu 610041 China duweiyb@scu.edu.cn ycchen@scu.edu.cn +86 28 85502609; College of Pharmacy, Third Military Medical University Shapingba, Chongqing 400038 China

## Abstract

We uncovered an asymmetric higher-order [10 + 2] cycloaddition reaction between diverse activated alkenes and a new type of π-allylpalladium complex-containing dipole-type 10π-cycloaddend, which was generated *in situ* from 2-methylene-1-indanols *via* a dehydrative insertion and deprotonation strategy under double activation of Pd(0) and phosphoric acid. A similar strategy was applied to an asymmetric higher-order [10 + 8] cycloaddition reaction or [10 + 4] cycloaddition reaction by using a heptafulvene derivative or a cyclic enone, respectively, as the acceptor. A variety of polycyclic frameworks imbedding an indene core were generally furnished in moderate to excellent yields with high levels of enantioselectivity by employing a newly designed chiral phosphoramidite ligand.

## Abstract 2

A type of π-allylpalladium complex-containing 10π-cycloaddend generated from 2-methylene-1-indanols under double activation of Pd(0) and phosphoric acid undergoes asymmetric higher-order [10 + 2] cycloadditions with diverse activated alkenes.

## Graphical abstract

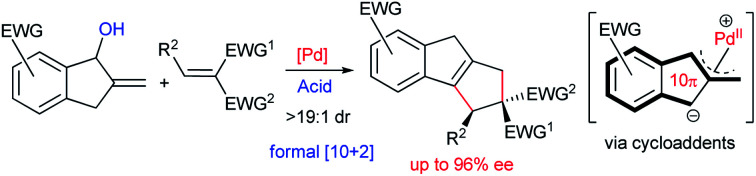

## Introduction

**Scheme 1:**
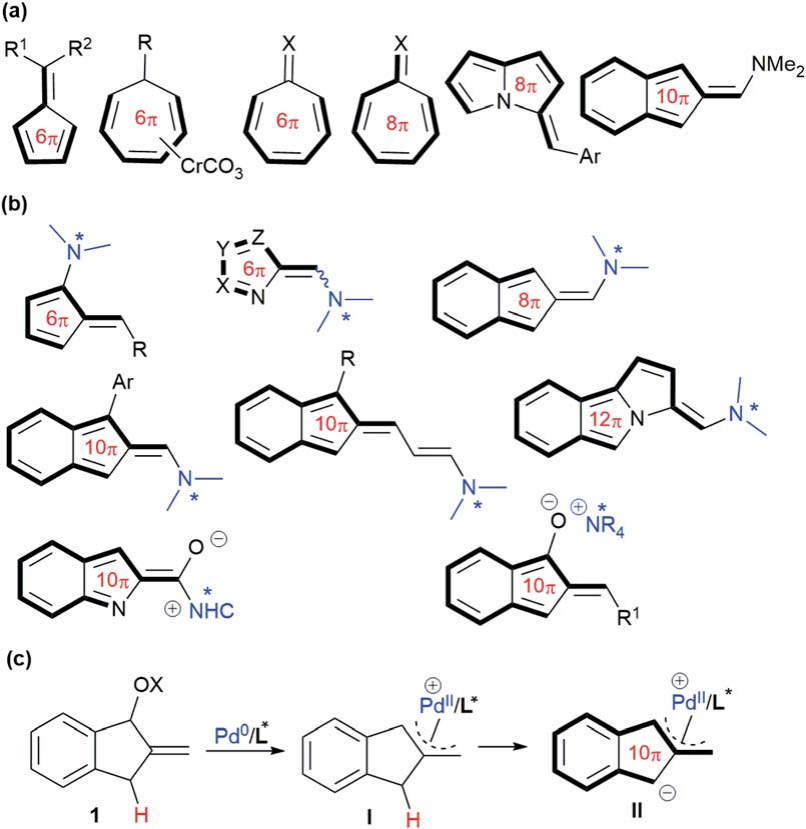
Summary of typical cycloaddends (>4π) used in higher-order cycloadditions and our design. (a) Diverse pre-prepared cycloaddends. (b) Diverse *in situ* formed cycloaddends *via* organocatalysis. (c) This work: *in situ* formed π-allylpalladium-containing 10π-cycloaddends.

Higher-order cycloaddition involving conjugated systems with more than 6π-electrons, which enables rapid construction of complicated cyclic frameworks,^1^ has received considerable attention since its discovery in the 1960s.^2^ In spite of its charm in organic synthesis, higher-order cycloaddition generally suffers from low reactivity, and poor periselectivity and stereoselectivity; thus a variety of pre-prepared cyclic polyenes (>4π) have been commonly utilised, including fulvenes,^3^ Cr(0)-cycloheptatriene complexes,^4^ tropone and its analogues,^5^ and 3*H*-pyrrolizines,^6^ as well as amino-stabilised isobenzofulvenes,^7^ in combination with diverse 2π- or 4π-systems under different conditions (Scheme 1a). Recently, significant progress in the field of higher-order cycloaddition reactions has been made with some well-designed carbonyl substrates, which could be activated by a suitable organocatalyst to generate several types of 6π, 8π, 10π or even 12π-cycloaddends *in situ*, typically featuring HOMO-raised polyenamine^8^ or polyenolate species^9^ (Scheme 1b). However, the development of relevant cycloaddends embedding a reactive metal-complexed motif catalytically, which can be successfully applied in higher-order cycloaddition reactions, has not been disclosed yet.

Over the past few decades, the zwitterionic reagents bearing a π-allylmetal moiety, usually generated *in situ* from allylic alcohol derivatives under palladium or iridium catalysis, have been extensively utilised as valuable 1,*n*-dipoles for various asymmetric formal cycloaddition reactions.^10^ However, such a reaction strategy, through designing suitable conjugated π-systems, has not been envisioned for potentially developing higher-order cycloadditions.^11^ As a result, it would be particularly intriguing to uncover a new type of metal-embedding dipole, which could participate in asymmetric higher-order cycloadditions as cycloaddends with more than 6π-electrons. With these considerations, we envisaged that allylic alcohol or its derivative 1, readily available from 1-indanone, would undergo oxidative addition under Pd(0) catalysis. The resultant π-allylpalladium complex I, having an indene-type structure,^12^ would feasibly increase the acidity of the benzylic C–H.^13^ Thus, zwitterionic intermediate II would be generated after deprotonation, as outlined in Scheme 1c, which might perform as a unique metal-containing 10π-cycloaddend to undertake higher-order [10 + *n*] cycloaddition reactions with suitable electrophilic counterparts.

## Results and discussion

### Condition optimisation

The initial attempt with allylic carbonate 1a and activated alkene α-cyano chalcone 2a proved to be unsuccessful in toluene at 50 °C under the catalysis of Pd(PPh_3_)_4_ (Table 1, entry 1). It was speculated that enhancing the acidity of the benzylic C–H, by introducing an electron-withdrawing group at the indane ring, would be helpful for the formation of the desired dipole intermediate. Pleasingly, 6-nitro-substituted substrate 1b showed good reactivity under the identical catalytic conditions. The expected formal [10 + 2] cycloadduct 3a′ was detected, whereas product 3a, after isomerisation of the double bond, was found to be the more thermally stable one. Moreover, by simple treatment with catalytic amounts of Et_3_N in one pot, pure 3a was efficiently isolated in a moderate yield with excellent diastereoselectivity (entry 2). We next explored the asymmetric version by utilising Pd_2_(dba)_3_ and chiral ligands. Unfortunately, only moderate enantioselectivity was attained with phosphoramidite L1 after extensively screening diverse ligands (entry 3).^14^ We turned to explore the reaction by using a new substrate and a new catalytic system. Compared to allylic carbonates or esters, free allylic alcohols represent more atom-economic and environmentally benign precursors for generating the corresponding π-allyl species under transition metal catalysis, as water is the sole side product.^15^ Nevertheless, alcohol 1c suffered from low reactivity under the catalysis of Pd(0) due to the poor leaving ability of the hydroxyl group (entry 4). Subsequently, we tried to add a phosphoric acid as a co-catalyst, as it has been demonstrated to be beneficial for the oxidative addition of Pd(0) towards allylic alcohols and also might be beneficial for enantiocontrol.^16^ To our gratification, the cycloaddition of 1c and 2a proceeded smoothly by using BINOL-derived (*R*)-A1 as an additive and PPh_3_ as the ligand, and 3a was afforded in a moderate yield albeit with low enantioselectivity (entry 5). It should be noted that such a dehydration strategy has not been previously utilised in Pd-based dipole chemistry.^10^ Moreover, it also suggested that acid (*R*)-A1 would play dual roles in sequential activation of both substrates 1c and 2a. Consequently, some chiral ligands were investigated in combination with (*R*)-A1. Chiral phosphoramidites L1 and L2 showed moderate reactivity but still with low enantiocontrol (entries 6 and 7). While the newly designed phosphoramidite L3 gave fair enantioselectivity (entry 8), (*R*)-BINOL derived L4, a diastereomer of L3, exhibited much higher enantioselectivity (entry 9). Moreover, an improved yield with a better ee value was obtained by using the combination of acid (*S*)-A1 and L4 (entry 10). Nevertheless, ligand L5 with a smaller TES group delivered reduced enantioselectivity (entry 11). A slightly higher yield was attained at 30 °C (entry 12). It was further found that the chiral acid was not necessary, and even higher catalytic activity and exclusive regioselectivity with retained enantioselectivity was achieved with simple phosphoric acid A2 (entry 13).

**Table 1:** Optimisation of reaction conditions of the asymmetric [10 + 2] cycloaddition reaction^a^

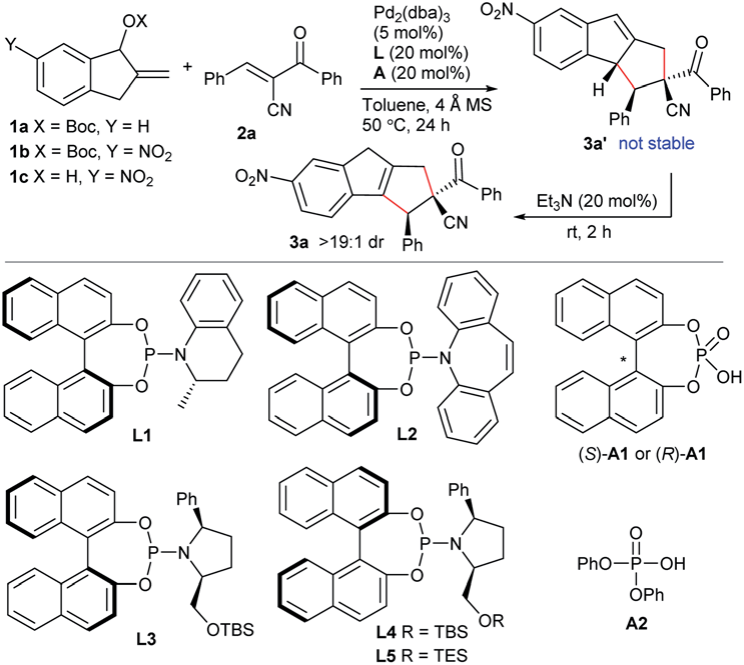					
Entry	1	L	A	Yield^b^ (%)	ee^c^ (%)
1^d^	1a	—	—	—	—
2^d^	1b	—	—	65	—
3	1b	L1	—	45	−65
4	1c	PPh_3_	—	<5%	—
5	1c	PPh_3_	(*R*)-A1	65	−12
6	1c	L1	(*R*)-A1	40	−5
7	1c	L2	(*R*)-A1	65	−25
8	1c	L3	(*R*)-A1	65	−30
9	1c	L4	(*R*)-A1	60	86
10	1c	L4	(*S*)-A1	68	91
11	1c	L5	(*S*)-A1	75	76
12^e^	1c	L4	(*S*)-A1	72	91
13^e^	1c	L4	A2	99	91

a Unless noted otherwise, reactions were performed with substrate 1 (0.1 mmol), α-cyano chalcone 2a (0.12 mmol), Pd_2_(dba)_3_ (5 mol%), L (20 mol%), additive (20 mol%) and 4 Å MS (100 mg) in toluene (1 mL) at 50 °C under Ar for 24 h. After completion, Et_3_N (20 mol%) was added, and the mixture was stirred at rt for 2 h.

b Yield of isolated product 3a.

c Determined by HPLC analysis on a chiral stationary phase, and dr > 19 : 1 by ^1^H NMR analysis.

d Pd(PPh_3_)_4_ (10 mol%) was used.

e At 30 °C.

### Substrate investigation of asymmetric [10 + 2] cycloadditions

Subsequently, we explored the substrate scope and limitations of the asymmetric formal [10 + 2] cycloaddition reaction under the cooperative catalysis of Pd/L4 and phosphoric acid A2. As summarised in Table 2, an array of α-cyano chalcones 2 were first tested in the reactions with benzocyclopentenol 1c. Acceptors 2 with different aromatic substituents at the β-position, including electron-deficient and -rich ones, smoothly gave corresponding products 3a–3j in moderate to good yields with high stereoselectivity (Table 2, entries 1–10). The one with a *p*-nitrophenyl group showed low reactivity under the standard conditions, but desired product 3e was obtained in a good yield with excellent enantioselectivity by using (*S*)-A1 as the additive (entry 5). Notably, the halogen-substituted ones were compatible with the reactions (entries 2–4). Similarly good results were generally produced for substrates 2 with diverse α′-aroyl groups (entries 11–18), whereas (*S*)-A1 was found to be helpful in some cases. Unfortunately, the activated alkenes with aliphatic substituents failed to afford the cycloadducts, probably because of the undesired acidic vinylogous C–H of these substrates.^14^ On the other hand, benzocyclopentenols 1 with a nitro group at different positions on the phenyl ring were applied in the reactions with 2a. When the one with a 4-nitro substituent was utilised, cycloadduct 3s was afforded in good yield with good enantioselectivity (entry 19).^14^ Nevertheless, the alcohol with a 5-nitro group also delivered product 3a, same as that from 1c (6-nitro), indicating an isomerisation process of the 10π-intermediate would be involved (entry 20).^14^ In addition, a 6-cyano-substituted alcohol showed comparable reactivity, and the expected cycloadduct 3t was attained quantitatively with high enantioselectivity (entry 21). We also conducted the reaction on a larger scale, and similar good results were afforded (entry 22).

**Table 2:** Substrate scope of asymmetric [10 + 2] cycloadditions between benzocyclopentenols 1 and α-cyano chalcones 2^a^

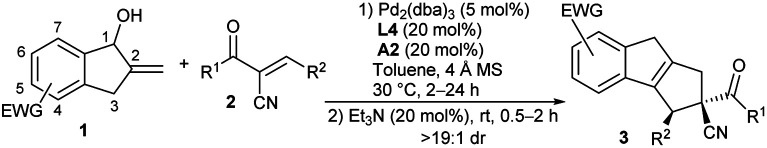				
Entry	EWG	R^1^, R^2^	Yield^b^ (%)	ee^c^ (%)
1	6-NO_2_	Ph, Ph	3a, 99	91^d^
2	6-NO_2_	Ph, 2-ClC_6_H_4_	3b, 96	93
3^e^	6-NO_2_	Ph, 3-ClC_6_H_4_	3c, 89 (80)	82 (92)
4	6-NO_2_	Ph, 4-BrC_6_H_4_	3d, 67	89
5^e^	6-NO_2_	Ph, 4-NO_2_C_6_H_4_	3e, — (70)	— (90)
6	6-NO_2_	Ph, 2-MeOC_6_H_4_	3f, 99	95
7^e^	6-NO_2_	Ph, 3-MeOC_6_H_4_	3g, 99 (75)	87 (93)
8	6-NO_2_	Ph, 4-MeOC_6_H_4_	3h, 99	88
9	6-NO_2_	Ph, 2-naphthyl	3i, 99	91
10	6-NO_2_	Ph, 2-thienyl	3j, 70	88
11^e^	6-NO_2_	2-BrC_6_H_4_, Ph	3k, — (73)	— (91)
12^e^	6-NO_2_	3-BrC_6_H_4_, Ph	3l, — (75)	— (82)
13	6-NO_2_	4-ClC_6_H_4_, Ph	3m, 99	93
14	6-NO_2_	4-BrC_6_H_4_, Ph	3n, 76	94
15^e^	6-NO_2_	2-MeC_6_H_4_, Ph	3o, 86 (90)	83 (89)
16^e^	6-NO_2_	3-MeC_6_H_4_, Ph	3p, 88 (72)	82 (91)
17	6-NO_2_	4-MeC_6_H_4_, Ph	3q, 99	92
18	6-NO_2_	2-Naphthyl, Ph	3r, 91	90
19	4-NO_2_	Ph, Ph	3s, 99	89
20	5-NO_2_	Ph, Ph	3a, 61	88
21	6-CN	Ph, Ph	3t, 99	89
22^f^	6-NO_2_	Ph, Ph	3a, 91	88

a Unless noted otherwise, reactions were performed with allylic alcohol 1 (0.1 mmol), activated alkene 2 (0.12 mmol), Pd_2_(dba)_3_ (5 mol%), L4 (20 mol%), acid A2 (20 mol%) and 4 Å MS (100 mg) in toluene (1 mL) at 30 °C under Ar for 2–24 h. After completion, Et_3_N (20 mol%) was added, and the mixture was stirred at rt for 0.5–2 h.

b Yield of the isolated product.

c Determined by HPLC analysis on a chiral stationary phase; dr > 19 : 1 by ^1^H NMR analysis.

d The absolute configuration of enantiopure 3a was determined by X-ray analysis. The other products were assigned by analogy.

e Data in parentheses were obtained with acid (*S*)-A1 (20 mol%).

f On a 1.0 mmol scale.

### More substrate exploration

Apart from α-cyano chalcones, we successfully extended the asymmetric [10 + 2] cycloaddition reactions to other types of activated alkenes for constructing polycyclic frameworks with more structural diversity. It was found that barbiturate-derived alkenes 4 could be well assembled with alcohol 1c under the standard catalytic conditions. As summarised in Table 3, alkenes 4 with a different aryl or heteroaryl substituent underwent the cycloaddition reaction smoothly, furnishing corresponding spirocyclic architectures 5a–5e in moderate to good yields with high stereoselectivity (entries 1–7). Besides, the one with a 2-styryl group also worked well, and product 5h was obtained in moderate yield and enantioselectivity (entry 8). (*S*)-A1 was further tested when the reactions did not work well (entries 2 and 7).

**Table 3:** Substrate scope of asymmetric [10 + 2] cycloadditions between benzocyclopentenol 1c and barbiturate-derived alkenes 4^a^

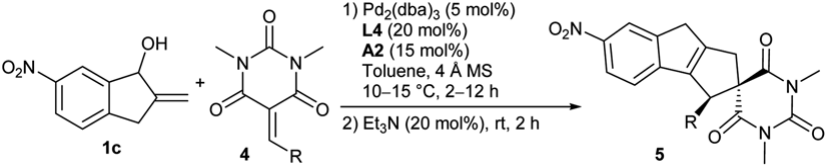			
Entry	R	Yield^b^ (%)	ee^c^ (%)
1	Ph	5a, 99	93
2^d^	4-BrC_6_H_4_	5b, — (60)	— (94)
3	4-MeC_6_H_4_	5c, 99	93
4	4-MeOC_6_H_4_	5d, 99	80
5	2-Naphthyl	5e, 75	93
6	2-Furyl	5f, 89	96
7^d^	2-Thienyl	5g, 76 (64)	80 (87)
8	2-Styryl	5h, 91	80

a Unless noted otherwise, reactions were performed with allylic alcohol 1c (0.1 mmol), alkene 4 (0.12 mmol), Pd_2_(dba)_3_ (0.005 mmol, 5 mol%), L4 (20 mol%), A2 (15 mol%) and 4 Å MS (100 mg) in toluene (1 mL) at 10–15 °C for 2–12 h under Ar. After completion, Et_3_N (20 mol%) was added, and the mixture was stirred at rt for 2 h.

b Yield of the isolated product.

c Determined by HPLC analysis on a chiral stationary phase.

d Data in parentheses were obtained with acid (*S*)-A1 (20 mol%).

**Scheme 2:**
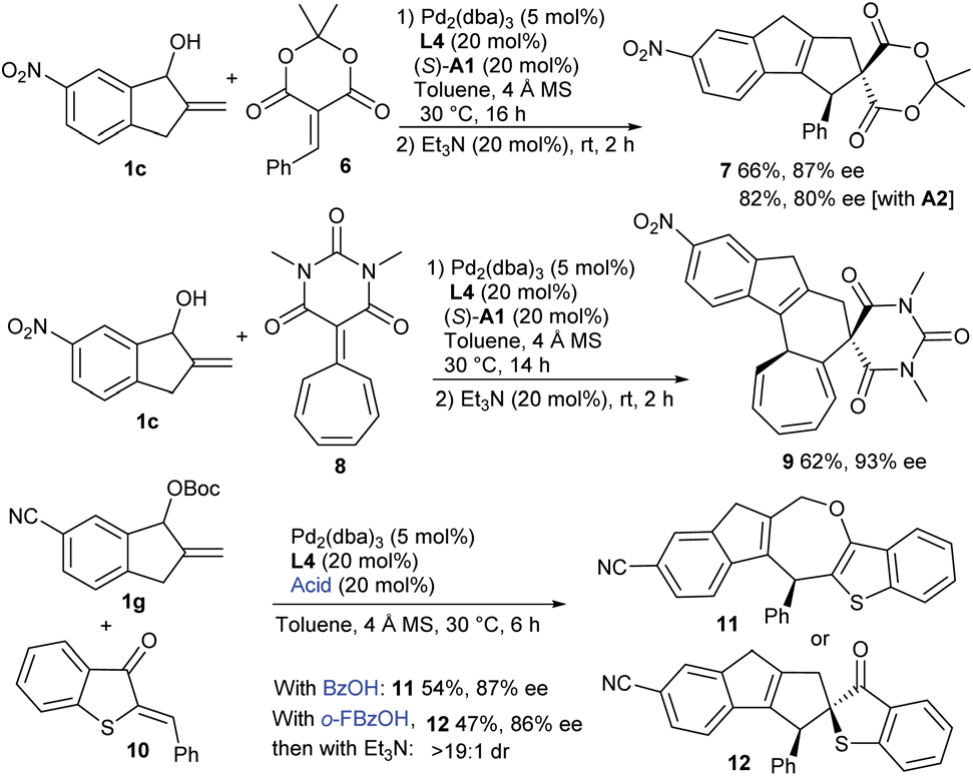
More exploration of higher-order cycloadditions.

Moreover, the formal [10 + 2] cycloaddition reaction could be extended to benzylidene Meldrum's acid 6 by using the combination of Pd/L4 and (*S*)-A1, delivering product 7 in a moderate yield with high enantioselectivity, whereas a higher yield with lower enantiocontrol was observed with acid A2 (Scheme 2). Interestingly, when barbiturate–heptafulvene 8 was employed,^17^ an asymmetric [10 + 8] higher-order cycloaddition reaction was applicable, and polycyclic 9 was constructed in a moderate yield with excellent enantiocontrol. Moreover, the assembly of carbonate 1g and 2-benzylidenebenzo[*b*]thiophen-3(2*H*)-one 10 was successful under the cooperative catalysis of Pd/L4 and benzoic acid, and [10 + 4] cycloadduct 11 embedding an oxepine motif was isolated in a moderate yield with high enantiocontrol, along with the observation of minor [10 + 2] product 12. Interestingly, using *o*-fluorobenzoic acid as the additive, cycloadduct 12 with similar enantiocontrol was delivered as the major one after the treatment with Et_3_N, albeit in a slightly lower yield.^14^

### Mechanistic proposal

**Scheme 3:**
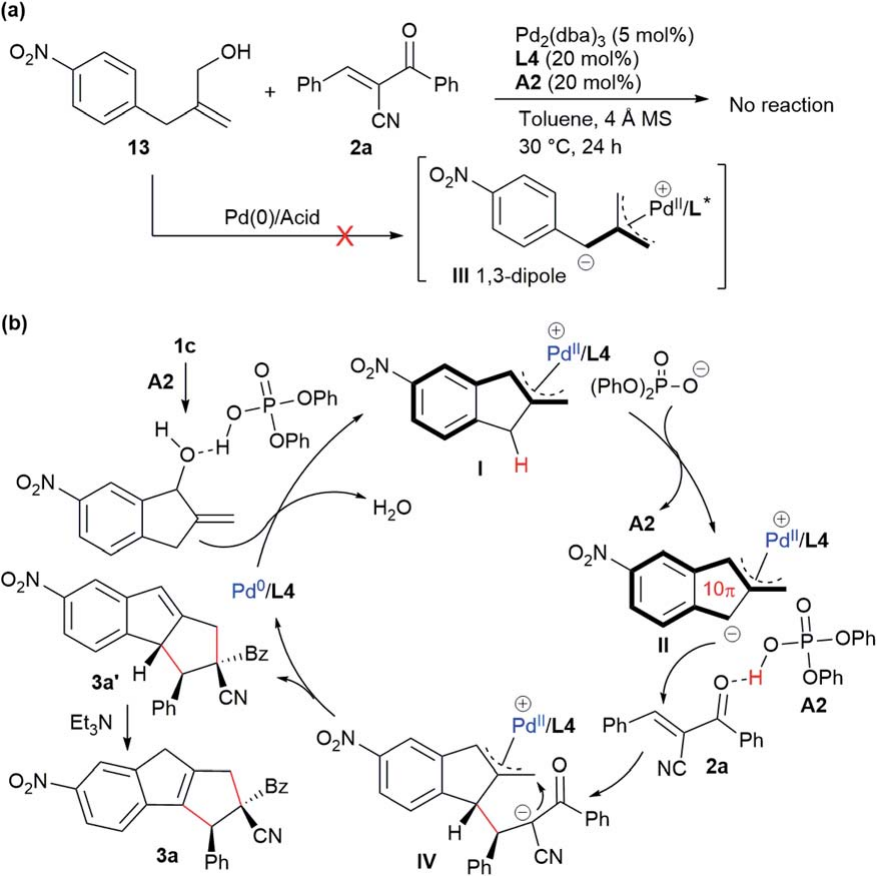
Mechanistic proposal. (a) Control experiment. (b) Proposed catalytic mechanism.

To gain more insight into the mechanism, a control experiment with the analogous acyclic alcohol 13 was conducted. As shown in Scheme 3a, no reaction occurred in combination with acceptor 2a in the presence of Pd/L4 and acid A2, which suggested that expected zwitterionic intermediate III might not be generated *via* a similar oxidative addition/deprotonation process under the standard conditions.^18^ These results indicated that the indene-based structure was crucial for the vinylogous activation of the benzylic C–H group to generate the active dipole species. Therefore, as outlined in Scheme 3b, it was proposed that allylic alcohol 1c would be partially protonated in the presence of phosphoric acid A2 and undergo oxidative addition with complex Pd(0)/L4 along with the release of H_2_O. The π-allylpalladium complex moiety of the resultant intermediate I would further enhance the acidity of the benzylic C–H group, and a deprotonation process would occur to give dipole II, which would more reasonably exist as a polyconjugated 10π-type cycloaddend. Subsequently, acid A2 would act as a Brønsted acid to activate α-cyano chalcone 2a, rendering the assembly with dipole II to deliver adduct IV. An intramolecular allylic alkylation would be followed to afford 3a′ together with the regeneration of Pd(0). Finally, an isomerisation process took place with the assistance of Et_3_N to furnish the thermally more stable product 3a.

## Conclusions

A new type of metal-containing 10π-cycloaddend could be generated from benzocyclopentenols with an electron-withdrawing group *via* a deprotonation strategy under the cooperative catalysis of Pd(0) and phosphoric acid. The subsequent asymmetric higher-order [10 + 2] cycloaddition reactions with diverse activated alkenes were efficiently accomplished by employing a newly designed phosphoramidite ligand, producing polycyclic frameworks embedding an indene core in moderate to excellent yields with good to excellent enantioselectivity. In addition, [10 + 8] and [10 + 4] higher-order cycloaddition reactions were similarly realised with a heptafulvene derivative or a cyclic enone substrate, respectively. The newly designed metal-containing 10π-synthons would have more potential in asymmetric reactions, and the results will be reported in due course.

## Data availability

The data that support the findings of this study are available in the ESI† or on request from the corresponding author.

## Author contributions

The manuscript was written through contributions of all authors. All authors have given approval to the final version of the manuscript.

## Conflicts of interest

There are no conflicts to declare.

## Supplementary Material

SC-013-D2SC02985E-s001

SC-013-D2SC02985E-s002
