# Supplementary material for: Asymmetric higher-order [10 + n] cycloadditions of palladium-containing 10π-cycloaddends
Source: Chem Sci. 2022 Jul 15;13(32):9265–70. doi: 10.1039/d2sc02985e (PMC9384823; doi:10.1039/d2sc02985e)
Supplement: SC-013-D2SC02985E-s001 [file SC-013-D2SC02985E-s001.pdf]

Electronic Supplementary Material (ESI) for Chemical Science.
This journal is © The Royal Society of Chemistry 2022

Asymmetric higher-order [10+n] cycloadditions of palladium-containing 10π-cycloaddends

Ao Li,a,‡ Yang Gao,a,‡ Jian-Bin Lu,a Zhi-Chao Chen,a Wei Du,*a and Ying-Chun Chen*a,b

[a] Key Laboratory of Drug-Targeting and Drug Delivery System of the Education Ministry and Sichuan Province, and Sichuan Research Center for Drug Precision Industrial Technology, West China School of Pharmacy, Sichuan University, Chengdu 610041, China.

[b] College of Pharmacy, Third Military Medical University, Shapingba, Chongqing 400038, China.

‡ A. Li and Y. Gao equally contributed to this work.

E-mail: duweiyb.scu.edu.cn; ycchen@scu.edu.cn

Supplementary Information

Table of Contents

1. General methods **S1**

2. Procedure for preparation of allylic alcohol and carbonate 1 .... **S2**

3. Procedure for preparation of ligand L4 .... **S7**

4. Detailed screening conditions for asymmetric [10+n] cycloadditions **S8**

5. General procedure for asymmetric cycloadditions **S13**

6. More screening studies for substrate scope **S40**

7. Asymmetric reaction on a 1.0 mmol scale **S44**

8. Crystal data and structural refinement for enantiopure 3a **S45**

9. Mechanism study **S47**

10. References **S50**

11. NMR, HRMS spectra and HPLC chromatograms **S51**

1. General methods

Unless otherwise noted, all reactions were carried out at ambient temperature; when the reactions required heating, the heat source was oil bath. ^{1}H NMR (400 or 600 MHz), ^{13}C NMR (100 or 150 MHz) and ^{19}F NMR (376 MHz) spectra were recorded on Varian INOVA-400/54, Agilent DD2-600/54 or Bruker Ascend^{TM} 400 instruments (Chemical shifts were reported in ppm from tetramethylsilane with the solvent resonance as the internal standard in $CDCl_3$ solution, unless otherwise noted). The following abbreviations were used to explain the multiplicities: s = singlet, d = doublet, t = triplet, dd = double doublet, ddd = double double doublet, dt = double triplet; td = triple doublet; tt = triple triplet, m = multiplet, br = broad, and coupling constants (*J*) are reported in Hertz (Hz). High resolution mass spectra (HRMS) were recorded on a Waters SYNAPT G2, Agilent G1969-85000 or Shimadzu LCMS-IT-TOF using a time-of-flight mass spectrometer equipped with electrospray ionization (ESI) source. X-ray diffraction experiments were carried out on an Agilent Gemini or Bruker APEX-Ⅱ CCD diffractometer and the data obtained were deposited at the Cambridge Crystallographic Data Centre. In each case, diastereomeric ratio was determined by ^{1}H NMR analysis and enantiomeric excess was determined by HPLC (Agilent Technologies: 1220 Infinity II, 1200 Series, 1260 Infinity) analysis on a chiral column in comparison with an authentic racemate, using a Daicel Chiralpak AD-H Column (250 × 4.6 mm), Chiralpak IE (250 × 4.6 mm) or Chiralpak IA Column (250 × 4.6 mm). UV detection was monitored at 254 nm. The specific optical rotation was obtained from Rudolph Research Analytical Autopol I automatic polarimeter in $CHCl_3$ solution at 25 °C. The melting points were obtained from WRX-4 Mel-Temp apparatus. Column chromatography was performed on silica gel (200–300 mesh) eluting with ethyl acetate (EtOAc) and petroleum ether. TLC was performed on glass-backed silica plates. UV light, I_2, and solution of potassium permanganate were used to visualize products or starting materials. All chemicals were used without purification as commercially available unless otherwise noted. Petroleum ether (60–90 °C) was redistilled. (*S*)-(+)-1,1'-Binaphthyl-2,2'-diyl phosphate (*S*)-**A1** and diphenyl phosphate **A2** were passed over a column of silica to remove water. 2-Methylene-2,3-dihydro-1*H*-inden-1-one **S1**,[1] 5-nitro-1-indanone **S7**,[2] α-cyano chalcones **2**,[3] barbiturate-derived alkenes **4**,[4] benzylidene Meldrum's acid **6**,[5] barbiturate-heptafulvene **8**,[6] 2-benzylidenebenzo[*b*]thiophen-3(2*H*)-one **10**,[7] and (2*S*,5*R*)-2-(((tert-butyldimethylsilyl)oxy) methyl)-5-phenylpyrrolidine **S13**[8] were prepared according to the literature procedures.

2. Procedure for preparation of allylic alcohol and carbonate 1

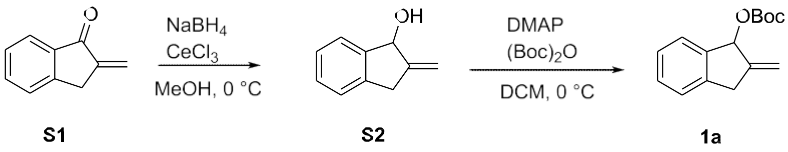

$CeCl_3$ (0.222 g, 0.901 mmol, 1.0 equiv) was added to a stirred solution of **S1** (0.130 g, 0.902 mmol, 1.0 equiv) in MeOH (2 mL) at 0 °C. After 10 min, $NaBH_4$ (34.0 mg, 0.900 mmol, 1.0 equiv) was added, and the mixture was stirred for 0.2 h. Water (5 mL) was added and the resultant slurry was filtered through Celite and washed with Et_2O (15 mL). The combined filtrates were washed with saturated $NaHCO_3$ (2 × 20 mL), brine (2 × 20 mL) and dried over anhydrous Na_2SO_4. After concentration, the residue was purified by flash chromatography on silica gel (EtOAc/petroleum ether = 1/5) to afford **S2 (**0.123 g, 84% yield) as a colorless oil.

DMAP (9.8 mg, 0.080 mmol, 0.2 equiv) was added to a stirred solution of **S2** (60.0 mg, 0.411 mmol, 1.0 equiv) in DCM (5 mL). $(Boc)_2O$ (0.140 mL, 0.620 mmol, 1.5 equiv) was added dropwise at 0 °C, and the mixture was stirred for 0.2 h. After completion, the mixture was concentrated and purified by chromatography on silica gel (EtOAc/petroleum ether = 1/15) to afford **1a** (18.1 mg, 18% yield) as a yellow oil.

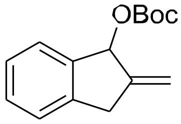

***tert*-Butyl (2-methylene-2,3-dihydro-1*H*-inden-1-yl) carbonate (1a)**: Colorless oil; **^{1}H NMR** (400 MHz, $CDCl_3$): δ (ppm) 7.43 (d, J = 7.2 Hz, 1H), 7.35 (d, J = 7.6 Hz, 1H), 7.29–7.21 (m, 1H), 7.22–7.13 (m, 1H), 6.83 (s, 1H), 5.00 (s, 2H), 3.44 (s, 2H), 1.50 (s, 9H); **^{13}C NMR** (100 MHz, $CDCl_3$): δ (ppm) 153.5, 144.2, 143.5, 142.7, 130.7, 126.4, 124.9, 123.7, 121.2, 82.3, 65.2, 39.4, 27.8; **HRMS** (ESI-TOF) m/z: $[M + Na]^+$ Calcd for $C_{15}H_{18}O_3Na^+$ 269.1148; Found 269.1140.

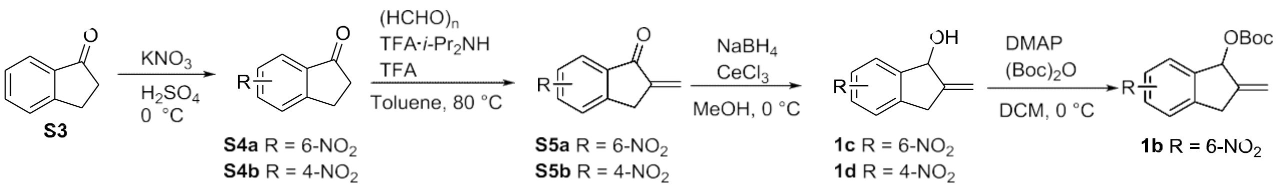

A solution of potassium nitrate (8.79 g, 86.9 mmol, 2.25 equiv) in concentrated sulfuric acid (50 mL) was added dropwise to the solution of **S3** (5.15 g, 39.0 mmol, 1.0 equiv) in concentrated sulfuric acid (30 mL) at 0 °C over 0.5 h. The mixture was stirred at 0 °C for 1 h. Then the mixture was poured into ice water (300 mL) and extracted with EtOAc (3 × 50 mL). After the removal of solvent, the crude product was purified by flash chromatography on silica gel (EtOAc/petroleum ether = 1/5) to afford **S4a** (2.99 g, 43% yield) and **S4b** (1.31 g, 19% yield) as a brown solid.

To a suspension of **S4a** (2.20 g, 12.4 mmol, 1.0 equiv), $(HCHO)_n$ (1.49 g, 49.6 mmol, 4.0 equiv) and TFA·*i*-Pr_2NH (2.67 g, 12.4 mmol, 1.0 equiv) in toluene (60 mL) was added TFA (0.090 mL, 1.2 mmol, 0.1 equiv). The mixture was stirred at 80 °C. After completion, the mixture was cooled to room temperature and filtered through Celite. The cake was washed with EtOAc (3 × 20 mL). The combined filtrates were washed with saturated $NaHCO_3$ (2 × 30 mL), brine (2 × 30 mL) and dried over anhydrous Na_2SO_4. After concentration, the residue was purified by flash chromatography on silica gel (EtOAc/petroleum ether = 1/5) to afford **S5a** (1.60 g, 68% yield) as a white solid. **S5b** was prepared similarly from **S5b**.

$CeCl_3$ (0.389 g, 1.58 mmol, 1.0 equiv) was added to a stirred and cooled (0 °C) solution of **S5a** (0.300 g, 1.59 mmol, 1.0 equiv) in MeOH (5 mL). After 10 min, $NaBH_4$ (0.0597 g, 1.58 mmol, 1.0 equiv) was added, and the mixture was stirred at 0 °C for 0.2 h. Water (5 mL) was added and the resultant slurry was filtered through Celite and washed with Et_2O (25 mL). The combined filtrates were washed with saturated $NaHCO_3$ (2 × 20 mL), brine (2 × 20 mL) and dried over anhydrous Na_2SO_4. After concentration, the residue was purified by flash chromatography on silica gel (EtOAc/petroleum ether = 1/5) to afford **1c** (0.209 g, 69% yield) as a white solid. **1d** was prepared similarly from **S5b**.

DMAP (12.7 mg, 0.104 mmol, 0.2 equiv) was added to a stirred solution of **1c** (0.100 mg, 0.523 mmol, 1.0 equiv) in DCM (7 mL). $(Boc)_2O$ (0.180 mL, 0.784 mmol, 1.5 equiv) was added dropwise at 0 °C, and the mixture was stirred for 0.2 h. After completion, the mixture was concentrated and purified by chromatography on silica gel (EtOAc/petroleum ether = 1/15) to afford **1b** (0.136 g, 90% yield) as a colorless oil.

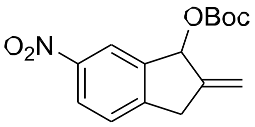

***tert*-Butyl (2-methylene-6-nitro-2,3-dihydro-1*H*-inden-1-yl) carbonate (1b)**: Yellow oil; **^{1}H NMR** (600 MHz, $CDCl_3$): δ (ppm) 8.35 (s, 1H), 8.21 (d, J = 8.4 Hz, 1H), 7.41 (d, J = 8.4 Hz, 1H), 6.43 (s, 1H), 5.63 (s, 1H), 5.45 (s, 1H), 3.89 (d, J = 20.4 Hz, 1H), 3.67 (d, J = 21.0 Hz, 1H), 1.54 (s, 9H); **^{13}C NMR** (150 MHz, $CDCl_3$): δ (ppm) 153.4, 149.9, 145.2, 141.6, 125.4, 124.8, 121.5, 115.5, 83.1, 78.8, 36.8, 27.8, 27.4; **HRMS** (ESI-TOF) m/z: $[M + Na]^+$ Calcd for $C_{15}H_{17}NO_5Na^+$ 314.0999; Found 314.0990.

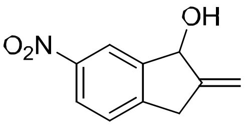

2-Methylene-6-nitro-2,3-dihydro-1*H*-inden-1-ol (1c): White solid, mp: 59–61 °C; **^{1}H NMR** (400 MHz, $CDCl_3$): δ (ppm) 8.28 (d, J = 2.4 Hz, 1H), 8.13 (dd, J = 8.4, 2.4 Hz, 1H), 7.38 (d, J = 8.4 Hz, 1H), 5.55–5.53 (m, 2H), 5.35–5.33 (m, 1H), 3.80–3.65 (m, 2H), 2.43 (s, 1H); **^{13}C NMR** (100 MHz, $CDCl_3$): δ (ppm) 151.1, 148.3, 147.7, 145.7, 125.4, 124.0, 120.3, 111.6, 75.7, 36.6; **HRMS** (ESI-TOF) m/z: $[M + H]^+$ Calcd for $C_{10}H_{10}NO_3^+$ 192.0661; Found 192.0652.

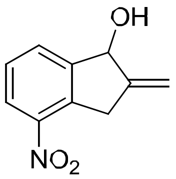

2-Methylene-4-nitro-2,3-dihydro-1*H*-inden-1-ol (1d): Yellow solid, mp: 53–55 °C; **^{1}H NMR** (400 MHz, $CDCl_3$): δ (ppm) 8.15 (d, J = 8.4 Hz, 1H), 7.78 (d, J = 7.2 Hz, 1H), 7.49–7.44 (m, 1H), 5.55–5.52 (m, 2H), 5.40–5.38 (m, 1H), 4.21–4.01 (m, 2H), 2.25 (s, 1H); **^{13}C NMR** (100 MHz, $CDCl_3$): δ (ppm) 150.8, 147.7, 137.3, 131.0, 128.4, 124.4, 111.6, 75.6, 37.8; **HRMS** (ESI-TOF) m/z: $[M + H]^+$ Calcd for $C_{10}H_{10}NO_3^+$ 192.0661; Found 192.0670.

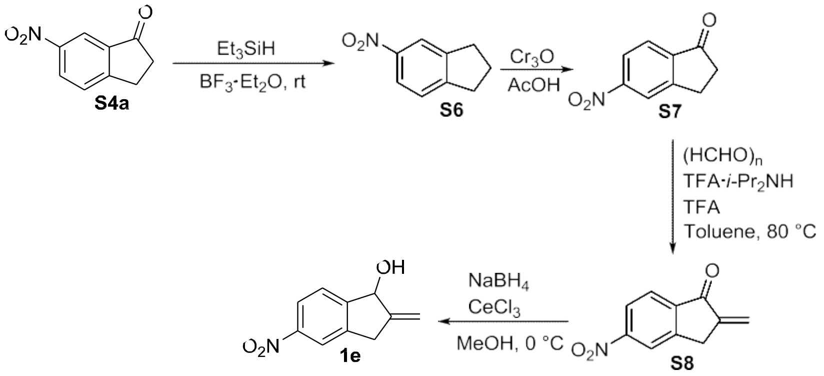

S4a (3.54 g, 20.0 mmol, 1.0 equiv) and Et_3SiH (12.7 mL, 79.7 mmol, 4.0 equiv) were added into $BF_3\cdot Et_2O$ (48%, 50 mL) at room temperature and the solution was stirred for 8 h. After completion, it was quenched with water (100 mL) and extracted with EtOAc (2 × 20 mL). The organic layer was dried over anhydrous Na_2SO_4 and concentrated in vacuo. Crude product **S6** was obtained as a yellow solid and used without further purification.

To a solution of **S6** (0.196 g, 1.20 mmol, 1.0 equiv) in AcOH (50 mL) was added CrO_3 (6.00 g, 60.0 mmol, 5.0 equiv), and the mixture was stirred at 50 °C for 30 h. Then the mixture was poured into water (200 mL) and extracted with EtOAc (2 × 50 mL). The organic layer was dried over anhydrous Na_2SO_4. After concentration, the residue was purified by flash chromatography on silica gel (EtOAc/petroleum ether = 1/5) to afford **S7** (0.766 g, 36% yield) as a yellow solid.

To a suspension of **S7** (0.640 g, 3.61 mmol, 1.0 equiv), $(HCHO)_n$ (0.434 g, 14.5 mmol, 4.0 equiv) and TFA· *i*-Pr_2NH (0.744 g, 3.60 mmol, 1.0 equiv) in toluene (30 mL) was added TFA (0.030 mL, 0.40 mmol, 0.1 equiv). The mixture was stirred at 80 °C for 6 h. The mixture was cooled to room temperature and filtered through Celite. The cake was washed with EtOAc (3 × 10 mL). The combined filtrates were washed with saturated $NaHCO_3$ (2 × 20 mL), brine (2 × 20 mL) and dried over anhydrous Na_2SO_4. After concentration, the residue was purified by flash chromatography on silica gel (EtOAc/petroleum ether = 1/10) to afford **S8** (0.381 g, 56% yield) as a yellow solid.

$CeCl_3$ (0.493 g, 2.00 mmol, 1.0 equiv) was added to a stirred solution of **S8** (0.381 g, 2.02 mmol, 1.0 equiv) in MeOH (5 mL) at 0 °C. After 15 min, $NaBH_4$ (0.0740 g, 1.96 mmol, 1.0 equiv) was added, and the mixture was stirred for 0.2 h. Water (5 mL) was added and the resultant slurry

was filtered through Celite and washed with Et_2O (15 mL). The combined filtrates were washed with saturated $NaHCO_3$ (2 × 20 mL), brine (2 × 20 mL) and dried over anhydrous Na_2SO_4. After concentration, the residue was purified by flash chromatography on silica gel (EtOAc/petroleum ether = 1/10) to afford **1e** (0.211 g, 55% yield) as a white semisolid.

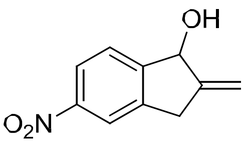

2-Methylene-5-nitro-2,3-dihydro-1*H*-inden-1-ol (1e): White semisolid; **^{1}H NMR** (400 MHz, $CDCl_3$): δ (ppm) 8.13–8.07 (m, 2H), 7.59 (d, J = 8.4 Hz, 1H), 5.52 (s, 2H), 5.34 (s, 1H), 3.77–3.67 (m, 2H), 2.38 (s, 1H); **^{13}C NMR** (100 MHz, $CDCl_3$): δ (ppm) 151.1, 151.0, 148.4, 142.3, 125.6, 122.8, 120.0, 111.4, 75.7, 36.3; **HRMS** (ESI-TOF) m/z: $[M + Na]^+$ Calcd for $C_{10}H_9NO_3Na^+$ 214.0475; Found 214.0470.

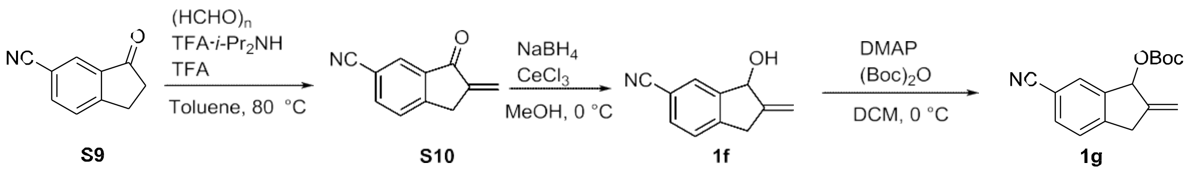

To a suspension of **S9** (0.314 g, 2.00 mmol, 1.0 equiv), $(HCHO)_n$ (0.240 g, 7.99 mmol, 4.0 equiv) and TFA·*i*-Pr_2NH (0.430 g, 2.00 mmol, 1.0 equiv) in toluene (10 mL) was added TFA (0.015 mL, 0.20 mmol, 0.1 equiv). The mixture was stirred at 80 °C for 6 h. The mixture was cooled to room temperature and filtered through Celite. The cake was washed with EtOAc (3 × 20 mL), and the combined filtrates were washed with saturated $NaHCO_3$ (2 × 30 mL), brine (2 × 30 mL) and dried over anhydrous Na_2SO_4. After concentration, the residue was purified by flash chromatography on silica gel (EtOAc/petroleum ether = 1/5) to afford **S10** (0.280 g, 83% yield) as a white solid.

$CeCl_3$ (0.409 g, 1.66 mmol, 1.0 equiv) was added to a stirred solution of **S10** (0.280 g, 1.66 mmol, 1.0 equiv) in MeOH (5 mL) at 0 °C. After 10 min, $NaBH_4$ (0.614 g, 1.62 mmol, 1.0 equiv) was added, and the mixture was stirred for 0.2 h. Water (5 mL) was added and the resultant slurry was filtered through Celite and washed with Et_2O (15 mL). The combined filtrates were washed with saturated $NaHCO_3$ (2 × 20 mL), brine (2 × 20 mL) and dried over anhydrous Na_2SO_4. After concentration, the residue was purified by flash chromatography on silica gel (EtOAc/petroleum ether = 1/5) to afford **1f (**0.115 g, 39% yield) as a white solid.

DMAP (4.3 mg, 0.035 mmol, 0.1 equiv) was added to a stirred solution of **1f** (30.0 mg, 0.175 mmol, 1.0 equiv) in DCM (2 mL). $(Boc)_2O$ (65.0 μL, 0.283 mmol, 1.5 equiv) was added dropwise at 0 °C, and the mixture was stirred for 0.2 h. After completion, the mixture was concentrated and purified by chromatography on silica gel (EtOAc/petroleum ether = 1/15) to afford **1g** (47.1 mg, 99% yield) as a white solid.

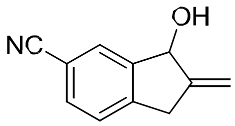

3-Hydroxy-2-methylene-2,3-dihydro-1*H*-indene-5-carbonitrile (1f): White solid, mp: 75–76 °C; **^{1}H NMR** (400 MHz, $CDCl_3$): δ (ppm) 7.73 (s, 1H), 7.55 (dd, J = 8.0, 1.2 Hz, 1H), 7.36–7.34 (m, 1H), 5.53–5.51 (m, 1H), 5.48 (s, 1H), 5.34–5.32 (m, 1H), 3.79–3.59 (m, 2H), 2.35 (s, 1H); **^{13}C NMR** (100 MHz, $CDCl_3$): δ (ppm) 151.0, 146.3, 145.4, 132.4, 128.9, 125.6, 119.1, 111.4, 110.9, 75.7, 36.8; **HRMS** (ESI-TOF) m/z: $[M + Na]^+$ Calcd for $C_{11}H_9NONa^+$ 194.0582; Found 194.0584.

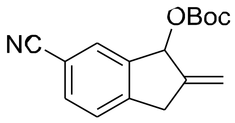

***tert*-Butyl (6-cyano-2-methylene-2,3-dihydro-1*H*-inden-1-yl) carbonate (1g)**: White solid, mp: 89–90 °C; **^{1}H NMR** (600 MHz, $CDCl_3$): δ (ppm) 7.79 (s, 1H), 7.59 (d, J = 6.0 Hz, 1H), 7.36 (d, J = 6.0 Hz, 1H), 6.37 (s, 1H), 5.60 (s, 1H), 5.43 (s, 1H), 3.86 (d, J = 18.0 Hz, 1H), 3.63 (d, J = 18.0 Hz, 1H), 1.53 (s, 9H); **^{13}C NMR** (150 MHz, $CDCl_3$): δ (ppm) 153.5, 148.1, 145.0, 141.4, 133.2, 130.1, 125.7, 118.8, 115.4, 111.1, 83.0, 79.0, 37.0, 27.8; **HRMS** (ESI-TOF) m/z: $[M + Na]^+$ Calcd for $C_{16}H_{17}NO_3Na^+$ 294.1101; Found 294.1109.

3. Procedure for preparation of ligand L4

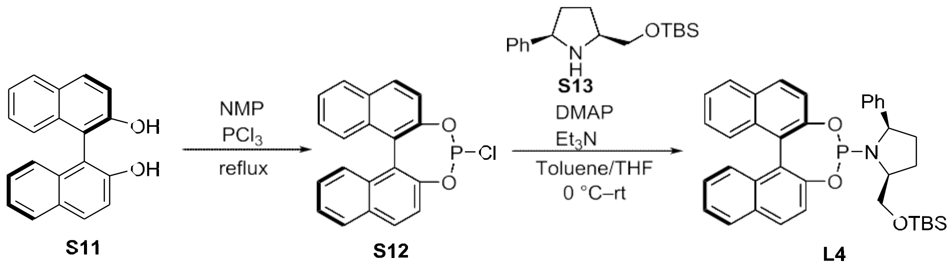

A 25 mL Schlenk flask was charged with **S11** (0.570 g, 1.99 mmol), phosphorus trichloride (1.75 mL, 20.1 mmol, 10.0 equiv) and 1-methyl-2-pyrrolidinone (2.0 µL, 0.021 mmol) under argon. The mixture was heated to 90 °C for 30 min, then the volatiles was evaporated under vacuum and further coevaporat with dry toluene three times to afford crude product **S12** as an orange solid and crude product **S12** was used directly in the next step.

A 25 mL round-bottom flask was charged with **S12** (0.292 g, 1.00 mmol, 1.0 equiv), Et_3N (0.690 mL, 4.97 mmol, 5.0 equiv), DMAP (24.4 mg, 0.200 mmol, 0.2 equiv) and toluene (1 mL). **S13** (0.525 g, 1.50 mmol, 1.5 equiv) was dissolved in THF (1 mL) and the mixture was transferred to the former flask at 0 °C under argon. The mixture was stirred at rt for 6 h. The solid was removed by filtration. The filtrate was concentrated and purified by flash column chromatography (EtOAc/petroleum ether = 1/50) to afford **L4** (0.301 g, 50% yield) as a white semisolid.

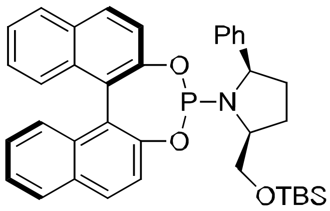

(2*S*,5*R*)-2-(((*tert*-Butyldimethylsilyl)oxy)methyl)-1-(dinaphtho[2,1-*d*:1',2'-*f*][1,3,2]dioxaphosphepin-4-yl)-5-phenylpyrrolidine (L4): White semisolid; **^{1}H NMR** (400 MHz, $CDCl_3$): δ (ppm) 7.94 (d, J = 9.2 Hz, 1H), 7.91–7.85 (m, 3H), 7.48 (d, J = 8.8 Hz, 1H), 7.42–7.40 (m, 6H), 7.36–7.37 (m, 3H), 7.26–7.23 (m, 3H), 4.90–4.83 (m, 1H), 3.65 (dd, J = 6.0, 4.0 Hz, 1H), 3.31 (t, J = 9.6 Hz, 1H), 3.20 (s, 1H), 2.32–2.25 (m, 1H), 1.89–1.82 (m, 2H), 1.80–1.76 (m, 1H), 0.53 (s, 9H), –0.32 (s, 3H), –0.57 (s, 3H); **^{13}C NMR** (100 MHz, $CDCl_3$): δ (ppm) 150.4, 150.3, 149.6, 144.5, 132.80, 132.76, 131.4, 130.8, 130.2, 129.8, 128.4, 128.2, 127.1, 126.6, 126.0, 124.8, 124.5, 122.3, 122.0, 121.5, 65.6, 62.5, 62.3, 60.3, 34.9, 34.8, 28.4, 25.5, 17.8, –5.5, –5.9; **HRMS** (ESI-TOF) m/z: $[M + H]^+$ Calcd for $C_{37}H_{41}NO_3PSi^+$ 606.2593; Found 606.2604.

4. Detailed screening conditions for asymmetric [10+n] cycloadditions

Table S1. Asymmetric [10+2] cycloaddition of allyl *tert*-butyl carbonate 1b with alkene 2a[a]

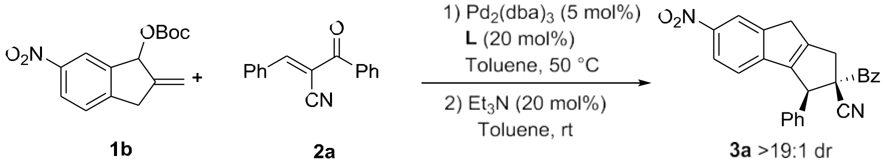

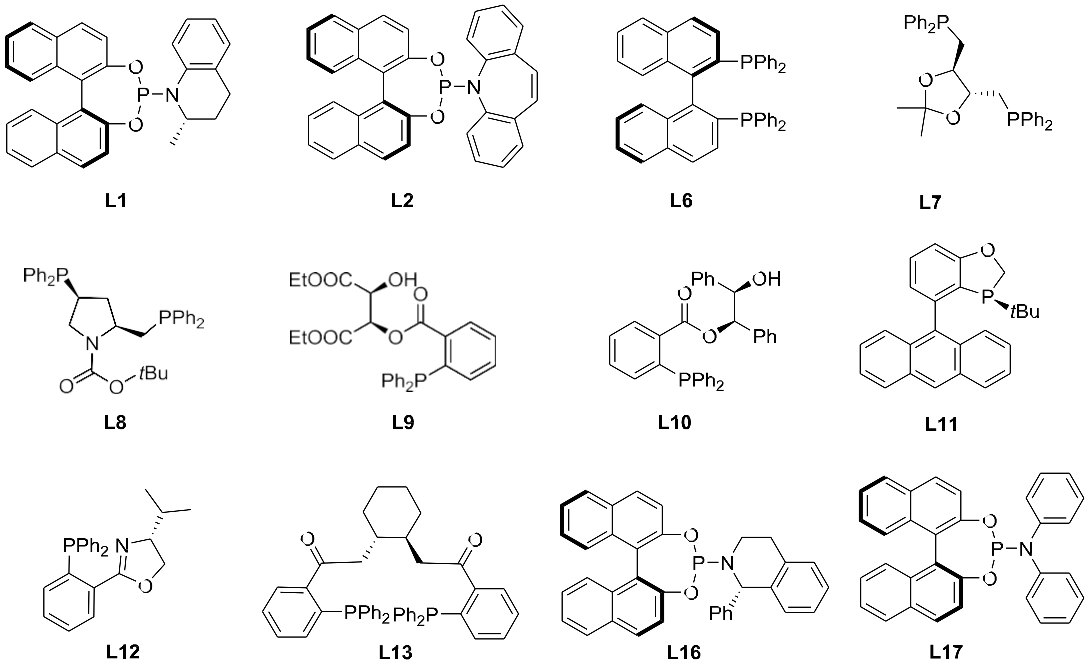

Entry	L	Yield of **3a** (%)[b]	ee of **3a** (%)[c]
1	**L1**	45	–65
2	**L2**	57	30
3	**L6**	<5	/
4	**L7**	45	0
5	**L8**	48	–12
6	**L9**	48	–7
7	**L10**	47	–17
8	**L11**	<5	/
9	**L12**	<5	/
10	**L13**	<5	/
11	**L16**	54	–18
12	**L17**	42	–23

[a] Reactions were performed with allylic alcohol **1b** (0.1 mmol), alkene **2a** (0.12 mmol), $Pd_2(dba)_3$ (5 mol%) and **L** (20 mol%) in toluene (1 mL) at 50 °C under Ar for 24 h. After completion, Et_3N (20 mol%) was added and the mixture was stirred at rt for 2 h. [b] Yield of isolated product. [c] Determined by HPLC analysis on a chiral stationary phase.

Table S2. Asymmetric [10+2] cycloaddition of allylic alcohol 1c with alkene 2a[a]

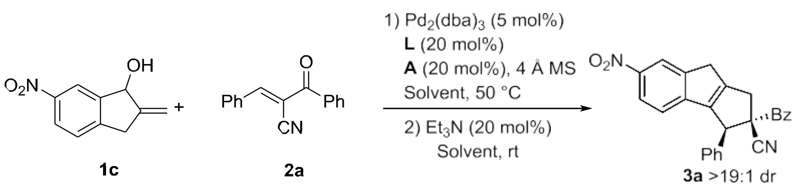

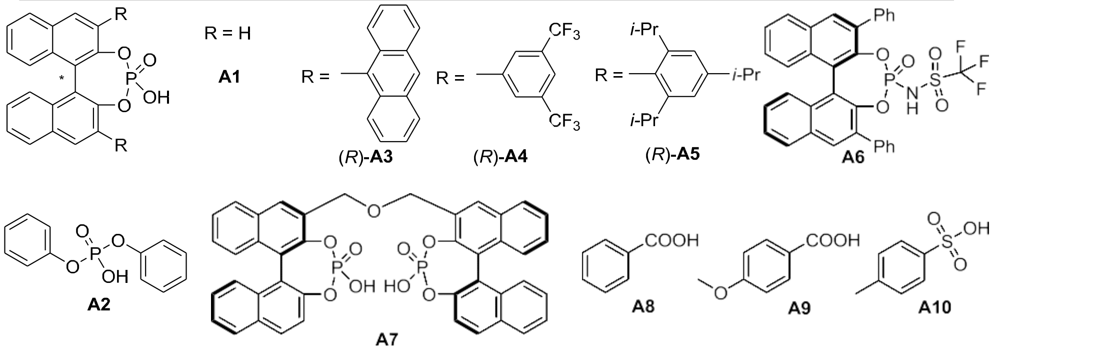

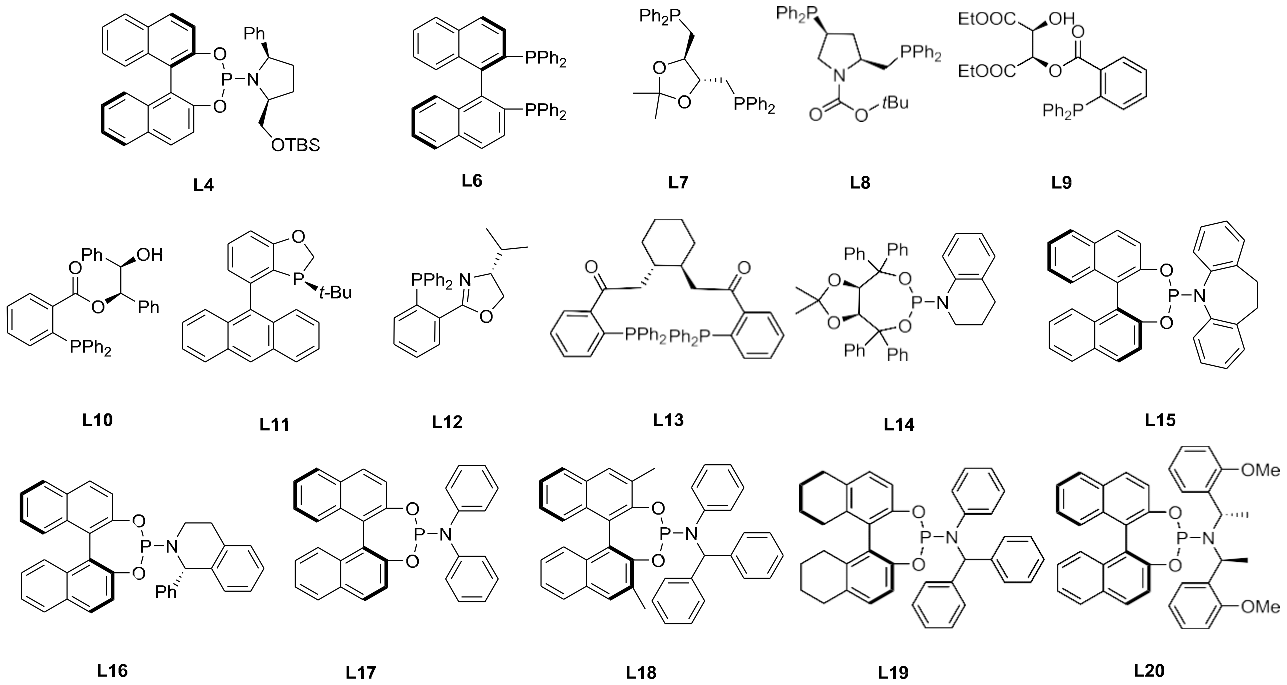

Entry	Solvent	L	A	Yield of **3a** (%)[b]	ee of **3a** (%)[c]
1	Toluene	PPh_3	(*R*)-**A1**	65	–12
2	Toluene	PPh_3	(*R*)-**A3**	40	–7
3	Toluene	PPh_3	(*R*)-**A4**	30	–8
4	Toluene	PPh_3	(*R*)-**A5**	50	–9
5	Toluene	PPh_3	**A6**	<5	/
6	Toluene	PPh_3	**A7**	<5	/
7	Toluene	PPh_3	/	<5	/
8	Toluene	**L4**	(*R*)-**A1**	60	86
9	Toluene	**L6**	(*R*)-**A1**	<5	/
10	Toluene	**L7**	(*R*)-**A1**	40	0
11	Toluene	**L8**	(*R*)-**A1**	40	–10
12	Toluene	**L9**	(*R*)-**A1**	50	–8

13	Toluene	**L10**	(*R*)-**A1**	45	–14
14	Toluene	**L11**	(*R*)-**A1**	<5	/
15	Toluene	**L12**	(*R*)-**A1**	<5	/
16	Toluene	**L13**	(*R*)-**A1**	35	–15
17	Toluene	**L14**	(*R*)-**A1**	<5	/
18	Toluene	**L15**	(*R*)-**A1**	60	27
19	Toluene	**L16**	(*R*)-**A1**	35	–15
20	Toluene	**L17**	(*R*)-**A1**	40	–28
21	Toluene	**L18**	(*R*)-**A1**	30	0
22	Toluene	**L19**	(*R*)-**A1**	45	–46
23	Toluene	**L20**	(*R*)-**A1**	40	18
24	Toluene	**L4**	(*S*)-**A1**	68	91
25	THF	**L4**	(*S*)-**A1**	35	82
26	$CHCl_3$	**L4**	(*S*)-**A1**	55	85
27	DCM	**L4**	(*S*)-**A1**	52	86
28[d]	Toluene	**L4**	(*S*)-**A1**	70	91
29[e]	Toluene	**L4**	(*S*)-**A1**	72	91
30[f]	Toluene	**L4**	(*S*)-**A1**	72	91
31[e,g]	Toluene	**L4**	(*S*)-**A1**	70	90
32[e,h]	Toluene	**L4**	(*S*)-**A1**	65	91
33[e,i]	Toluene	**L4**	(*S*)-**A1**	68	90
34[e,j]	Toluene	**L4**	(*S*)-**A1**	72	91
35[e]	Toluene	**L4**	**A8**	70	80
36[e]	Toluene	**L4**	**A9**	72	80
37[e]	Toluene	**L4**	**A10**	<5	/
38[e]	Toluene	**L4**	TFA	<5	/
39[e]	Toluene	**L4**	**A2**	99	91

[a] Unless noted otherwise, reactions were performed with allylic alcohol **1c** (0.1 mmol), alkene **2a** (0.12 mmol), $Pd_2(dba)_3$ (5 mol%), **L** (20 mol%), **A** (20 mol%) and 4 Å MS (100 mg) in toluene (1 mL) at 50 °C under Ar for 24 h. After completion, Et_3N (20 mol%) was added and the mixture was stirred at rt for 2 h. [b] Yield of isolated product. [c] Determined by HPLC analysis on a chiral stationary phase. [d] At 40 °C. [e] At 30 °C. [f] At rt for 36 h. [g] With 3 Å MS (100 mg). [h] With 5 Å MS (100 mg). [i] With 4 Å MS (50 mg). [j] With 4 Å MS (150 mg).

Table S3. Asymmetric [10+2] cycloaddition of allylic alcohol 1c with alkene 4a[a]

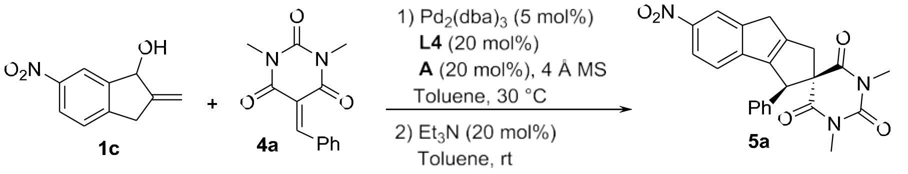

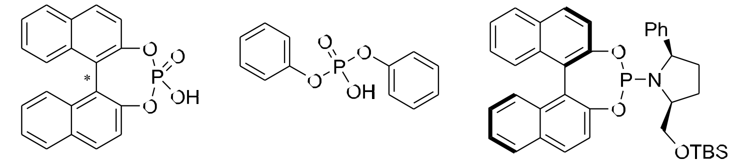

A1 **A2** **L4**

Entry	**A**	Solvent	Yield (%)[b]	ee (%)[c]
1	(*S*)-**A1**	Toluene	65	93
2	(*R*)-**A1**	Toluene	73	85
3	(*S*)-**A1**	THF	57	80
4	(*S*)-**A1**	$CHCl_3$	60	77
5	(*S*)-**A1**	DCM	45	72
6[d]	(*S*)-**A1**	Toluene	62	90
7[e]	(*S*)-**A1**	Toluene	60	88
8[f,g]	**A2**	Toluene	99	93

[a] Unless noted otherwise, reactions were performed with allylic alcohol **1c** (0.1 mmol), alkene **4a** (0.12 mmol), $Pd_2(dba)_3$ (5 mol%), **L4** (20 mol%), **A** (20 mol%) and 4 Å MS (100 mg) in toluene (1 mL) at 30 °C under Ar for 12 h. After completion, Et_3N (20 mol%) was added and the mixture was stirred at rt for 2 h. [b] Yield of isolated product. [c] Determined by HPLC analysis on a chiral stationary phase. [d] At 40 °C. [e] At 50 °C. [f] At 15 °C. [g] Data in parentheses were obtained with acid **A2** (15 mol%).

Table S4. Asymmetric [10+4] and [10+2] cycloaddition of allyl *tert*-butyl carbonate 1g with 2-benzylidenebenzo[*b*]thiophen-3(2*H*)-one 10[a]

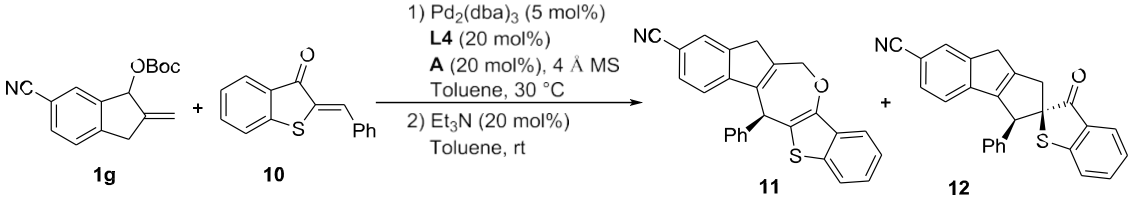

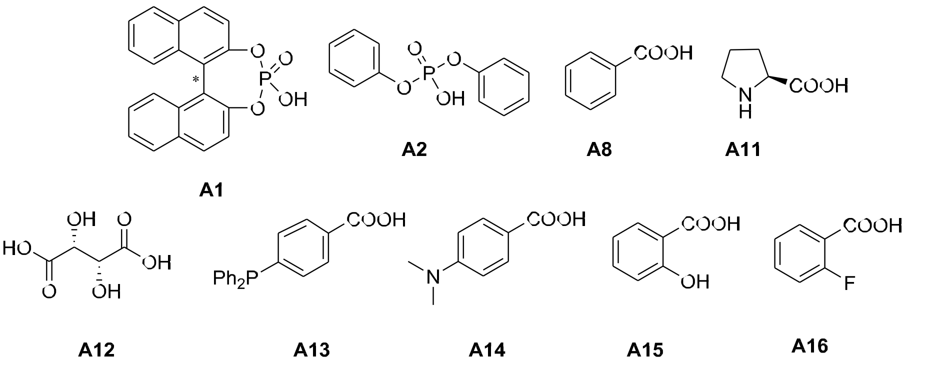

Entry	A	Yield of **11**(%)[b]	ee of **11** (%)[c]	Yield of **12**(%)[b]	ee of **12** (%)[c]
1	/	60	61	<20	/
2	(*S*)-**A1**	<5	/	<5	/
3	(*R*)-**A1**	<5	/	<5	/
4	**A2**	33	46	<20	/
5	**A8**	54	87	<20	/
6	**A11**	45	33	<20	/
7	**A12**	49	60	<20	/
8	**A13**	60	65	<20	/
9	**A14**	49	85	<20	/
10	**A15**	51	77	<20	/
11	**A16**	<20	/	47	86
12[d]	**A8**	<20	/	<20	/

[a] Unless noted otherwise, reactions were performed with allylic alcohol **1g** (0.1 mmol), 2-benzylidenebenzo[*b*]thiophen-3(2*H*)-one **10** (0.12 mmol), $Pd_2(dba)_3$ (5 mol%), **L4** (20 mol%), **A** (20 mol%) and 4 Å MS (100 mg) in toluene (1 mL) at 30 °C under Ar for 24 h. After completion, Et_3N (20 mol%) was added and the mixture was stirred at rt for 2 h. [b] Yield of isolated product. [c] Determined by HPLC analysis on a chiral stationary phase. [d] At 15 °C.

5. General procedure for asymmetric cycloadditions

5.1 Asymmetric [10+2] cycloadditions of allylic alcohol 1 with alkenes 2

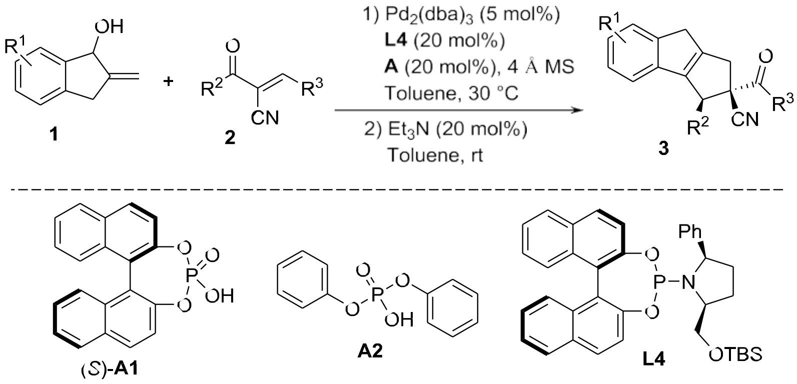

A flame-dried 10 mL Schlenk tube equipped with a magnetic stirring bar was cooled to room temperature. To this flask were added $Pd_2(dba)_3$ (0.0050 mmol), **L4** (0.020 mmol), acid **A** (0.020 mmol) and 4 Å MS (100.0 mg), and the flask was evacuated and back-filled three times with argon. Then toluene (0.5 mL) was added by syringe. The mixture was stirred at 30 °C for 10 min. To another dry tube equipped with a magnetic stirring bar were added **1** (0.10 mmol) and **2** (0.12 mmol), evacuated and back-filled three times with argon. Then toluene (0.5 mL) was added by syringe. The mixture was stirred at rt for 5 min. Then the latter solution was added to the first Schlenk tube by syringe. The mixture was stirred at 30 °C for 2–24 h. After completion, Et_3N (0.020 mmol) was added to the mixture and stirred at rt for 0.5–2 h. After completion, the solvent was evaporated in vacuo. The residue was purified by flash chromatography on silica gel (EtOAc/petroleum ether) to give the product.

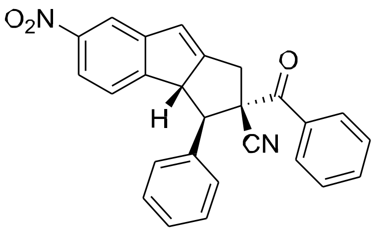

Synthesis of 3a′: A flame-dried 10 mL Schlenk tube equipped with a magnetic stirring bar was cooled to room temperature. To this flask were added $Pd_2(dba)_3$ (4.6 mg, 0.0050 mmol, 0.05 equiv), **L4** (12.1 mg, 0.0200 mmol, 0.2 equiv), **A2** (5.0 mg, 0.020 mmol, 0.2 equiv) and 4 Å MS (100.0 mg), and the flask was evacuated and back-filled three times with argon. Then toluene (0.5 mL) was added by syringe. The mixture was stirred at 30 °C for 10 min. To another dry tube equipped with a magnetic stirring bar were added 2-methylene-6-nitro-2,3-dihydro-1*H*-inden-1-ol **1c** (19.1 mg, 0.100 mmol, 1.0 equiv) and (*E*)-2-benzoyl-3-phenylacrylonitrile **2a** (28.0 mg, 0.120 mmol, 1.2 equiv), evacuated and back-filled three times with argon. Then toluene (0.5 mL) was added by syringe. The mixture was stirred at rt for 5 min. Then the latter solution was added to the first Schlenk tube by syringe. The mixture was stirred at 30 °C for 2 h. After completion, the solvent was evaporated in vacuo. The residue was purified by flash chromatography on silica gel (EtOAc/petroleum ether = 1/8) to give product **3a′**:

40.4 mg, 99% yield, as a yellow solid; mp: 111–113 °C; $[\alpha]^{25}_{D}$ = +76.5 (c = 1.5 in $CHCl_3$); >19:1 dr; 93% ee, determined by HPLC analysis [Chiralpak IA, *n*-hexane/*i*-PrOH = 80/20, 1.0 mL min^{-1}, λ = 254 nm, t (minor) = 15.34 min, t (major) = 18.27 min]; **^{1}H NMR** (400 MHz, $CDCl_3$): *δ* (ppm) 8.12 (d, *J* = 2.4 Hz, 1H), 7.93–7.85 (m, 3H), 7.64 (dd, *J* = 7.6, 2.4 Hz, 2H), 7.59 (t, *J* = 7.6 Hz, 1H), 7.48–7.38 (m, 5H), 7.01 (d, *J* = 8.0 Hz, 1H), 6.64 (s, 1H), 4.70 (d, *J* = 13.6 Hz, 1H), 3.88 (dd, *J* = 17.2, 2.4 Hz, 1H), 3.64 (d, *J* = 13.6 Hz, 1H), 3.40 (d, *J* = 17.2 Hz, 1H); **^{13}C NMR** (100 MHz, $CDCl_3$): *δ* (ppm) 191.0, 154.4, 149.6, 148.9, 148.3, 135.2, 134.3, 133.6, 129.5, 129.3, 128.91, 128.88, 123.8, 123.77, 123.75, 120.4, 120.1, 116.1, 62.9, 57.5, 52.2, 38.6; **HRMS** (ESI-TOF) m/z: $[M + Na]^+$ Calcd for $C_{26}H_{18}N_2O_3Na^+$ 429.1210; Found 429.1218.

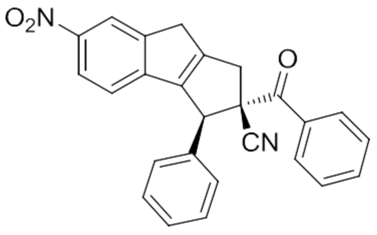

Synthesis of 3a: A flame-dried 10 mL Schlenk tube equipped with a magnetic stirring bar was cooled to room temperature. To this flask were added $Pd_2(dba)_3$ (4.6 mg, 0.0050 mmol, 0.05 equiv), **L4** (12.1 mg, 0.0200 mmol, 0.2 equiv), **A2** (5.0 mg, 0.020 mmol, 0.2 equiv) and 4 Å MS (100.0 mg), and the flask was evacuated and back-filled three times with argon. Then toluene (0.5 mL) was added by syringe. The mixture was stirred at 30 °C for 10 min. To another dry tube equipped with a magnetic stirring bar were added 2-methylene-6-nitro-2,3-dihydro-1*H*-inden-1-ol **1c** (19.1 mg, 0.100 mmol, 1.0 equiv) and (*E*)-2-benzoyl-3-phenylacrylonitrile **2a** (28.0 mg, 0.120 mmol, 1.2 equiv), evacuated and back-filled three times with argon. Then toluene (0.5 mL) was added by syringe. The mixture was stirred at rt for 5 min. Then the latter solution was added to the first Schlenk tube by syringe. The mixture was stirred at 30 °C for 2 h. After completion, Et_3N (2.8 μL, 0.020 mmol, 0.2 equiv) was added to the reaction mixture and stirred at rt for 2 h. After completion, the solvent was evaporated in vacuo. The residue was purified by flash chromatography on silica gel (EtOAc/petroleum ether = 1/8) to give product **3a**: 40.1 mg, 99% yield, as a yellow solid; mp: 124–125 °C; $[\alpha]^{25}_{D}$ = +107.5 (c = 1.5 in $CHCl_3$); >19:1 dr; 91% ee, determined by HPLC analysis [Chiralpak IA, *n*-hexane/*i*-PrOH = 80/20, 1.0 mL min^{-1}, λ = 254 nm, t (minor) = 12.03 min, t (major) = 14.91 min]; **^{1}H NMR** (400 MHz, $CDCl_3$): *δ* (ppm) 8.27 (d, *J* = 2.0 Hz, 1H), 8.19–8.10 (m, 2H), 8.03 (dd, *J* = 8.4, 2.0 Hz, 1H), 7.66 (t, *J* = 7.2 Hz, 1H), 7.56–7.50 (m, 2H), 7.44–7.36 (m, 3H), 7.34–7.26 (m, 2H), 6.81 (d, *J* = 8.4 Hz, 1H), 5.18 (s, 1H), 3.82 (d, *J* = 18.8 Hz, 1H), 3.74–3.59 (m, 2H), 3.51 (dd, *J* = 22.8, 2.8 Hz, 1H); **^{13}C NMR** (100 MHz, $CDCl_3$): *δ* (ppm) 190.6, 153.3, 148.4, 146.2, 145.8, 145.5, 136.0, 134.4, 132.8, 130.0, 129.1, 129.0, 128.93, 128.91, 123.2, 119.7, 119.5, 119.3, 62.4, 53.8, 41.8, 35.7; **HRMS** (ESI-TOF) m/z: $[M + H]^+$ Calcd for $C_{26}H_{18}N_2O_3Na^+$ 429.1210; Found 429.1208.

Synthesis of 3b: A flame-dried 10 mL Schlenk tube equipped with a magnetic stirring bar was cooled to room temperature. To this flask were added $Pd_2(dba)_3$ (4.6 mg, 0.0050 mmol, 0.05 equiv),

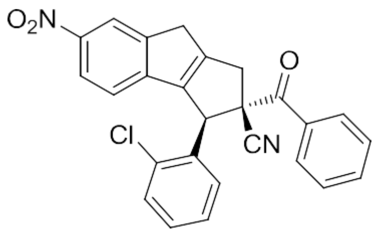

L4 (12.1 mg, 0.0200 mmol, 0.2 equiv), **A2** (5.0 mg, 0.020 mmol, 0.2 equiv) and 4 Å MS (100.0 mg), and the flask was evacuated and back-filled three times with argon. Then toluene (0.5 mL) was added by syringe. The mixture was stirred at 30 °C for 10 min. To another dry tube equipped with a magnetic stirring bar were added 2-methylene-6-nitro-2,3-dihydro-1*H*-inden-1-ol **1c** (19.1 mg, 0.100 mmol, 1.0 equiv) and (*E*)-2-benzoyl-3-(2-chlorophenyl)acrylonitrile **2b** (32.0 mg, 0.120 mmol, 1.2 equiv), evacuated and back-filled three times with argon. Then toluene (0.5 mL) was added by syringe. The mixture was stirred at rt for 5 min. Then the latter solution was added to the first Schlenk tube by syringe. The mixture was stirred at 30 °C for 2 h. After completion, Et_3N (2.8 μL, 0.020 mmol, 0.2 equiv) was added to the reaction mixture and stirred at rt for 2 h. After completion, the solvent was evaporated in vacuo. The residue was purified by flash chromatography on silica gel (EtOAc/petroleum ether = 1/8) to give product **3b**: 42.1 mg, 96% yield, as a yellow solid; mp: 130–131 °C; $[\alpha]_D^{25}$ = +95.2 (*c* = 1.8 in $CHCl_3$); >19:1 dr; 93% ee, determined by HPLC analysis [Chiralpak IA, *n*-hexane/*i*-PrOH = 80/20, 1.0 mL min^{-1}, λ = 254 nm, t (major) = 15.33 min, t (minor) = 16.96 min]; **^{1}H NMR** (400 MHz, $CDCl_3$): *δ* (ppm) 8.29 (d, *J* = 2.0 Hz, 1H), 8.13–8.04 (m, 3H), 7.64 (t, *J* = 7.4 Hz, 1H), 7.55–7.48 (m, 3H), 7.33 (td, *J* = 7.6, 1.6 Hz, 1H), 7.24 (td, *J* = 7.6, 1.6 Hz, 1H), 7.11 (dd, *J* = 8.0, 2.0 Hz, 1H), 6.94 (d, *J* = 8.0 Hz, 1H), 5.81 (s, 1H), 3.77 (s, 2H), 3.64 (d, *J* = 23.6 Hz, 1H), 3.51 (dd, *J* = 22.8, 2.8 Hz, 1H); **^{13}C NMR** (100 MHz, $CDCl_3$): *δ* (ppm) 190.7, 153.4, 148.4, 146.5, 145.7, 145.5, 134.8, 134.3, 134.2, 132.7, 130.2, 130.0, 129.9, 129.8, 128.8, 127.4, 123.3, 119.6, 119.5, 119.4, 60.8, 50.3, 42.8, 35.8; **HRMS** (ESI-TOF) m/z: $[M + Na]^+$ Calcd for $C_{26}H_{17}ClN_2O_3Na^+$ 463.0825 (^{35}Cl) and 465.0796 (^{37}Cl); Found 463.0822 (^{35}Cl) and 465.0800 (^{37}Cl).

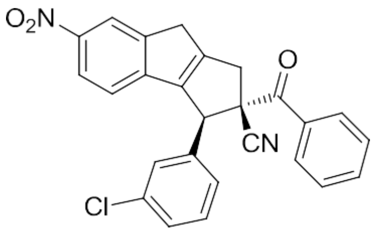

Synthesis of 3c: A flame-dried 10 mL Schlenk tube equipped with a magnetic stirring bar was cooled to room temperature. To this flask were added $Pd_2(dba)_3$ (4.6 mg, 0.0050 mmol, 0.05 equiv), **L4** (12.1 mg, 0.0200 mmol, 0.2 equiv), (*S*)-**A1** (7.0 mg, 0.020 mmol, 0.2 equiv) and 4 Å MS (100.0 mg), and the flask was evacuated and back-filled three times with argon. Then toluene (0.5 mL) was added by syringe. The mixture was stirred at rt for 10 min. To another dry tube equipped with a magnetic stirring bar were added 2-methylene-6-nitro-2,3-dihydro-1*H*-inden-1-ol **1c** (19.1 mg, 0.100 mmol, 1.0 equiv), (*E*)-2-benzoyl-3-(3-chlorophenyl)acrylonitrile **2c** (32.0 mg, 0.120 mmol, 1.2 equiv), evacuated and back-filled three times with argon. Then toluene (0.5 mL) was added by syringe. The mixture was stirred at rt for 5 min. Then the latter solution was added to the first Schlenk tube by syringe. The mixture was stirred at 30 °C for 2 h. After completion, Et_3N (2.8 μL, 0.020 mmol, 0.2 equiv) was

added to the reaction mixture and stirred at rt for 2 h. After completion, the solvent was evaporated in vacuo. The residue was purified by flash chromatography on silica gel (EtOAc/petroleum ether = 1/8) to give product **3c**: 35.2 mg, 80% yield, as a yellow solid; mp: 122–123 °C; $[\alpha]_D^{25}$ = +82.7 (*c* = 1.7 in $CHCl_3$); >19:1 dr; 92% ee, determined by HPLC analysis [Chiralpak IA, *n*-hexane/*i*-PrOH = 80/20, 1.0 mL min^{-1}, λ = 254 nm, t (minor) = 12.75 min, t (major) = 15.10 min]; **^{1}H NMR** (400 MHz, $CDCl_3$): *δ* (ppm) 8.29 (d, *J* = 2.0 Hz, 1H), 8.20–8.09 (m, 2H), 8.07 (dd, *J* = 8.4, 2.4 Hz, 1H), 7.68 (t, *J* = 7.6 Hz, 1H), 7.59–7.51 (m, 2H), 7.42–7.32 (m, 2H), 7.31–7.29 (m, 1H), 7.20 (dt, *J* = 6.8, 1.6 Hz, 1H), 6.85 (d, *J* = 8.4 Hz, 1H), 5.25 (s, 1H), 3.75 (s, 1H), 3.66 (d, *J* = 24.0 Hz, 1H), 3.50 (dd, *J* = 23.2, 3.6 Hz, 1H); **^{13}C NMR** (100 MHz, $CDCl_3$): *δ* (ppm) 190.2, 153.4, 148.3, 145.7, 145.6, 145.5, 138.1, 135.0, 134.6, 132.7, 130.4, 130.0, 129.2, 129.1, 129.0, 127.3, 123.3, 119.6, 119.4, 119.3, 62.4, 52.8, 42.1, 35.8; **HRMS** (ESI-TOF) m/z: $[M + H]^+$ Calcd for $C_{26}H_{18}ClN_2O_3^+$ 441.1006 (^{35}Cl) and 443.0976 (^{37}Cl); Found 441.1000 (^{35}Cl) and 443.0976 (^{37}Cl).

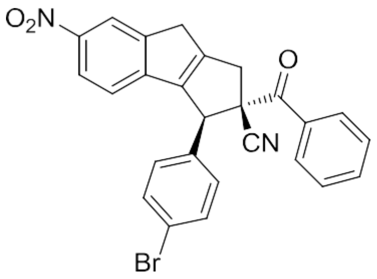

Synthesis of 3d: A flame-dried 10 mL Schlenk tube equipped with a magnetic stirring bar was cooled to room temperature. To this flask were added $Pd_2(dba)_3$ (4.6 mg, 0.0050 mmol, 0.05 equiv), **L4** (12.1 mg, 0.0200 mmol, 0.2 equiv), **A2** (5.0 mg, 0.020 mmol, 0.2 equiv) and 4 Å MS (100.0 mg), and the flask was evacuated and back-filled three times with argon. Then toluene (0.5 mL) was added by syringe. The mixture was stirred at 30 °C for 10 min. To another dry tube equipped with a magnetic stirring bar were added 2-methylene-6-nitro-2,3-dihydro-1*H*-inden-1-ol **1c** (19.1 mg, 0.100 mmol, 1.0 equiv) and (*E*)-2-benzoyl-3-(4-bromophenyl)acrylonitrile **2d** (37.3 mg, 0.120 mmol, 1.2 equiv), evacuated and back-filled three times with argon. Then toluene (0.5 mL) was added by syringe. The mixture was stirred at rt for 5 min. Then the latter solution was added to the first Schlenk tube by syringe. The mixture was stirred at 30 °C for 12 h. After completion, Et_3N (2.8 μL, 0.020 mmol, 0.2 equiv) was added to the reaction mixture and stirred at rt for 2 h. After completion, the solvent was evaporated in vacuo. The residue was purified by flash chromatography on silica gel (EtOAc/petroleum ether = 1/8) to give product **3d**: 32.5 mg, 67% yield, as a yellow solid; mp 118–119 °C; $[\alpha]_D^{25}$ = +86.6 (*c* = 1.5 in $CHCl_3$); >19:1 dr; 89% ee, determined by HPLC analysis [Chiralpak IA, *n*-hexane/*i*-PrOH = 80/20, 1.0 mL min^{-1}, λ = 254 nm, t (minor) = 13.89 min, t (major) = 20.92 min]; **^{1}H NMR** (400 MHz, $CDCl_3$): *δ* (ppm) 8.29 (d, *J* = 2.0 Hz, 1H), 8.16–8.06 (m, 2H), 8.06 (dd, *J* = 8.4, 2.0 Hz, 1H), 7.70–7.67 (m, 1H), 7.66–7.52 (m, 4H), 7.19 (d, *J* = 8.4 Hz, 2H), 6.82 (d, *J* = 8.4 Hz, 1H), 5.25 (d, *J* = 1.2 Hz, 1H), 3.74 (s, 2H), 3.68–3.47 (m, 2H); **^{13}C NMR** (100 MHz, $CDCl_3$): *δ* (ppm) 190.3, 153.3, 148.3, 145.8, 145.6, 145.5, 135.0, 134.6, 132.7, 132.3, 130.8, 130.0, 129.0, 123.2, 123.0, 119.6, 119.5, 119.3, 62.5, 52.8, 42.1, 35.8; **HRMS** (ESI-TOF) m/z: $[M + H]^+$

Calcd for $C_{26}H_{18}BrN_2O_3^+$ 485.0501 (^{79}Br) and 487.0480 (^{81}Br); Found 485.0509 (^{79}Br) and 487.0473 (^{81}Br).

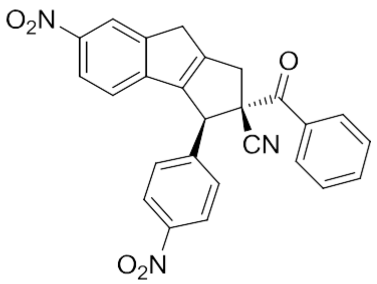

Synthesis of 3e: A flame-dried 10 mL Schlenk tube equipped with a magnetic stirring bar was cooled to room temperature. To this flask were added $Pd_2(dba)_3$ (4.6 mg, 0.0050 mmol, 0.05 equiv), **L4** (12.1 mg, 0.0200 mmol, 0.2 equiv), (*S*)-**A1** (7.0 mg, 0.020 mmol, 0.2 equiv) and 4 Å MS (100.0 mg), and the flask was evacuated and back-filled three times with argon. Then toluene (0.5 mL) was added by syringe. The mixture was stirred at 30 °C for 10 min. To another dry tube equipped with a magnetic stirring bar were added 2-methylene-6-nitro-2,3-dihydro-1*H*-inden-1-ol **1c** (19.1 mg, 0.100 mmol, 1.0 equiv) and (*E*)-2-benzoyl-3-(4-nitrophenyl)acrylonitrile **2e** (33.4 mg, 0.120 mmol, 1.2 equiv), evacuated and back-filled three times with argon. Then toluene (0.5 mL) was added by syringe. The mixture was stirred at rt for 5 min. Then the latter solution was added to the first Schlenk tube by syringe. The mixture was stirred at 30 °C for 24 h. After completion, Et_3N (2.8 μL, 0.020 mmol, 0.2 equiv) was added to the reaction mixture and stirred at rt for 2 h. After completion, the solvent was evaporated in vacuo. The residue was purified by flash chromatography on silica gel (EtOAc/petroleum ether = 1/7) to give product **3e**: 31.5 mg, 70% yield, as a yellow solid; mp: 121–122 °C; $[\alpha]_D^{25}$ = +93.9 (*c* = 1.6 in $CHCl_3$); >19:1 dr; 90% ee, determined by HPLC analysis [Chiralpak IA, *n*-hexane/*i*-PrOH = 60/40, 1.0 mL min^{-1}, λ = 254 nm, t (minor) = 12.47 min, t (major) = 19.31 min]; **^{1}H NMR** (400 MHz, $CDCl_3$): *δ* (ppm) 8.31 (d, *J* = 2.0 Hz, 1H), 8.29–8.25 (m, 2H), 8.19–8.14 (m, 2H), 8.08 (dd, *J* = 8.4, 2.0 Hz, 1H), 7.70 (t, *J* = 7.4 Hz, 1H), 7.65–7.46 (m, 4H), 6.82 (d, *J* = 8.4 Hz, 1H), 5.55 (s, 1H), 3.86 (d, *J* = 17.6 Hz, 1H), 3.79–3.63 (m, 2H), 3.52 (dd, *J* = 23.2, 3.2 Hz, 1H); **^{13}C NMR** (100 MHz, $CDCl_3$): *δ* (ppm) 189.8, 153.5, 148.3, 148.1, 145.8, 145.3, 145.1, 143.5, 134.8, 132.5, 130.2, 130.0, 129.1, 124.2, 123.3, 119.8, 119.3, 119.1, 62.7, 52.2, 42.5, 35.9; **HRMS** (ESI-TOF) m/z: $[M + Na]^+$ Calcd for $C_{26}H_{17}N_3O_5Na^+$ 474.1066; Found 474.1057.

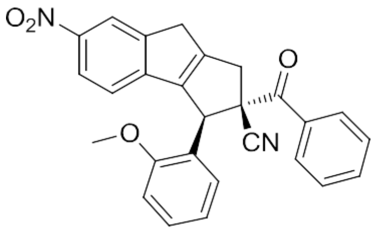

Synthesis of 3f: A flame-dried 10 mL Schlenk tube equipped with a magnetic stirring bar was cooled to room temperature. To this flask were added $Pd_2(dba)_3$ (4.6 mg, 0.0050 mmol, 0.05 equiv), **L4** (12.1 mg, 0.0200 mmol, 0.2 equiv), **A2** (5.0 mg, 0.020 mmol, 0.2 equiv) and 4 Å MS (100.0 mg), and the flask was evacuated and back-filled three times with argon. Then toluene (0.5 mL) was added by syringe. The mixture was stirred at 30 °C for 10 min. To another dry tube equipped with a magnetic stirring bar were added 2-methylene-6-nitro-2,3-dihydro-1*H*-inden-1-ol **1c** (19.1 mg, 0.100 mmol, 1.0 equiv) and

(*E*)-2-benzoyl-3-(2-methoxyphenyl)acrylonitrile **2f** (31.6 mg, 0.120 mmol, 1.2 equiv), evacuated and back-filled three times with argon. Then toluene (0.5 mL) was added by syringe. The mixture was stirred at rt for 5 min. Then the latter solution was added to the first Schlenk tube by syringe. The mixture was stirred at 30 °C for 2 h. After completion, Et_3N (2.8 μL, 0.020 mmol, 0.2 equiv) was added to the reaction mixture and stirred at rt for 2 h. After completion, the solvent was evaporated in vacuo. The residue was purified by flash chromatography on silica gel (EtOAc/petroleum ether = 1/8) to give product **3f**: 43.2 mg, 99% yield, as a yellow solid; mp: 120–121 °C; $[\alpha]_D^{25}$ = +88.5 (*c* = 1.6 in $CHCl_3$); >19:1 dr; 95% ee, determined by HPLC analysis [Chiralpak IA, *n*-hexane/*i*-PrOH = 80/20, 1.0 mL min^{-1}, λ = 254 nm, t (major) = 12.24 min, t (minor) = 16.42 min]; **^{1}H NMR** (400 MHz, $CDCl_3$): *δ* (ppm) 8.28 (s, 1H), 8.11–8.03 (m, 3H), 7.63 (t, *J* = 7.4 Hz, 1H), 7.55–7.45 (m, 2H), 7.42–7.32 (m, 1H), 7.05 (s, 1H), 6.98–6.91 (m, 3H), 5.60 (s, 1H), 3.95–3.56 (m, 6H), 3.51 (dd, *J* = 23.2, 3.2 Hz, 1H); **^{13}C NMR** (100 MHz, $CDCl_3$): *δ* (ppm) 191.9, 157.4, 153.9, 148.5, 146.3, 145.4, 133.9, 133.4, 130.1, 129.6, 128.7, 124.5, 123.2, 120.9, 120.1, 119.5, 110.8, 61.9, 55.1, 48.3, 42.5, 35.8; **HRMS** (ESI-TOF) m/z: $[M + Na]^+$ Calcd for $C_{27}H_{20}N_2O_4Na^+$ 459.1321; Found 459.1327.

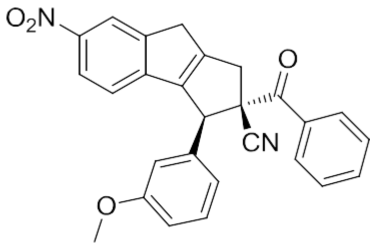

Synthesis of 3g: A flame-dried 10 mL Schlenk tube equipped with a magnetic stirring bar was cooled to room temperature. To this flask were added $Pd_2(dba)_3$ (4.6 mg, 0.0050 mmol, 0.05 equiv), **L4** (12.1 mg, 0.0200 mmol, 0.2 equiv), (*S*)-**A1** (7.0 mg, 0.020 mmol, 0.2 equiv) and 4 Å MS (100.0 mg), and the flask was evacuated and back-filled three times with argon. Then toluene (0.5 mL) was added by syringe. The mixture was stirred at rt for 10 min. To another dry tube equipped with a magnetic stirring bar were added 2-methylene-6-nitro-2,3-dihydro-1*H*-inden-1-ol **1c** (19.1 mg, 0.100 mmol, 1.0 equiv) and (*E*)-2-benzoyl-3-(3-methoxyphenyl)acrylonitrile **2g** (31.6 mg, 0.120 mmol, 1.2 equiv), evacuated and back-filled three times with argon. Then toluene (0.5 mL) was added by syringe. The mixture was stirred at rt for 5 min. Then the latter solution was added to the first Schlenk tube by syringe. The mixture was stirred at 30 °C for 12 h. After completion, Et_3N (2.8 μL, 0.020 mmol, 0.2 equiv) was added to the reaction mixture and stirred at rt for 2 h. After completion, the solvent was evaporated in vacuo. The residue was purified by flash chromatography on silica gel (EtOAc/petroleum ether = 1/7) to give product **3g**: 32.7 mg, 75% yield, as a yellow solid; mp: 126–127 °C; $[\alpha]_D^{25}$ = +84.4 (*c* = 1.6 in $CHCl_3$); >19:1 dr; 93% ee, determined by HPLC analysis [Chiralpak IA, *n*-hexane/*i*-PrOH = 80/20, 1.0 mL min-1, λ = 254 nm, t (minor) = 14.03 min, t (major) = 15.44 min]; **^{1}H NMR** (400 MHz, $CDCl_3$): *δ* (ppm) 8.35 (d, *J* = 2.0 Hz, 1H), 8.18–8.14 (m, 2H), 8.04 (dd, *J* = 8.0, 2.8 Hz, 1H), 7.66 (t, *J* = 7.4 Hz, 1H), 7.56–7.50 (m, 2H), 7.35–7.31 (m, 1H),

6.92 (dd, J = 8.3, 2.6 Hz, 1H), 6.90–6.82 (m, 3H), 5.12 (s, 1H), 3.85–3.75 (m, 4H), 3.86–3.77 (m, 4H), 3.72–3.58 (m, 2H), 3.50 (dd, J = 23.2, 3.2 Hz, 1H); **^{13}C NMR** (100 MHz, $CDCl_3$): δ (ppm) 190.5, 160.0, 153.4, 148.3, 146.1, 145.8, 145.4, 137.5, 134.4, 132.7, 130.1, 130.0, 128.9, 123.2, 121.3, 119.6, 119.5, 119.3, 115.2, 113.7, 62.3, 55.3, 53.8, 41.8, 35.7; **HRMS** (ESI-TOF) m/z: $[M + Na]^+$ Calcd for $C_{27}H_{20}N_2O_4Na^+$ 459.1321; Found 459.1312.

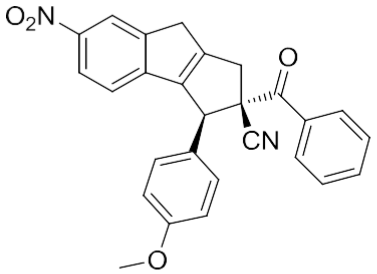

Synthesis of 3h: A flame-dried 10 mL Schlenk tube equipped with a magnetic stirring bar was cooled to room temperature. To this flask were added $Pd_2(dba)_3$ (4.6 mg, 0.0050 mmol, 0.05 equiv), **L4** (12.1 mg, 0.0200 mmol, 0.2 equiv), **A2** (5.0 mg, 0.020 mmol, 0.2 equiv) and 4 Å MS (100.0 mg), and the flask was evacuated and back-filled three times with argon. Then toluene (0.5 mL) was added by syringe. The mixture was stirred at 30 °C for 10 min. To another dry tube equipped with a magnetic stirring bar were added 2-methylene-6-nitro-2,3-dihydro-1*H*-inden-1-ol **1c** (19.1 mg, 0.100 mmol, 1.0 equiv) and (*E*)-2-benzoyl-3-(4-methoxyphenyl)acrylonitrile **2h** (31.6 mg, 0.120 mmol, 1.2 equiv), evacuated and back-filled three times with argon. Then toluene (0.5 mL) was added by syringe. The mixture was stirred at rt for 5 min. Then the latter solution was added to the first Schlenk tube by syringe. The mixture was stirred at 30 °C for 2 h. After completion, Et_3N (2.8 μL, 0.020 mmol, 0.2 equiv) was added to the reaction mixture and stirred at rt for 2 h. After completion, the solvent was evaporated in vacuo. The residue was purified by flash chromatography on silica gel (EtOAc/petroleum ether = 1/7) to give product **3h**: 43.2 mg, 99% yield, as a yellow solid; mp: 120–121 °C; $[\alpha]_D^{25}$ = +83.4 (c = 1.5 in $CHCl_3$); >19:1 dr; 88% ee, determined by HPLC analysis [Chiralpak IB, *n*-hexane/*i*-PrOH = 80/20, 1.0 mL min^{-1}, λ = 254 nm, t (major) = 14.59 min, t (minor) = 18.06 min]; **^{1}H NMR** (400 MHz, $CDCl_3$): δ (ppm) 8.26 (d, J = 2.0 Hz, 1H), 8.16–8.12 (m, 2H), 8.03 (dd, J = 8.4, 2.0 Hz, 1H), 7.66 (t, J = 7.4 Hz, 1H), 7.57–7.49 (m, 2H), 7.24–7.20 (m, 2H), 6.96–6.90 (m, 2H), 6.82 (d, J = 8.4 Hz, 1H), 5.11 (s, 1H), 3.86–3.77 (m, 4H), 3.71–3.58 (m, 2H), 3.50 (dd, J = 23.2, 3.2 Hz, 1H); **^{13}C NMR** (100 MHz, $CDCl_3$): δ (ppm) 190.7, 159.9, 153.1, 148.4, 146.4, 145.9, 145.5, 134.4, 132.8, 130.3, 130.0, 128.9, 127.8, 123.2, 119.8, 119.5, 119.2, 114.5, 62.6, 55.3, 53.4, 41.7, 35.7; **HRMS** (ESI-TOF) m/z: $[M + H]^+$ Calcd for $C_{27}H_{21}N_2O_4^+$ 437.1501; Found 437.1494.

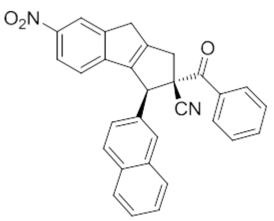

Synthesis of 3i: A flame-dried 10 mL Schlenk tube equipped with a magnetic stirring bar was cooled to room temperature. To this flask were added $Pd_2(dba)_3$ (4.6 mg, 0.0050 mmol, 0.05 equiv), **L4** (12.1 mg, 0.0200 mmol, 0.2 equiv), **A2** (5.0 mg, 0.020 mmol, 0.2 equiv) and 4 Å MS (100.0

mg), and the flask was evacuated and back-filled three times with argon. Then toluene (0.5 mL) was added by syringe. The mixture was stirred at 30 °C for 10 min. To another dry tube equipped with a magnetic stirring bar were added 2-methylene-6-nitro-2,3-dihydro-1*H*-inden-1-ol **1c** (19.1 mg, 0.100 mmol, 1.0 equiv) and (*E*)-2-benzoyl-3-(naphthalen-2-yl)acrylonitrile **2i** (34.0 mg, 0.120 mmol, 1.2 equiv), evacuated and back-filled three times with argon. Then toluene (0.5 mL) was added by syringe. The mixture was stirred at rt for 5 min. Then the latter solution was added to the first Schlenk tube by syringe. The mixture was stirred at 30 °C for 2 h. After completion, Et_3N (2.8 μL, 0.020 mmol, 0.2 equiv) was added to the reaction mixture and stirred at rt for 2 h. After completion, the solvent was evaporated in vacuo. The residue was purified by flash chromatography on silica gel (EtOAc/petroleum ether = 1/8) to give product **3i**: 45.1 mg, 99% yield, as a yellow solid; mp: 123–124 °C; $[\alpha]_D^{25}$ = +73.6 (*c* = 1.8 in $CHCl_3$); >19:1 dr; 91% ee, determined by HPLC analysis [Chiralpak IA, *n*-hexane/*i*-PrOH = 80/20, 1.0 mL min^{-1}, λ = 254 nm, t (major) = 16.03 min, t (minor) = 19.28 min]; **^{1}H NMR** (400 MHz, $CDCl_3$): *δ* (ppm) 8.28 (d, *J* = 2.0 Hz, 1H), 8.23–8.13 (m, 2H), 7.97 (dd, *J* = 8.4, 2.0 Hz, 1H), 7.91–7.78 (m, 4H), 7.66 (t, *J* = 7.6 Hz, 1H), 7.56–7.47 (m, 4H), 7.38 (dd, *J* = 8.4, 2.0 Hz, 1H), 6.78 (d, *J* = 8.4 Hz, 1H), 5.35 (s, 1H), 3.87 (d, *J* = 17.6 Hz, 1H), 3.77 (d, *J* = 17.6 Hz, 1H), 3.69 (d, *J* = 23.2 Hz, 1H), 3.54 (dd, *J* = 23.2, 3.2 Hz, 1H); **^{13}C NMR** (100 MHz, $CDCl_3$): *δ* (ppm) 191.5, 152.6, 148.4, 147.3, 146.0, 145.6, 134.4, 134.2, 132.8, 132.3, 132.2, 130.0, 129.5, 129.3, 128.9, 127.02, 127.00, 126.0, 125.4, 123.2, 123.1, 119.7, 119.58, 119.51, 61.9, 48.1, 43.5, 35.8; **HRMS** (ESI-TOF) m/z: $[M + H]^+$ Calcd for $C_{30}H_{21}N_2O_3^+$ 457.1552; Found 457.1559.

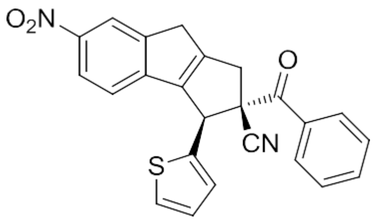

Synthesis of 3j: A flame-dried 10 mL Schlenk tube equipped with a magnetic stirring bar was cooled to room temperature. To this flask were added $Pd_2(dba)_3$ (4.6 mg, 0.0050 mmol, 0.05 equiv), **L4** (12.1 mg, 0.0200 mmol, 0.2 equiv), **A2** (5.0 mg, 0.020 mmol, 0.2 equiv) and 4 Å MS (100.0 mg), and the flask was evacuated and back-filled three times with argon. Then toluene (0.5 mL) was added by syringe. The mixture was stirred at 30 °C for 10 min. To another dry tube equipped with a magnetic stirring bar were added 2-methylene-6-nitro-2,3-dihydro-1*H*-inden-1-ol **1c** (19.1 mg, 0.100 mmol, 1.0 equiv) and (*E*)-2-benzoyl-3-(thiophen-2-yl)acrylonitrile **2j** (28.7 mg, 0.123 mmol, 1.2 equiv), evacuated and back-filled three times with argon. Then toluene (0.5 mL) was added by syringe. The mixture was stirred at rt for 5 min. Then the latter solution was added to the first Schlenk tube by syringe. The mixture was stirred at 30 °C for 2 h. After completion, Et_3N (2.8 μL, 0.020 mmol, 0.2 equiv) was added to the reaction mixture and stirred at rt for 2 h. After completion, the solvent was evaporated in vacuo. The residue was purified by flash chromatography on silica gel (EtOAc/petroleum ether =

1/8) to give product **3j**: 28.8 mg, 70% yield, as a yellow solid; mp: 117–118 °C; $[\alpha]_D^{25}$ = +90.6 (*c* = 1.5 in $CHCl_3$); >19:1 dr; 88% ee, determined by HPLC analysis [Chiralpak IA, *n*-hexane/*i*-PrOH = 80/20, 1.0 mL min^{-1}, λ = 254 nm, t (minor) = 14.09 min, t (major) = 17.10 min]; **^{1}H NMR** (400 MHz, $CDCl_3$): *δ* (ppm) 8.27 (d, *J* = 2.0 Hz, 1H), 8.24–8.20 (m, 2H), 8.09 (dd, *J* = 8.4, 2.0 Hz, 1H), 7.68 (t, *J* = 7.4 Hz, 1H), 7.62–7.53 (m, 2H), 7.34 (dd, *J* = 5.2, 1.6 Hz, 1H), 7.11–7.07 (m, 2H), 7.01 (d, *J* = 8.4 Hz, 1H), 5.49 (s, 1H), 3.77 (d, *J* = 17.6 Hz, 1H), 3.70 (dd, *J* = 17.6, 2.0 Hz, 1H), 3.64 (d, *J* = 22.8 Hz, 1H), 3.51 (dd, *J* = 22.8, 3.2 Hz, 1H); **^{13}C NMR** (100 MHz, $CDCl_3$): *δ* (ppm) 190.1, 153.3, 148.2, 146.0, 145.6, 145.5, 138.7, 134.5, 132.5, 130.1, 129.0, 127.7, 127.6, 126.5, 123.2, 119.6, 119.3, 119.1, 62.5, 48.7, 40.9, 35.7; **HRMS** (ESI-TOF) m/z: $[M + Na]^+$ Calcd for $C_{24}H_{16}N_2O_3SNa^+$ 435.0779; Found 435.0773.

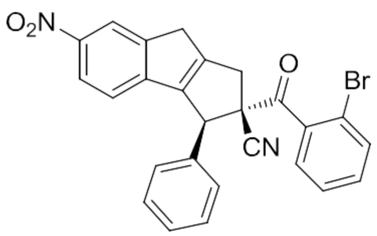

Synthesis of 3k: A flame-dried 10 mL Schlenk tube equipped with a magnetic stirring bar was cooled to room temperature. To this flask were added $Pd_2(dba)_3$ (4.6 mg, 0.0050 mmol, 0.05 equiv), **L4** (12.1 mg, 0.0200 mmol, 0.2 equiv), (*S*)-**A1** (7.0 mg, 0.020 mmol, 0.2 equiv) and 4 Å MS (100.0 mg), and the flask was evacuated and back-filled three times with argon. Then toluene (0.5 mL) was added by syringe. The mixture was stirred at 30 °C for 10 min. To another dry tube equipped with a magnetic stirring bar were added 2-methylene-6-nitro-2,3-dihydro-1*H*-inden-1-ol **1c** (19.1 mg, 0.100 mmol, 1.0 equiv) and (*E*)-2-(2-bromobenzoyl)-3-phenylacrylonitrile **2k** (37.3 mg, 0.120 mmol, 1.2 equiv), evacuated and back-filled three times with argon. Then toluene (0.5 mL) was added by syringe. The mixture was stirred at rt for 5 min. Then the latter solution was added to the first Schlenk tube by syringe. The mixture was stirred at 30 °C for 14 h. After completion, Et_3N (2.8 μL, 0.020 mmol, 0.2 equiv) was added to the reaction mixture and stirred at rt for 2 h. After completion, the solvent was evaporated in vacuo. The residue was purified by flash chromatography on silica gel (EtOAc/petroleum ether = 1/7) to give product **3k**: 35.2 mg, 73% yield, as a yellow solid; mp: 114–115 °C; $[\alpha]_D^{25}$ = +90.4 (*c* = 1.7 in $CHCl_3$); >19:1 dr; 91% ee, determined by HPLC analysis [Chiralpak IA, *n*-hexane/*i*-PrOH = 80/20, 1.0 mL min^{-1}, λ = 254 nm, t (minor) = 15.36 min, t (major) = 16.34 min]; **^{1}H NMR** (400 MHz, $CDCl_3$): *δ* (ppm) 8.30 (d, *J* = 2.4 Hz, 1H), 8.06 (dd, *J* = 8.4, 2.0 Hz, 1H), 7.71–7.61 (m, 1H), 7.48–7.44 (m, 1H), 7.42–7.34 (m, 5H), 7.23–7.18 (m, 2H), 6.83 (d, *J* = 8.4 Hz, 1H), 5.22 (s, 1H), 3.74 (dd, *J* = 17.2, 1.6 Hz, 1H), 3.71–3.55 (m, 2H), 3.52 (dd, *J* = 23.2, 3.2 Hz, 1H); **^{13}C NMR** (100 MHz, $CDCl_3$): *δ* (ppm) 196.4, 153.5, 148.4, 146.0, 145.8, 145.6, 138.9, 135.3, 133.7, 132.3, 129.0, 128.98, 128.95, 127.6, 127.4, 123.2, 119.6, 119.4, 119.1, 118.7, 65.9, 54.5, 42.3, 35.8; **HRMS** (ESI-TOF) m/z: $[M + Na]^+$ Calcd for $C_{26}H_{17}BrN_2O_3Na^+$ 507.0320 (^{79}Br) and 509.0300 (^{81}Br); Found 507.0313 (^{79}Br) and 509.0291 (^{81}Br).

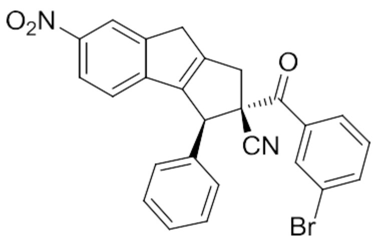

Synthesis of 3l: A flame-dried 10 mL Schlenk tube equipped with a magnetic stirring bar was cooled to room temperature. To this flask were added $Pd_2(dba)_3$ (4.6 mg, 0.0050 mmol, 0.05 equiv), **L4** (12.1 mg, 0.0200 mmol, 0.2 equiv), (*S*)-**A1** (7.0 mg, 0.020 mmol, 0.2 equiv), and 4 Å MS (100.0 mg), and the flask was evacuated and back-filled three times with argon. Then toluene (0.5 mL) was added by syringe. The mixture was stirred at 30 °C for 10 min. To another dry tube equipped with a magnetic stirring bar were added 2-methylene-6-nitro-2,3-dihydro-1*H*-inden-1-ol **1c** (19.1 mg, 0.100 mmol, 1.0 equiv) and (*E*)-2-(3-bromobenzoyl)-3-phenylacrylonitrile **2l** (37.3 mg, 0.120 mmol, 1.2 equiv), evacuated and back-filled three times with argon. Then toluene (0.5 mL) was added by syringe. The mixture was stirred at rt for 5 min. Then the latter solution was added to the first Schlenk tube by syringe. The mixture was stirred at 30 °C for 14 h. After completion, Et_3N (2.8 μL, 0.020 mmol, 0.2 equiv) was added to the reaction mixture and stirred at rt for 2 h. After completion, the solvent was evaporated in vacuo. The residue was purified by flash chromatography on silica gel (EtOAc/petroleum ether = 1/7) to give product **3l**: 36.3 mg, 75% yield, as a yellow solid; mp: 128–129 °C; $[\alpha]_D^{25}$ = +88.5 (*c* = 1.7 in $CHCl_3$); >19:1 dr; 82% ee, determined by HPLC analysis [Chiralpak IA, *n*-hexane/*i*-PrOH = 90/10, 1.0 mL min^{-1}, λ = 254 nm, t (major) = 24.16 min, t (minor) = 27.15 min]; **^{1}H NMR** (400 MHz, $CDCl_3$): δ (ppm) 8.27 (d, *J* = 2.0 Hz, 1H), 8.24–8.23 (m, 1H), 8.07 (d, *J* = 8.0 Hz, 1H), 8.03 (dd, *J* = 8.4, 2.0 Hz, 1H), 7.80–7.76 (m, 1H), 7.45–7.36 (m, 4H), 7.32–7.27 (m, 2H), 6.80 (d, *J* = 8.4 Hz, 1H), 5.11 (s, 1H), 3.83 (d, *J* = 17.2 Hz, 1H), 3.74–3.60 (m, 2H), 3.52 (dd, *J* = 23.2, 3.2 Hz, 1H); **^{13}C NMR** (100 MHz, $CDCl_3$): δ (ppm) 189.5, 153.2, 148.3, 146.0, 145.7, 145.5, 137.3, 135.6, 134.5, 133.0, 130.3, 129.2, 129.1, 128.4, 123.3, 123.2, 119.6, 119.3, 119.26, 62.5, 54.0, 41.6, 35.8; **HRMS** (ESI-TOF) m/z: $[M + Na]^+$ Calcd for $C_{26}H_{17}BrN_2O_3Na^+$ 507.0320 (^{79}Br) and 509.0300 (^{81}Br); Found 507.0314 (^{79}Br) and 509.0296 (^{81}Br).

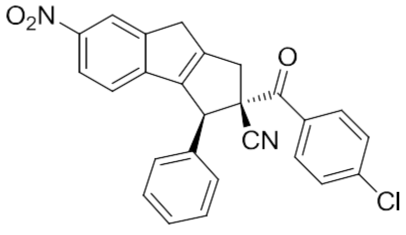

Synthesis of 3m: A flame-dried 10 mL Schlenk tube equipped with a magnetic stirring bar was cooled to room temperature. To this flask were added $Pd_2(dba)_3$ (4.6 mg, 0.0050 mmol, 0.05 equiv), **L4** (12.1 mg, 0.0200 mmol, 0.2 equiv), **A2** (5.0 mg, 0.020 mmol, 0.2 equiv) and 4 Å MS (100.0 mg), and the flask was evacuated and back-filled three times with argon. Then toluene (0.5 mL) was added by syringe. The mixture was stirred at 30 °C for 10 min. To another dry tube equipped with a magnetic stirring bar were added 2-methylene-6-nitro-2,3-dihydro-1*H*-inden-1-ol **1c** (19.1 mg, 0.100 mmol, 1.0 equiv) and (*E*)-2-(4-chlorobenzoyl)-3-phenylacrylonitrile **2m** (32.0 mg, 0.120 mmol, 1.2 equiv), evacuated

and back-filled three times with argon. Then toluene (0.5 mL) was added by syringe. The mixture was stirred at rt for 5 min. Then the latter solution was added to the first Schlenk tube by syringe. The mixture was stirred at 30 °C for 0.5 h. After completion, Et_3N (2.8 μL, 0.020 mmol, 0.2 equiv) was added to the reaction mixture and stirred at rt for 2 h. After completion, the solvent was evaporated in vacuo. The residue was purified by flash chromatography on silica gel (EtOAc/petroleum ether = 1/8) to give product **3m**: 43.9 mg, 99% yield, as a yellow solid; mp: 113–114 °C; $[\alpha]_D^{25}$ = +72.6 (*c* = 1.4 in $CHCl_3$); >19:1 dr; 93% ee, determined by HPLC analysis [Chiralpak IA, *n*-hexane/*i*-PrOH = 80/20, 1.0 mL min^{-1}, λ = 254 nm, t (minor) = 12.40 min, t (major) = 16.17 min]; **^{1}H NMR** (400 MHz, $CDCl_3$): *δ* (ppm) 8.27 (d, *J* = 2.0 Hz, 1H), 8.10–8.06 (m, 2H), 8.03 (dd, *J* = 8.4, 2.0 Hz, 1H), 7.52–7.48 (m, 2H), 7.44–7.39 (m, 3H), 7.31–7.27 (m, 2H), 6.80 (d, *J* = 8.4 Hz, 1H), 5.12 (s, 1H), 3.81 (d, *J* = 17.8 Hz, 1H), 3.73–3.58 (m, 2H), 3.51 (dd, *J* = 23.6, 3.2 Hz, 1H); **^{13}C NMR** (100 MHz, $CDCl_3$): *δ* (ppm) 189.5, 153.2, 148.3, 146.1, 145.7, 145.5, 141.2, 135.7, 131.4, 131.0, 129.3, 129.2, 129.06, 129.03, 123.2, 119.56, 119.52, 119.3, 62.4, 54.0, 41.7, 35.7; **HRMS** (ESI-TOF) m/z: $[M + Na]^+$ Calcd for $C_{26}H_{17}ClN_2O_3Na^+$ 463.0825 (^{35}Cl) and 465.0976 (^{37}Cl); Found 463.0821 (^{35}Cl) and 465.0803 (^{37}Cl).

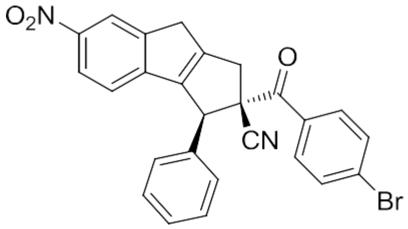

Synthesis of 3n: A flame-dried 10 mL Schlenk tube equipped with a magnetic stirring bar was cooled to room temperature. To this flask were added $Pd_2(dba)_3$ (4.6 mg, 0.0050 mmol, 0.05 equiv), **L4** (12.1 mg, 0.0200 mmol, 0.2 equiv), **A2** (5.0 mg, 0.020 mmol, 0.2 equiv) and 4 Å MS (100.0 mg), and the flask was evacuated and back-filled three times with argon. Then toluene (0.5 mL) was added by syringe. The mixture was stirred at 30 °C for 10 min. To another dry tube equipped with a magnetic stirring bar were added 2-methylene-6-nitro-2,3-dihydro-1*H*-inden-1-ol **1c** (19.1 mg, 0.100 mmol, 1.0 equiv) and (*E*)-2-(4-bromobenzoyl)-3-phenylacrylonitrile **2n** (37.3 mg, 0.120 mmol, 1.2 equiv), evacuated and back-filled three times with argon. Then toluene (0.5 mL) was added by syringe. The mixture was stirred at rt for 5 min. Then the latter solution was added to the first Schlenk tube by syringe. The mixture was stirred at 30 °C for 24 h. After completion, Et_3N (2.8 μL, 0.020 mmol, 0.2 equiv) was added to the reaction mixture and stirred at rt for 2 h. After completion, the solvent was evaporated in vacuo. The residue was purified by flash chromatography on silica gel (EtOAc/petroleum ether = 1/8) to give product **3n**: 36.8 mg, 76% yield, as a yellow solid; mp: 116–117 °C; $[\alpha]_D^{25}$ = +94.4 (*c* = 1.5 in $CHCl_3$); >19:1 dr; 94% ee, determined by HPLC analysis [Chiralpak IA, *n*-hexane/*i*-PrOH = 80/20, 1.0 mL min^{-1}, λ = 254 nm, t (minor) = 13.50 min, t (major) = 17.42 min]; **^{1}H NMR** (400 MHz, $CDCl_3$): *δ* (ppm) 8.27 (d, *J* = 2.4 Hz, 1H), 8.10–7.92 (m, 3H), 7.70–7.65 (m, 2H), 7.46–7.38 (m, 3H), 7.32–7.22 (m, 2H), 6.81 (d, *J* = 8.4 Hz, 1H), 5.12 (s, 1H),

3.82 (d, J = 18.0 Hz, 1H), 3.73–3.60 (m, 2H), 3.52 (dd, J = 23.2, 3.2 Hz, 1H); **^{13}C NMR** (100 MHz, $CDCl_3$): δ (ppm) 189.7, 153.2, 148.3, 146.1, 145.7, 145.5, 135.7, 132.3, 131.4, 130.0, 129.2, 129.0, 123.2, 119.6, 119.5, 119.2, 62.4, 54.0, 41.7, 35.7; **HRMS** (ESI-TOF) m/z: $[M + Na]^+$ Calcd for $C_{26}H_{17}BrN_2O_3Na^+$ 507.0320 (^{79}Br) and 509.0300 (^{81}Br); Found 507.0316 (^{79}Br) and 509.0301 (^{81}Br).

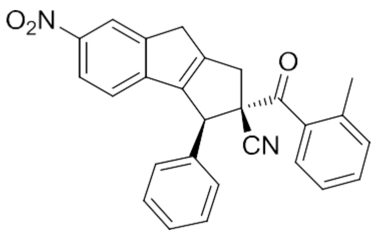

Synthesis of 3o: A flame-dried 10 mL Schlenk tube equipped with a magnetic stirring bar was cooled to room temperature. To this flask were added $Pd_2(dba)_3$ (4.6 mg, 0.0050 mmol, 0.05 equiv), **L4** (12.1 mg, 0.0200 mmol, 0.2 equiv), (*S*)-**A1** (7.0 mg, 0.020 mmol, 0.2 equiv), and 4 Å MS (100.0 mg), and the flask was evacuated and back-filled three times with argon. Then toluene (0.5 mL) was added by syringe. The mixture was stirred at 30 °C for 10 min. To another dry tube equipped with a magnetic stirring bar were added 2-methylene-6-nitro-2,3-dihydro-1*H*-inden-1-ol **1c** (19.1 mg, 0.100 mmol, 1.2 equiv) and (*E*)-2-(2-methylbenzoyl)-3-phenylacrylonitrile **2o** (29.6 mg, 0.120 mmol, 1.2 equiv), evacuated and back-filled three times with argon. Then toluene (0.5 mL) was added by syringe. The mixture was stirred at rt for 5 min. Then the latter solution was added to the first Schlenk tube by syringe. The mixture was stirred at 30 °C for 14 h. After completion, Et_3N (2.8 μL, 0.020 mmol, 0.2 equiv) was added to the reaction mixture and stirred at rt for 2 h. After completion, the solvent was evaporated in vacuo. The residue was purified by flash chromatography on silica gel (EtOAc/petroleum ether = 1/7) to give product **3o**: 37.8 mg, 90% yield, as a yellow solid; mp: 124–125 °C; $[\alpha]_D^{25}$ = +80.9 (c = 1.7 in $CHCl_3$); >19:1 dr; 89% ee, determined by HPLC analysis [Chiralpak IA, *n*-hexane/*i*-PrOH = 80/20, 1.0 mL min^{-1}, λ = 254 nm, t (minor) = 11.52 min, t (major) = 12.75 min]; **^{1}H NMR** (400 MHz, $CDCl_3$): δ (ppm) 8.21 (d, J = 2.0 Hz, 1H), 7.97 (dd, J = 8.4, 2.4 Hz, 1H), 7.67 (d, J = 7.6 Hz, 1H), 7.36 (td, J = 7.6, 1.2 Hz, 1H), 7.32–7.29 (m, 3H), 7.26–7.19 (m, 2H), 7.19–7.15 (m, 2H), 6.74 (d, J = 8.4 Hz, 1H), 5.13 (s, 1H), 3.64 (d, J = 17.2 Hz, 1H), 3.61–3.51 (m, 2H), 3.43 (dd, J = 23.2, 3.6 Hz, 1H), 2.34 (s, 3H); **^{13}C NMR** (100 MHz, $CDCl_3$): δ (ppm) 195.8, 153.4, 148.4, 146.2, 145.9, 145.5, 138.7, 135.7, 134.8, 132.2, 132.1, 129.04, 129.02, 128.9, 127.9, 125.6 123.2, 119.58, 119.56, 119.4, 65.0, 54.3, 42.5, 35.8, 21.1; **HRMS** (ESI-TOF) m/z: $[M + H]^+$ Calcd for $C_{27}H_{21}N_2O_3^+$ 421.1552; Found 421.1553.

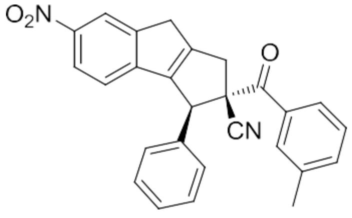

Synthesis of 3p: A flame-dried 10 mL Schlenk tube equipped with a magnetic stirring bar was cooled to room temperature. To this flask were added $Pd_2(dba)_3$ (4.6 mg, 0.0050 mmol, 0.05 equiv), **L4** (12.1 mg, 0.0200 mmol, 0.2 equiv), (*S*)-**A1** (7.0 mg, 0.020 mmol, 0.2 equiv)

and 4 Å MS (100.0 mg), and the flask was evacuated and back-filled three times with argon. Then toluene (0.5 mL) was added by syringe. The mixture was stirred at 30 °C for 10 min. To another dry tube equipped with a magnetic stirring bar were added 2-methylene-6-nitro-2,3-dihydro-1*H*-inden-1-ol **1c** (19.1 mg, 0.100 mmol, 1.2 equiv) and (*E*)-2-(3-methylbenzoyl)-3-phenylacrylonitrile **2p** (29.6 mg, 0.120 mmol, 1.2 equiv), evacuated and back-filled three times with argon. Then toluene (0.5 mL) was added by syringe. The mixture was stirred at rt for 5 min. Then the latter solution was added to the first Schlenk tube by syringe. The mixture was stirred at 30 °C for 12 h. After completion, Et_3N (2.8 μL, 0.020 mmol, 0.2 equiv) was added to the reaction mixture and stirred at rt for 2 h. After completion, the solvent was evaporated in vacuo. The residue was purified by flash chromatography on silica gel (EtOAc/petroleum ether = 1/7) to give product **3p**: 30.1 mg, 72% yield, as a yellow solid; mp: 121–122 °C; $[\alpha]_D^{25}$ = +86.4 (*c* = 1.5 in $CHCl_3$); >19:1 dr; 91% ee, determined by HPLC analysis [Chiralpak IA, *n*-hexane/*i*-PrOH = 80/20, 1.0 mL min^{-1}, λ = 254 nm, t (minor) = 11.16 min, t (major) = 12.86 min]; **^{1}H NMR** (400 MHz, $CDCl_3$): *δ* (ppm) 8.26 (d, *J* = 2.0 Hz, 1H), 8.06 (dd, *J* = 8.4, 2.0 Hz, 1H), 7.95 (d, *J* = 7.6 Hz, 1H), 7.91 (s, 1H), 7.49–7.44 (m, 1H), 7.44–7.38 (m, 4H), 7.32–7.28 (m, 2H), 6.80 (d, *J* = 8.4 Hz, 1H), 5.14 (s, 1H), 3.85 (d, *J* = 17.2 Hz, 1H), 3.74–3.56 (m, 2H), 3.51 (dd, *J* = 23.2, 3.2 Hz, 1H), 2.42 (s, 3H); **^{13}C NMR** (100 MHz, $CDCl_3$): *δ* (ppm) 190.8, 153.5, 148.4, 146.2, 145.9, 145.5, 138.8, 136.0, 135.2, 132.8, 130.6, 129.1, 129.0, 128.8, 128.7, 127.2, 123.2, 119.7, 119.5, 119.2, 62.5, 54.0, 41.8, 35.8, 21.4; **HRMS** (ESI-TOF) m/z: $[M + H]^+$ Calcd for $C_{27}H_{21}N_2O_3^+$ 421.1552; Found 421.1549.

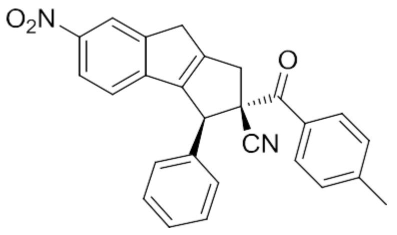

Synthesis of 3q: A flame-dried 10 mL Schlenk tube equipped with a magnetic stirring bar was cooled to room temperature. To this flask were added $Pd_2(dba)_3$ (4.6 mg, 0.0050 mmol, 0.05 equiv), **L4** (12.1 mg, 0.0200 mmol, 0.2 equiv), **A2** (5.0 mg, 0.020 mmol, 0.2 equiv) and 4 Å MS (100.0 mg), and the flask was evacuated and back-filled three times with argon. Then toluene (0.5 mL) was added by syringe. The mixture was stirred at rt for 10 min. To another dry tube equipped with a magnetic stirring bar were added 2-methylene-6-nitro-2,3-dihydro-1*H*-inden-1-ol **1c** (19.1 mg, 0.100 mmol, 1.0 equiv) and (*E*)-2-(4-methylbenzoyl)-3-phenylacrylonitrile **2q** (29.6 mg, 0.120 mmol, 1.2 equiv), evacuated and back-filled three times with argon. Then toluene (0.5 mL) was added by syringe. The mixture was stirred at rt for 5 min. Then the latter solution was added to the first Schlenk tube by syringe. The mixture was stirred at 30 °C for 2 h. After completion, Et_3N (2.8 μL, 0.020 mmol, 0.2 equiv) was added to the reaction mixture and stirred at rt for 1 h. After completion, the solvent was evaporated in vacuo. The residue was purified by flash chromatography on silica gel (EtOAc/petroleum ether = 1/8) to give product **3q**: 41.6 mg, 99% yield, as a yellow solid; mp: 113–114 °C; $[\alpha]_D^{25}$ = +79.3 (*c* =

1.5 in $CHCl_3$); >19:1 dr; 92% ee, determined by HPLC analysis [Chiralpak IA, *n*-hexane/*i*-PrOH = 80/20, 1.0 mL min^{-1}, λ = 254 nm, t (minor) = 13.35 min, t (major) = 16.62 min]; **^{1}H NMR** (400 MHz, $CDCl_3$): *δ* (ppm) 8.26 (d, *J* = 2.0 Hz, 1H), 8.07–8.01 (m, 3H), 7.46–7.36 (m, 3H), 7.37–7.28 (m, 4H), 6.81 (d, *J* = 8.4 Hz, 1H), 5.16 (s, 1H), 3.82 (d, *J* = 18.0 Hz, 1H), 3.75–3.58 (m, 2H), 3.51 (dd, *J* = 23.2, 3.2 Hz, 1H), 2.45 (s, 3H); **^{13}C NMR** (100 MHz, $CDCl_3$): *δ* (ppm) 190.1, 153.4, 148.4, 146.3, 145.9, 145.7, 145.5, 136.1, 130.2, 130.1, 129.6, 129.1, 128.9, 123.2, 119.8, 119.5, 119.2, 62.3, 53.9, 41.8, 35.7, 21.8; **HRMS** (ESI-TOF) m/z: $[M + Na]^+$ Calcd for $C_{27}H_{20}N_2O_3Na^+$ 443.1372; Found 443.1365.

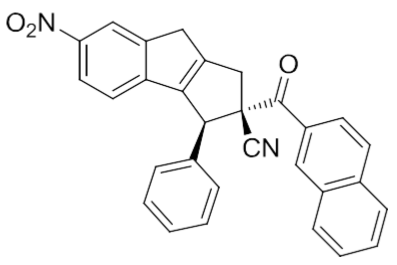

Synthesis of 3r: A flame-dried 10 mL Schlenk tube equipped with a magnetic stirring bar was cooled to room temperature. To this flask were added $Pd_2(dba)_3$ (4.6 mg, 0.0050 mmol, 0.05 equiv), **L4** (12.1 mg, 0.0200 mmol, 0.2 equiv), **A2** (5.0 mg, 0.020 mmol, 0.2 equiv) and 4 Å MS (100.0 mg), and the flask was evacuated and back-filled three times with argon. Then toluene (0.5 mL) was added by syringe. The mixture was stirred at 30 °C for 10 min. To another dry tube equipped with a magnetic stirring bar were added 2-methylene-6-nitro-2,3-dihydro-1*H*-inden-1-ol **1c** (19.1 mg, 0.100 mmol, 1.0 equiv) and (*E*)-2-(2-naphthoyl)-3-phenylacrylonitrile **2r** (34.0 mg, 0.120 mmol, 1.2 equiv), evacuated and back-filled three times with argon. Then toluene (0.5 mL) was added by syringe. The mixture was stirred at rt for 5 min. Then the latter solution was added to the first Schlenk tube by syringe. The mixture was stirred at 30 °C for 2 h. After completion, Et_3N (2.8 μL, 0.020 mmol, 0.2 equiv) was added to the reaction mixture and stirred at rt for 2 h. After completion, the solvent was evaporated in vacuo. The residue was purified by flash chromatography on silica gel (EtOAc/petroleum ether = 1/8) to give product **3r**: 41.6 mg, 91% yield, as a yellow solid; mp: 115–116 °C; $[\alpha]_D^{25}$ = +79.2 (*c* = 1.5 in $CHCl_3$); >19:1 dr; 90% ee, determined by HPLC analysis [Chiralpak IA, *n*-hexane/*i*-PrOH = 80/20, 1.0 mL min^{-1}, λ = 254 nm, t (minor) = 17.17 min, t (major) = 19.02 min]; **^{1}H NMR** (400 MHz, $CDCl_3$): *δ* (ppm) 8.67 (d, *J* = 2.0 Hz, 1H), 8.26 (d, *J* = 2.0 Hz, 1H), 8.13 (dd, *J* = 8.8, 2.0 Hz, 1H), 8.01 (dd, *J* = 8.4, 2.0 Hz, 1H), 7.94 (d, *J* = 8.8 Hz, 1H), 7.90 (dd, *J* = 8.0, 2.8 Hz, 2H), 7.69–7.63 (m, 1H), 7.61–7.55 (m, 1H), 7.46–7.40 (m, 3H), 7.38–7.32 (m, 2H), 6.78 (d, *J* = 8.4 Hz, 1H), 5.18 (s, 1H), 3.96 (d, *J* = 17.6 Hz, 1H), 3.73 (d, *J* = 17.6 Hz, 1H), 3.65 (d, *J* = 23.2 Hz, 1H), 3.52 (dd, *J* = 23.2, 3.2 Hz, 1H); **^{13}C NMR** (100 MHz, $CDCl_3$): *δ* (ppm) 190.5, 153.6, 148.4, 146.1, 145.8, 145.5, 136.0, 135.9, 132.5, 132.1, 130.1, 129.9, 129.5, 129.2, 129.1, 129.0, 128.8, 127.8, 127.3, 125.0, 123.2, 119.9, 119.5, 119.2, 62.5, 54.4, 41.8, 35.8; **HRMS** (ESI-TOF) m/z: $[M + H]^+$ Calcd for $C_{30}H_{21}N_2O_3H^+$ 457.1552; Found 457.1546.

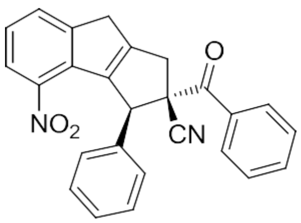

Synthesis of 3s: A flame-dried 10 mL Schlenk tube equipped with a magnetic stirring bar was cooled to room temperature. To this flask were added $Pd_2(dba)_3$ (4.6 mg, 0.0050 mmol, 0.05 equiv), **L4** (12.1 mg, 0.0200 mmol, 0.2 equiv), **A2** (5.0 mg, 0.020 mmol, 0.2 equiv) and 4 Å MS (100.0 mg), and the flask was evacuated and back-filled three times with argon. Then toluene (0.5 mL) was added by syringe. The mixture was stirred at 30 °C for 10 min. To another dry tube equipped with a magnetic stirring bar were added 2-methylene-4-nitro-2,3-dihydro-1*H*-inden-1-ol **1d** (19.1 mg, 0.100 mmol, 1.0 equiv) and (*E*)-2-benzoyl-3-phenylacrylonitrile **2a** (28.0 mg, 0.120 mmol, 1.2 equiv), evacuated and back-filled three times with argon. Then toluene (0.5 mL) was added by syringe. The mixture was stirred at rt for 5 min. Then the latter solution was added to the first Schlenk tube by syringe. The mixture was stirred at 30 °C for 5 h. After completion, Et_3N (2.8 μL, 0.020 mmol, 0.2 equiv) was added to the reaction mixture and stirred at rt for 2 h. After completion, the solvent was evaporated in vacuo. The residue was purified by flash chromatography on silica gel (EtOAc/petroleum ether = 1/8) to give product **3s**: 40.1 mg, 99% yield, as a yellow solid; mp: 120–121 °C; $[\alpha]_D^{25}$ = +101.5 (*c* = 1.4 in $CHCl_3$); >19:1 dr; 89% ee, determined by HPLC analysis [Chiralpak IA, *n*-hexane/*i*-PrOH = 80/20, 1.0 mL min^{-1}, λ = 254 nm, t (minor) = 15.94 min, t (major) = 16.96 min]; **^{1}H NMR** (400 MHz, $CDCl_3$): *δ* (ppm) 8.20–8.13 (m, 2H), 7.98 (d, *J* = 8.0 Hz, 1H), 7.66 (t, *J* = 7.4 Hz, 1H), 7.56–7.50 (m, 2H), 7.46–7.36 (m, 3H), 7.34–7.30 (m, 2H), 7.29–7.25 (m, 1H), 7.02 (d, *J* = 7.2 Hz, 1H), 5.21 (s, 1H), 4.05 (d, *J* = 23.2 Hz, 1H), 3.90 (dd, *J* = 24.0, 3.2 Hz, 1H), 3.80 (d, *J* = 17.6 Hz, 1H), 3.71 (d, *J* = 17.2 Hz, 1H); **^{13}C NMR** (100 MHz, $CDCl_3$): *δ* (ppm) 190.7, 149.5, 145.4, 144.7, 143.1, 142.5, 136.2, 134.4, 132.8, 130.0, 129.1, 129.0, 128.9, 128.8, 128.1, 125.1, 120.2, 119.7, 62.6, 53.8, 41.6, 37.2. **HRMS** (ESI-TOF) m/z: $[M – H]^-$ Calcd for $C_{26}H_{17}N_2O_3^-$ 405.1250; Found 405.1247.

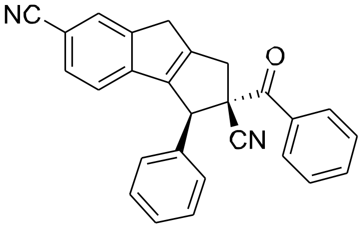

Synthesis of 3t: A flame-dried 10 mL Schlenk tube equipped with a magnetic stirring bar was cooled to room temperature. To this flask were added $Pd_2(dba)_3$ (4.6 mg, 0.0050 mmol, 0.05 equiv), **L4** (12.1 mg, 0.0200 mmol, 0.2 equiv), **A2** (5.0 mg, 0.020 mmol, 0.2 equiv) and 4 Å MS (100.0 mg), and the flask was evacuated and back-filled three times with argon. Then toluene (0.5 mL) was added by syringe. The mixture was stirred at 30 °C for 10 min. To another dry tube equipped with a magnetic stirring bar were added 3-hydroxy-2-methylene-2,3-dihydro-1*H*-indene-5-carbonitrile **1f** (17.1 mg, 0.100 mmol, 1.0 equiv) and (*E*)-2-benzoyl-3-phenylacrylonitrile **2a** (28.0 mg, 0.120 mmol, 1.2 equiv), evacuated and back-filled three times with argon. Then toluene (0.5 mL) was added by syringe. The mixture was stirred at rt for 5 min. Then the latter solution was added to the first Schlenk tube by syringe. The

mixture was stirred at 30 °C for 2 h. After completion, Et_3N (2.8 μL, 0.020 mmol, 0.2 equiv) was added to the reaction mixture and stirred at rt for 2 h. After completion, the solvent was evaporated in vacuo. The residue was purified by flash chromatography on silica gel (EtOAc/petroleum ether = 1/8) to give product **3t**: 38.5 mg, 99% yield, as a yellow solid; mp: 112–113 °C; $[\alpha]_D^{25}$ = +78.4 (*c* = 1.7 in $CHCl_3$); >19:1 dr; 89% ee, determined by HPLC analysis [Chiralpak IA, *n*-hexane/*i*-PrOH = 80/20, 1.0 mL min^{-1}, λ = 254 nm, t (minor) = 14.40 min, t (major) = 17.50 min]; **^{1}H NMR** (400 MHz, $CDCl_3$): *δ* (ppm) 8.15 (d, *J* = 4.4 Hz, 2H), 7.73–7.64 (m, 2H), 7.56–7.51 (m, 2H), 7.44–7.37 (m, 4H), 7.33–7.28 (m, 2H), 6.81 (d, *J* = 5.2 Hz, 1H), 5.17 (s, 1H), 3.83–3.66 (m, 2H), 3.64–3.40 (m, 2H); **^{13}C NMR** (100 MHz, $CDCl_3$): *δ* (ppm) 190.5, 151.4, 148.0, 146.3, 143.9, 136.0, 134.3, 132.7, 131.2, 130.0, 129.03, 129.02, 128.9, 128.8, 127.4, 119.9, 119.7, 119.6, 107.9, 62.4, 53.8, 41.6, 35.3; **HRMS** (ESI-TOF) m/z: $[M + Na]^+$ Calcd for $C_{27}H_{18}N_2ONa^+$ 409.1311; Found 409.1318.

5.2 Asymmetric [10+2] cycloadditions of allylic alcohol 1c with alkenes 4

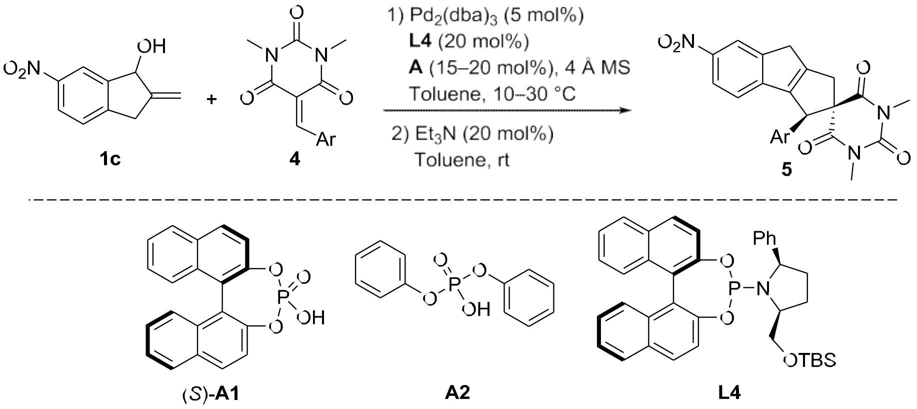

A flame-dried 10 mL Schlenk tube equipped with a magnetic stirring bar was cooled to room temperature. To this flask were added $Pd_2(dba)_3$ (0.0050 mmol), **L4** (0.0200 mmol), (*S*)-**A1** (0.020 mmol) or **A2** (0.015 mmol) and 4 Å MS (100.0 mg), and the flask was evacuated and back-filled three times with argon. Then toluene (0.5 mL) was added by syringe. The mixture was stirred at 30 °C for 10 min. To another dry tube equipped with a magnetic stirring bar were added **1c** (0.100 mmol) and **4** (0.120 mmol), evacuated and back-filled three times with argon. Then toluene (0.5 mL) was added by syringe. The mixture was stirred at rt for 5 min. Then the latter solution was added to the first Schlenk tube by syringe. The mixture was stirred at 10–30 °C for 2–12 h. After completion, Et_3N (0.020 mmol) was added to the reaction mixture and stirred at rt for 2 h. After completion, the solvent was evaporated in vacuo. The residue was purified by flash chromatography on silica gel (EtOAc/petroleum ether) to give the product.

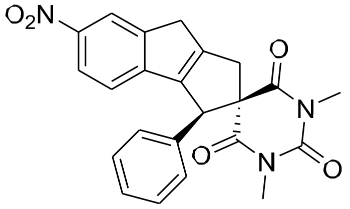

Synthesis of 5a: A flame-dried 10 mL Schlenk tube equipped with a magnetic stirring bar was cooled to room temperature. To this flask were added $Pd_2(dba)_3$ (4.6 mg, 0.0050 mmol, 0.05 equiv), **L4** (12.1 mg, 0.0200 mmol, 0.2 equiv), **A2** (3.8 mg, 0.015 mmol, 0.15 equiv) and 4 Å MS (100.0 mg), and the flask was evacuated and back-filled three times with argon. Then toluene (0.5 mL) was added by syringe. The mixture was stirred at 30 °C for 10 min. To another dry tube equipped with a magnetic stirring bar were added 2-methylene-6-nitro-2,3-dihydro-1*H*-inden-1-ol **1c** (19.1 mg, 0.100 mmol, 1.0 equiv) and 5-benzylidene-1,3-dimethylpyrimidine-2,4,6(1*H*,3*H*,5*H*)-trione **4a** (29.3 mg, 0.120 mmol, 1.0 equiv), evacuated and back-filled three times with argon. Then toluene (0.5 mL) was added by syringe. The mixture was stirred at rt for 5 min. Then the latter solution was added to the first Schlenk tube by syringe. The mixture was stirred at 15 °C for 3 h. After completion, Et_3N (2.8 μL, 0.020 mmol, 0.2 equiv) was added to the reaction mixture and stirred at rt for 2 h. After completion, the solvent was evaporated in vacuo. The residue was purified by flash chromatography on silica gel (EtOAc/petroleum ether = 1/5) to give product **5a**: 41.2 mg, 99% yield, as a yellow solid; mp:

138–139 °C; $[\alpha]_D^{25}$ = +96.6 (*c* = 1.4 in $CHCl_3$); 93% ee, determined by HPLC analysis [Chiralpak IA, *n*-hexane/*i*-PrOH = 80/20, 1.0 mL min^{-1}, λ = 254 nm, t (minor) = 17.48 min, t (major) = 14.40 min]; **^{1}H NMR** (400 MHz, $CDCl_3$): *δ* (ppm) δ 8.22 (d, *J* = 2.0 Hz, 1H), 7.93 (dd, *J* = 8.4, 2.0 Hz, 1H), 7.34–7.20 (m, 3H), 6.92 (d, *J* = 7.8 Hz, 2H), 6.54 (d, *J* = 8.4 Hz, 1H), 4.64 (s, 1H), 3.74–3.42 (m, 4H), 3.37 (s, 3H), 2.47 (s, 3H).); **^{13}C NMR** (100 MHz, $CDCl_3$): *δ* (ppm) 170.1, 167.6, 157.8, 149.8, 147.6, 145.2, 144.1, 141.0, 133.5, 128.1, 127.8, 127.6, 122.0, 118.5, 117.7, 67.9, 60.8, 35.3, 34.9, 28.2, 27.1; **HRMS** (ESI-TOF) m/z: $[M + Na]^+$ Calcd for $C_{23}H_{19}N_3O_5Na^+$ 440.1217; Found 440.1217.

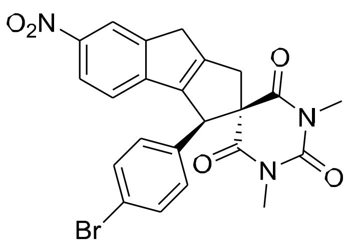

Synthesis of 5b: A flame-dried 10 mL Schlenk tube equipped with a magnetic stirring bar was cooled to room temperature. To this flask were added $Pd_2(dba)_3$ (4.6 mg, 0.0050 mmol, 0.05 equiv), **L4** (12.1 mg, 0.0200 mmol, 0.2 equiv), (*S*)-**A1** (7.0 mg, 0.020 mmol, 0.2 equiv) and 4 Å MS (100.0 mg), and the flask was evacuated and back-filled three times with argon. Then toluene (0.5 mL) was added by syringe. The mixture was stirred at 30 °C for 10 min. To another dry tube equipped with a magnetic stirring bar were added 2-methylene-6-nitro-2,3-dihydro-1*H*-inden-1-ol **1c** (19.1 mg, 0.100 mmol, 1.0 equiv) and 5-(4-bromobenzylidene)-1,3-dimethylpyrimidine-2,4,6(1*H*,3*H*,5*H*)-trione **4b** (38.7 mg, 0.120 mmol, 1.2 equiv), evacuated and back-filled three times with argon. Then toluene (0.5 mL) was added by syringe. The mixture was stirred at rt for 5 min. Then the latter solution was added to the first Schlenk tube by syringe. The mixture was stirred at 30 °C for 12 h. After completion, Et_3N (2.8 μL, 0.020 mmol, 0.2 equiv) was added to the reaction mixture and stirred at rt for 2 h. After completion, the solvent was evaporated in vacuo. The residue was purified by flash chromatography on silica gel (EtOAc/petroleum ether = 1/5) to give product **5b**: 29.7 mg, 60% yield; as a white solid; mp: 145–146 °C; $[\alpha]_D^{25}$ = +93.6 (*c* = 1.5 in $CHCl_3$); 94% ee, determined by HPLC analysis [Chiralpak IA, *n*-hexane/*i*-PrOH = 80/20, 1.0 mL min^{-1}, λ = 254 nm, t (minor) = 12.59 min, t (major) = 13.69 min]; **^{1}H NMR** (400 MHz, $CDCl_3$): *δ* (ppm) 8.28 (d, *J* = 2.0 Hz, 1H), 8.01 (dd, *J* = 8.4, 2.0 Hz, 1H), 7.45 (d, *J* = 8.0 Hz, 2H), 6.89 (d, *J* = 6.8 Hz, 2H), 6.60 (d, *J* = 8.4 Hz, 1H), 4.67 (s, 1H), 3.76–3.49 (m, 4H), 3.43 (s, 3H), 2.62 (s, 3H); **^{13}C NMR** (100 MHz, $CDCl_3$): *δ* (ppm) 171.0, 168.4, 159.2, 150.7, 148.6, 145.8, 145.2, 141.6, 133.6, 132.0, 130.2, 123.2, 123.1, 119.6, 118.6, 68.6, 60.9, 36.6, 35.9, 29.3, 28.2; **HRMS** (ESI-TOF) m/z: $[M + Na]^+$ Calcd for $C_{23}H_{18}BrN_3O_5Na^+$ 518.0328 (^{79}Br) and 520.0307 (^{81}Br); Found 518.0304 (^{79}Br) and 520.0287 (^{81}Br).

Synthesis of 5c: A flame-dried 10 mL Schlenk tube equipped with a magnetic stirring bar was cooled to room temperature. To this flask were added $Pd_2(dba)_3$ (4.6 mg, 0.0050 mmol, 0.05 equiv), **L4** (12.1 mg, 0.0200 mmol, 0.2 equiv), **A2** (3.8 mg, 0.015 mmol, 0.15 equiv) and 4 Å MS (100.0

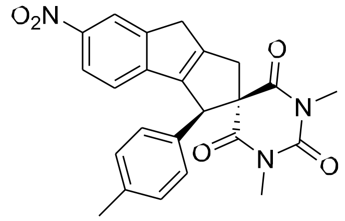

mg), and the flask was evacuated and back-filled three times with argon. Then toluene (0.5 mL) was added by syringe. The mixture was stirred at 30 °C for 10 min. To another dry tube equipped with a magnetic stirring bar were added 2-methylene-6-nitro-2,3-dihydro-1*H*-inden-1-ol **1c** (19.1 mg, 0.100 mmol, 1.0 equiv) and 1,3-dimethyl-5-(4-methylbenzylidene)pyrimidine-2,4,6(1*H*,3*H*,5*H*)-trione **4c** (31.0 mg, 0.120 mmol, 1.2 equiv), evacuated and back-filled three times with argon. Then toluene (0.5 mL) was added by syringe. The mixture was stirred at rt for 5 min. Then the latter solution was added to the first Schlenk tube by syringe. The mixture was stirred at 15 °C for 2 h. After completion, Et_3N (2.8 μL, 0.020 mmol, 0.2 equiv) was added to the reaction mixture and stirred at rt for 2 h. After completion, the solvent was evaporated in vacuo. The residue was purified by flash chromatography on silica gel (EtOAc/petroleum ether = 1/5) to give product **5c**: 42.8 mg, 99% yield, as a yellow solid; mp: 120–121 °C; $[\alpha]_D^{25}$ = +94.7 (*c* = 1.4 in $CHCl_3$); 93% ee, determined by HPLC analysis [Chiralpak IA, *n*-hexane/*i*-PrOH = 80/20, 1.0 mL min^{-1}, λ = 254 nm, t (minor) = 8.39 min, t (major) = 11.68 min]; **^{1}H NMR** (400 MHz, $CDCl_3$): δ (ppm) 8.27 (d, *J* = 2.0 Hz, 1H), 7.99 (dd, *J* = 8.4, 2.0 Hz, 1H), 7.11 (d, *J* = 7.6 Hz, 2H), 6.87 (d, *J* = 7.6 Hz, 2H), 6.61 (d, *J* = 8.4 Hz, 1H), 4.68 (s, 1H), 3.78–3.47 (m, 4H), 3.43 (s, 3H), 2.55 (s, 3H), 2.34 (s, 3H); **^{13}C NMR** (100 MHz, $CDCl_3$): δ (ppm) 170.1, 167.6, 157.8, 149.8, 147.6, 145.2, 144.1, 141.0, 133.5, 128.1, 127.8, 127.6, 122.0, 118.5, 117.7, 67.9, 60.8, 35.3, 34.9, 28.2, 27.1; **HRMS** (ESI-TOF) m/z: $[M + Na]^+$ Calcd for $C_{24}H_{21}N_3O_5Na^+$ 454.1373; Found 454.1362.

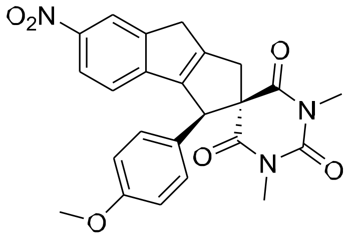

Synthesis of 5d: A flame-dried 10 mL Schlenk tube equipped with a magnetic stirring bar was cooled to room temperature. To this flask were added $Pd_2(dba)_3$ (4.6 mg, 0.0050 mmol, 0.05 equiv), **L4** (12.1 mg, 0.0200 mmol, 0.2 equiv), **A2** (3.8 mg, 0.015 mmol, 0.15 equiv) and 4 Å MS (100.0 mg), and the flask was evacuated and back-filled three times with argon. Then toluene (0.5 mL) was added by syringe. The mixture was stirred at 30 °C for 10 min. To another dry tube equipped with a magnetic stirring bar were added 2-methylene-6-nitro-2,3-dihydro-1*H*-inden-1-ol **1c** (19.1 mg, 0.100 mmol, 1.0 equiv) and 5-(4-methoxybenzylidene)-1,3-dimethylpyrimidine-2,4,6(1*H*,3*H*,5*H*)-trione **4d** (32.9 mg, 0.100 mmol, 1.0 equiv), evacuated and back-filled three times with argon. Then toluene (0.5 mL) was added by syringe. The mixture was stirred at rt for 5 min. Then the latter solution was added to the first Schlenk tube by syringe. The mixture was stirred at 15 °C for 2 h. After completion, Et_3N (2.8 μL, 0.020 mmol, 0.2 equiv) was added to the reaction mixture and stirred at rt for 2 h. After completin, the solvent was evaporated in vacuo. The residue was purified by flash chromatography

on silica gel (EtOAc/petroleum ether = 1/5) to give product **5d**: 44.6 mg, 99% yield, as a yellow solid; mp: 131–132 °C; $[\alpha]_D^{25}$ = +98.4 (*c* = 1.5 in $CHCl_3$); 80% ee, determined by HPLC analysis [Chiralpak IA, *n*-hexane/*i*-PrOH = 80/20, 1.0 mL min^{-1}, λ = 254 nm, t (minor) = 14.40 min, t (major) = 19.63 min]; **^{1}H NMR** (400 MHz, $CDCl_3$): *δ* (ppm) 8.28 (d, *J* = 2.0 Hz, 1H), 7.99 (dd, *J* = 8.4, 2.0 Hz, 1H), 6.97–6.87 (m, 2H), 6.86–6.79 (m, 2H), 6.62 (d, *J* = 8.4 Hz, 1H), 4.67 (s, 1H), 3.80 (s, 3H), 3.74–3.49 (m, 4H), 3.43 (s, 3H), 2.61 (s, 3H); **^{13}C NMR** (100 MHz, $CDCl_3$): *δ* (ppm) 171.3, 168.7, 160.1, 158.6, 150.9, 148.6, 146.3, 145.1, 142.3, 129.8, 126.3, 123.0, 119.5, 118.7, 114.2, 69.0, 61.4, 55.3, 36.1, 35.9, 29.2, 28.3; **HRMS** (ESI-TOF) m/z: $[M + Na]^+$ Calcd for $C_{24}H_{21}N_3O_6Na^+$ 470.1323; Found 470.1314.

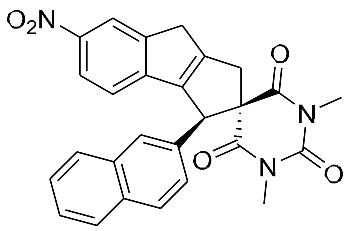

Synthesis of 5e: A flame-dried 10 mL Schlenk tube equipped with a magnetic stirring bar was cooled to room temperature. To this flask were added $Pd_2(dba)_3$ (4.6 mg, 0.0050 mmol, 0.05 equiv), **L4** (12.1 mg, 0.0200 mmol, 0.2 equiv), **A2** (3.8 mg, 0.015 mmol, 0.15 equiv) and 4 Å MS (100.0 mg), and the flask was evacuated and back-filled three times with argon. Then toluene (0.5 mL) was added by syringe. The mixture was stirred at 30 °C for 10 min. To another dry tube equipped with a magnetic stirring bar were added 2-methylene-6-nitro-2,3-dihydro-1*H*-inden-1-ol **1c** (19.1 mg, 0.100 mmol, 1.0 equiv) and 1,3-dimethyl-5-(naphthalen-2-ylmethylene)pyrimidine-2,4,6(1*H*,3*H*,5*H*)-trione **4e** (35.9 mg, 0.122 mmol, 1.2 equiv), evacuated and back-filled three times with argon. Then toluene (0.5 mL) was added by syringe. The mixture was stirred at rt for 5 min. Then the latter solution was added to the first Schlenk tube by syringe. The mixture was stirred at 14 °C for 2 h. After completion, Et_3N (2.8 μL, 0.020 mmol, 0.2 equiv) was added to the reaction mixture and stirred at rt for 2 h. After completion, the solvent was evaporated in vacuo. The residue was purified by flash chromatography on silica gel (EtOAc/petroleum ether = 1/5) to give product **5e**: 35.2mg, 75% yield, as a yellow solid; mp: 135–136 °C; $[\alpha]_D^{25}$ = +96.4 (*c* = 1.6 in $CHCl_3$); 93% ee, determined by HPLC analysis [Chiralpak IA, *n*-hexane/*i*-PrOH = 80/20, 1.0 mL min^{-1}, λ = 254 nm, t (minor) = 22.11 min, t (major) = 26.36 min]; **^{1}H NMR** (400 MHz, $CDCl_3$): *δ* (ppm) 8.30 (d, *J* = 2.0 Hz, 1H), 7.94 (dd, *J* = 8.4, 2.0 Hz, 1H), 7.85–7.70 (m, 3H), 7.55–7.45 (m, 3H), 7.07 (s, 1H), 6.59 (d, *J* = 8.4 Hz, 1H), 4.89 (s, 1H), 3.95–3.52 (m, 4H), 3.47 (s, 3H), 2.31 (s, 3H); **^{13}C NMR** (100 MHz, $CDCl_3$): *δ* (ppm) 171.2, 168.6, 159.0, 150.8, 148.6, 146.3, 145.1, 142.1, 133.2, 133.0, 131.9, 128.6, 128.1, 128.0, 127.7, 126.9, 125.8, 123.1, 119.5, 118.8, 69.0, 61.9, 36.4, 36.0, 29.3, 28.1. **HRMS** (ESI-TOF) m/z: $[M + Na]^+$ Calcd for $C_{27}H_{21}N_3O_5Na^+$ 490.1373; Found 490.1372.

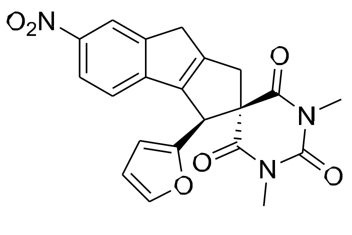

Synthesis of 5f: A flame-dried 10 mL Schlenk tube equipped with a magnetic stirring bar was cooled to room temperature. To this flask were added $Pd_2(dba)_3$ (4.6 mg, 0.0050 mmol, 0.05 equiv), **L4** (12.1 mg, 0.0200 mmol, 0.2 equiv), **A2** (3.8 mg, 0.015 mmol, 0.15 equiv) and 4 Å MS (100.0 mg), and the flask was evacuated and back-filled three times with argon. Then toluene (0.5 mL) was added by syringe. The mixture was stirred at 30 °C for 10 min. To another dry tube equipped with a magnetic stirring bar were added 2-methylene-6-nitro-2,3-dihydro-1*H*-inden-1-ol **1c** (19.1 mg, 0.100 mmol, 1.0 equiv), 5-(furan-2-ylmethylene)-1,3-dimethylpyrimidine-2,4,6(1*H*,3*H*,5*H*)-trione **4f** (28.1 mg, 0.120 mmol,
1.2 equiv) and evacuated and back-filled three times with argon. Then toluene (0.5 mL) was added by syringe. The mixture was stirred at rt for 5 min. Then the latter solution was added to the first Schlenk tube by syringe. The mixture was stirred at 10 °C for 2 h. After completion, Et_3N (2.8 μL, 0.020 mmol, 0.2 equiv) was added to the reaction mixture and stirred at rt for 2 h. After completion, the solvent was evaporated in vacuo. The residue was purified by flash chromatography on silica gel (EtOAc/petroleum ether = 1/5) to give product **5f**: 36.3 mg, 89% yield, as a white solid; mp: 127–128 °C; $[\alpha]_D^{25}$ = +92.8 (*c* = 1.2 in $CHCl_3$); 96% ee, determined by HPLC analysis [Chiralpak IA, *n*-hexane/*i*-PrOH = 80/20, 1.0 mL min^{-1}, λ = 254 nm, t (major) = 19.90 min, t (minor) = 24.15 min]; ^{1}**H NMR** (400 MHz, $CDCl_3$): δ (ppm) 8.29 (d, *J* = 2.4 Hz, 1H), 8.09 (d, *J* = 7.6 Hz, 1H), 7.40 (s, 1H), 6.91 (d, *J* = 8.4 Hz, 1H), 6.36 (s, 1H), 6.08 (d, *J* = 3.2 Hz, 1H), 4.85 (s, 1H), 3.72–3.47 (m, 4H), 3.44 (s, 3H), 2.88 (s, 3H); ^{13}**C NMR** (100 MHz, $CDCl_3$): δ (ppm) 170.8, 168.5, 159.0, 151.0, 148.9, 148.5, 146.0, 145.3, 143.4, 139.8, 123.1, 119.5, 118.7, 111.1, 110.4, 67.7, 54.4, 36.5, 35.9, 29.3, 28.8; **HRMS** (ESI-TOF) m/z: $[M + Na]^+$ Calcd for $C_{21}H_{17}N_3O_6Na^+$ 430.1010; Found 430.1020.

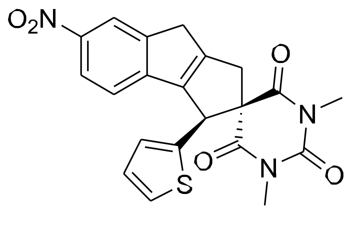

Synthesis of 5g: A flame-dried 10 mL Schlenk tube equipped with a magnetic stirring bar was cooled to room temperature. To this flask were added $Pd_2(dba)_3$ (4.6 mg, 0.0050 mmol, 0.05 equiv), **L4** (12.1 mg, 0.0200 mmol, 0.2 equiv), (*S*)-**A1** (7.0 mg, 0.020 mmol, 0.2 equiv) and 4 Å MS (100.0 mg), and the flask was evacuated and back-filled three times with argon. Then toluene (0.5 mL) was added by syringe. The mixture was stirred at 30 °C for 10 min. To another dry tube equipped with a magnetic stirring bar were added 2-methylene-6-nitro-2,3-dihydro-1*H*-inden-1-ol **1c** (19.1 mg, 0.100 mmol, 1.0 equiv) and 1,3-dimethyl-5-(thiophen-2-ylmethylene)pyrimidine-2,4,6(1*H*,3*H*,5*H*)-trione **4g** (30.0 mg, 0.120 mmol, 1.2 equiv), evacuated and back-filled three times with argon. Then toluene (0.5 mL) was added by syringe. The mixture was stirred at rt for 5 min. Then the latter solution was added to the

first Schlenk tube by syringe. The mixture was stirred at 30 °C for 12 h. After completion, Et_3N (2.8 μL, 0.020 mmol, 0.2 equiv) was added to the reaction mixture and stirred at rt for 2 h. After completion, the solvent was evaporated in vacuo. The residue was purified by flash chromatography on silica gel (EtOAc/petroleum ether = 1/5) to give product **5g**: 27.1 mg, 64% yield, as a yellow solid; mp: 133–134 °C; $[\alpha]_D^{25}$ = +95.6 (*c* = 1.4 in $CHCl_3$); 87% ee, determined by HPLC analysis [Chiralpak IA, *n*-hexane/*i*-PrOH = 80/20, 1.0 mL min^{-1}, λ = 254 nm, t (minor) = 10.88 min, t (major) = 14.17 min]; **^{1}H NMR** (400 MHz, $CDCl_3$): *δ* (ppm) 8.28 (d, *J* = 2.0 Hz, 1H), 8.04 (dd, *J* = 8.4, 2.0 Hz, 1H), 7.30 (d, *J* = 4.8 Hz, 1H), 7.01–6.96 (m, 1H), 6.79–6.72 (m, 2H), 4.99 (s, 1H), 3.78–3.48 (m, 4H), 3.43 (s, 3H), 2.76 (s, 3H); **^{13}C NMR** (100 MHz, $CDCl_3$): *δ* (ppm) 170.9, 168.5, 158.7, 150.9, 148.5, 146.0, 145.2, 141.8, 136.6, 128.0, 127.5, 126.6, 123.0, 119.5, 118.8, 69.1, 56.2, 36.5, 35.9, 29.3, 28.5; **HRMS** (ESI-TOF) m/z: $[M + Na]^+$ Calcd for $C_{21}H_{17}N_3O_5SNa^+$ 446.0781; Found 446.0791.

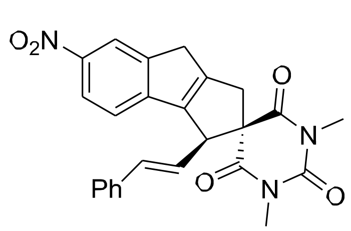

Synthesis of 5h: A flame-dried 10 mL Schlenk tube equipped with a magnetic stirring bar was cooled to room temperature. To this flask were added $Pd_2(dba)_3$ (4.6 mg, 0.0050 mmol, 0.05 equiv), **L4** (12.1 mg, 0.0200 mmol, 0.2 equiv), **A2** (3.8 mg, 0.015 mmol, 0.15 equiv) and 4 Å MS (100.0 mg), and the flask was evacuated and back-filled three times with argon. Then toluene (0.5 mL) was added by syringe. The mixture was stirred at rt for 10 min. To another dry tube equipped with a magnetic stirring bar were added 2-methylene-6-nitro-2,3-dihydro-1*H*-inden-1-ol **1c** (19.1 mg, 0.100 mmol, 1.0 equiv) and (*E*)-1,3-dimethyl-5-(3-phenylallylidene)pyrimidine-2,4,6(1*H*,3*H*,5*H*)-trione **4h** (32.4 mg, 0.120 mmol, 1.2 equiv), evacuated and back-filled three times with argon. Then toluene (0.5 mL) was added by syringe. The mixture was stirred at 30 °C for 5 min. Then the latter solution was added to the first Schlenk tube by syringe. The mixture was stirred at 15 °C for 2 h. After completion, Et_3N (2.8 μL, 0.020 mmol, 0.2 equiv) was added to the reaction mixture and stirred at rt for 2 h. After completion, the solvent was evaporated in vacuo. The residue was purified by flash chromatography on silica gel (EtOAc/petroleum ether = 1/5) to give product **5h**: 40.5 mg, 91% yield, as a yellow solid; mp: 130–131 °C; $[\alpha]_D^{25}$ = +97.6 (*c* = 1.5 in $CHCl_3$); 80% ee, determined by HPLC analysis [Chiralpak IA, *n*-hexane/*i*-PrOH = 80/20, 1.0 mL min^{-1}, λ = 254 nm, t (major) = 13.32 min, t (minor) = 14.42 min]; **^{1}H NMR** (400 MHz, $CDCl_3$): *δ* (ppm) 8.26 (d, *J* = 2.0 Hz, 1H), 8.09 (dd, *J* = 7.6, 1.6 Hz, 1H), 7.37–7.27 (m, 5H), 7.11 (d, *J* = 8.0 Hz, 1H), 6.66 (d, *J* = 16.0 Hz, 1H), 6.07 (dd, *J* = 16.0, 9.6 Hz, 1H), 4.24 (d, *J* = 9.2 Hz, 1H), 3.59–3.49 (m, 4H), 3.41 (s, 3H), 3.14 (s, 3H); **^{13}C NMR** (100 MHz, $CDCl_3$): *δ* (ppm) 171.2, 169.0, 157.7, 151.2, 148.6, 146.4, 145.2, 142.5, 136.1, 135.5, 128.9,

128.7, 126.7, 123.9, 123.1, 119.5, 118.3, 67.3, 58.8, 36.7, 35.7, 29.3, 28.8; **HRMS** (ESI-TOF) m/z: $[M + Na]^+$ Calcd for $C_{25}H_{21}N_3O_5Na^+$ 466.1373; Found 466.1362.

5.3 Asymmetric [10+2] cycloaddition of allylic alcohol 1c with alkene 6

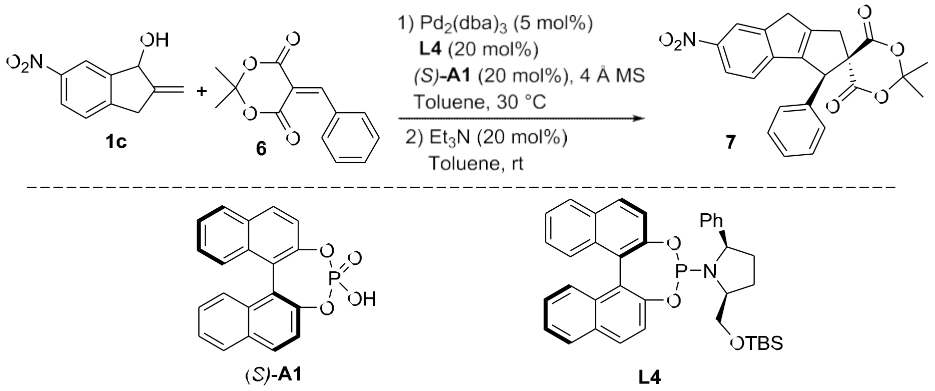

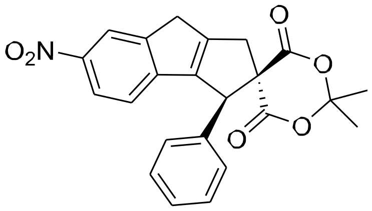

Synthesis of 7: A flame-dried 10 mL Schlenk tube equipped with a magnetic stirring bar was cooled to room temperature. To this flask were added $Pd_2(dba)_3$ (4.6 mg, 0.0050 mmol, 0.05 equiv), **L4** (12.1 mg, 0.020 mmol, 0.2 equiv), (*S*)-**A1** (7.0 mg, 0.020 mmol, 0.2 equiv) and 4 Å MS (100.0 mg), and the flask was evacuated and back-filled three times with argon. Then toluene (0.5 mL) was added by syringe. The mixture was stirred at 30 °C for 10 min. To another dry tube equipped with a magnetic stirring bar were added 2-methylene-6-nitro-2,3-dihydro-1*H*-inden-1-ol **1c** (19.1 mg, 0.100 mmol, 1.0 equiv) and 5-benzylidene-2,2-dimethyl-1,3-dioxane-4,6-dione **6** (27.8 mg, 0.120 mmol, 1.2 equiv), evacuated and back-filled three times with argon. Then toluene (0.5 mL) was added by syringe. The mixture was stirred at 30 °C for 5 min. Then the latter solution was added to the first Schlenk tube by syringe. The mixture was stirred at 30 °C for 16 h. After completion, Et_3N (2.8 μL, 0.020 mmol, 0.2 equiv) was added to the reaction mixture and stirred at rt for 2 h. After completion, the solvent was evaporated in vacuo. The residue was purified by flash chromatography on silica gel (EtOAc/petroleum ether = 1/7) to give product **7**: 26.7 mg, 66% yield, as a white solid; mp: 146–147 °C; $[\alpha]_D^{25}$ = +78.5 (*c* = 1.3 in $CHCl_3$); 87% ee, determined by HPLC analysis [Chiralpak IA, *n*-hexane/*i*-PrOH = 80/20, 1.0 mL min^{-1}, λ = 254 nm, t (minor) = 15.57 min, t (major) = 22.12 min]; **^{1}H NMR** (400 MHz, $CDCl_3$): *δ* (ppm) 8.22 (d, *J* = 2.0 Hz, 1H), 7.93 (dd, *J* = 8.4, 2.0 Hz, 1H), 7.33–7.20 (m, 3H), 6.92 (d, *J* = 7.8 Hz, 2H), 6.54 (d, *J* = 8.4 Hz, 1H), 4.65 (s, 1H), 3.75–3.42 (m, 3H), 3.37 (s, 3H), 2.47 (s, 3H); **^{13}C NMR** (100 MHz, $CDCl_3$): *δ* (ppm) 170.3, 168.9, 157.2, 149.8, 149.5, 148.2, 134.4, 129.2, 129.1, 128.7, 123.5, 123.1, 119.8, 115.9, 105.6, 65.5, 58.6, 57.5, 36.5, 30.3, 27.6; **HRMS** (ESI-TOF) m/z: $[M + Na]^+$ Calcd for $C_{23}H_{19}N_3O_6Na^+$ 428.1105; Found 428.1100.

5.4 Asymmetric [10+8] cycloaddition of allylic alcohol 1c with alkene 8

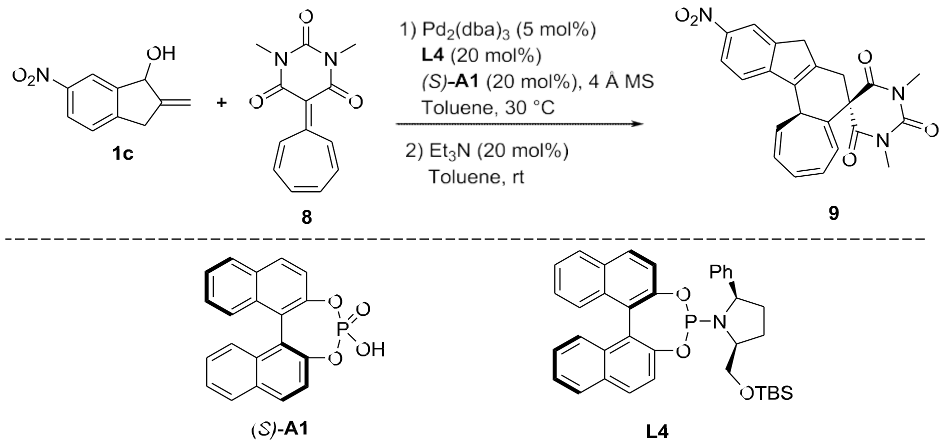

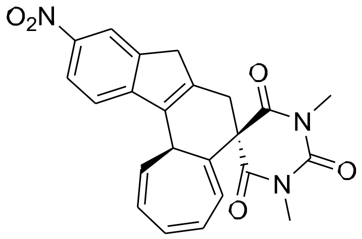

Synthesis of 9: A flame-dried 10 mL Schlenk tube equipped with a magnetic stirring bar was cooled to room temperature. To this flask were added $Pd_2(dba)_3$ (4.6 mg, 0.0050 mmol, 0.05 equiv), **L4** (12.1 mg, 0.020 mmol, 0.2 equiv), (*S*)-**A1** (7.0 mg, 0.020 mmol, 0.2 equiv) and 4 Å MS (100.0 mg), and the flask was evacuated and back-filled three times with argon. Then toluene (0.5 mL) was added by syringe. The mixture was stirred at 30 °C for 10 min. To another dry tube equipped with a magnetic stirring bar were added 2-methylene-6-nitro-2,3-dihydro-1*H*-inden-1-ol **1c** (22.9 mg, 0.120 mmol, 1.2 equiv) and 5-(cyclohepta-2,4,6-trien-1-ylidene)-1,3-dimethylpyrimidine-2,4,6(1*H*,3*H*,5*H*)-trione **8** (24.4 mg, 0.100 mmol, 1.0 equiv), evacuated and back-filled three times with argon. Then toluene (0.5 mL) was added by syringe. The mixture was stirred at rt for 5 min. Then the latter solution was added to the first Schlenk tube by syringe. The mixture was stirred at 30 °C for 14 h. After completion, Et_3N (2.8 μL, 0.020 mmol, 0.2 equiv) was added to the reaction mixture and stirred at rt for 2 h. After completion, the solvent was evaporated in vacuo. The residue was purified by flash chromatography on silica gel (EtOAc/petroleum ether = 1/6) to give product **9**: 25.8 mg, 62% yield, as a white solid; mp: 146–147 °C; $[\alpha]_D^{25}$ = +78.5 (*c* = 1.3 in $CHCl_3$); 93% ee, determined by HPLC analysis [Chiralpak AD-H, *n*-hexane/*i*-PrOH = 90/10, 1.0 mL min^{-1}, λ = 254 nm, t (major) = 9.25 min, t (minor) = 11.90 min]; **^{1}H NMR** (400 MHz, $CDCl_3$): *δ* (ppm) 8.30 (d, J = 2.0 Hz, 1H), 8.19 (dd, *J* = 8.4, 2.0 Hz, 1H), 7.30 (d, *J* = 8.3 Hz, 1H), 6.84 (dd, *J* = 11.2, 5.2 Hz, 1H), 6.67 (dd, *J* = 11.2, 6.0 Hz, 1H), 6.41–6.28 (m, 1H), 6.02 (d, *J* = 5.6 Hz, 1H), 5.32 (dd, *J* = 9.2, 6.8 Hz, 1H), 3.68–3.62 (m, 3H), 3.44 (s, 3H), 3.10 (m, 4H), 2.79 (d, *J* = 3.2 Hz, 1H); **^{13}C NMR** (100 MHz, $CDCl_3$): *δ* (ppm) 170.8, 169.5, 150.8, 150.7, 145.4, 145.2, 143.5, 133.9, 132.2, 129.8, 128.9, 128.6, 126.0, 123.1, 122.5, 118.9, 118.1, 56.0, 40.7, 38.0, 32.2, 29.4, 29.1; **HRMS** (ESI-TOF) m/z: $[M + Na]^+$ Calcd for $C_{23}H_{19}N_3O_5Na^+$ 440.1222; Found 440.1217.

5.5 Asymmetric [10+4] and [10+2] cycloadditions of allylic carbonate 1g with alkene 10

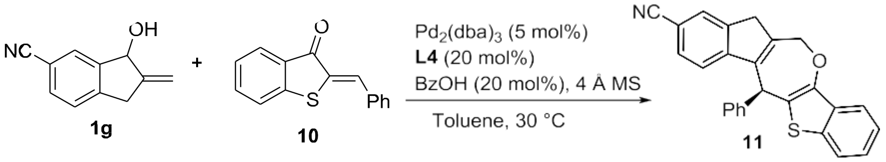

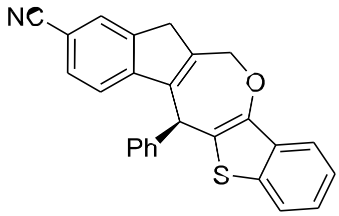

Synthesis of 11: A flame-dried 10 mL Schlenk tube equipped with a magnetic stirring bar was cooled to room temperature. To this flask were added $Pd_2(dba)_3$ (4.6 mg, 0.0050 mmol, 0.05 equiv), **L4** (12.1 mg, 0.0200 mmol, 0.2 equiv), BzOH (2.4 mg, 0.020 mmol, 0.2 equiv) and 4 Å MS (100.0 mg), and the flask was evacuated and back-filled three times with argon. Then toluene (0.5 mL) was added by syringe. The mixture was stirred at 30 °C for 10 min. To another dry tube equipped with a magnetic stirring bar were added *tert*-butyl (6-cyano-2-methylene-2,3-dihydro-1*H*-inden-1-yl) carbonate **1g** (27.1 mg, 0.100 mmol, 1.0 equiv) and (*Z*)-2-benzylidenebenzo[*b*]thiophen-3(2*H*)-one **10** (28.6 mg, 0.120 mmol, 1.2 equiv), evacuated and back-filled three times with argon. Then toluene (0.5 mL) was added by syringe. The mixture was stirred at rt for 5 min. Then the latter solution was added to the first Schlenk tube by syringe. After completion, the solvent was evaporated in vacuo. The residue was purified by flash chromatography on silica gel (EtOAc/petroleum ether = 1/15) to give product **10**: 21.2 mg, 54% yield, as a white solid; mp: 110–111 °C; $[\alpha]_D^{25}$ = –59.2 (*c* = 0.6 in $CHCl_3$); 87% ee, determined by HPLC analysis [Chiralpak IA, *n*-hexane/*i*-PrOH = 95/5, 1.0 mL min^{-1}, λ = 254 nm, t (minor) = 22.75 min, t (major) = 24.87 min]; **^{1}H NMR** (400 MHz, $CDCl_3$): *δ* (ppm) 7.73 (d, *J* = 7.2 Hz, 1H), 7.70–7.60 (m, 2H), 7.52–7.49 (m, 3H), 7.38–7.31 (m, 1H), 7.31–7.26 (m, 3H), 7.19–7.17 (m, 2H), 5.34–5.26 (m, 2H), 5.03 (d, *J* = 15.6 Hz, 1H), 3.56 (s, 2H); **^{13}C NMR** (100 MHz, $CDCl_3$): *δ* (ppm) 149.3, 146.7, 144.9, 142.2, 141.9, 137.7, 135.4, 134.3, 131.2, 129.0, 127.7, 127.4, 126.9, 125.0, 124.6, 124.2, 122.4, 120.7, 119.9, 119.7, 108.1, 69.2, 44.1, 40.1; **HRMS** (ESI-TOF) m/z: $[M+H]^+$ Calcd for $C_{26}H_{18}NOS^+$ 392.1104; Found 392.1103.

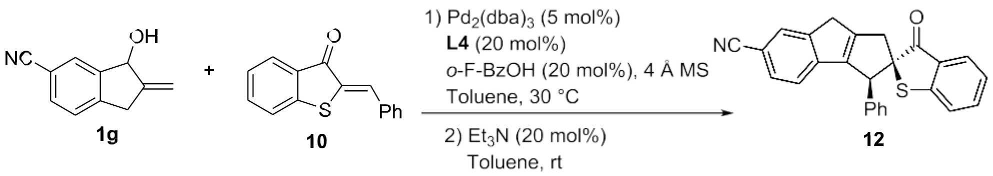

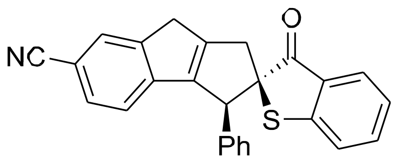

Synthesis of 12: A flame-dried 10 mL Schlenk tube equipped with a magnetic stirring bar was cooled to room temperature. To this flask were added $Pd_2(dba)_3$ (4.6 mg, 0.0050 mmol, 0.05 equiv), **L4** (12.1 mg, 0.0200 mmol, 0.2 equiv), *o*-F-BzOH (2.8 mg, 0.020 mmol, 0.2 equiv) and 4 Å MS (100.0 mg), and the flask was evacuated and back-filled three times with argon. Then toluene (0.5 mL) was added by syringe. The mixture was stirred at 30 °C for 10

min. To another dry tube equipped with a magnetic stirring bar were added *tert*-butyl (6-cyano-2-methylene-2,3-dihydro-1*H*-inden-1-yl) carbonate **1g** (27.1 mg, 0.100 mmol, 1.0 equiv) and (*Z*)-2-benzylidenebenzo[*b*]thiophen-3(2*H*)-one **10** (28.6 mg, 0.120 mmol, 1.2 equiv), evacuated and back-filled three times with argon. Then toluene (0.5 mL) was added by syringe. The mixture was stirred at rt for 5 min. Then the latter solution was added to the first Schlenk tube by syringe. The mixture was stirred at 30 °C for 6 h. After completion, the solvent was evaporated in vacuo. The residue was purified by flash chromatography on silica gel (EtOAc/petroleum ether = 1/15) to give the unisomerized [10+2] product. The unisomerized [10+2] product was dissolved in toluene (0.5ml), and Et_3N (2.8 μL, 0.020 mmol, 0.2 equiv) was added to the reaction mixture. The mixture was stirred at rt for 2 h. After completion, the solvent was evaporated in vacuo. The residue was purified by flash chromatography on silica gel (EtOAc/petroleum ether = 1/10) to give product **12**: 18.4 mg, 47% yield, as a white solid; mp: 67–69 °C; $[\alpha]_D^{25}$ = +112.0 (*c* = 0.6 in $CHCl_3$); 86% ee, determined by HPLC analysis [Chiralpak IA, *n*-hexane/*i*-PrOH = 95/5, 1.0 mL min^{-1}, λ = 254 nm, t (minor) = 30.94 min, t (major) = 32.53 min]; **^{1}H NMR** (600 MHz, $CDCl_3$): *δ* (ppm) 7.88–7.76 (m, 1H), 7.69 (d, *J* = 3.6 Hz, 1H), 7.54–7.36 (m, 2H), 7.25–7.18 (m, 4H), 7.14 (dd, *J* = 8.4, 4.2 Hz, 1H), 7.03–7.00 (m, 2H), 6.86 (dd, *J* = 8.4, 4.2 Hz, 1H), 4.85 (s, 1H), 3.65–3.51 (m, 2H), 3.46 (d, *J* = 23.4 Hz, 1H), 3.35 (d, *J* = 23.4 Hz, 1H); **^{13}C NMR** (150 MHz, $CDCl_3$): *δ* (ppm) 203.8, 153.4, 152.1, 148.3, 147.1, 144.9, 137.6, 136.1, 131.2, 129.6, 129.0, 128.8, 128.3, 127.8, 127.3, 127.1, 124.8, 123.8, 120.1, 119.9, 107.4, 56.1, 44.4, 35.6; **HRMS** (ESI-TOF) m/z: $[M+H]^+$ Calcd for $C_{26}H_{18}NOS^+$ 392.1104; Found 392.1109.

6. More screening studies for substrate scope

6.1 More screening studies on other allylic alcohols

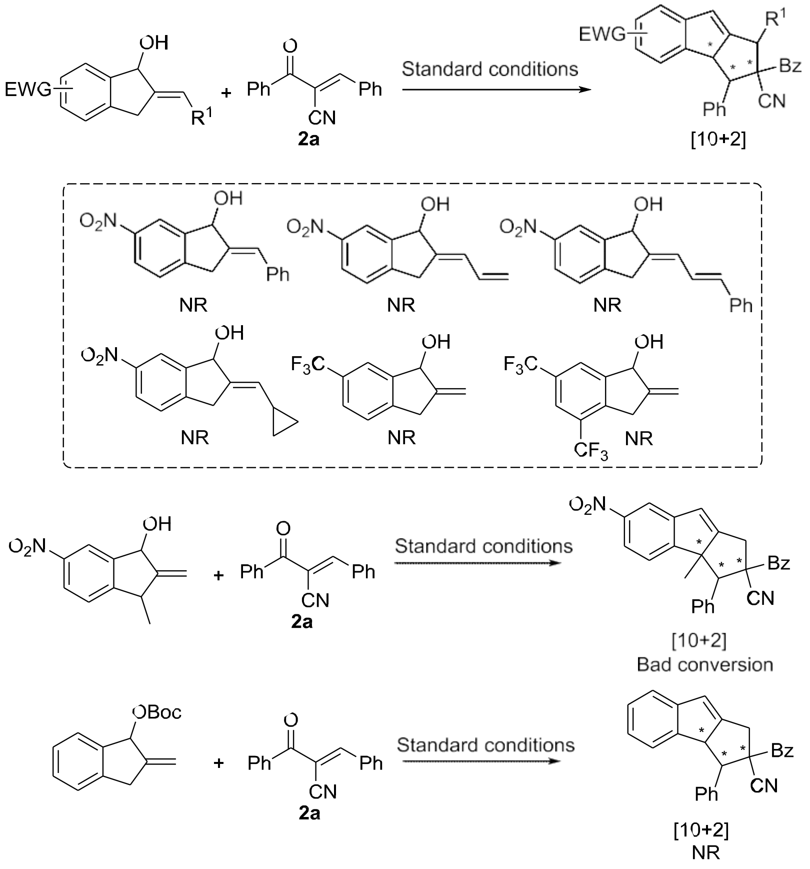

Reactions were performed with allylic alcohol or allyl *tert*-butyl carbonate (0.1 mmol), activated alkene **2a** (0.12 mmol), $Pd_2(dba)_3$ (5 mol%), **L4** (20 mol%), acid (*S*)-**A1** (20 mol%) and 4 Å MS (100 mg) in toluene (1 mL) at 30 °C.

6.2 More screening studies on activated alkenes 2

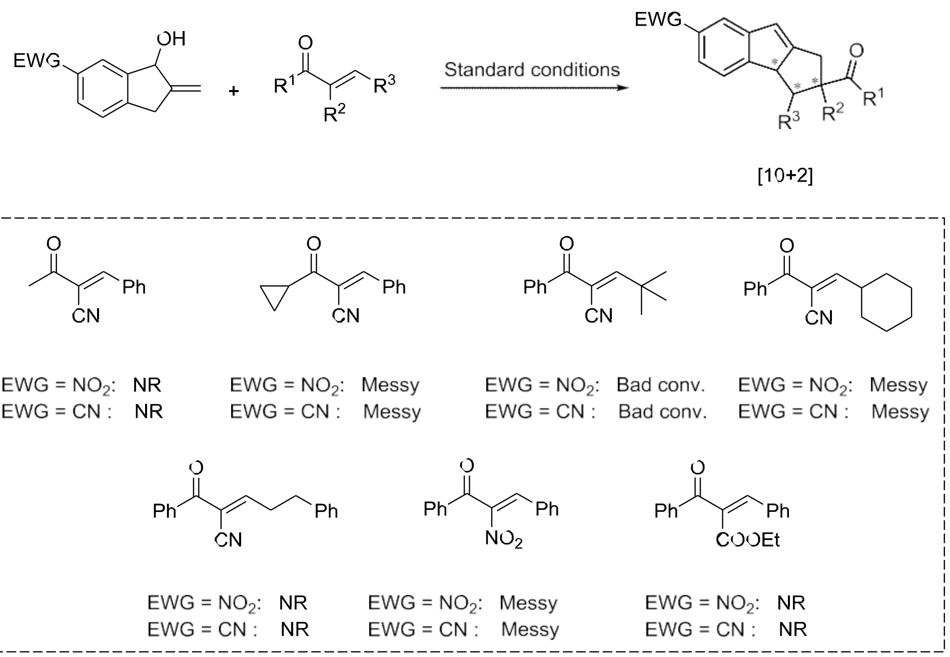

Reactions were performed with allylic alcohol (0.1 mmol), activated alkene **2** (0.12 mmol), $Pd_2(dba)_3$ (5 mol%), **L4** (20 mol%), acid (*S*)-**A1** (20 mol%) and 4 Å MS (100 mg) in toluene (1 mL) at 30 °C.

6.3 More screening studies on tropone derivatives

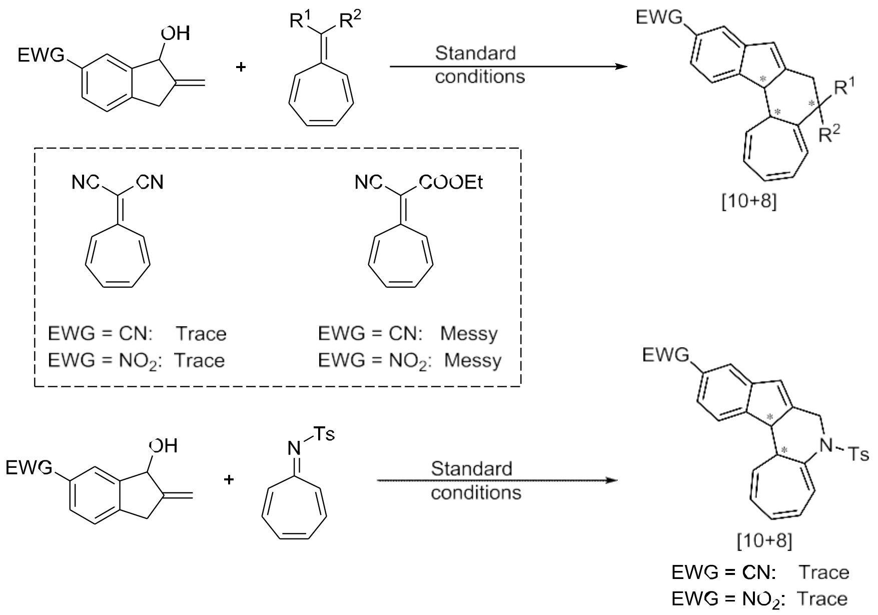

Reactions were performed with allylic alcohol (0.1 mmol), tropone derivative (0.12 mmol), $Pd_2(dba)_3$ (5 mol%), **L4** (20 mol%), acid (*S*)-**A1** (20 mol%) and 4 Å MS (100 mg) in toluene (1 mL) at 30 °C.

6.4 More screening studies on other electrophiles

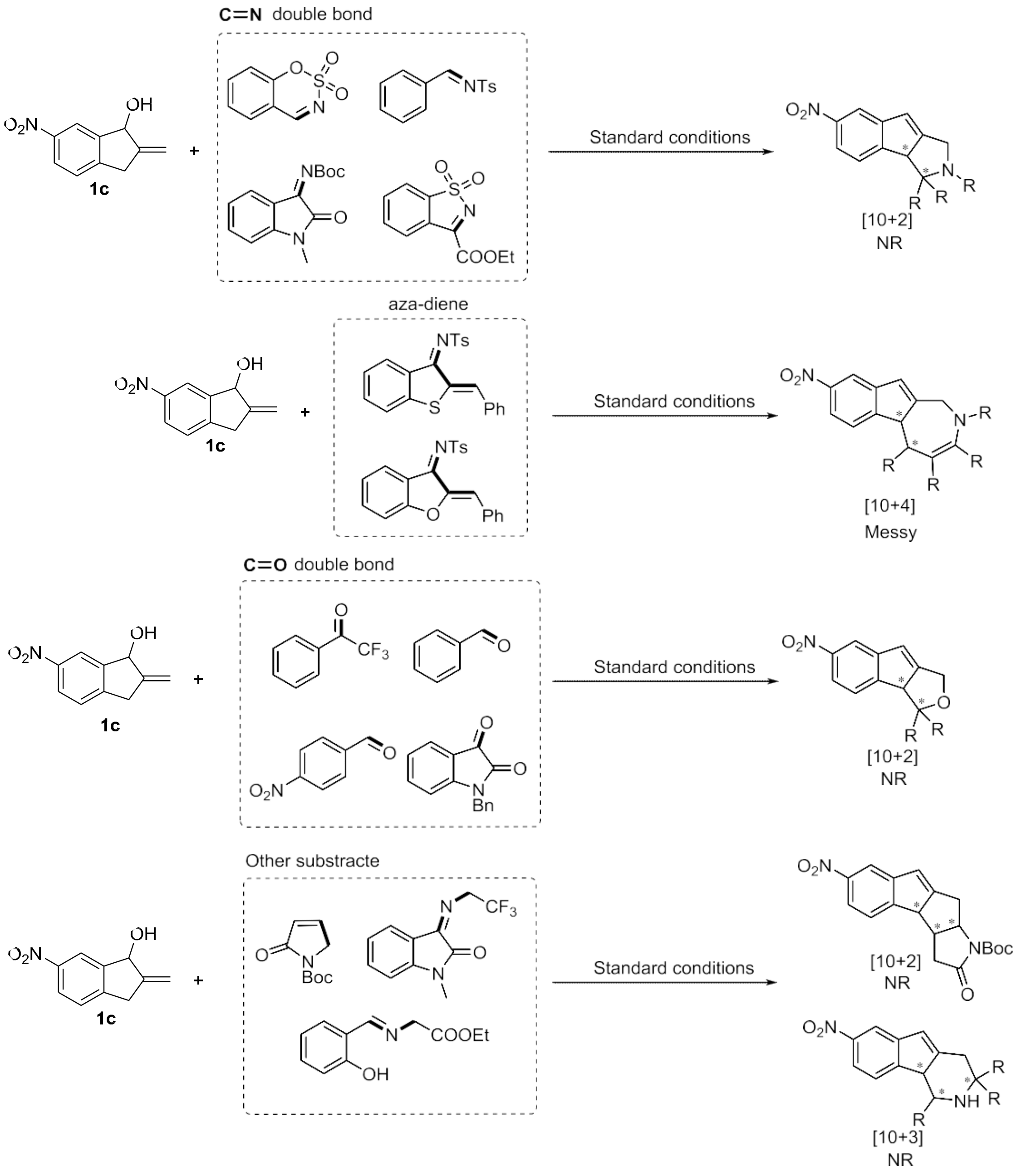

Reactions were performed with allylic alcohol **1c** (0.1 mmol), electrophile (0.12 mmol), $Pd_2(dba)_3$ (5 mol%), **L4** (20 mol%), acid (*S*)-**A1** (20 mol%) and 4 Å MS (100 mg) in toluene (1 mL) at 30 °C.

7. Asymmetric reaction on a 1.0 mmol scale

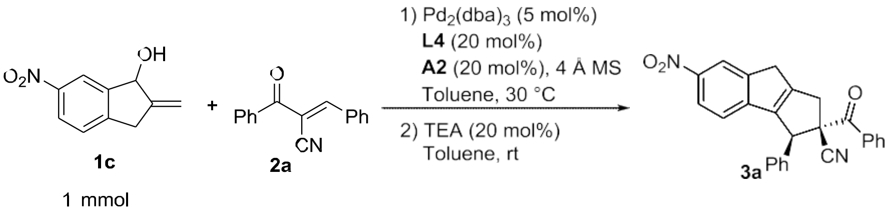

A flame-dried 10 mL Schlenk tube equipped with a magnetic stirring bar was cooled to room temperature. To this flask were added $Pd_2(dba)_3$ (45.8 mg, 0.0500 mmol, 0.05 equiv), **L4** (121.1 mg, 0.2001 mmol, 0.2 equiv), **A2** (50.0 mg, 0.200 mmol, 0.2 equiv) and 4 Å MS (1.0 g), and the flask evacuated and back-filled three times with argon. Then toluene (5 mL) was added by syringe. The mixture was stirred at 30 °C for 10 min. To another dry tube equipped with a magnetic stirring bar were added 2-methylene-6-nitro-2,3-dihydro-1*H*-inden-1-ol **1c** (191.1 mg, 1.000 mmol, 1.0 equiv) and (*E*)-2-benzoyl-3-phenylacrylonitrile **2a** (279.7 mg, 1.200 mmol, 1.2 equiv), evacuated and back-filled three times with argon. Then toluene (5 mL) was added by syringe. The mixture was stirred at rt for 5 min. Then the latter solution was added to the first Schlenk tube by syringe. The mixture was stirred at 30 °C for 12 h. After completion, Et_3N (28.0 μL, 0.202 mmol, 0.2 equiv) was added to the reaction mixture and stirred at rt for 2 h. After completion, the solvent was evaporated in vacuo. The residue was purified by flash chromatography on silica gel (EtOAc/petroleum ether = 1/8) to give the product **3a**: 370.7 mg, 91% yield, as a yellow solid; >19:1 dr; 88% ee.

8. Crystal data and structural refinement for enantiopure 3a

Preparation of the single crystals of enantiopure **3a**: 35.0 mg of compound **3a** (91% ee) was dissolved in DCM (1.0 mL) in a 10 mL tube, and *n*-hexane (3.0 mL) was added. The tube was sealed by a piece of weighing paper with several tiny holes, thus allowing slow evaporation of the solvents at room temperature. After 4 days, several small particles could be observed at the bottom of the tube. The crystals were chosen and subjected to the single crystal X-ray diffraction analysis for the determination of the absolute configuration of **3a**. The data were collected by an Agilent Gemini equipped with a Cu radiation source (Kα = 1.54184 Å) at 296.0(9) K. CCDC 2174555 (**3a**) contains the supplementary crystallographic data for this paper. These data can be obtained free of charge via www.ccdc.cam.ac.uk/data_request/cif.

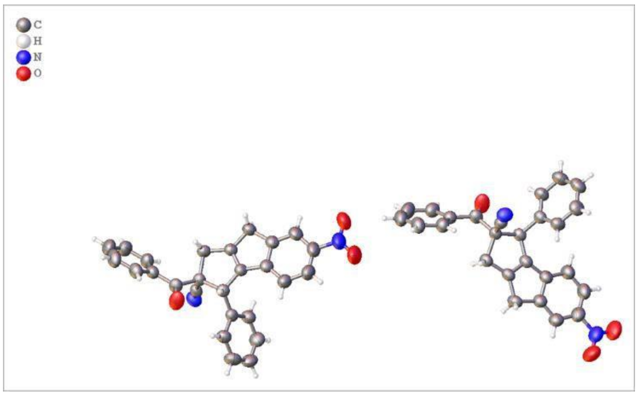

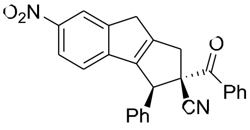

3a (CCDC 2174555)

(ellipsoid contour probability 50%)

Identification code	**3a**
Empirical formula	$C_{26}H_{18}N_2O_3$
Formula weight	406.42
Temperature/K	296.0(9)
Crystal system	monoclinic
Space group	$P2_1$
a/Å	6.34570(7)
b/Å	12.49324(16)
c/Å	29.7405(4)
α/°	90
β/°	93.4861(11)
γ/°	90
Volume/Å^3	2353.41(5)
Z	4
ρ_{calc}g/cm^3	1.147
μ/mm^{-1}	0.613
F(000)	848.0
Crystal size/mm^3	0.45 × 0.35 × 0.3

Radiation	CuKα (λ = 1.54184)
2Θ range for data collection/°	7.678 to 142.826
Index ranges	-7 ≤ h ≤ 5, -15 ≤ k ≤ 15, -33 ≤ l ≤ 36
Reflections collected	25763
Independent reflections	9006 [R_{int} = 0.0406, R_{sigma} = 0.0314]
Data/restraints/parameters	9006/1/559
Goodness-of-fit on F^2	1.037
Final R indexes [I>=2σ (I)]	R_1 = 0.0505, wR_2 = 0.1373
Final R indexes [all data]	R_1 = 0.0543, wR_2 = 0.1426
Largest diff. peak/hole / e $Å^{-3}$	0.15/-0.24
Flack parameter	-0.01(14)

9. Mechanism study

9.1 Control experiments

a) The formation of 3a with 5-ntiro 1e as the substrate

When 5-nitro **1e** was utilised as the substrate, **3a** was isolated in a moderate yield after treatment with Et3N. The results indicated an isomerisation process of the 10π-intermediate would be involved. Under the catalysis of Pd/**A2**, **1e** underwent an oxidative addition/deprotonation to give intermediate **I′**, which would isomerise to the more stable intermediate **I** to afford **3a** followed by [10+2] cycloaddition with **2a** and isomerisation.

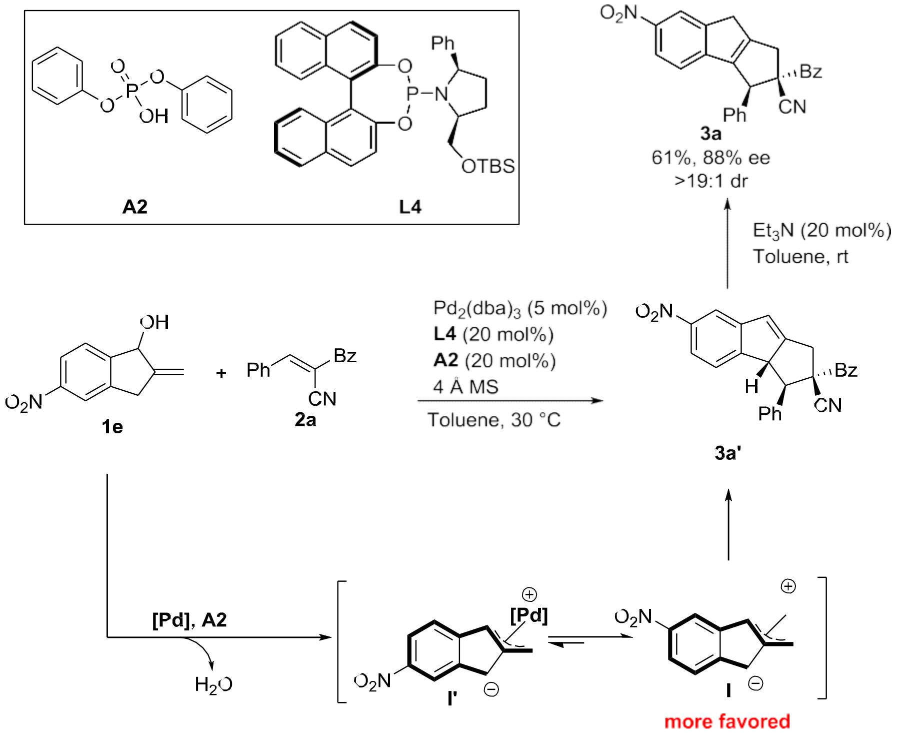

b) With acyclic alcohol 13 as the substrate

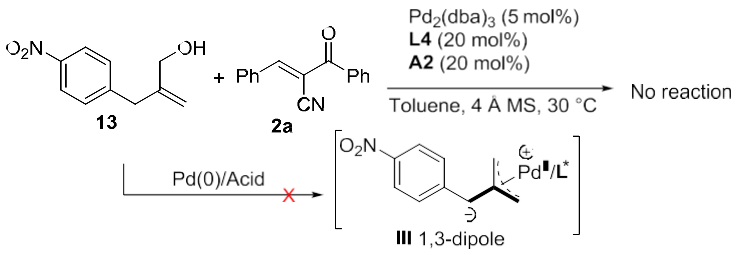

9.2 The formation of 3s with 1d as the substrate

When **1d** with a 4-nitro group was applied to the reaction with **2a** under the standard conditions, **3s** (confirmed by ^{1}H NMR and ^{13}C NMR) was obtained in a quantitative yield with good enantioselectivity, and no isomerisation phenomenon for the zwitterionic intermediate was observed. It was envisaged that **1d** underwent oxidative addition and deprotonation reaction, and resultant intermediate **V** was formed. The anion at the C-3 position could be stabilised by the *ortho*-NO_2 group; in contrast, intermediate **VI** would be relatively less stable. Indeed, cycloadduct **3s″** has not been detected, indicating that the isomerisation process was not favored. Instead, **V** participated in [10+2] cycloaddition with **2a** to give **3s′**, and then **3s** was finally afforded after treatment with Et_3N.

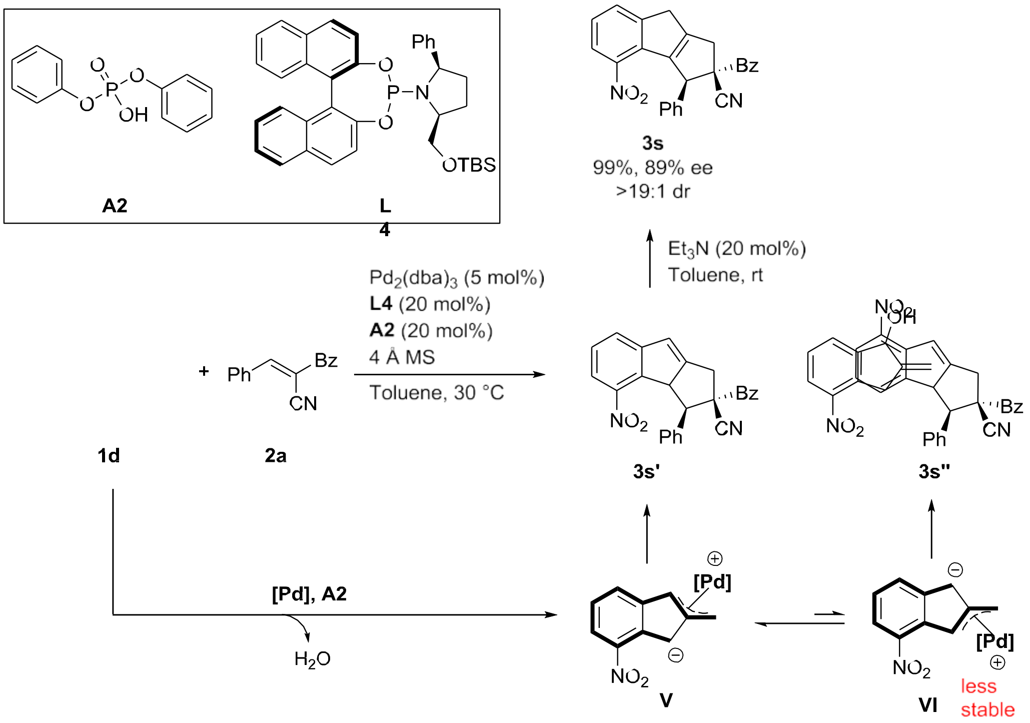

9.3 Proposed catalytic cycle

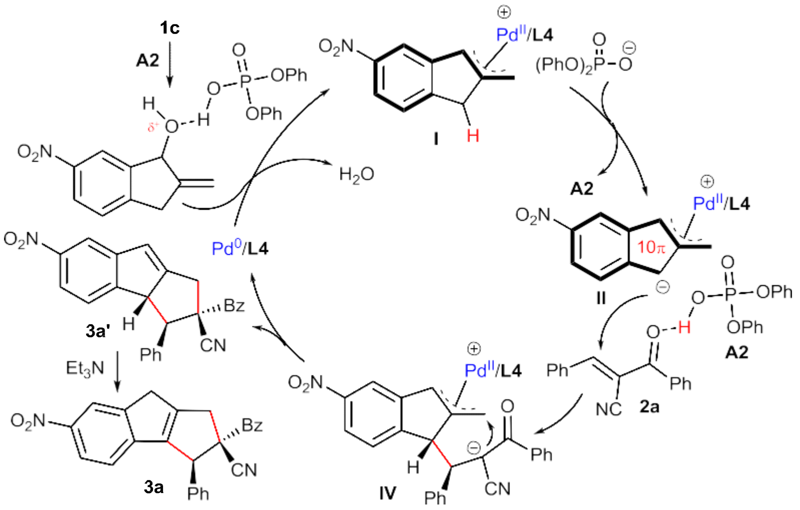

10. References

1. P. Zhao, Z. Li, J. He, X. Liu and X. Feng, *Sci. China Chem*., 2021, **64**, 1355.
2. Y. Yang, Y. Jiang, W. Du and Y.-C. Chen, *Chem.* **-** *Eur. J*., 2020, **26**, 1754.
3. K.-K. Wang, P. Wang, Q. Ouyang, W. Du and Y.-C. Chen, *Chem. Commun*., 2016, **52**, 11104.
4. E. Fillion, A. Kavoosi, K. Nguyen and C. Ieritano, *Chem. Commun.*, 2016, **52**, 12813.
5. T. Drennhaus, L. Öhler, S. Djalali, S. Höfmann, C. Müller, J. Pietruszka and D. Worgull, *Adv. Synth. Catal*., 2020, **362**, 2385.
6. Z.-Z. Gao, C. Wang, L.-J. Zhou, C.-H. Yuan, Y.-M. Xiao and H.-C. Guo**,** *Org. Lett.*, 2018, **20**, 4302.
7. T. B. Nguyen and P. Retailleau, *Org. Lett*., 2018, **20**, 186.
8. A. Turočkin, W. Raven and P. Selig, *Eur. J. Org. Chem*., 2017, 296.

11. NMR, HRMS spectra and HPLC chromatograms

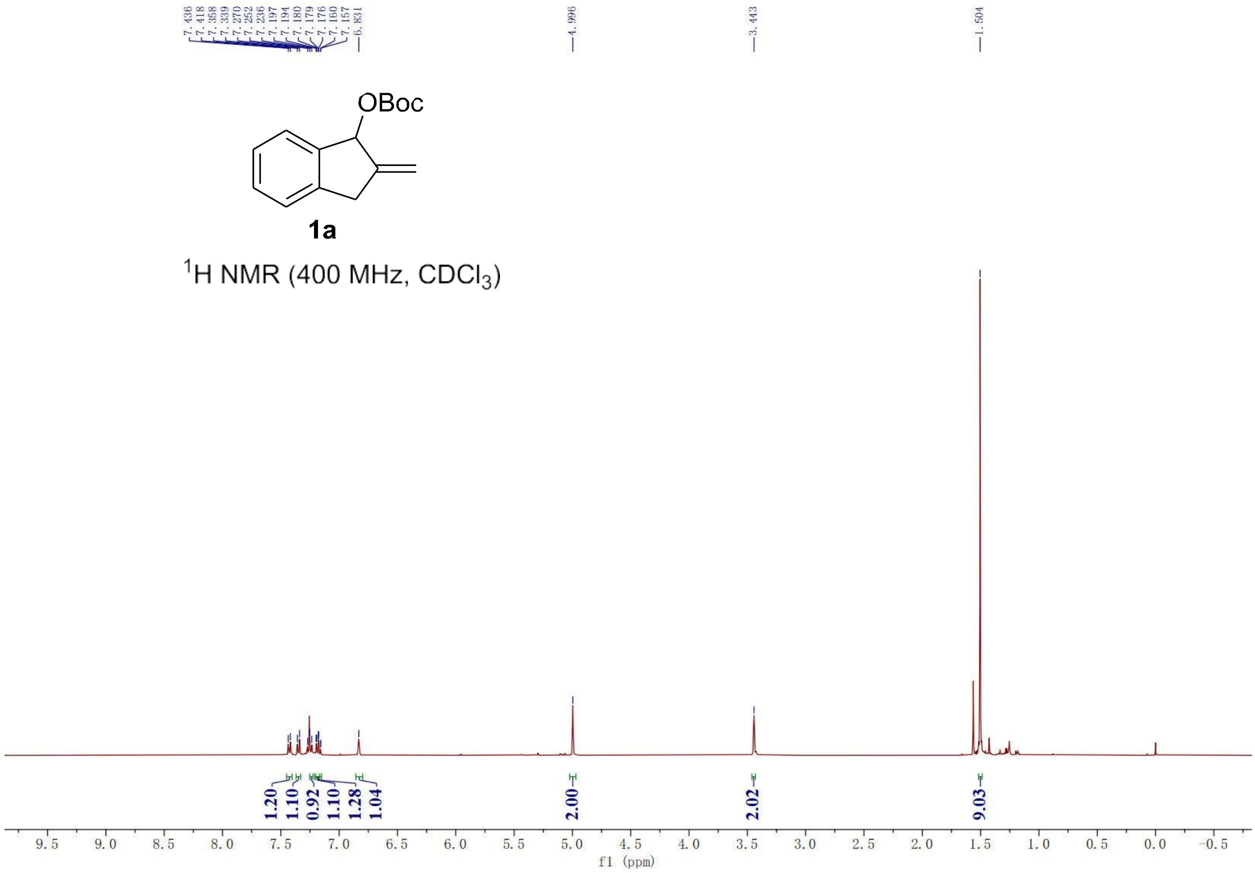

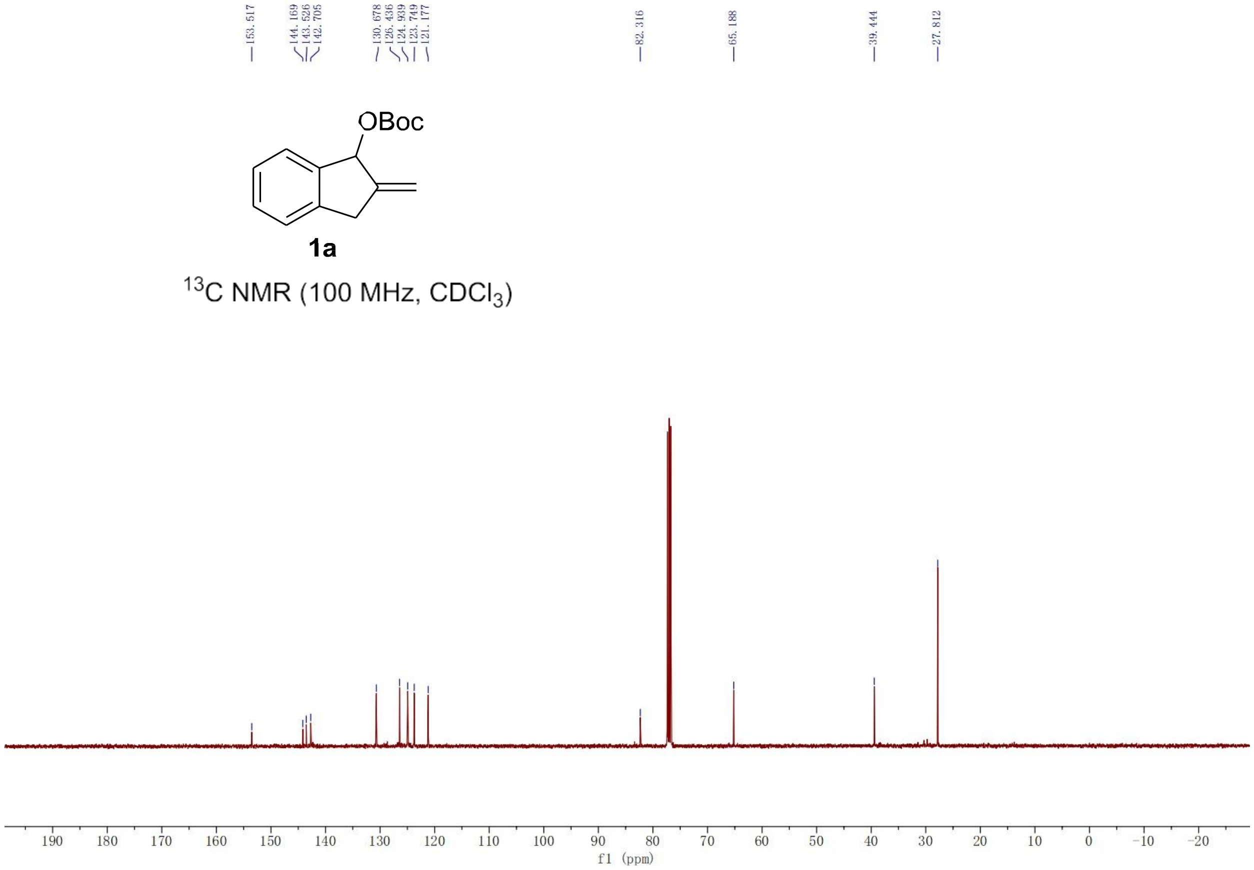

User Spectrum Plot Report

Name	CYC-220516-1	Rack Pos.		Instrument	Instrument 1	Operator	
Inj. Vol. (ul)	8	Plate Pos.		IRM Status	All ions missed		
Data File	CYC-220516-1.d	Method (Acq)	CJH220516.m	Comment		Acq. Time (Local)	5/20/2022 3:19:41 PM (UTC+08:00)

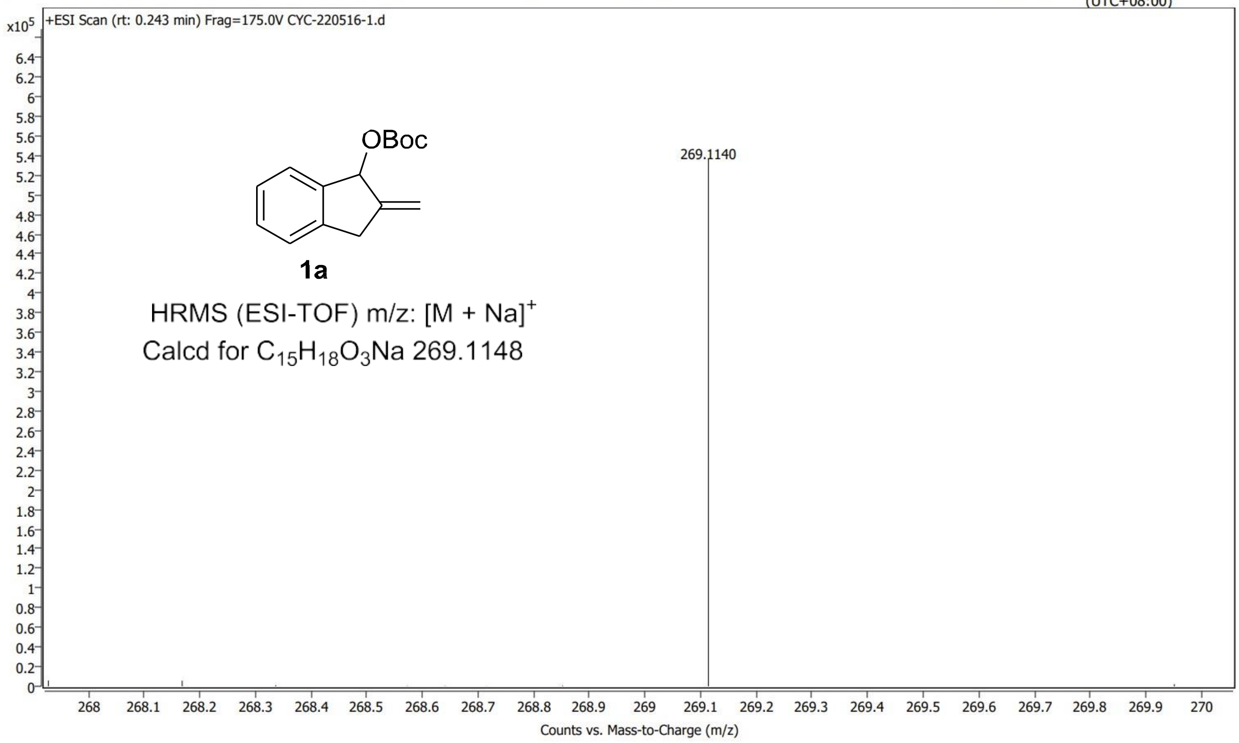

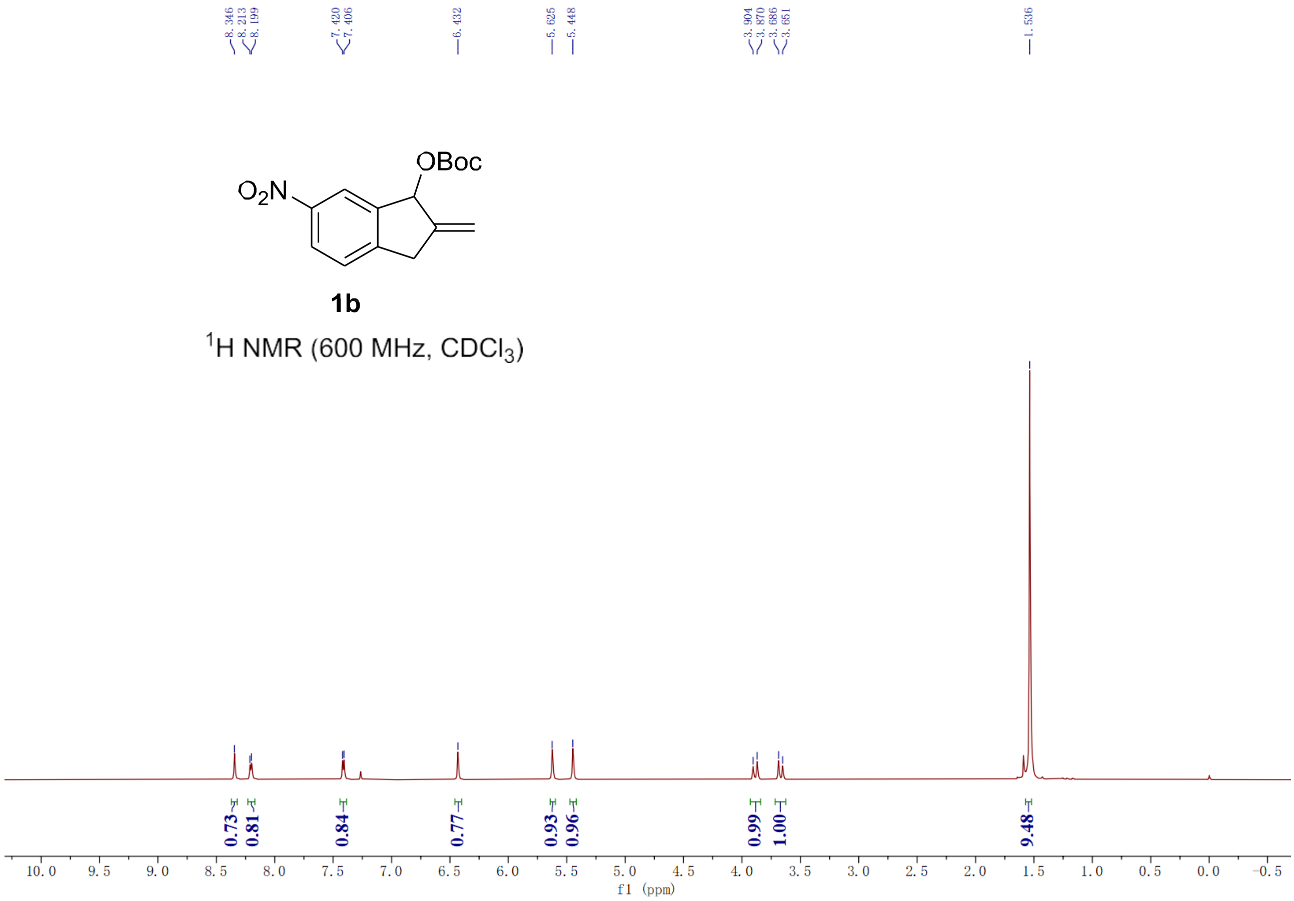

1b

^{1}H NMR (600 MHz, $CDCl_3$)

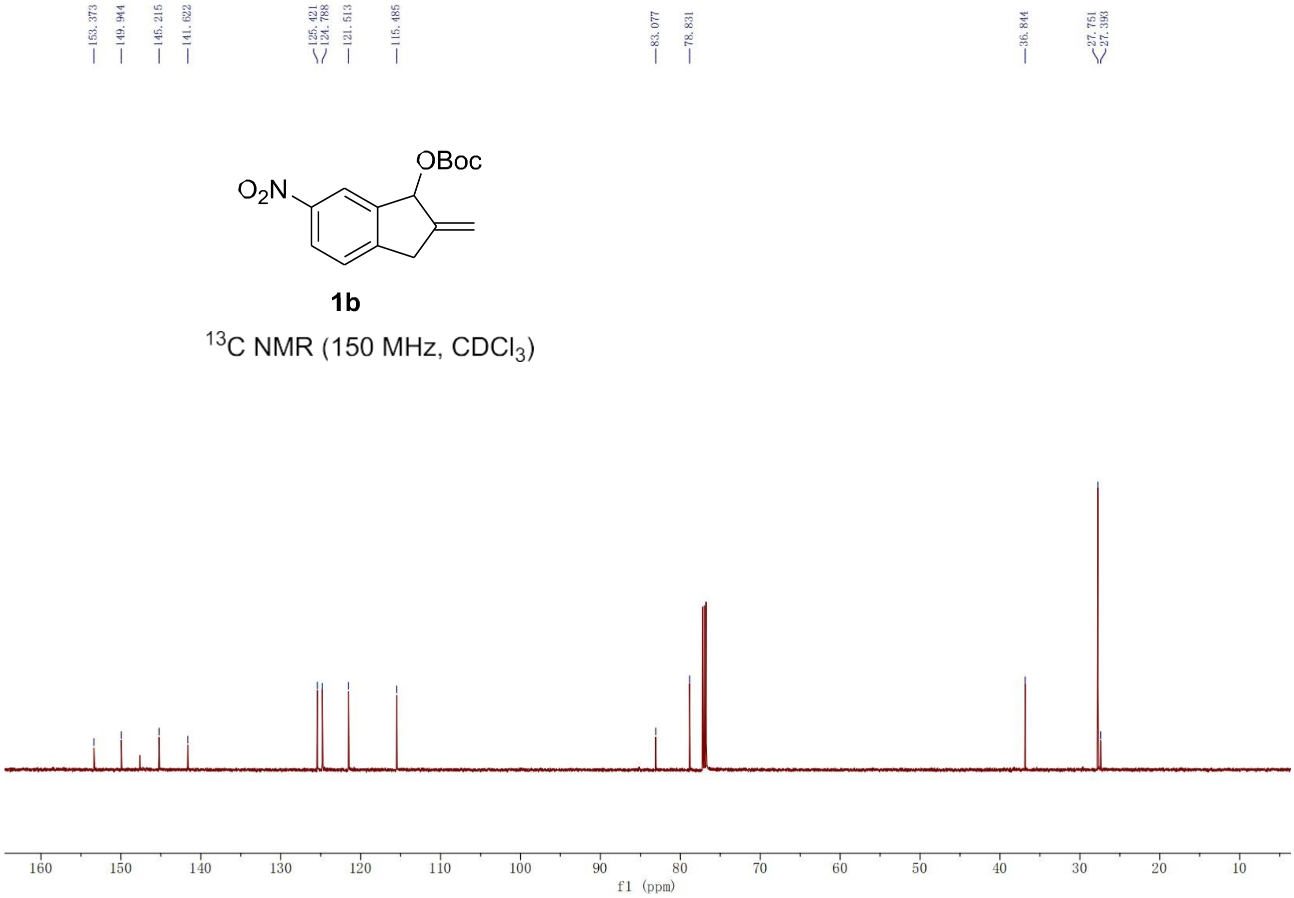

1b

^{13}C NMR (150 MHz, $CDCl_3$)

User Spectrum Plot Report

Agilent Trusted Answers

Name	CYC-220516-3	Rack Pos.		Instrument	Instrument 1	Operator	
Inj. Vol. (ul)	8	Plate Pos.		IRM Status	All ions missed		
Data File	CYC-220516-3.d	Method (Acq)	CJH220516.m	Comment		Acq. Time (Local)	5/20/2022 3:25:35 PM (UTC+08:00)

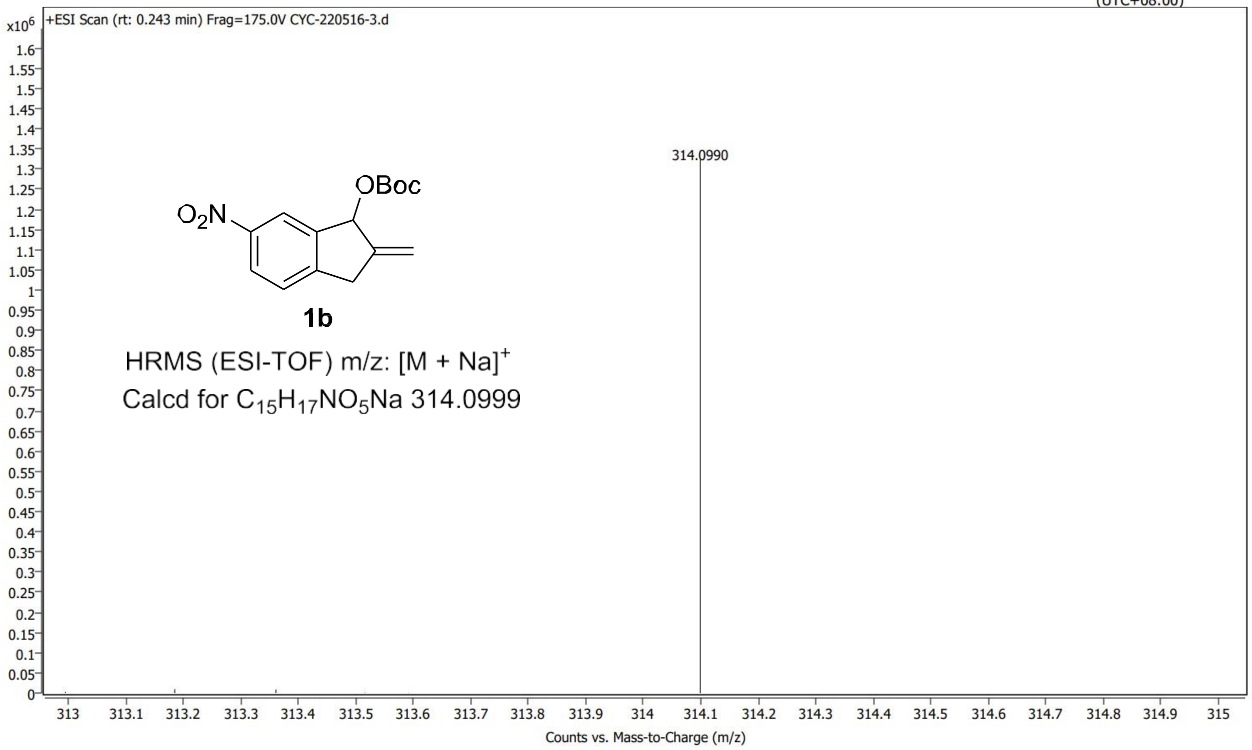

1b

HRMS (ESI-TOF) m/z: [M + Na]$^+$
Calcd for $C_{15}H_{17}NO_5Na$ 314.0999

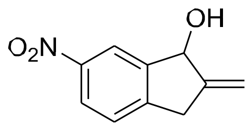

1c

^1H NMR (400 MHz, $CDCl_3$)

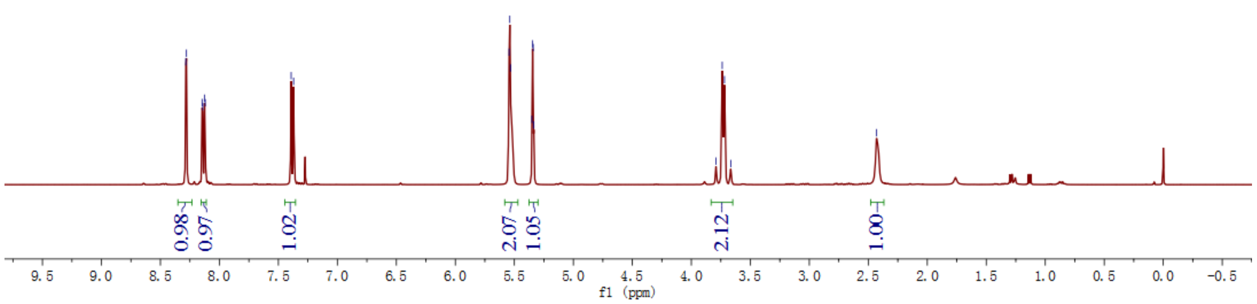

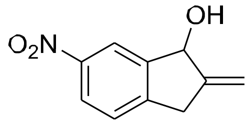

1c

^{13}C NMR (100 MHz, $CDCl_3$)

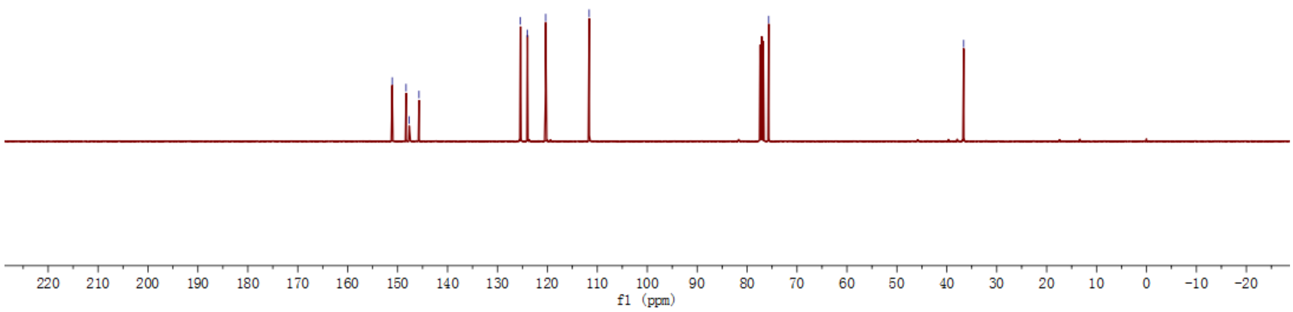

Spectrum Plot Report

Name	CYC-20201231-16	Rack Pos.		Instrument	Instrument 1	Operator	
Inj. Vol. (ul)	8	Plate Pos.		IRM Status	Success		
Data File	CYC-20201231-16.d	Method (Acq)	ZYJ-20201106.m	Comment		Acq. Time (Local)	12/24/2020 6:30:34 PM (UTC+08:00)

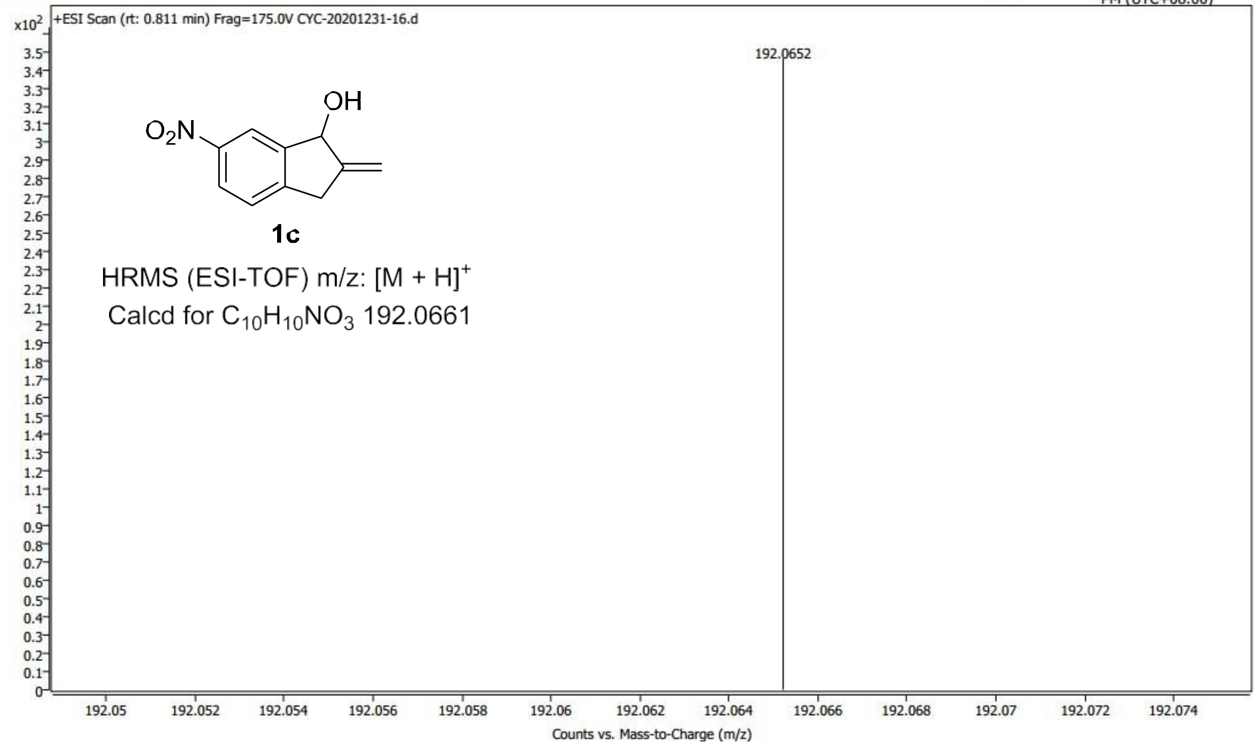

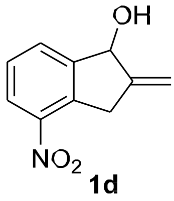

^{1}H NMR (400 MHz, $CDCl_3$)

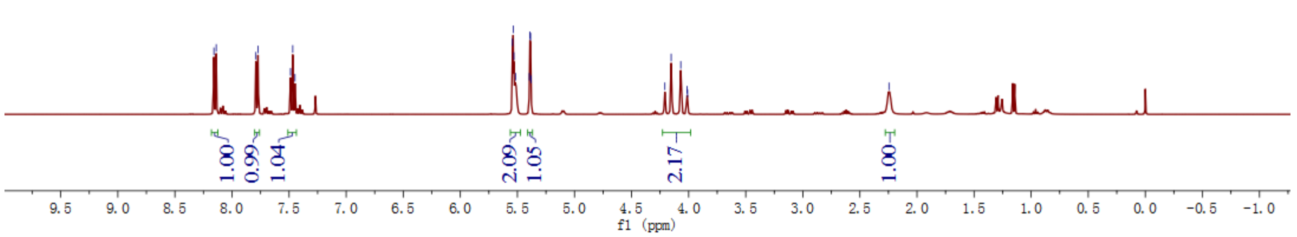

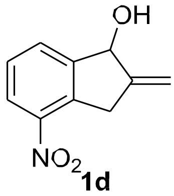

^{13}C NMR (100 MHz, $CDCl_3$)

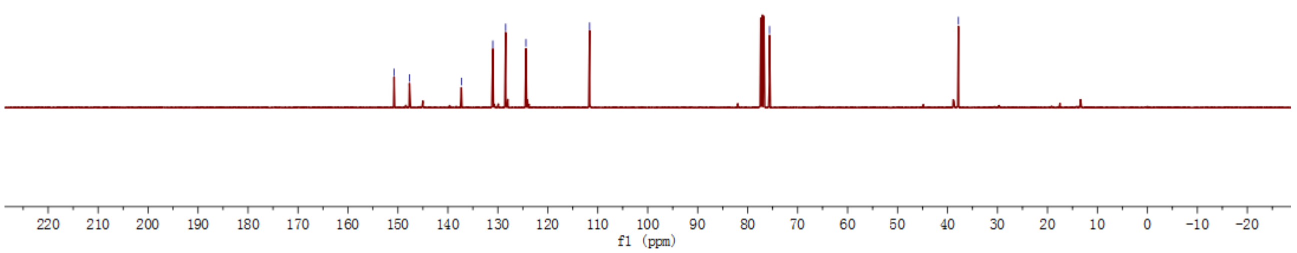

Spectrum Plot Report

Name	CYC-20201231-18	Rack Pos.		Instrument	Instrument 1	Operator	
Inj. Vol. (ul)	8	Plate Pos.		IRM Status	Success		
Data File	CYC-20201231-18.d	Method (Acq)	ZYJ-20201106.m	Comment		Acq. Time (Local)	12/24/2020 6:36:32 PM (UTC+08:00)

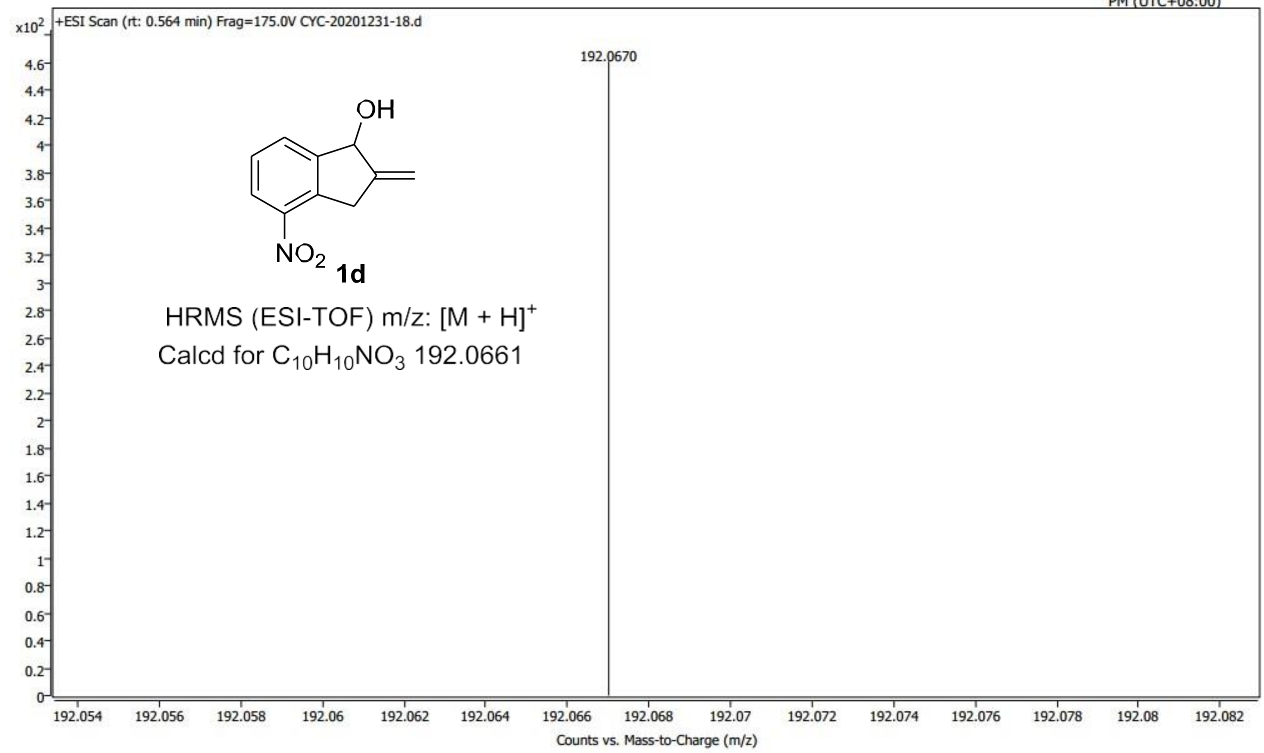

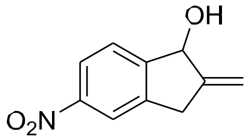

1e

^{1}H NMR (400 MHz, $CDCl_3$)

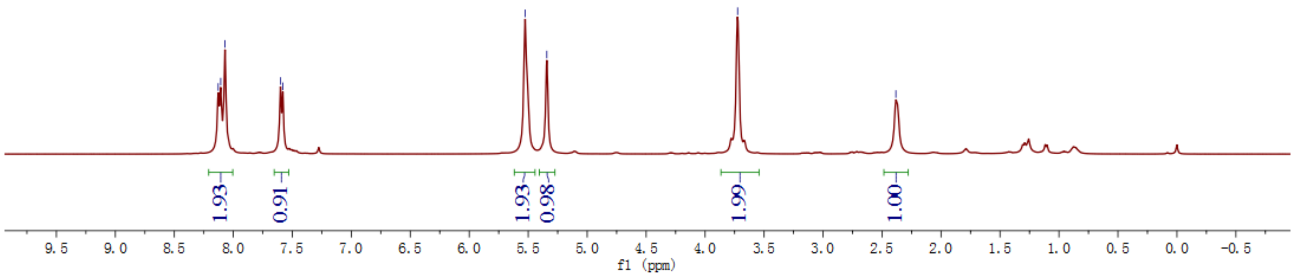

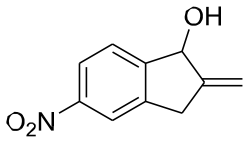

1e

^{13}C NMR (100 MHz, $CDCl_3$)

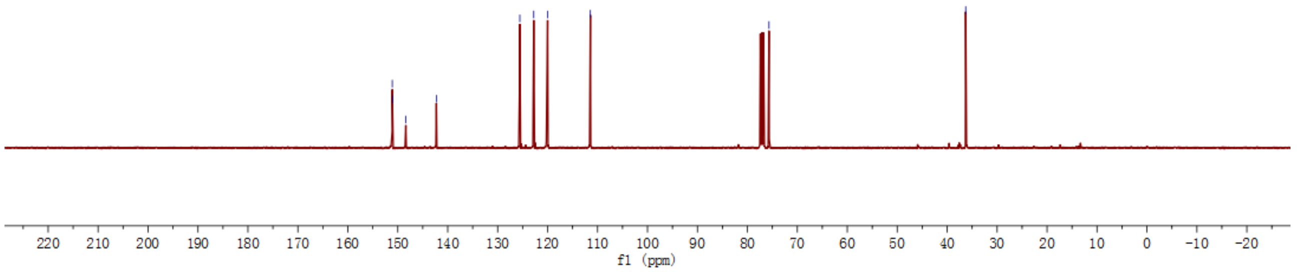

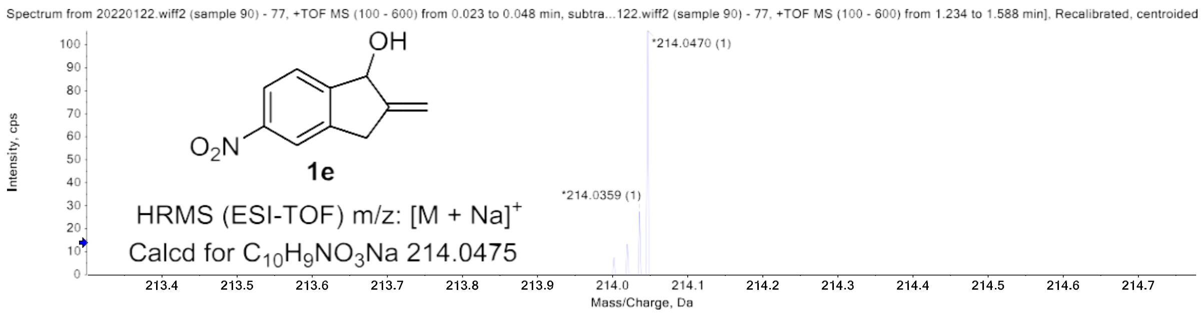
Spectrum from 20220122.wiff2 (sample 90) - 77, +TOF MS (100 - 600) from 0.023 to 0.048 min, subtra...122.wiff2 (sample 90) - 77, +TOF MS (100 - 600) from 1.234 to 1.588 min], Recalibrated, centroided
OH
O2N
1e
HRMS (ESI-TOF) m/z: [M + Na]+
Calcd for C10H9NO3Na 214.0475
*214.0470 (1)
*214.0359 (1)
Intensity, cps
Mass/Charge, Da

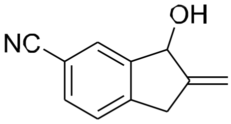

1f

^{1}H NMR (400 MHz, $CDCl_3$)

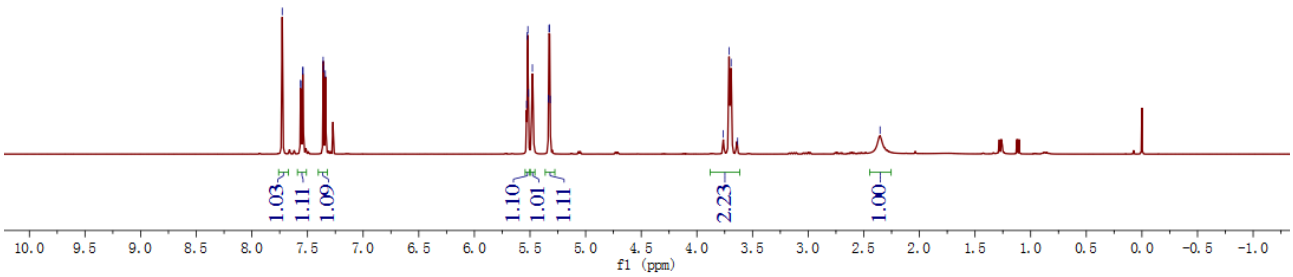

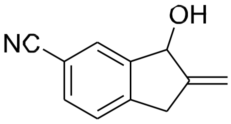

1f

^{13}C NMR (100 MHz, $CDCl_3$)

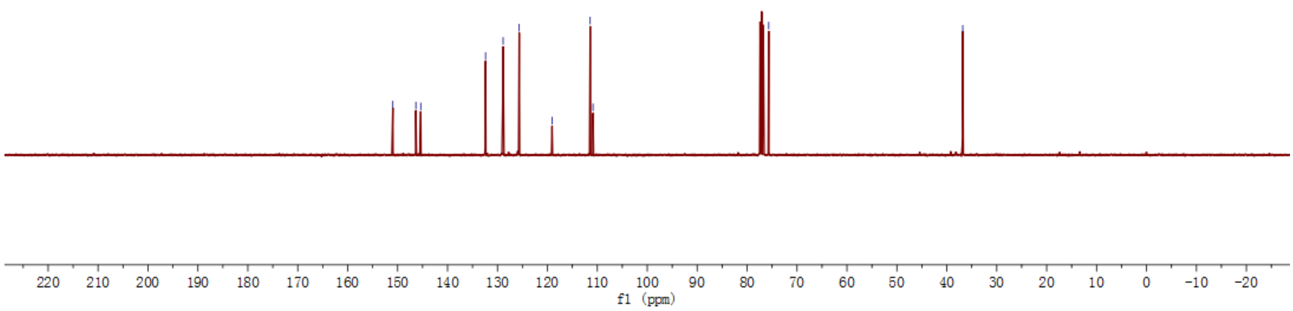

Spectrum Plot Report

Name	CYC-20201231-17	Rack Pos.		Instrument	Instrument 1	Operator	
Inj. Vol. (ul)	8	Plate Pos.		IRM Status	Success		
Data File	CYC-20201231-17.d	Method (Acq)	ZYJ-20201106.m	Comment		Acq. Time (Local)	12/24/2020 6:33:33 PM (UTC+08:00)

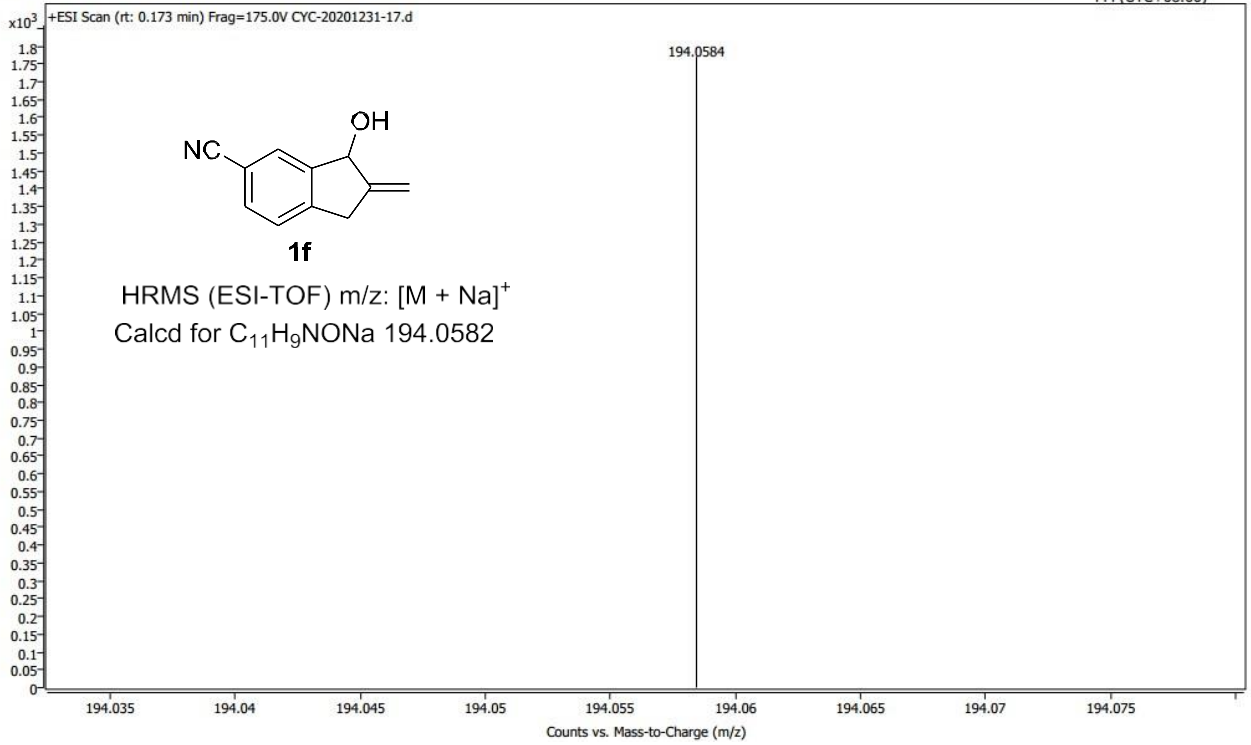

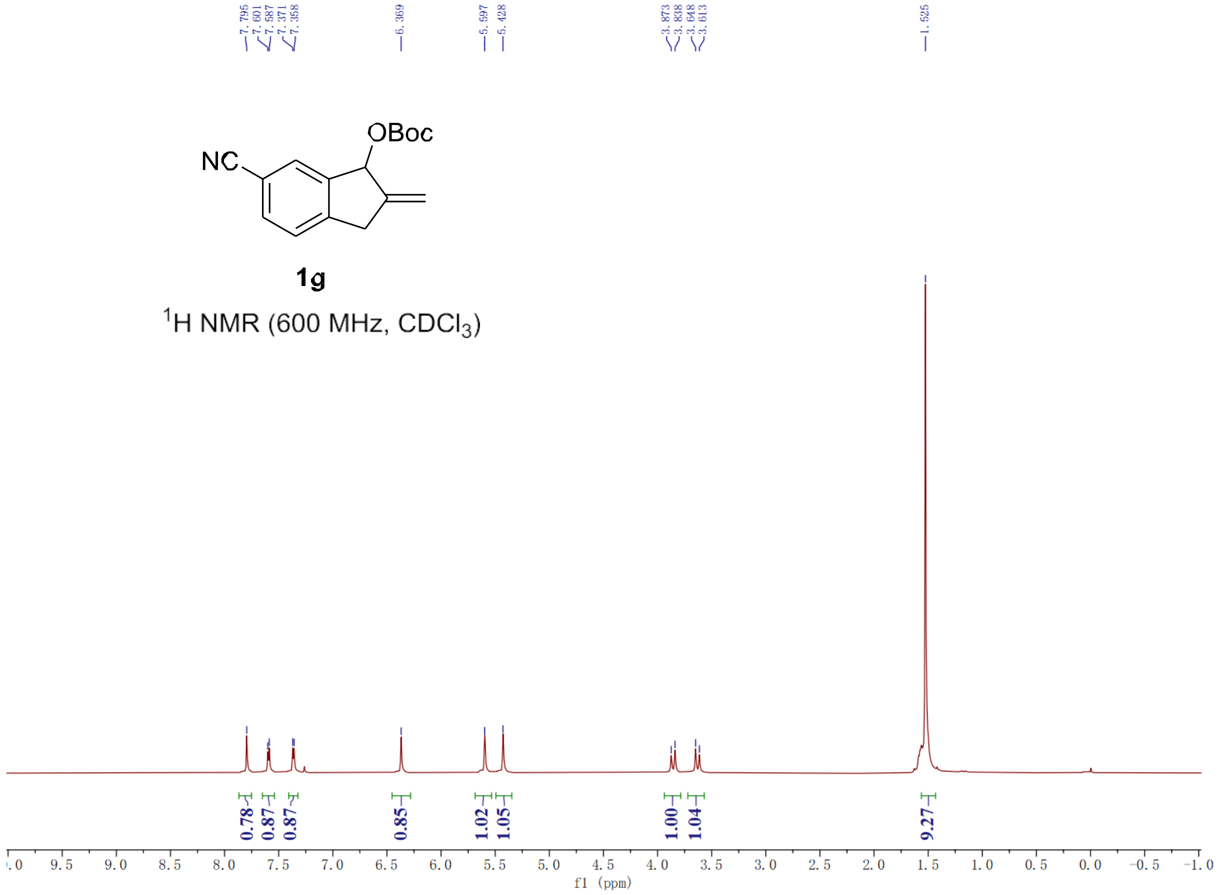
7.795
7.601
7.587
7.371
7.358
6.369
5.597
5.428
3.873
3.838
3.648
3.613
1.525
OBoc
NC
1g
1H NMR (600 MHz, CDCl3)
0.78
0.87
0.87
0.85
1.02
1.05
1.00
1.04
9.27
9.5 9.0 8.5 8.0 7.5 7.0 6.5 6.0 5.5 5.0 4.5 4.0 3.5 3.0 2.5 2.0 1.5 1.0 0.5 0.0 -0.5 -1.0
f1 (ppm)

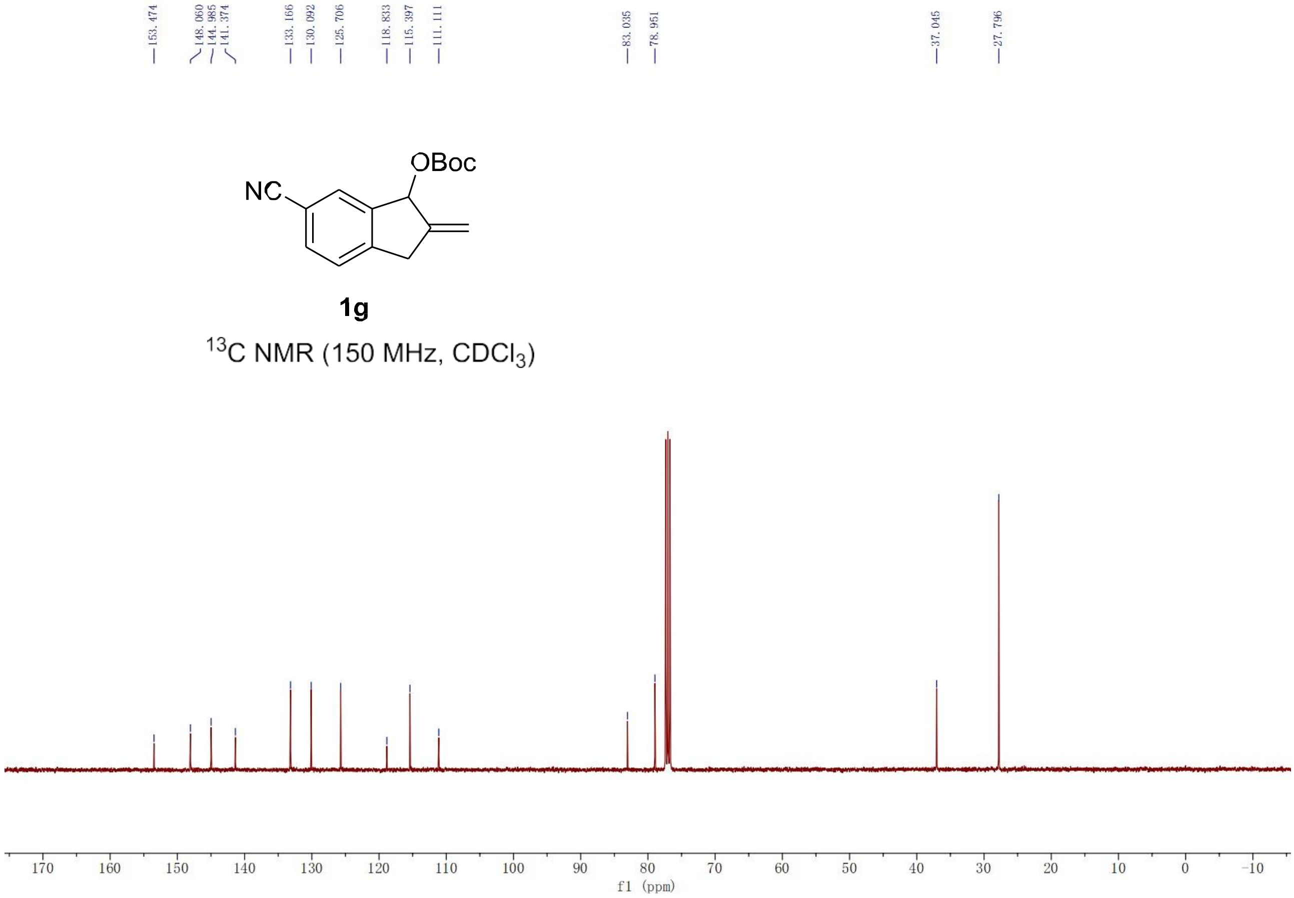
153.474
148.060
144.985
141.374
133.166
130.092
125.706
118.833
115.397
111.111
83.035
78.951
37.045
27.796
OBoc
NC
1g
13C NMR (150 MHz, CDCl3)
170 160 150 140 130 120 110 100 90 80 70 60 50 40 30 20 10 0 -10
f1 (ppm)

Spectrum Plot Report

Name	CYC-220425-27	Rack Pos.		Instrument	Instrument 1	Operator	
Inj. Vol. (ul)	8	Plate Pos.		IRM Status	Success		
Data File	CYC-220425-27.d	Method (Acq)	ZYJ-20201106.m	Comment		Acq. Time (Local)	4/29/2022 11:34:14 AM (UTC+08:00)

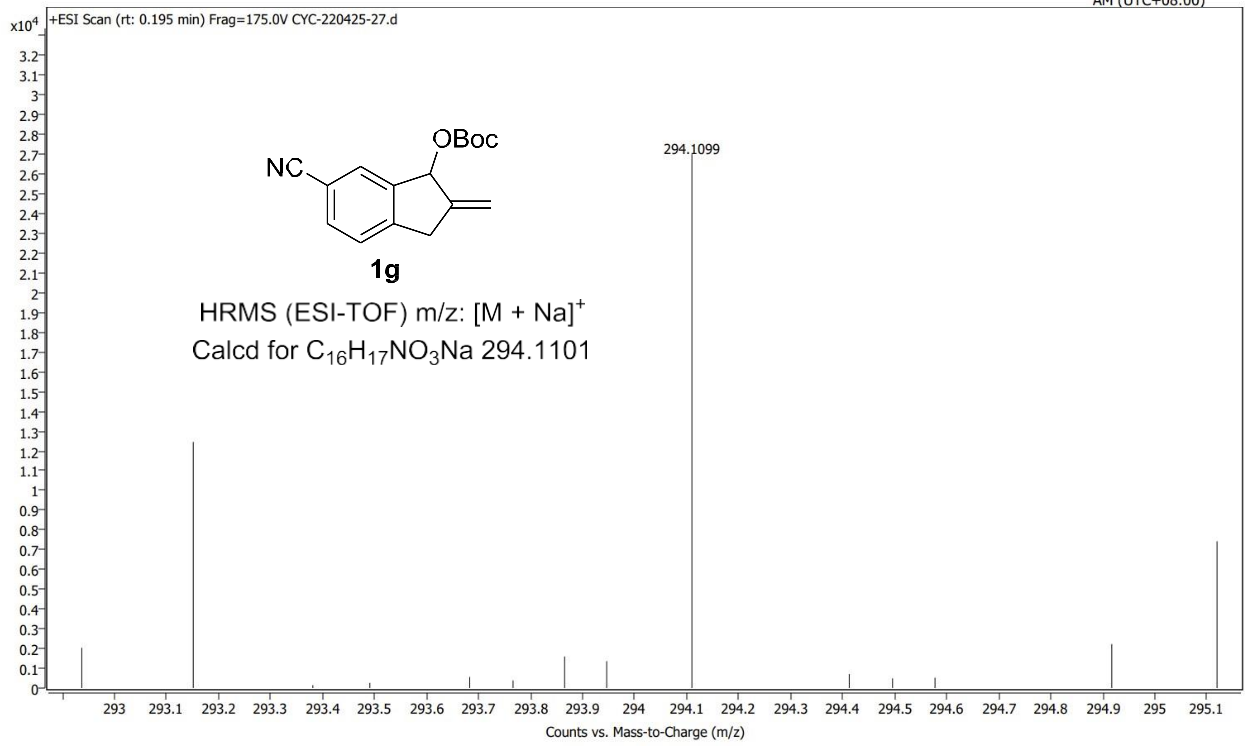

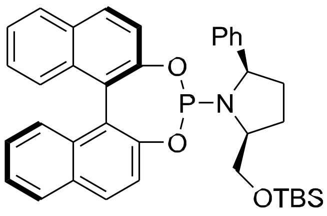

L4

^{1}H NMR (400 MHz, $CDCl_3$)

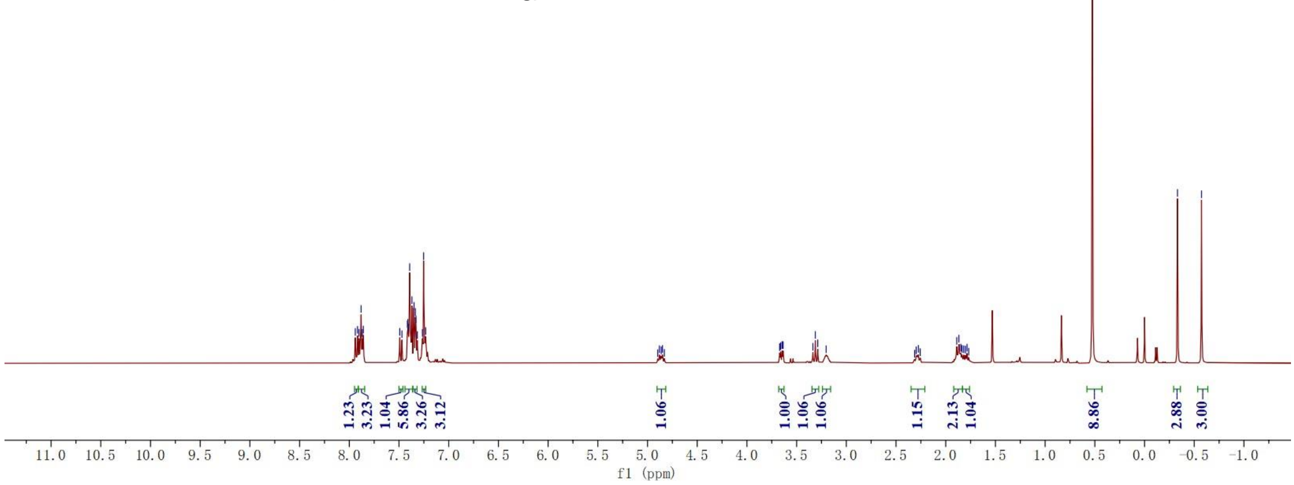

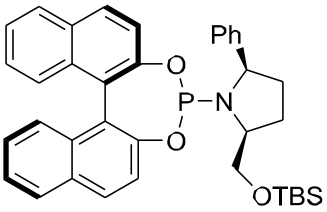

L4

^{13}C NMR (100 MHz, $CDCl_3$)

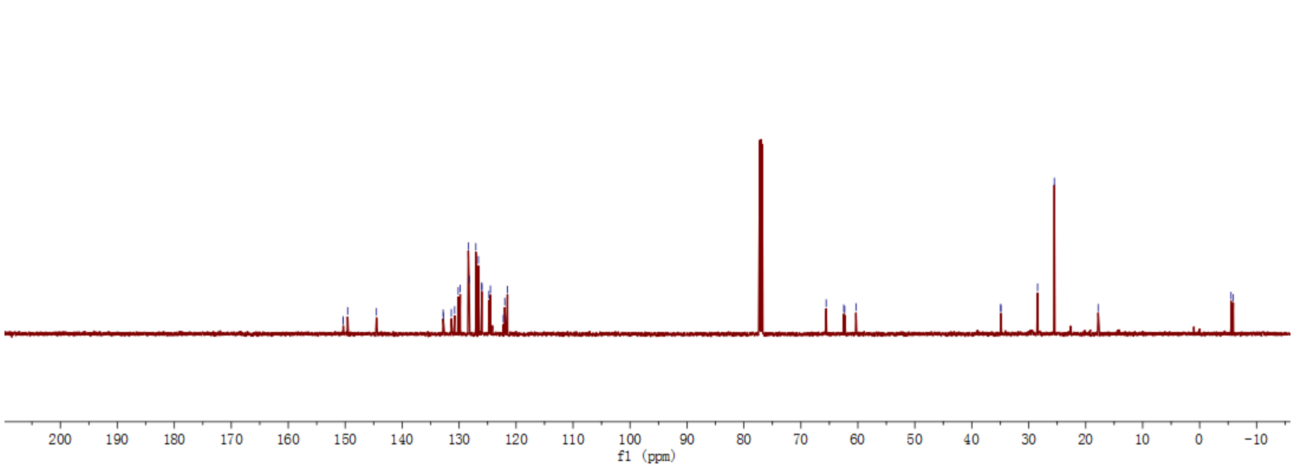

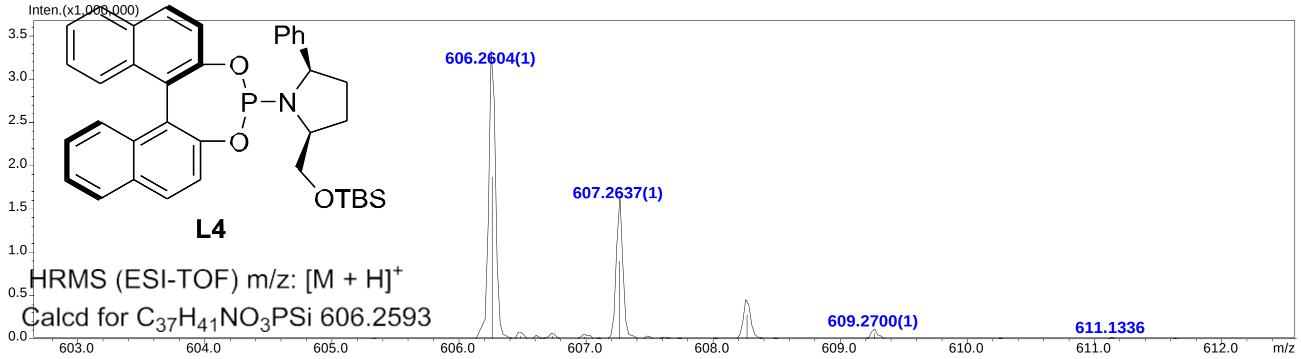

Inten.(x1,000,000)
3.5
3.0
2.5
2.0
1.5
1.0
0.5
0.0
Ph
O
P
N
O
OTBS
L4
HRMS (ESI-TOF) m/z: [M + H]+
Calcd for C37H41NO3PSi 606.2593
606.2604(1)
607.2637(1)
609.2700(1)
611.1336
603.0
604.0
605.0
606.0
607.0
608.0
609.0
610.0
611.0
612.0
m/z

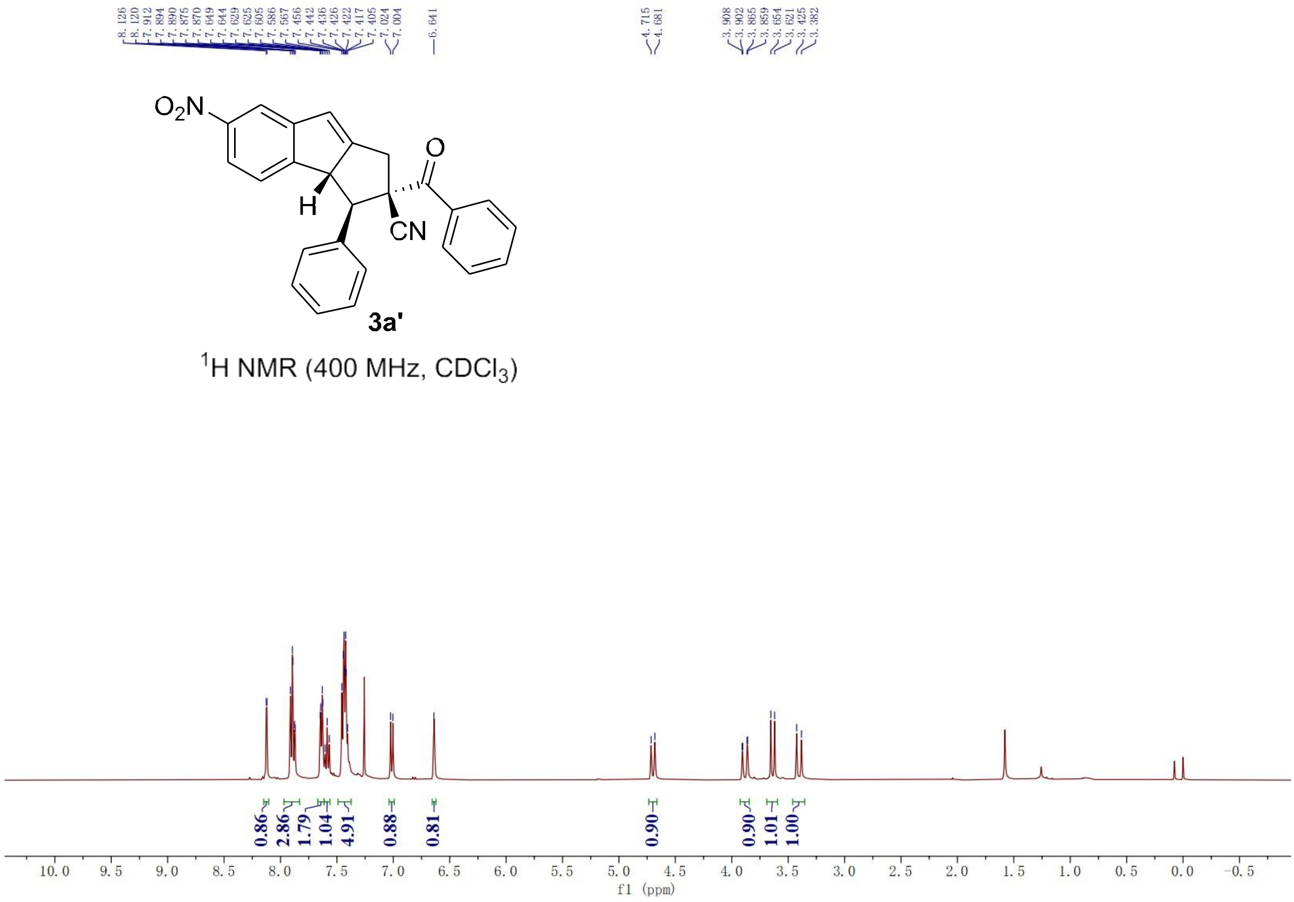

^{1}H NMR (400 MHz, $CDCl_3$)

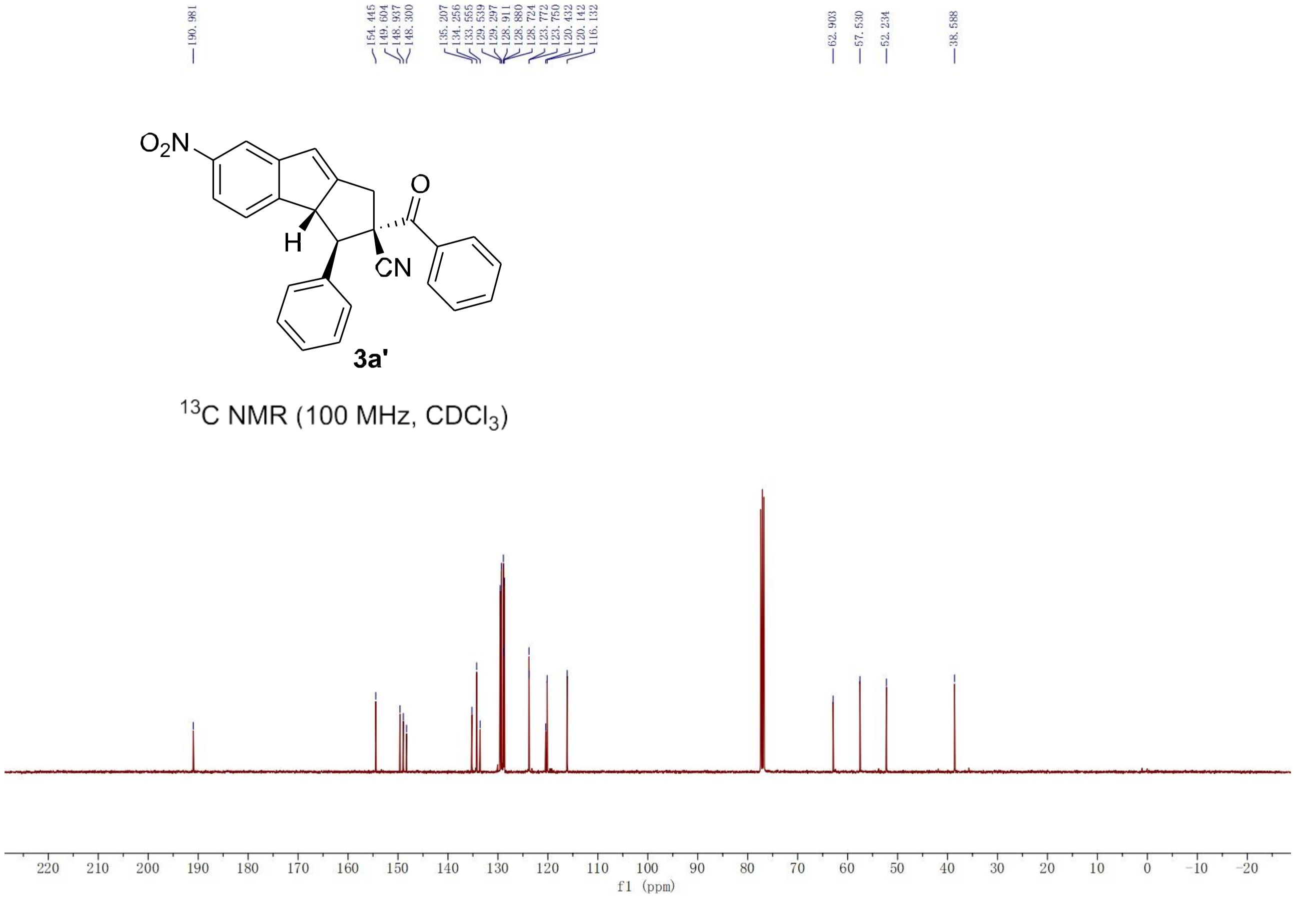

^{13}C NMR (100 MHz, $CDCl_3$)

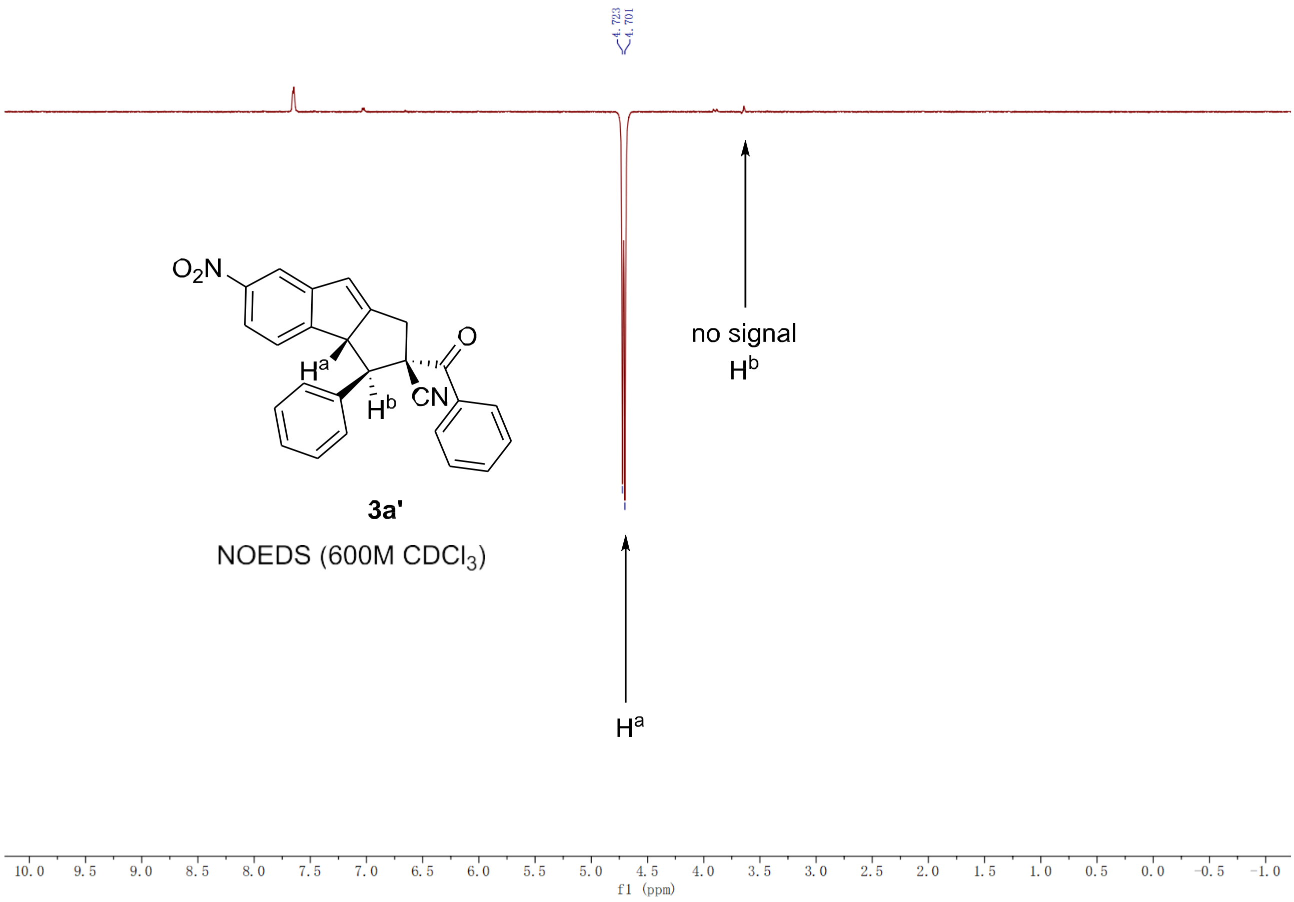
4.723
4.701
no signal
H^b
H^a
O2N
O
CN
3a'
NOEDS (600M CDCl3)
10.0 9.5 9.0 8.5 8.0 7.5 7.0 6.5 6.0 5.5 5.0 4.5 4.0 3.5 3.0 2.5 2.0 1.5 1.0 0.5 0.0 -0.5 -1.0
f1 (ppm)

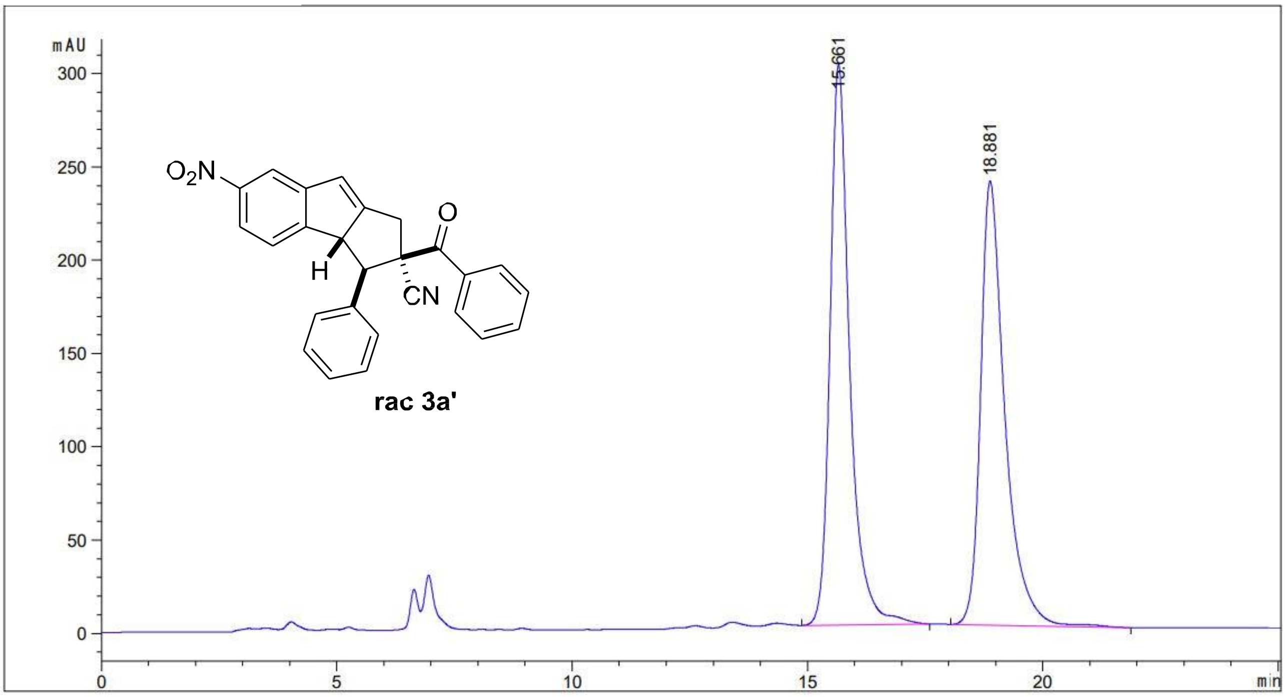

#	[min]		[min]	[mAU*s]	[mAU]	%
1	15.661	BB	0.4346	8752.58008	300.37457	50.5949
2	18.881	BB	0.5311	8546.74219	238.00890	49.4051

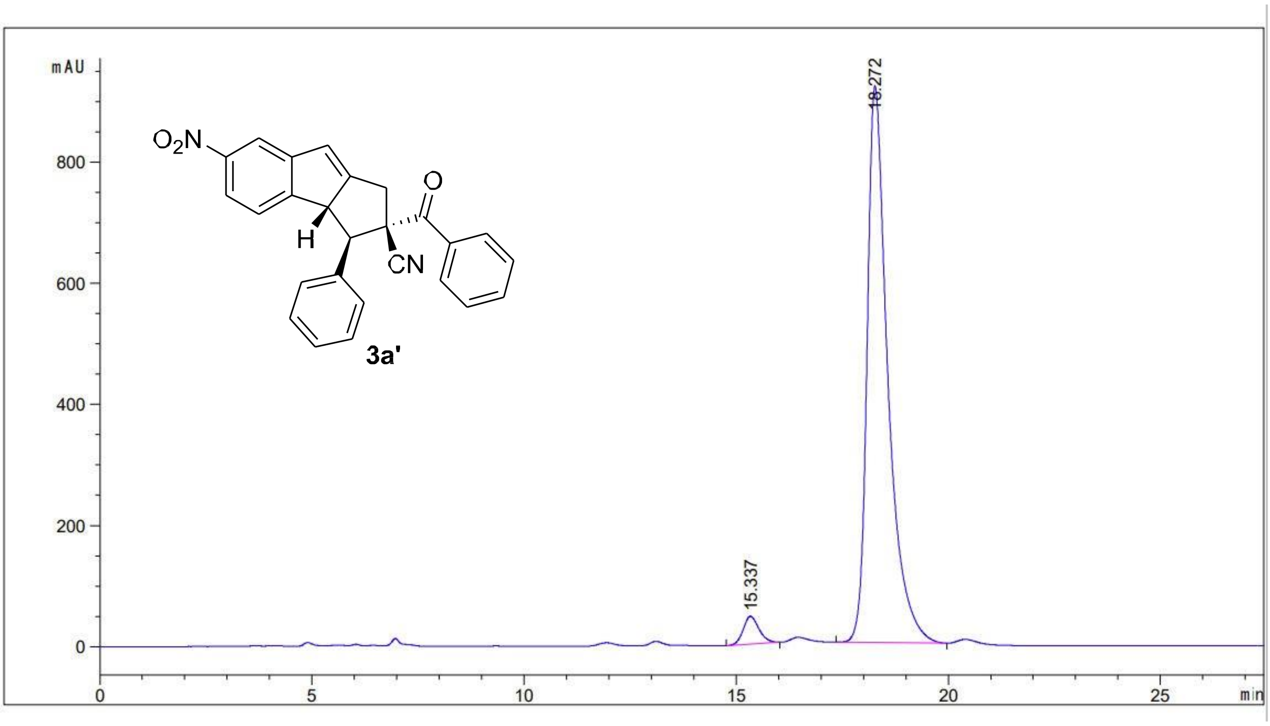

#	[min]		[min]	[mAU*s]	[mAU]	%
1	15.337	BB	0.3931	1189.36987	46.04202	3.6688
2	18.272	BB	0.5013	3.12292e4	918.93475	96.3312

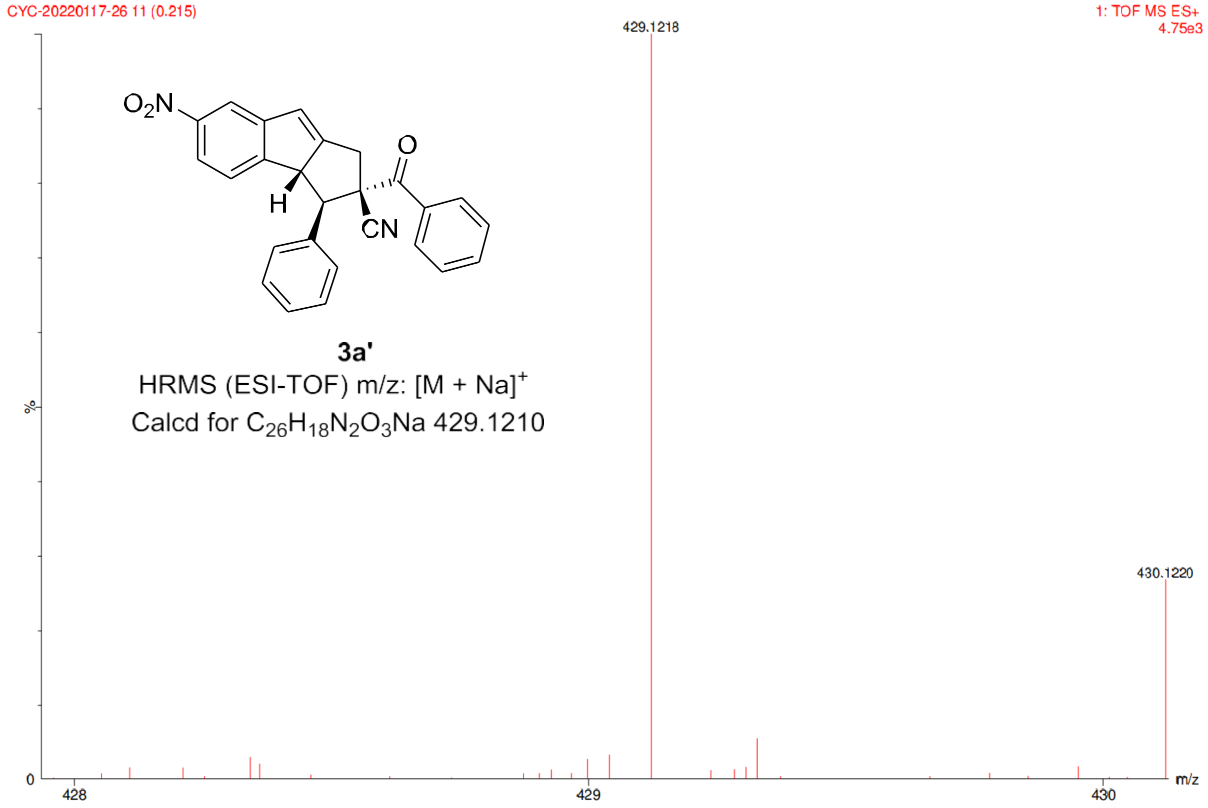
CYC-20220117-26 11 (0.215)
1: TOF MS ES+
4.75e3
429.1218
430.1220
O2N
H
O
CN
3a'
HRMS (ESI-TOF) m/z: [M + Na]+
Calcd for C26H18N2O3Na 429.1210
%
0
428
429
430
m/z

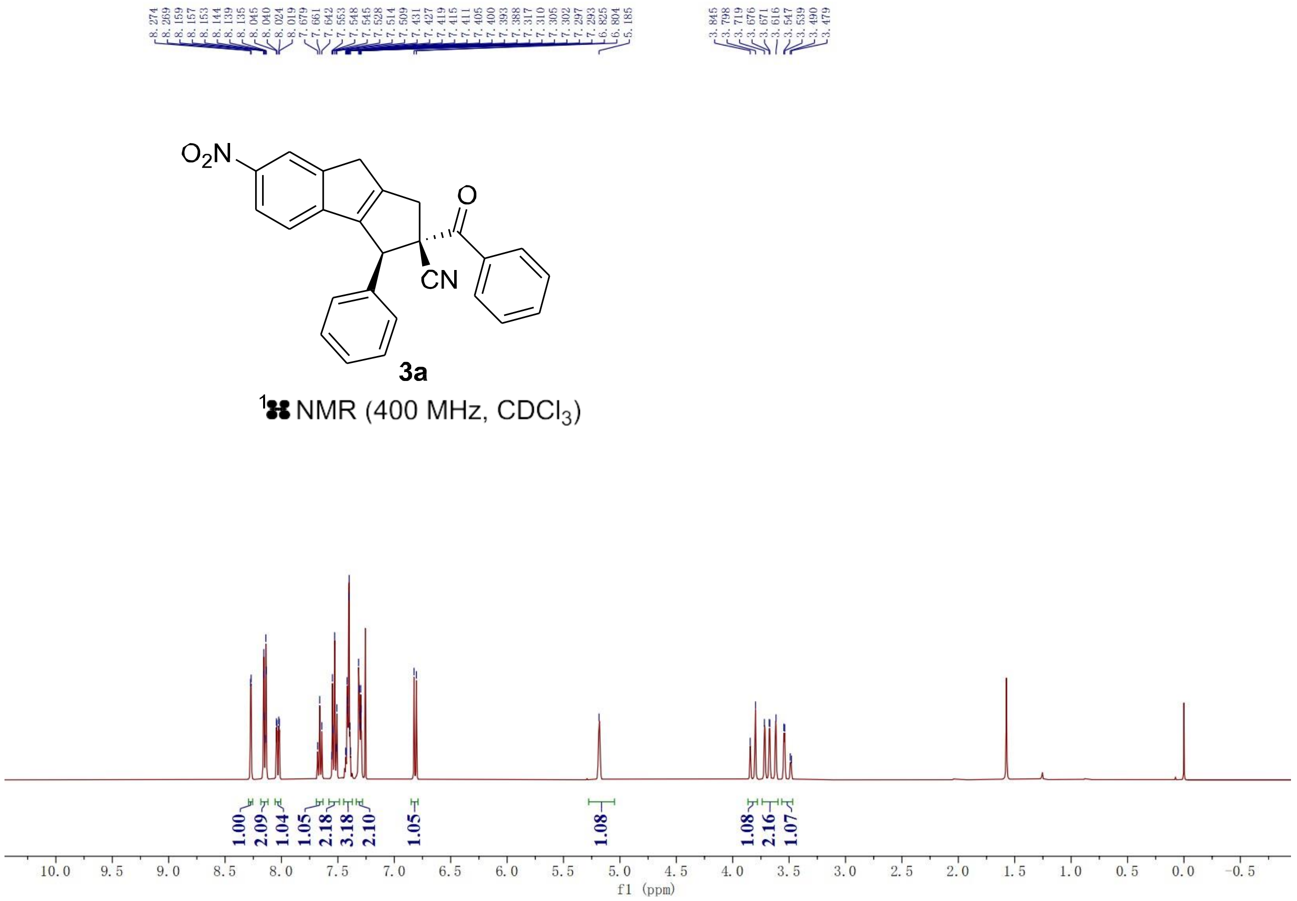

3a

^{1}H NMR (400 MHz, $CDCl_3$)

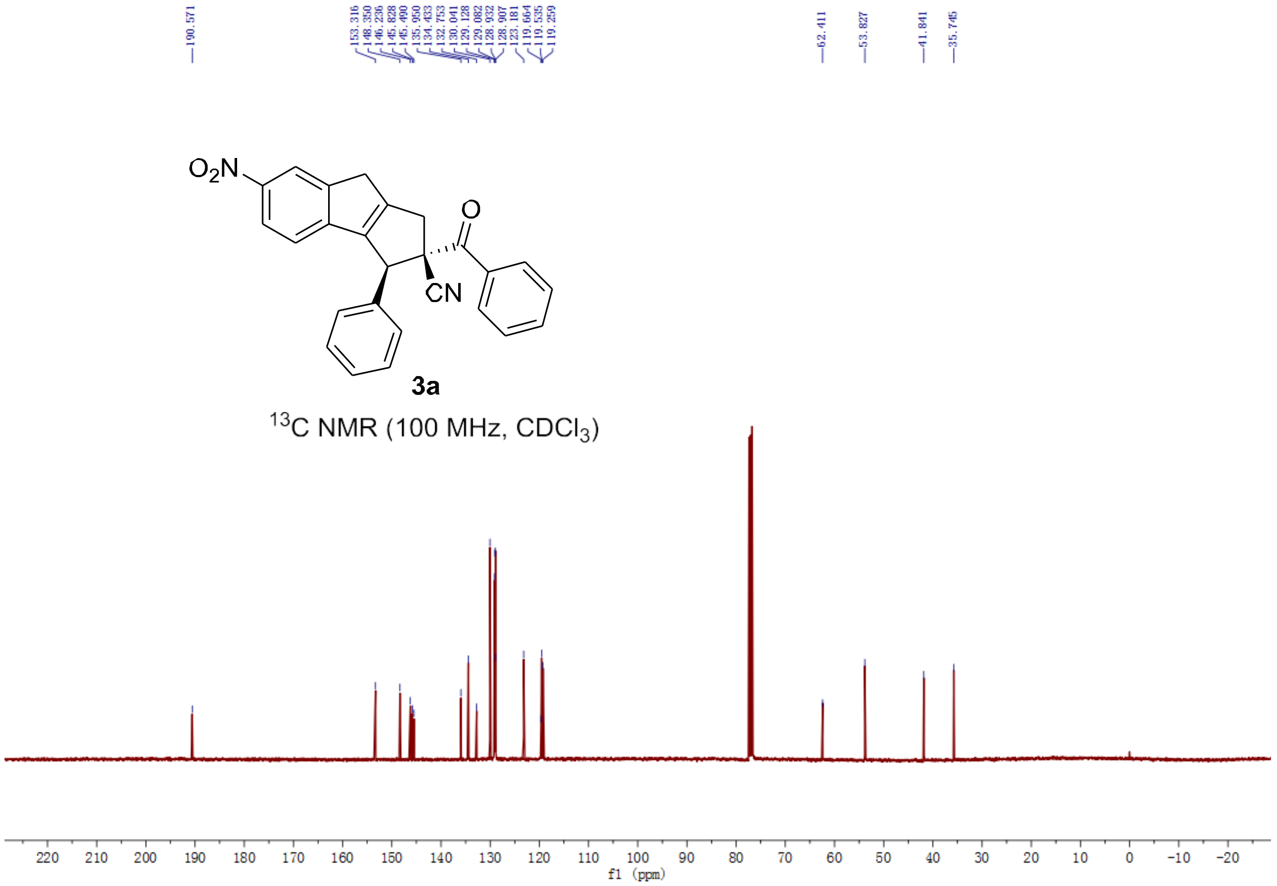

3a

^{13}C NMR (100 MHz, $CDCl_3$)

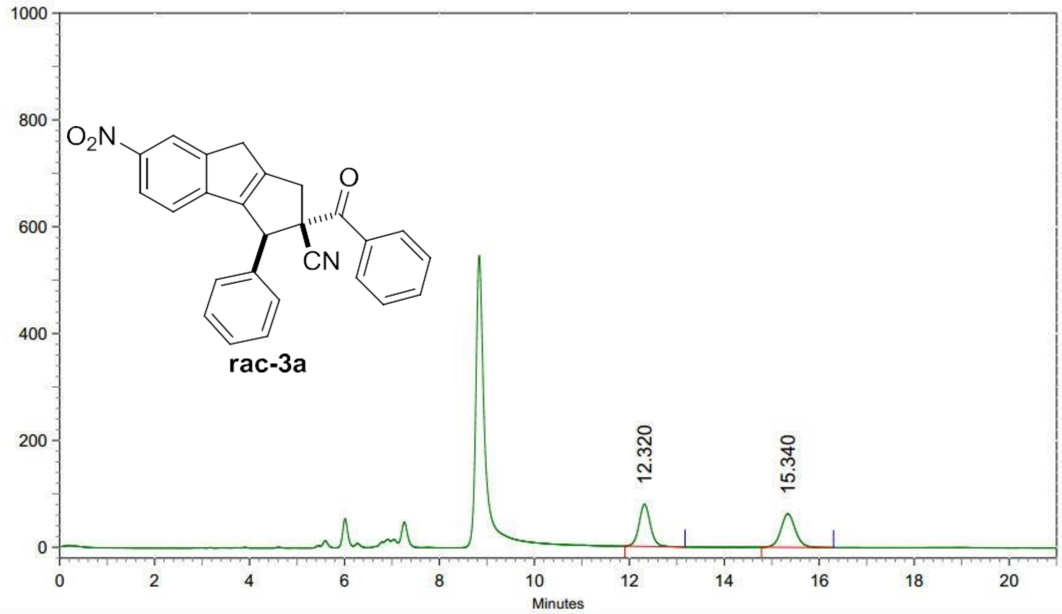

AREA PERCENT REPORT

Peak No.	Ret Time	Width	Height	Area	Area [%]
1	12.320	1.267	1321520	21879568	49.5716
2	15.340	1.513	1055084	22257713	50.4284

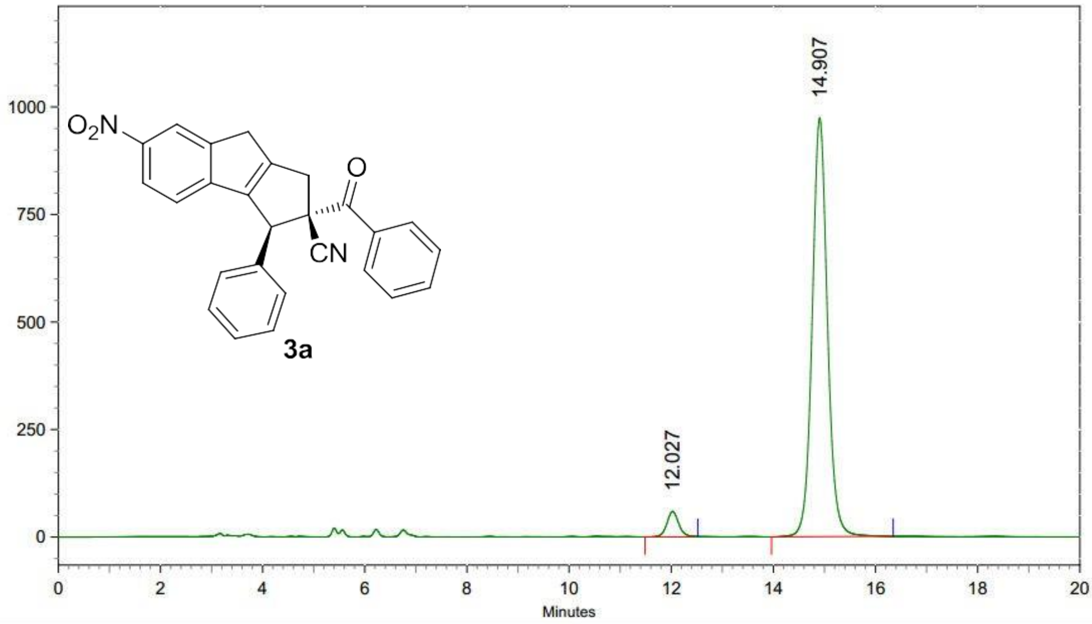

AREA PERCENT REPORT

Peak No.	Ret Time	Width	Height	Area	Area [%]
1	12.027	1.033	998434	16335364	4.6023
2	14.907	2.383	16334320	338606828	95.3977

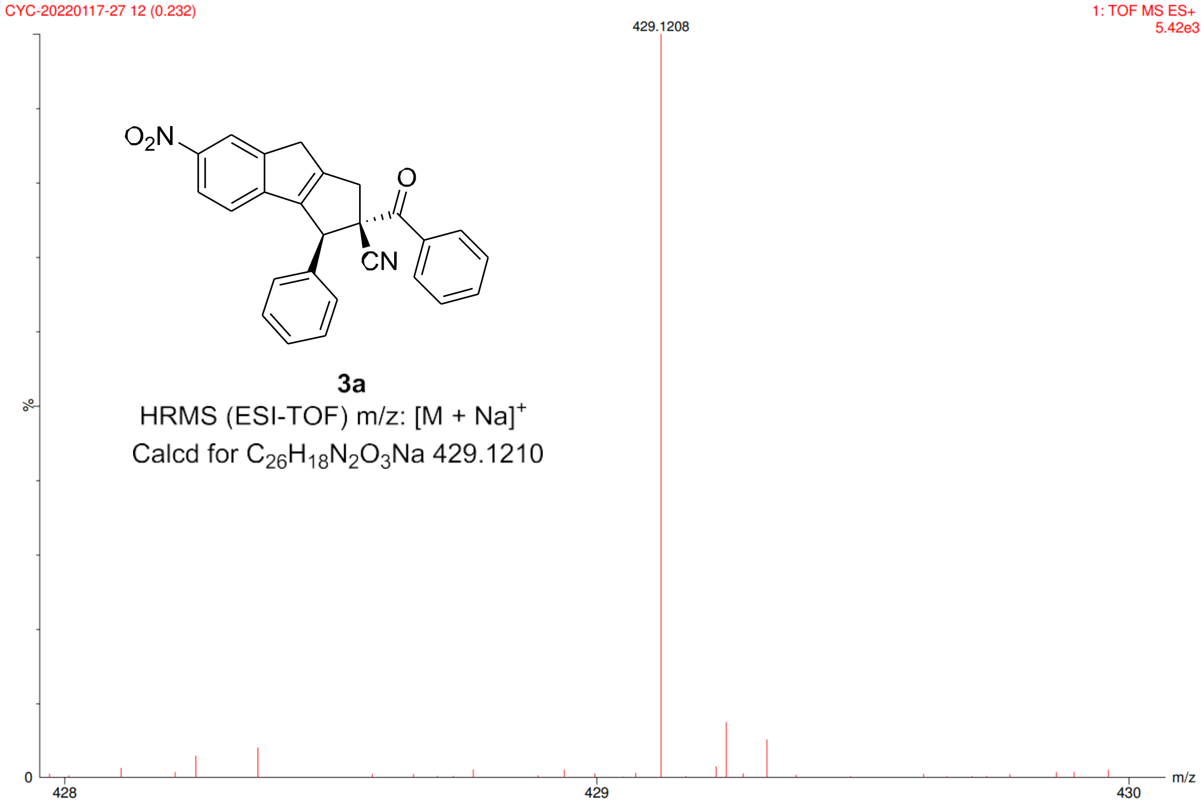
CYC-20220117-27 12 (0.232)
1: TOF MS ES+
5.42e3
429.1208
%
0
428
429
430
m/z
O2N
O
CN
3a
HRMS (ESI-TOF) m/z: [M + Na]+
Calcd for C26H18N2O3Na 429.1210

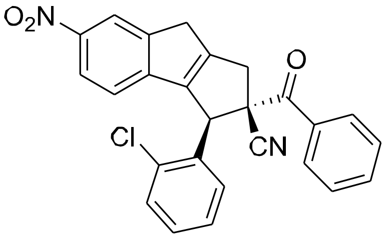

3b

^{1}H NMR (400 MHz, $CDCl_3$)

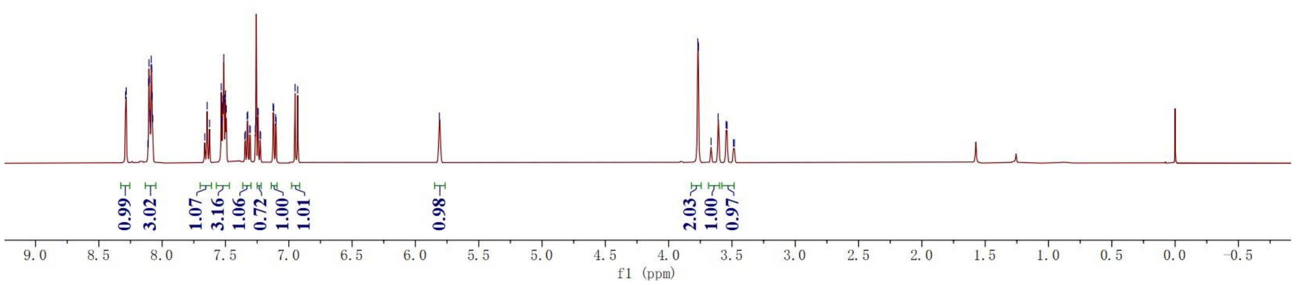

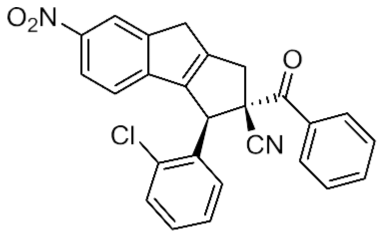

3b

^{13}C NMR (100 MHz, $CDCl_3$)

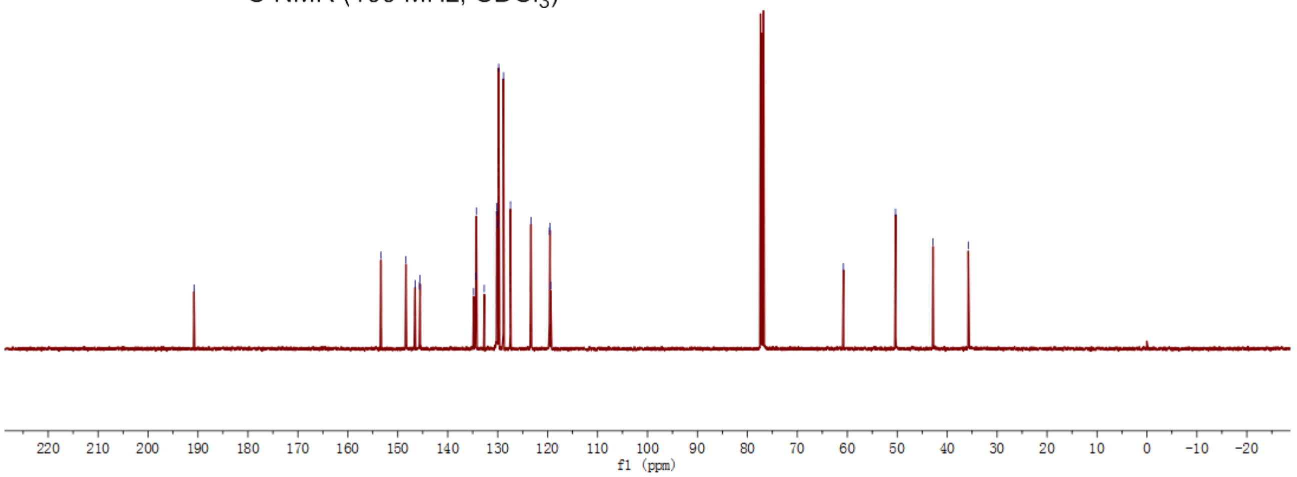

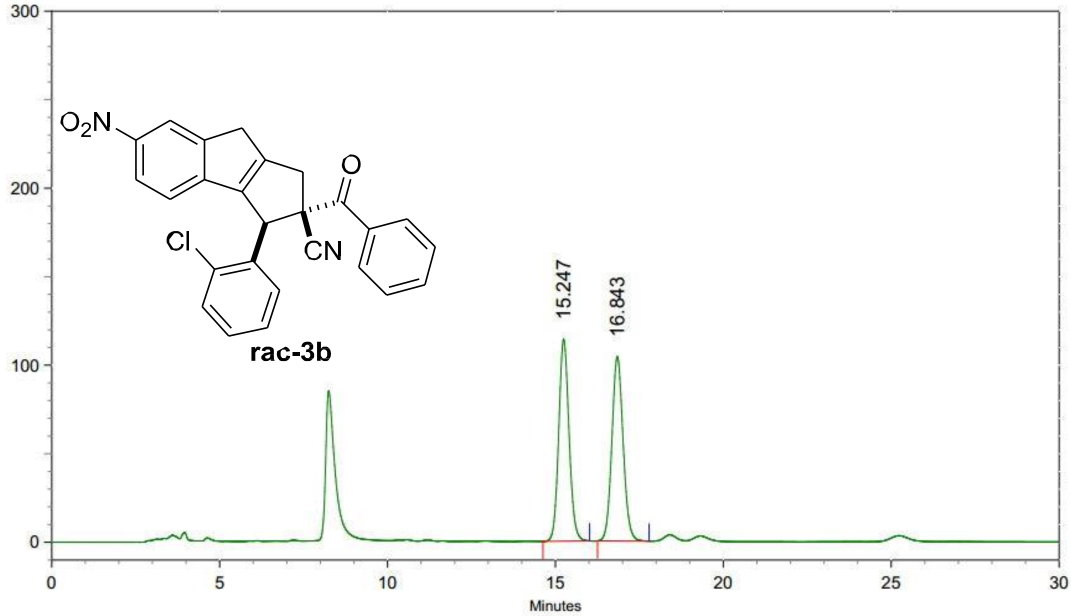

AREA PERCENT REPORT

Peak No.	Ret Time	Width	Height	Area	Area [%]
1	15.247	1.390	1914824	40880904	50.0501
2	16.843	1.537	1747360	40799080	49.9499

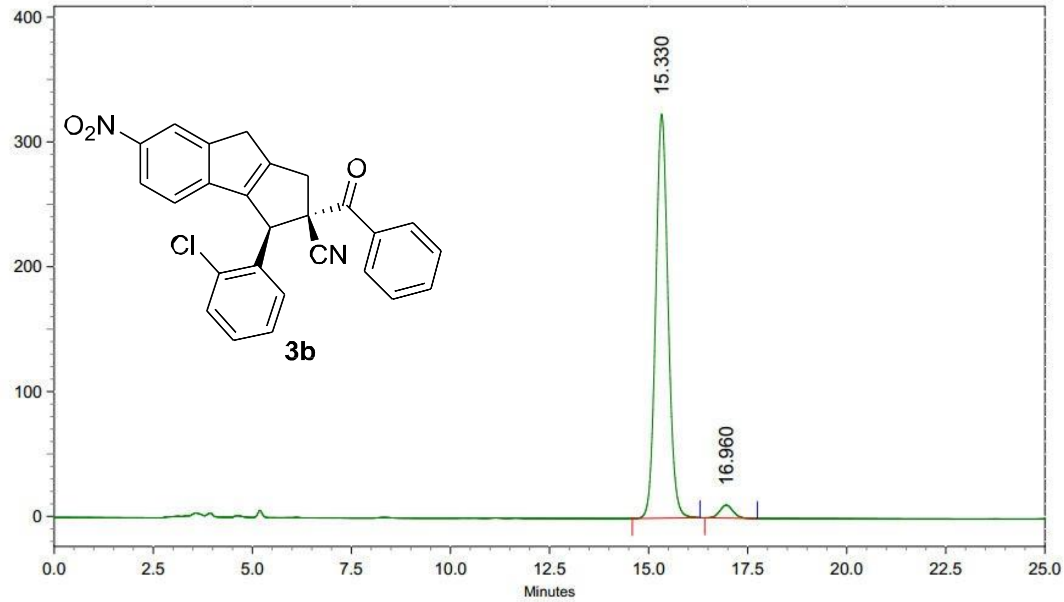

AREA PERCENT REPORT

Peak No.	Ret Time	Width	Height	Area	Area [%]
1	15.330	1.707	5427810	118074680	96.7091
2	16.960	1.330	173703	4017953	3.2909

Spectrum Plot Report

Name	CYC-20191103-7	Rack Pos.		Instrument	Instrument 1	Operator	
Inj. Vol. (ul)	10	Plate Pos.		IRM Status	Success		
Data File	CYC-20191103-7.d	Method (Acq)	TOF.m	Comment		Acq. Time (Local)	11/8/2019 10:38:36 AM (UTC+08:00)

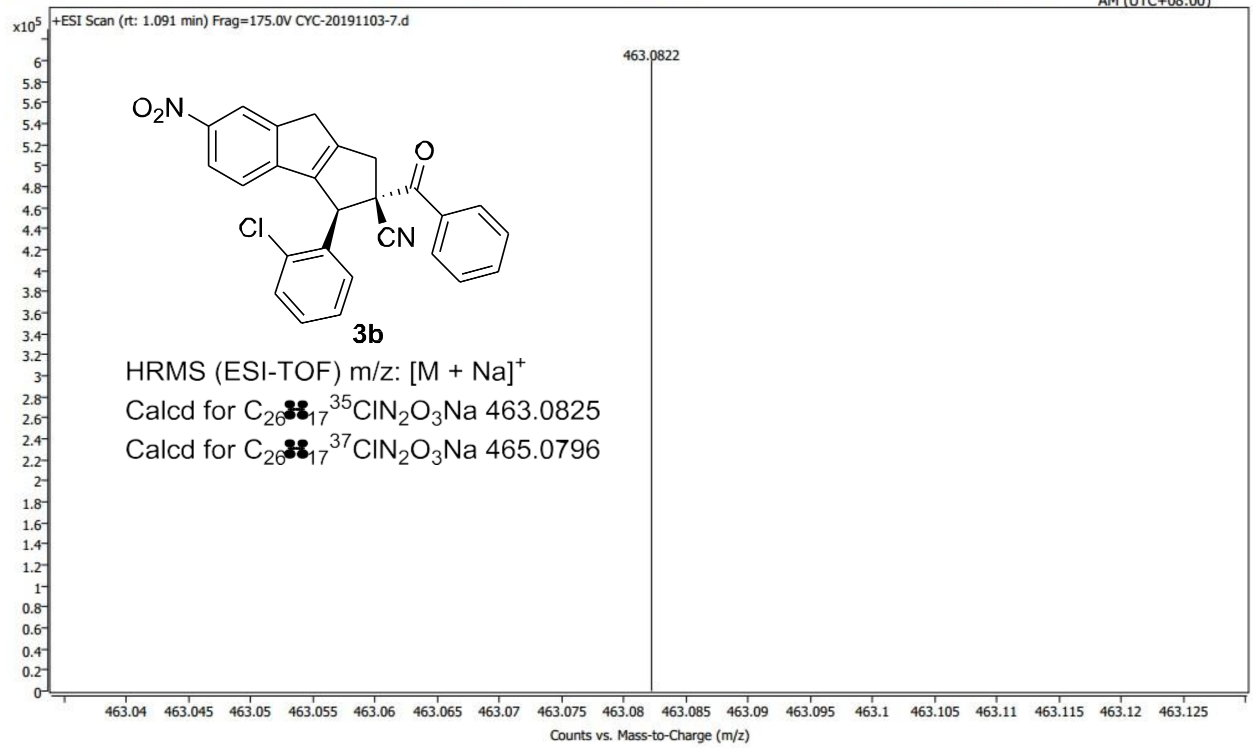

Spectrum Plot Report

Name	CYC-20191103-7	Rack Pos.		Instrument	Instrument 1	Operator	
Inj. Vol. (ul)	10	Plate Pos.		IRM Status	Success		
Data File	CYC-20191103-7.d	Method (Acq)	TOF.m	Comment		Acq. Time (Local)	11/8/2019 10:38:36 AM (UTC+08:00)

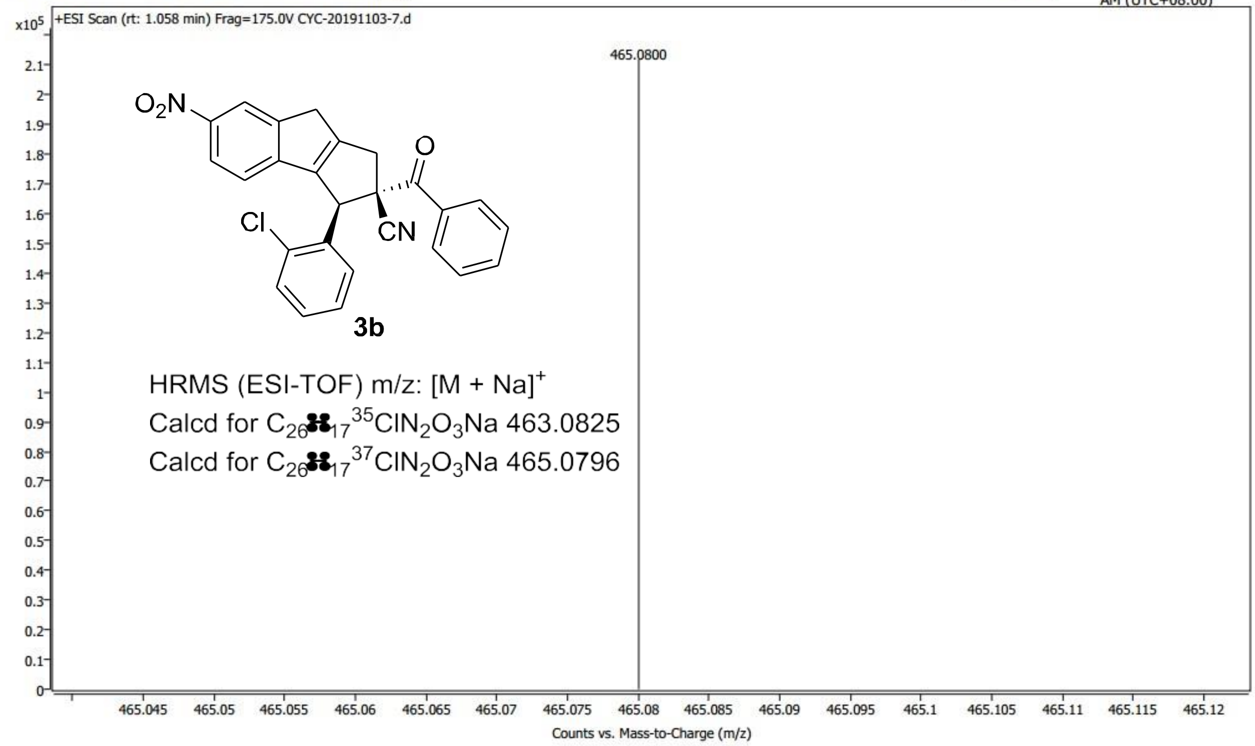

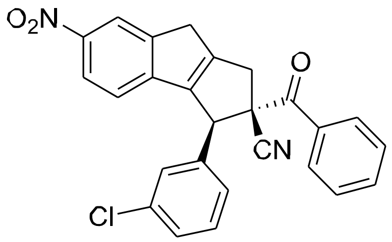

3c

^{1}H NMR (400 MHz, $CDCl_3$)

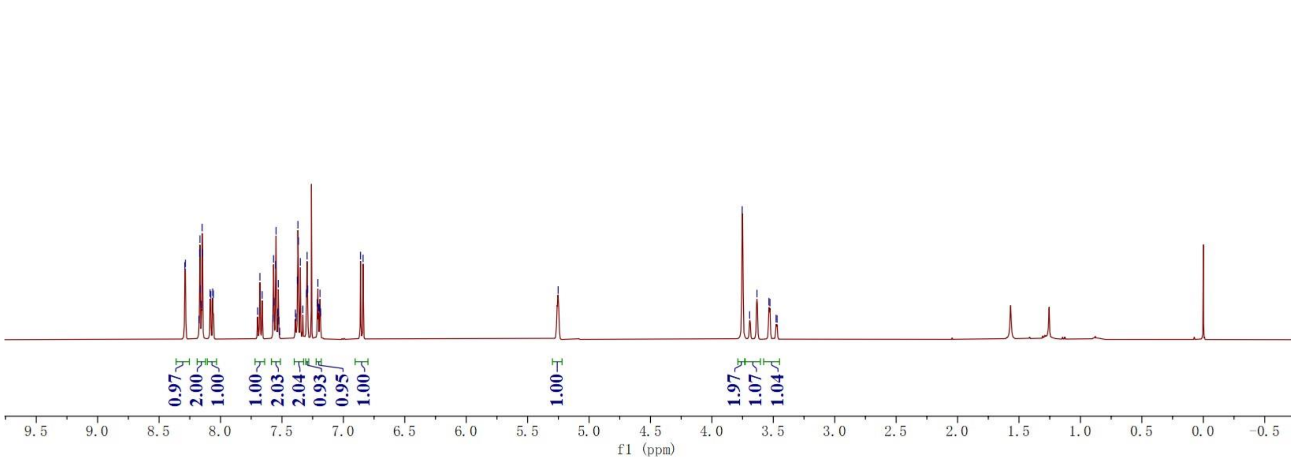

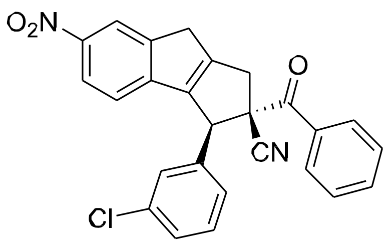

3c

^{13}C NMR (100 MHz, $CDCl_3$)

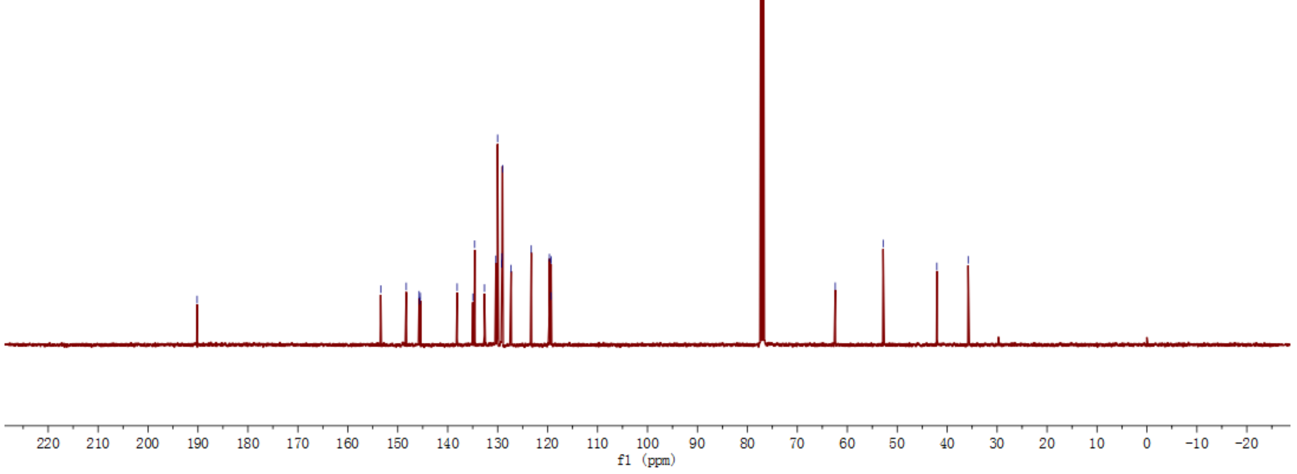

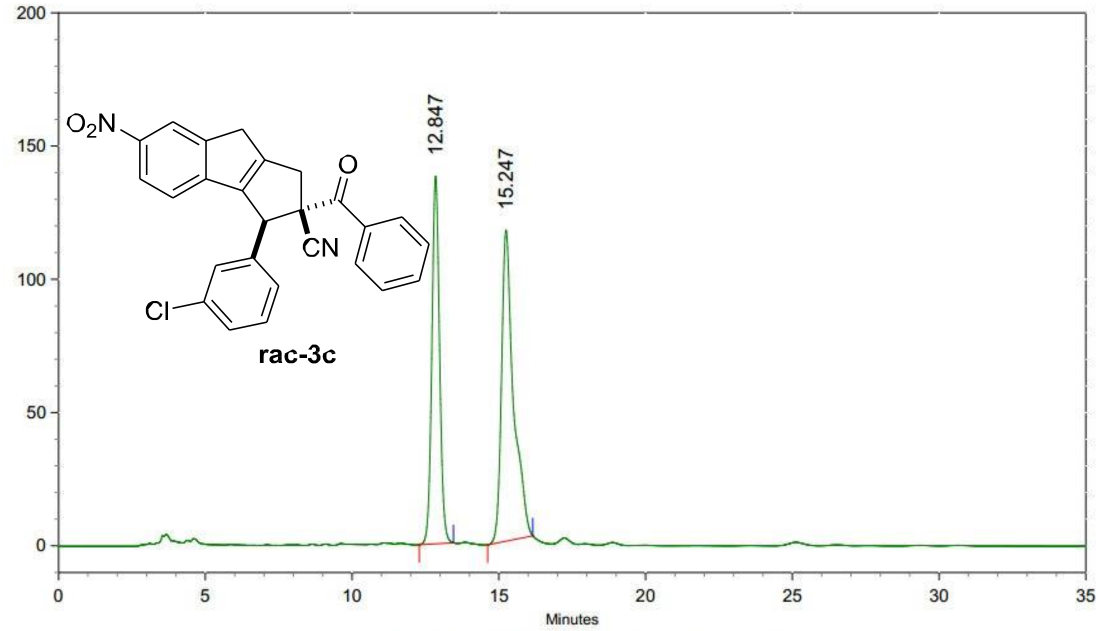

AREA PERCENT REPORT

Peak No.	Ret Time	Width	Height	Area	Area [%]
1	12.847	1.163	2314980	42109270	43.1324
2	15.247	1.537	1957978	55518592	56.8676

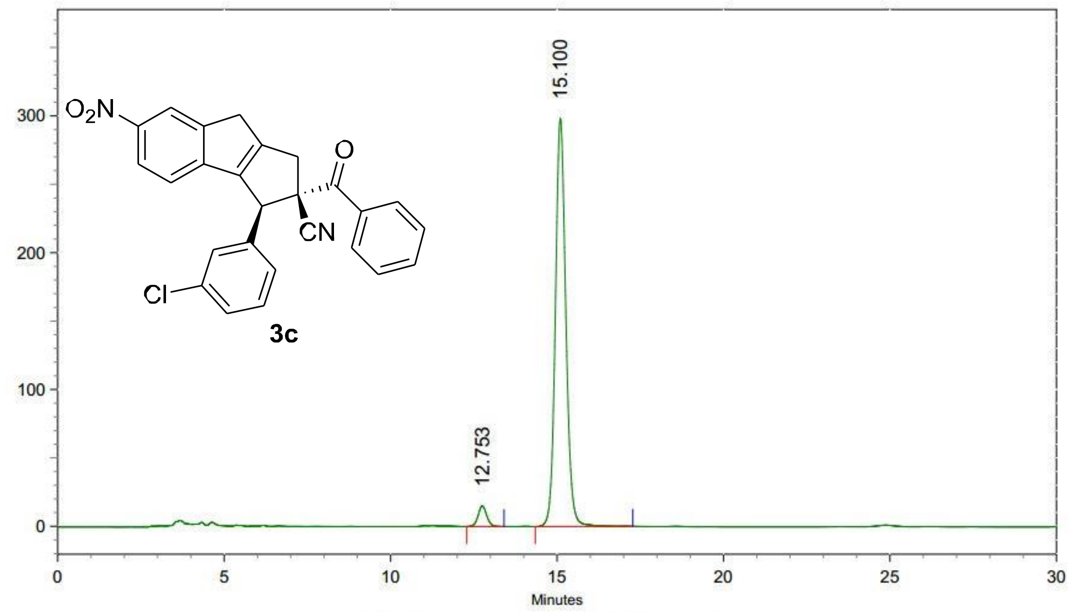

AREA PERCENT REPORT

Peak No.	Ret Time	Width	Height	Area	Area [%]
1	12.753	1.117	253142	4582450	4.0329
2	15.100	2.930	5005440	109043702	95.9671

Spectrum Plot Report

Name	CYC-20191018-66	Rack Pos.		Instrument	Instrument 1	Operator	
Inj. Vol. (ul)	10	Plate Pos.		IRM Status	Success		
Data File	CYC-20191018-66.d	Method (Acq)	TOF.m	Comment		Acq. Time (Local)	10/29/2019 4:13:27 PM (UTC+08:00)

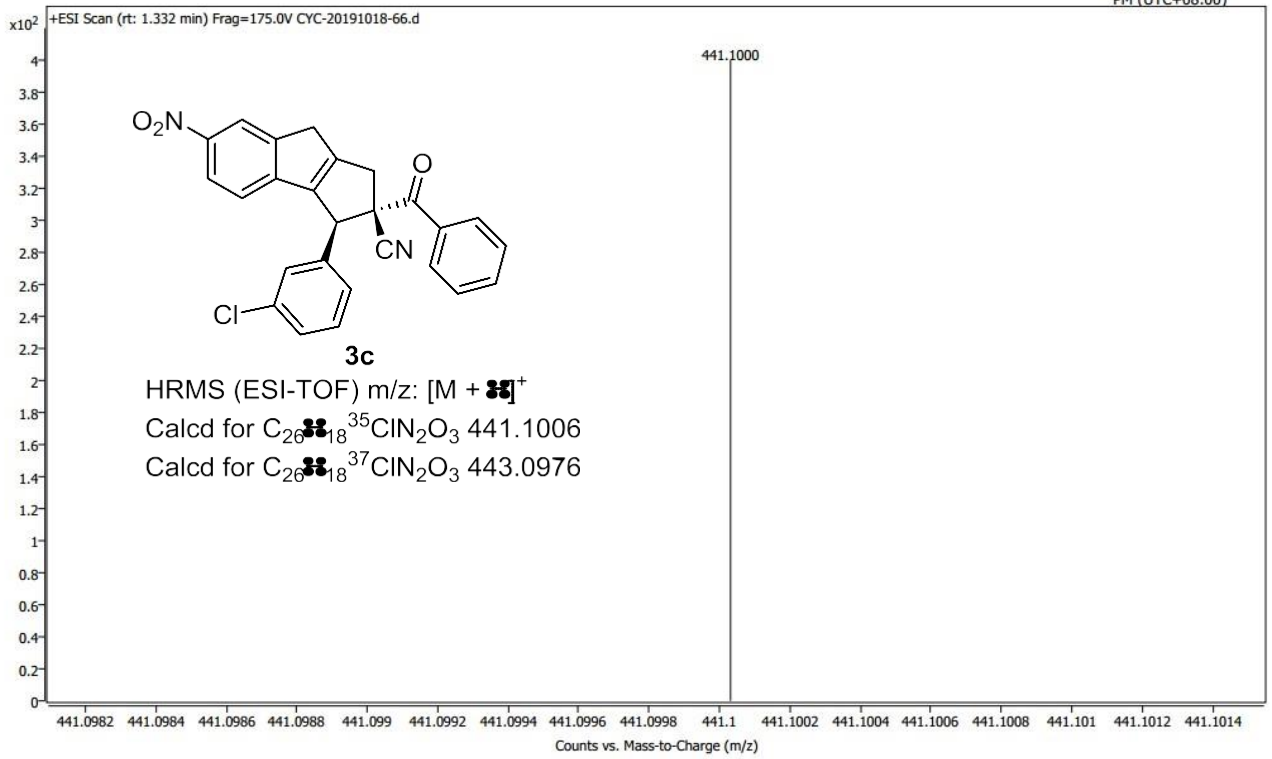

Spectrum Plot Report

Name	CYC-20191018-66	Rack Pos.		Instrument	Instrument 1	Operator	
Inj. Vol. (ul)	10	Plate Pos.		IRM Status	Success		
Data File	CYC-20191018-66.d	Method (Acq)	TOF.m	Comment		Acq. Time (Local)	10/29/2019 4:13:27 PM (UTC+08:00)

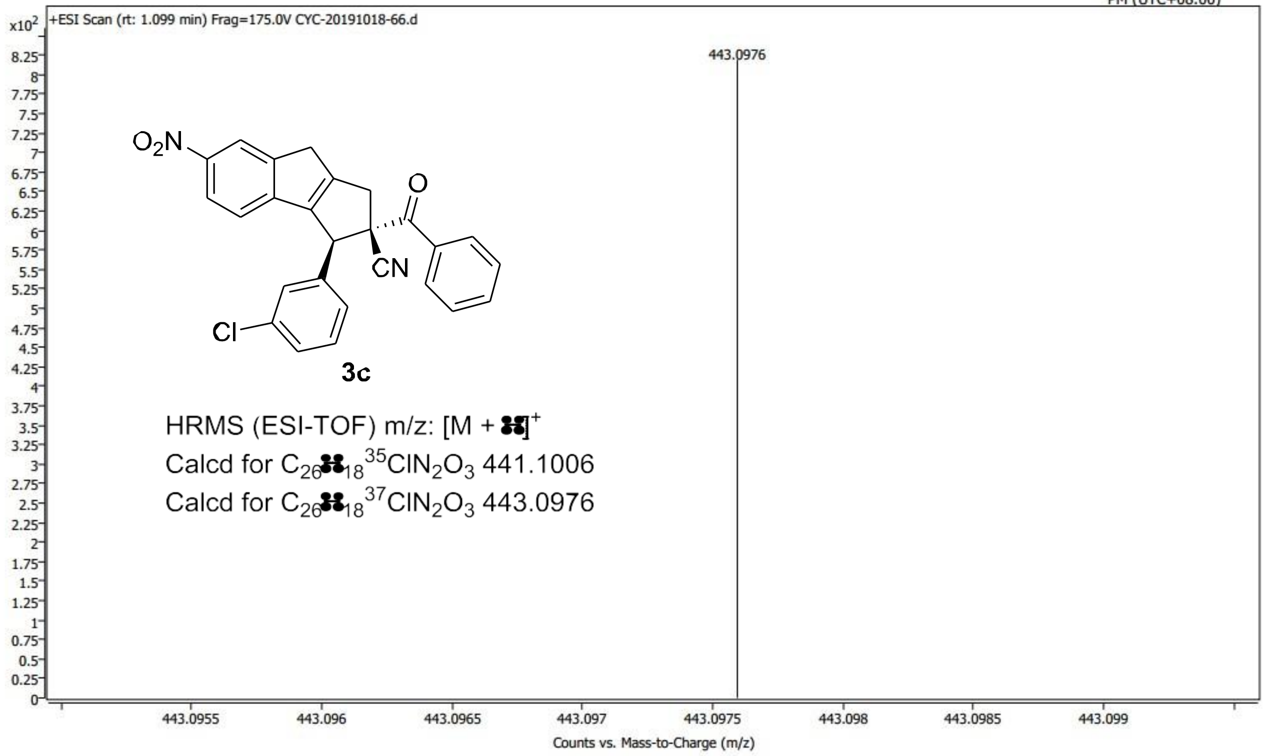

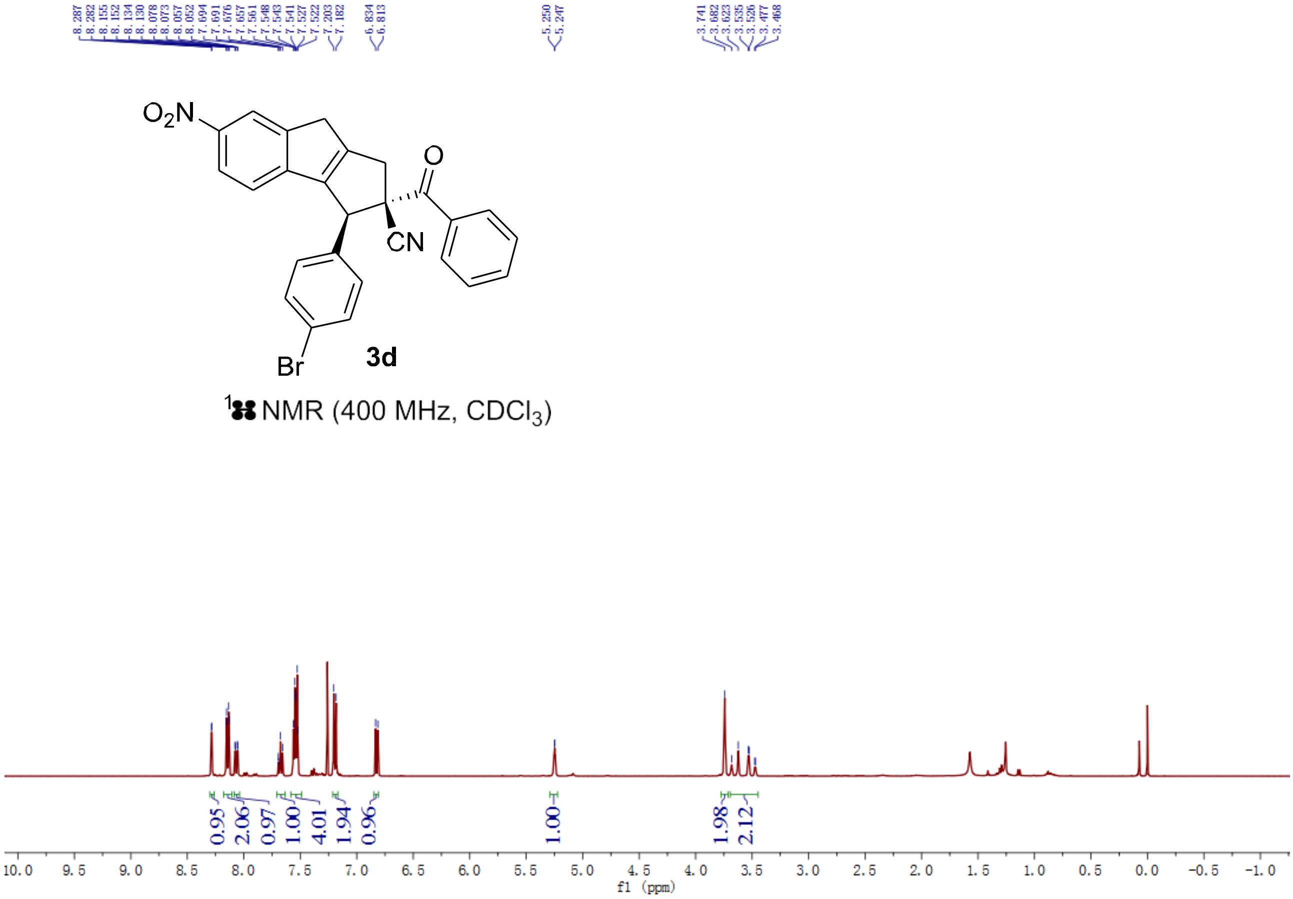

3d

^{1}[illegible] NMR (400 MHz, $CDCl_3$)

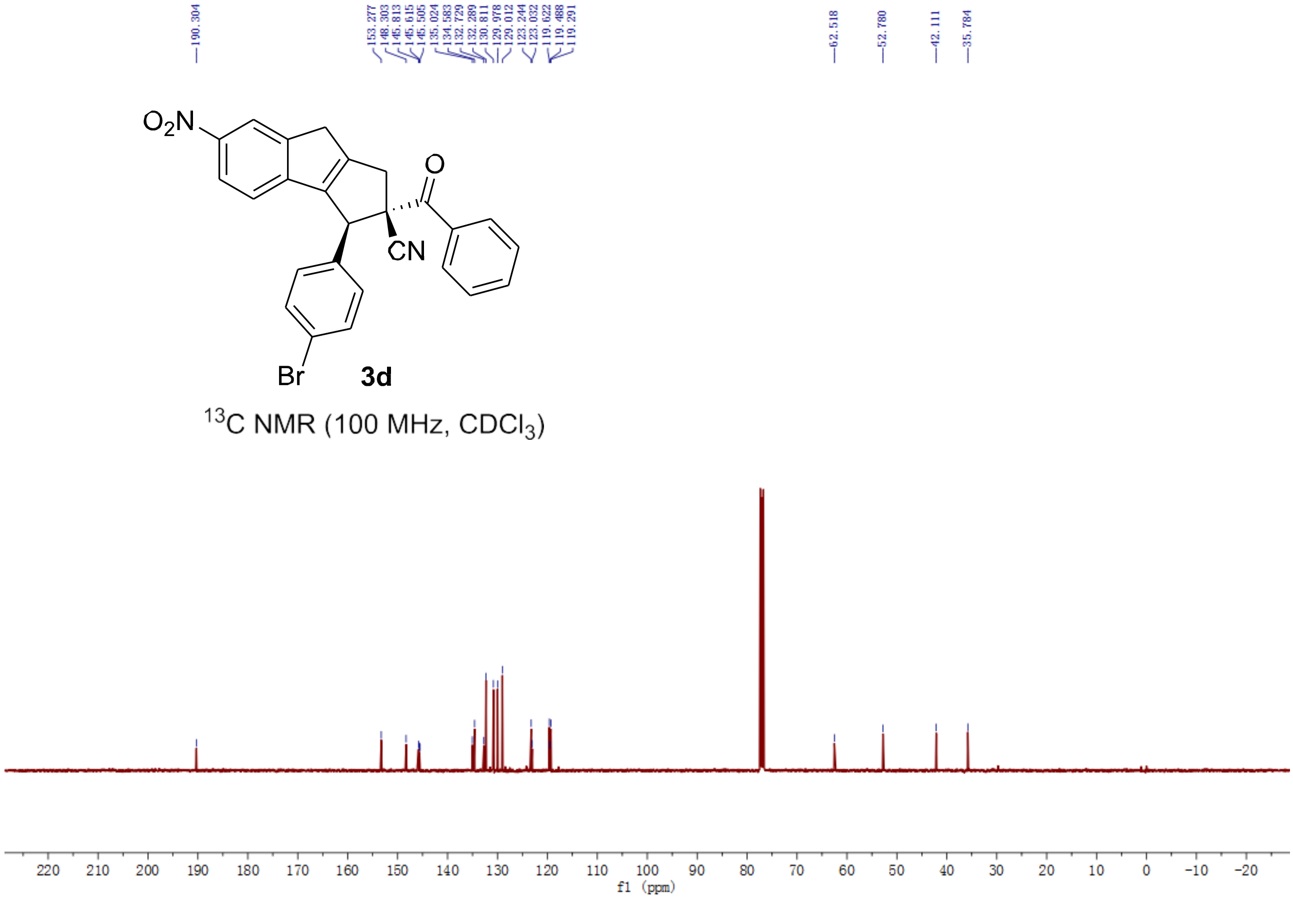

3d

^{13}C NMR (100 MHz, $CDCl_3$)

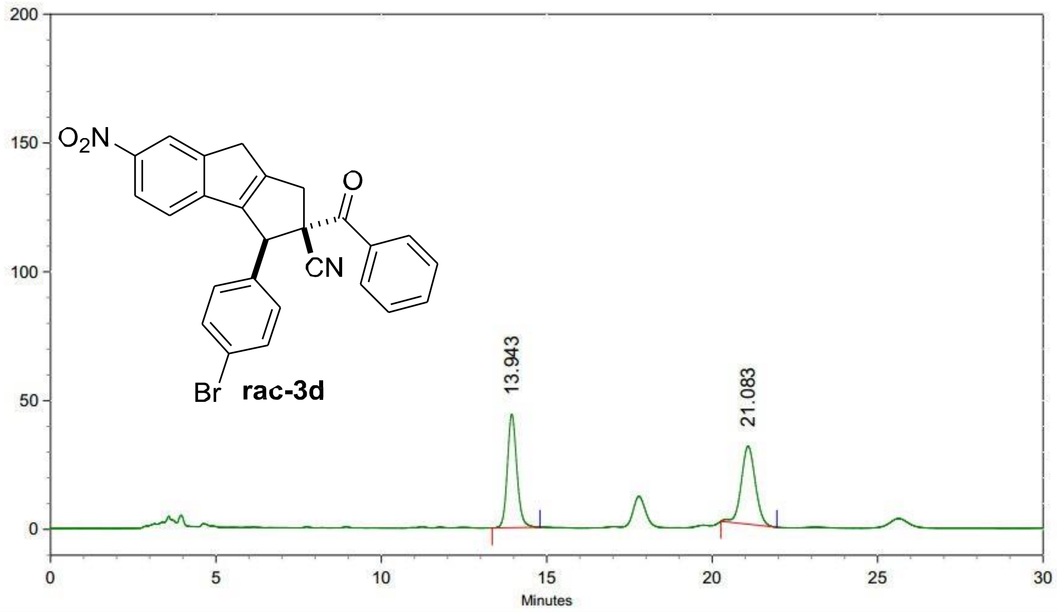

AREA PERCENT REPORT

Peak No.	Ret Time	Width	Height	Area	Area [%]
1	13.943	1.443	738074	14700066	49.4505
2	21.083	1.693	508083	15026763	50.5495

44

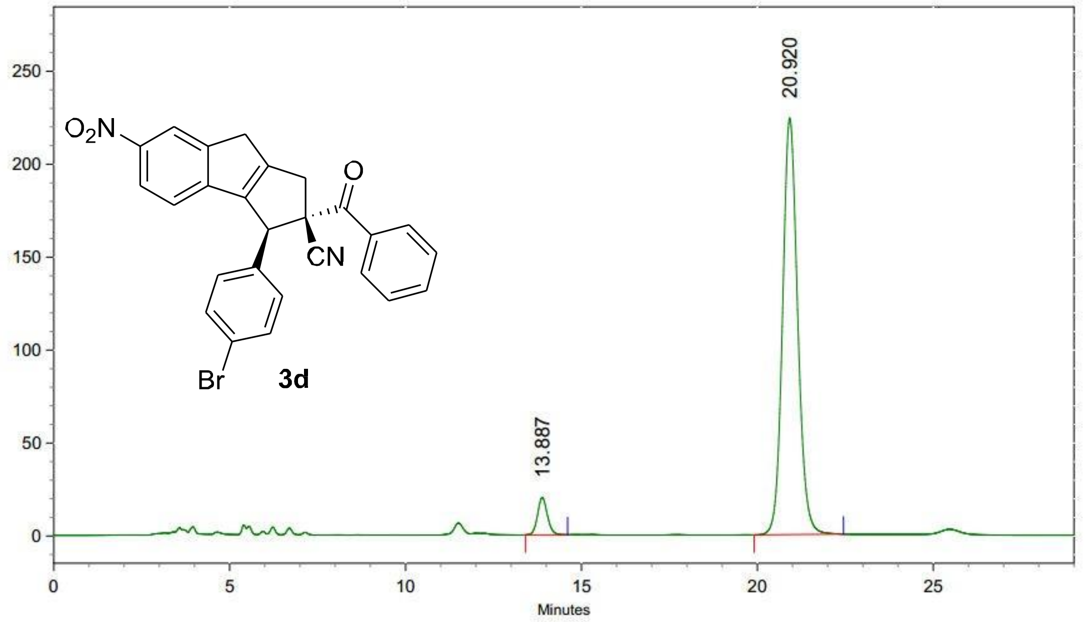

AREA PERCENT REPORT

Peak No.	Ret Time	Width	Height	Area	Area [%]
1	13.887	1.200	336924	6551077	5.5246
2	20.920	2.540	3758984	112030071	94.4754

Spectrum Plot Report

Agilent

Name	CYC-20191018-62	Rack Pos.		Instrument	Instrument 1	Operator	
Inj. Vol. (ul)	10	Plate Pos.		IRM Status	Success		
Data File	CYC-20191018-62.d	Method (Acq)	TOF.m	Comment		Acq. Time (Local)	10/29/2019 4:01:24 PM (UTC+08:00)

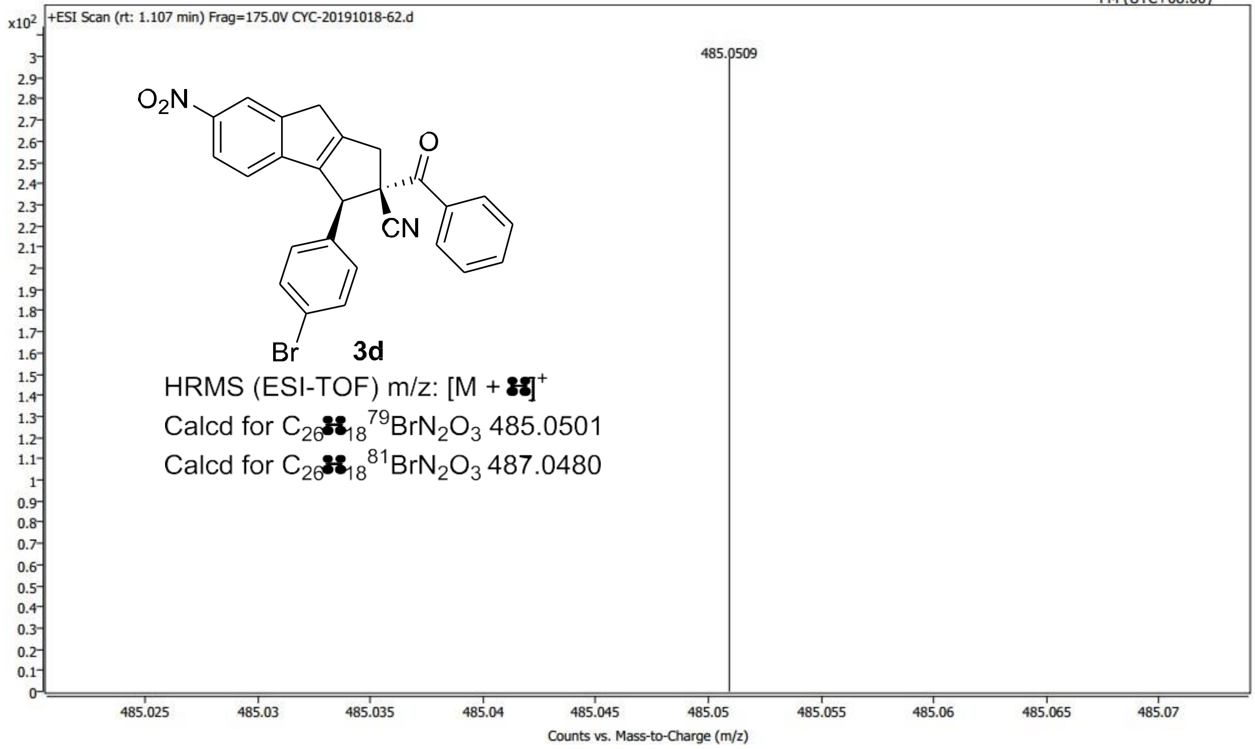

Spectrum Plot Report

Agilent

Name	CYC-20191018-62	Rack Pos.		Instrument	Instrument 1	Operator	
Inj. Vol. (ul)	10	Plate Pos.		IRM Status	Success		
Data File	CYC-20191018-62.d	Method (Acq)	TOF.m	Comment		Acq. Time (Local)	10/29/2019 4:01:24 PM (UTC+08:00)

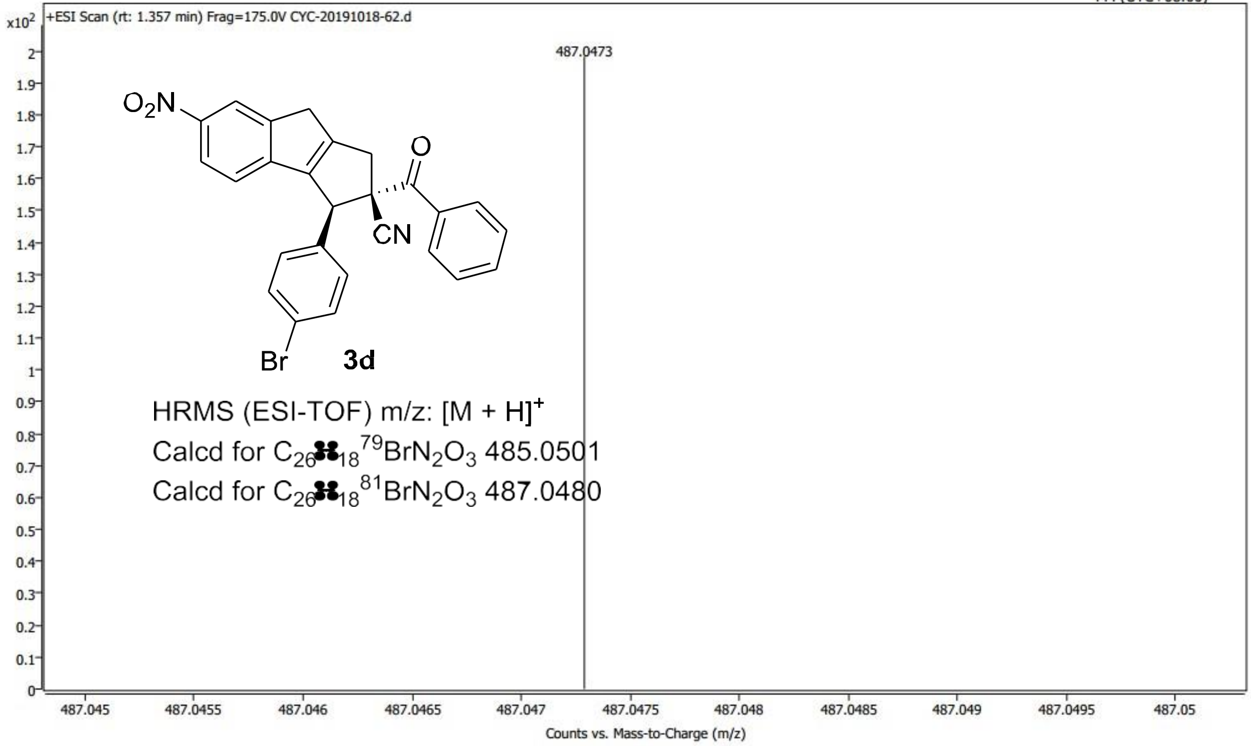

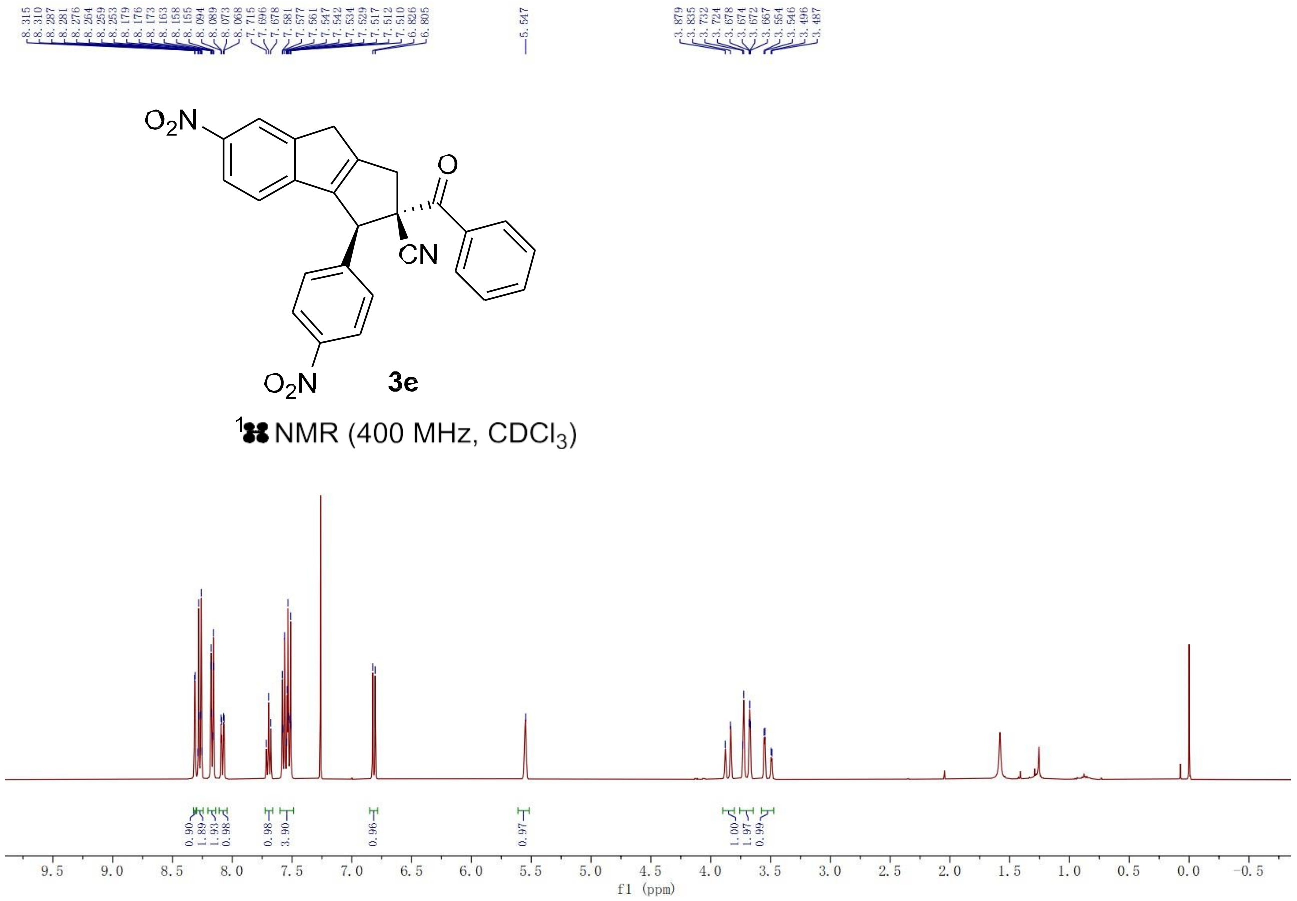

3e

^{1}H NMR (400 MHz, $CDCl_3$)

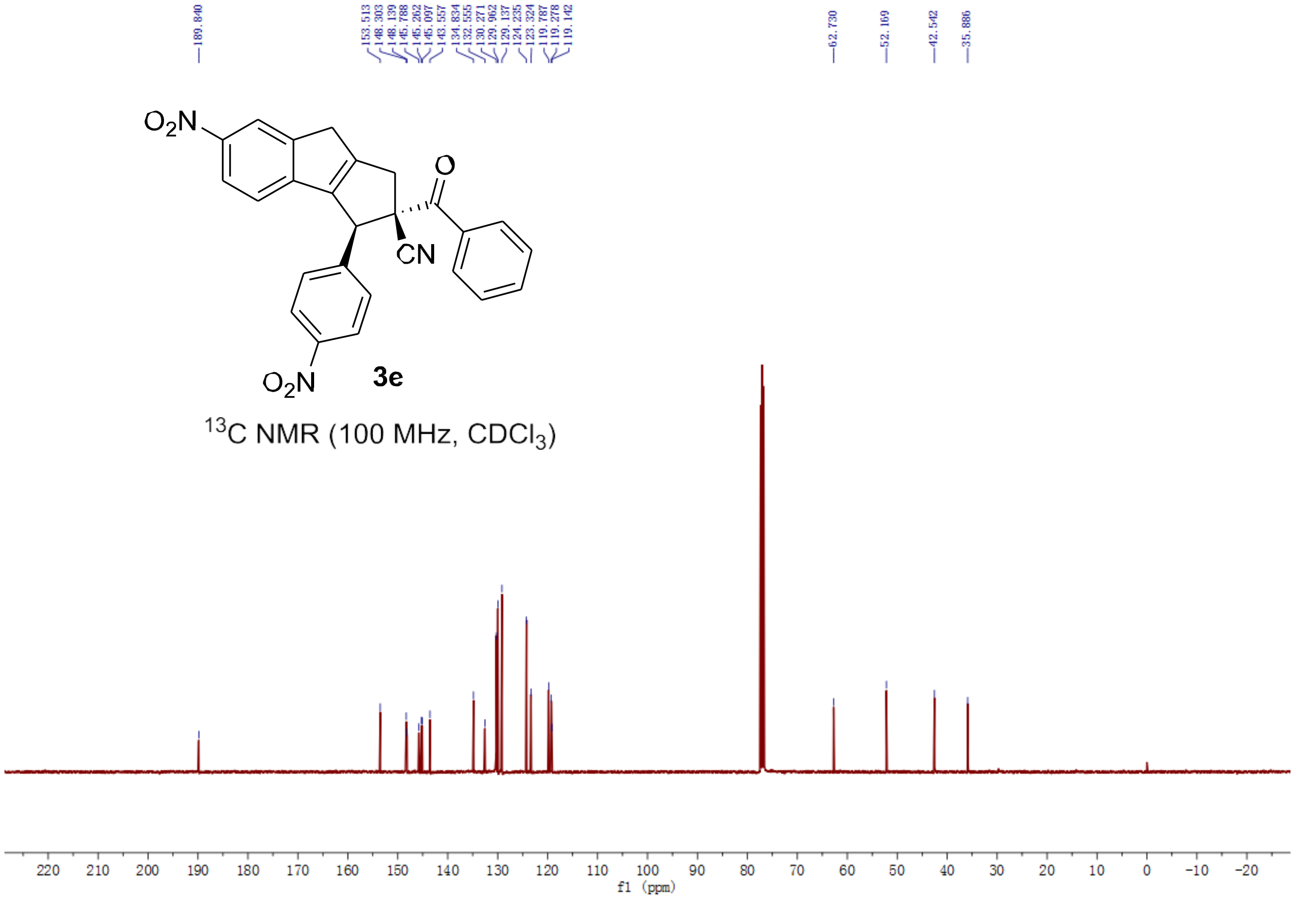

3e

^{13}C NMR (100 MHz, $CDCl_3$)

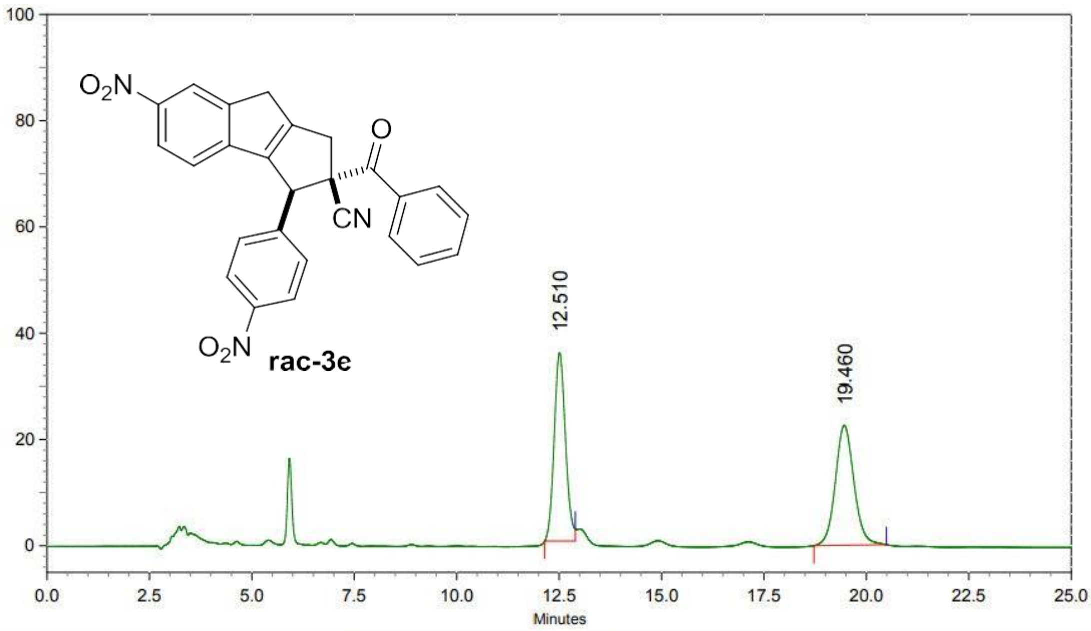

AREA PERCENT REPORT

Peak No.	Ret Time	Width	Height	Area	Area [%]
1	12.510	0.747	595146	11400732	48.8354
2	19.460	1.770	378707	11944488	51.1646

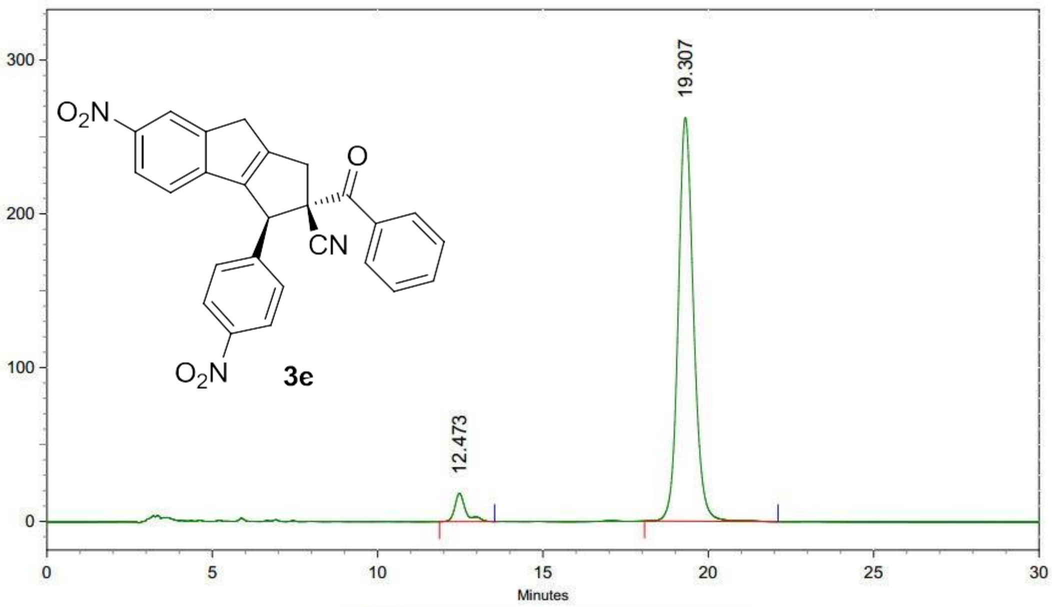

AREA PERCENT REPORT

Peak No.	Ret Time	Width	Height	Area	Area [%]
1	12.473	1.667	307638	7123908	4.7871
2	19.307	4.040	4404650	141689719	95.2129

Spectrum Plot Report

Agilent

Name	CYC-20191025-12	Rack Pos.		Instrument	Instrument 1	Operator	
Inj. Vol. (ul)	10	Plate Pos.		IRM Status	Success		
Data File	CYC-20191025-12.d	Method (Acq)	TOF.m	Comment		Acq. Time (Local)	11/9/2019 11:13:19 AM (UTC+08:00)

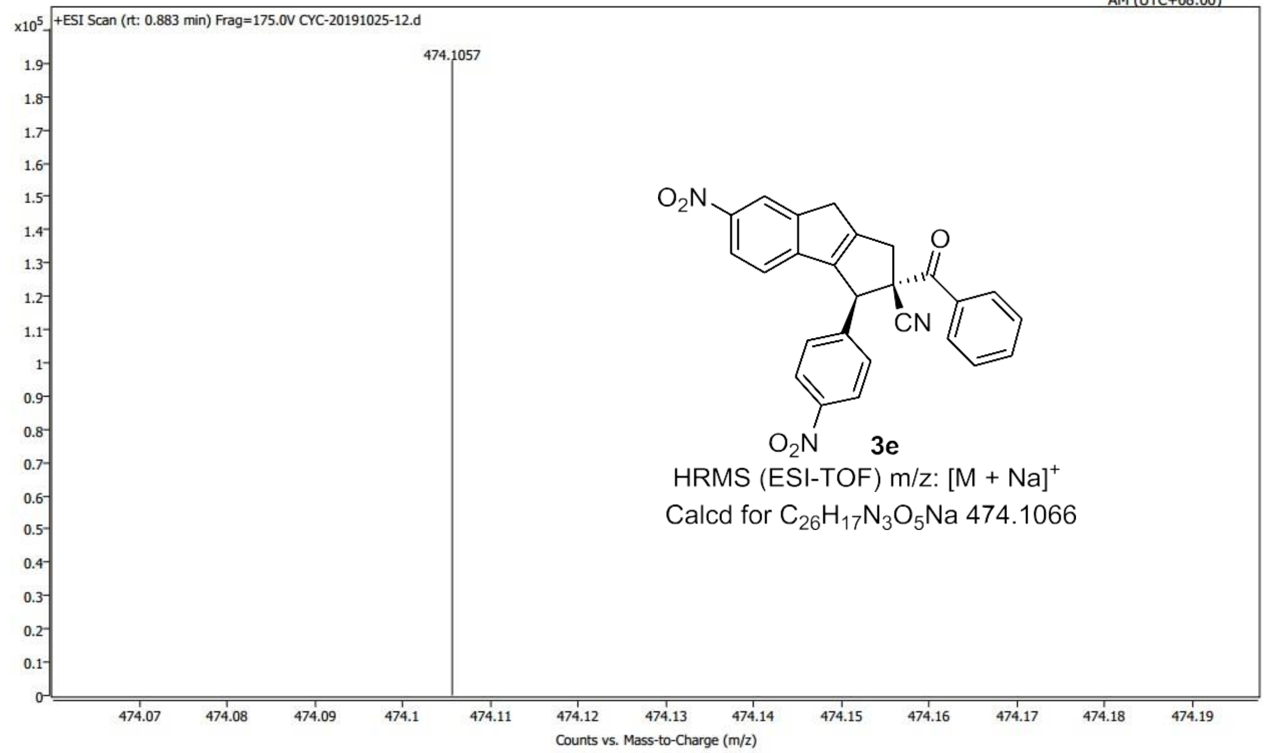

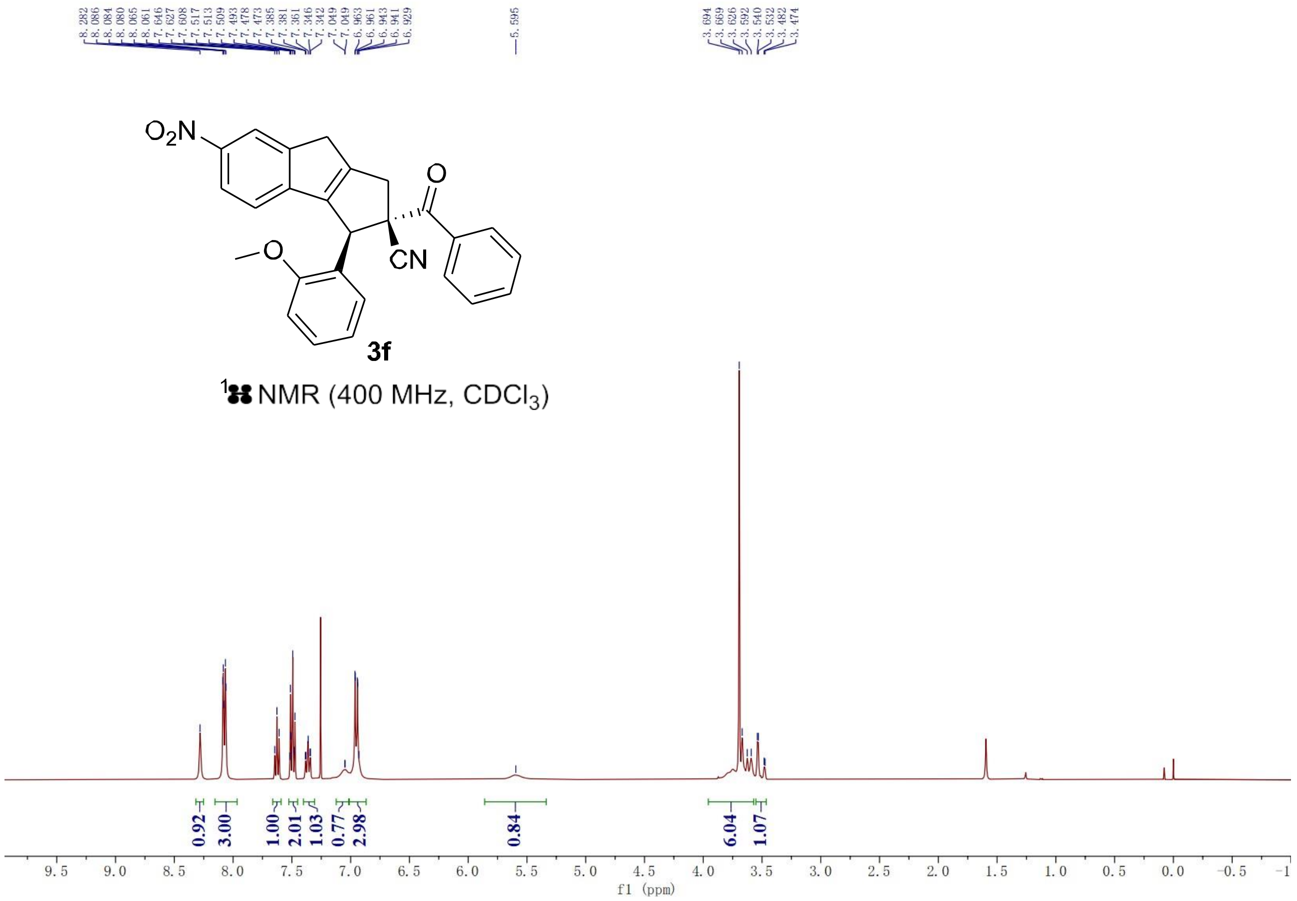
8.282
8.086
8.084
8.080
8.065
8.061
7.646
7.627
7.608
7.517
7.513
7.509
7.493
7.478
7.473
7.385
7.381
7.361
7.346
7.342
7.049
7.049
6.963
6.961
6.943
6.941
6.929
5.596
3.694
3.669
3.626
3.592
3.540
3.532
3.482
3.474
O2N
O
O
CN
3f
1H NMR (400 MHz, CDCl3)
0.92
3.00
1.00
2.01
1.03
0.77
2.98
0.84
6.04
1.07
f1 (ppm)

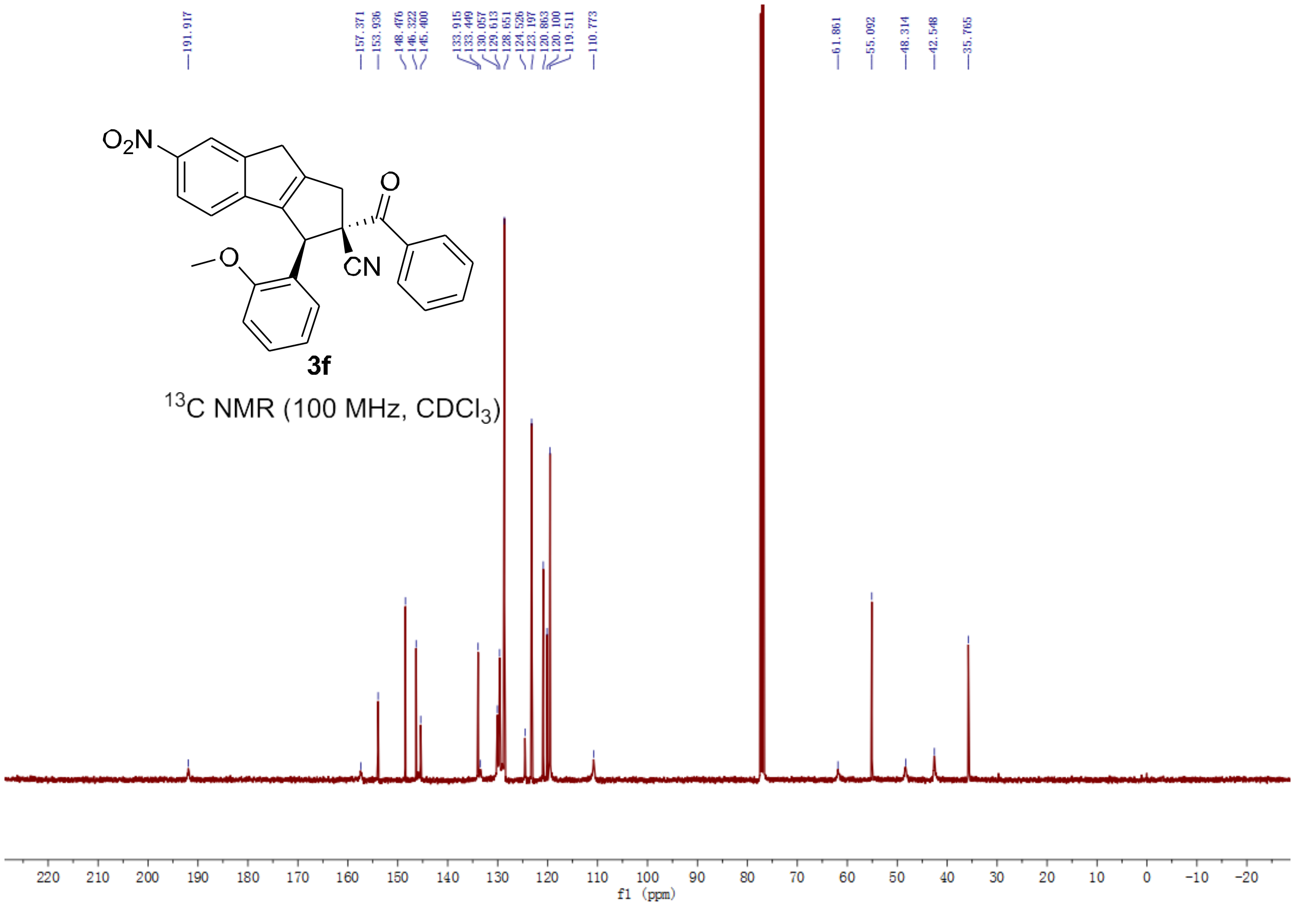
191.917
157.371
153.936
148.476
146.322
145.400
133.915
133.449
130.057
129.613
128.651
124.526
123.197
120.863
120.100
119.511
110.773
61.861
55.092
48.314
42.548
35.765
O2N
O
O
CN
3f
13C NMR (100 MHz, CDCl3)
f1 (ppm)

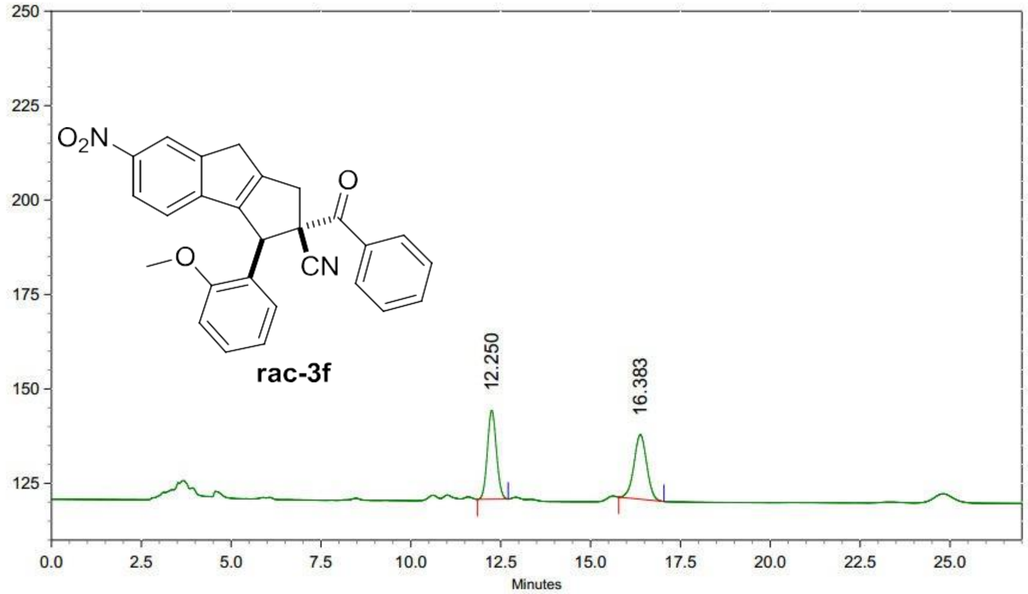

AREA PERCENT REPORT

Peak No.	Ret Time	Width	Height	Area	Area [%]
1	12.250	0.857	394552	6888211	49.3050
2	16.383	1.260	287049	7082392	50.6950

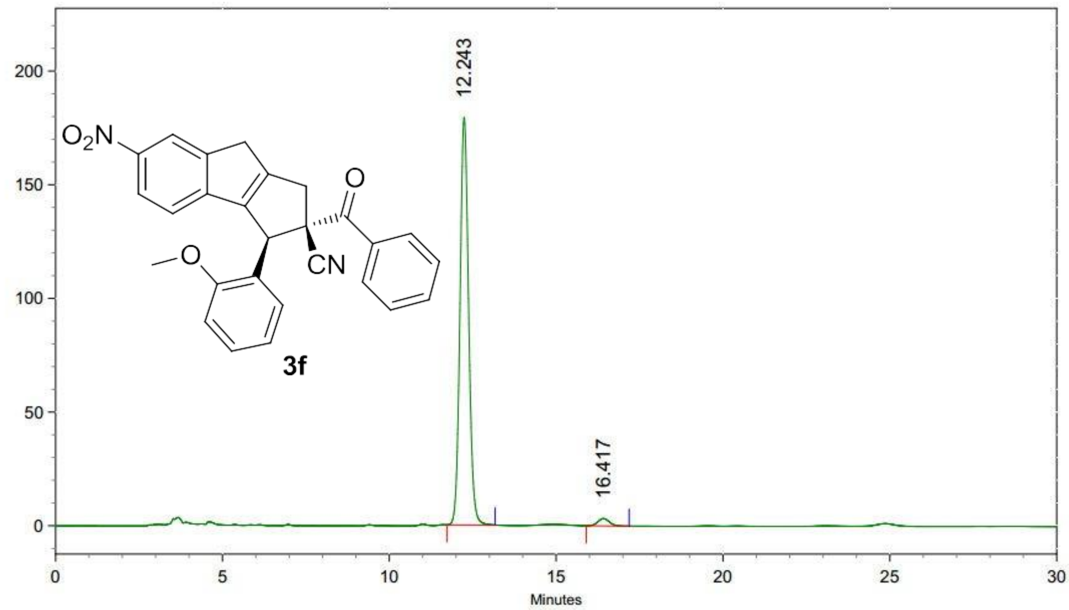

AREA PERCENT REPORT

Peak No.	Ret Time	Width	Height	Area	Area [%]
1	12.243	1.447	3007452	53967482	97.6296
2	16.417	1.283	54190	1310315	2.3704

Spectrum Plot Report

Name	CYC-20191124-7	Rack Pos.		Instrument	Instrument 1	Operator	
Inj. Vol. (ul)	10	Plate Pos.		IRM Status	Success		
Data File	CYC-20191124-7.d	Method (Acq)	TOF.m	Comment		Acq. Time (Local)	12/5/2019 4:51:21 PM (UTC+08:00)

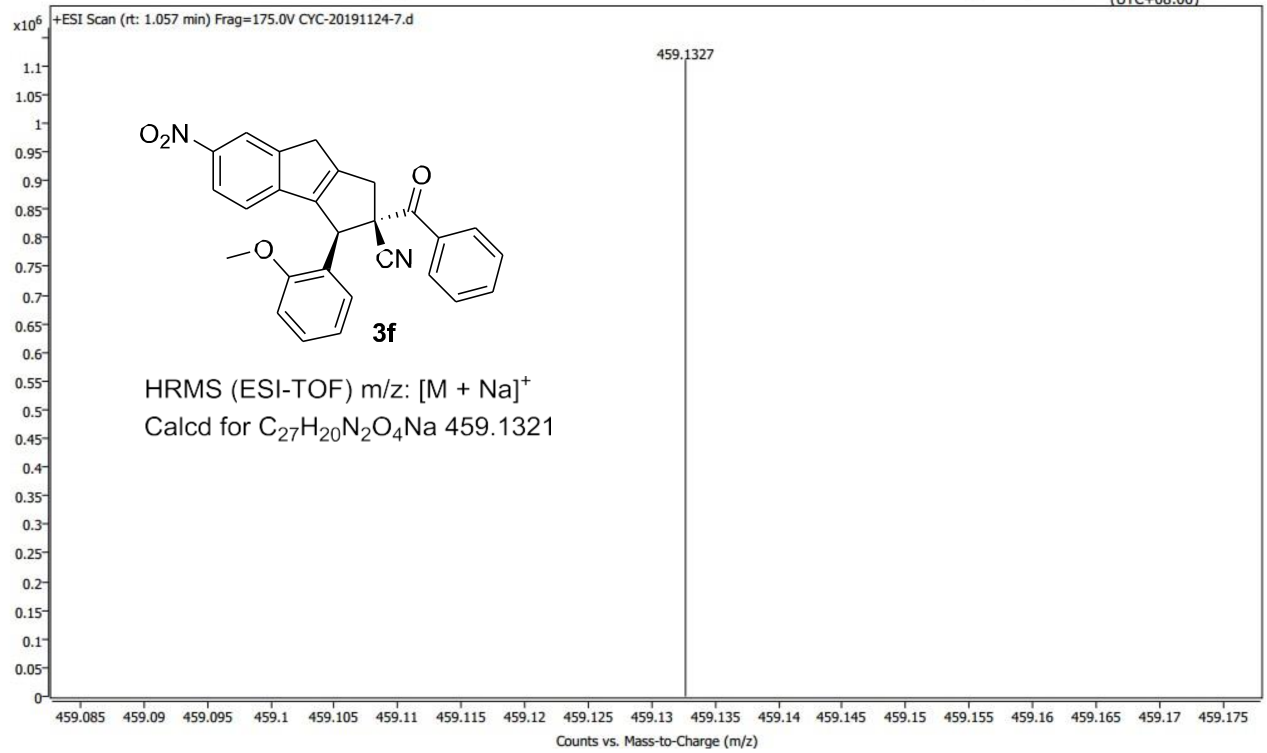

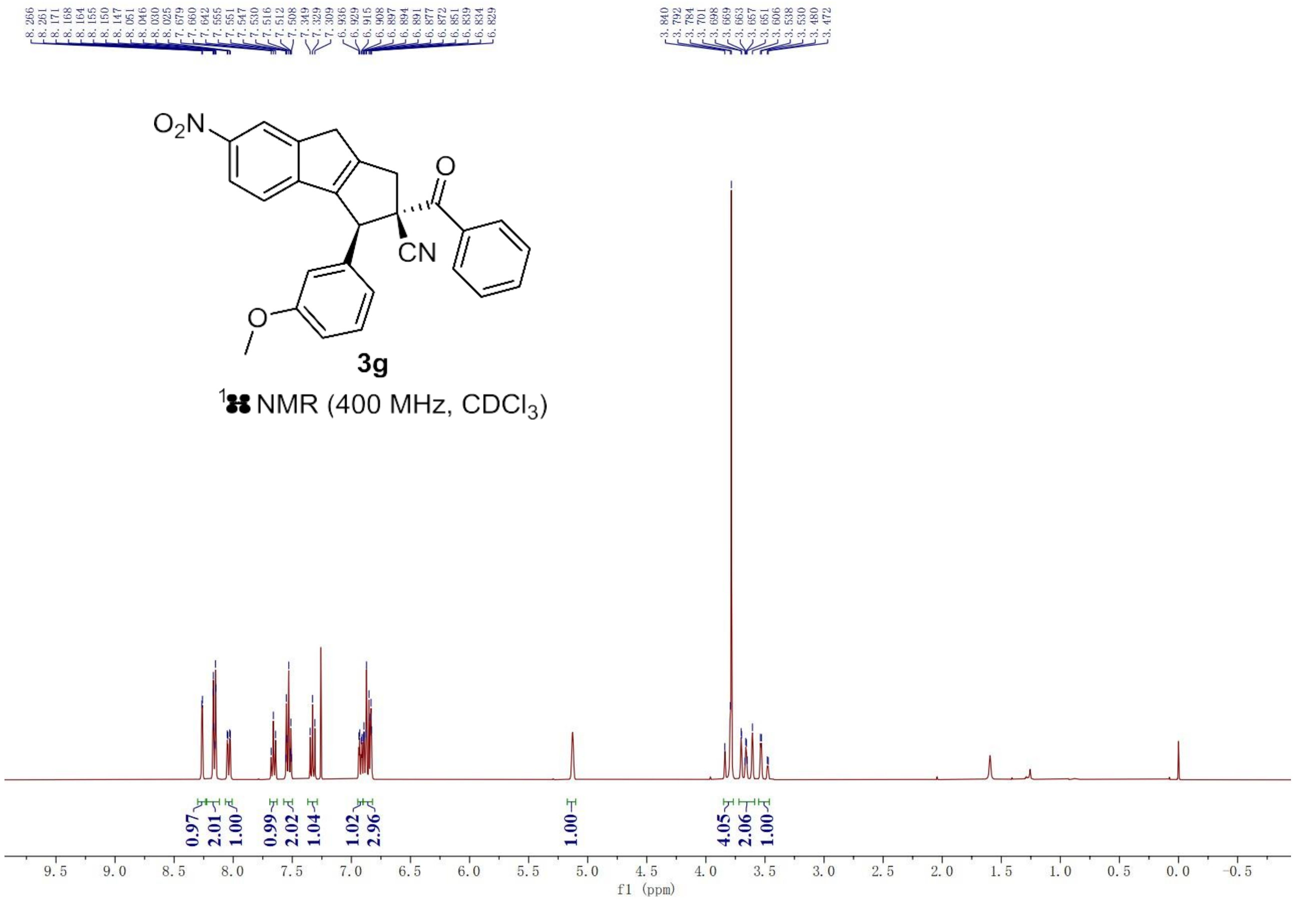
8.266 8.261 8.171 8.168 8.164 8.155 8.150 8.147 8.051 8.046 8.030 8.025 7.679 7.660 7.642 7.555 7.551 7.547 7.530 7.516 7.512 7.508 7.349 7.329 7.309 6.936 6.929 6.915 6.908 6.897 6.894 6.891 6.877 6.872 6.851 6.839 6.834 6.829
3.840 3.792 3.784 3.701 3.698 3.669 3.663 3.657 3.651 3.606 3.538 3.530 3.480 3.472
O2N
O
CN
O
3g
NMR (400 MHz, CDCl3)
0.97 2.01 1.00 0.99 2.02 1.04 1.02 2.96 1.00 4.05 2.06 1.00
9.5 9.0 8.5 8.0 7.5 7.0 6.5 6.0 5.5 5.0 4.5 4.0 3.5 3.0 2.5 2.0 1.5 1.0 0.5 0.0 -0.5
f1 (ppm)

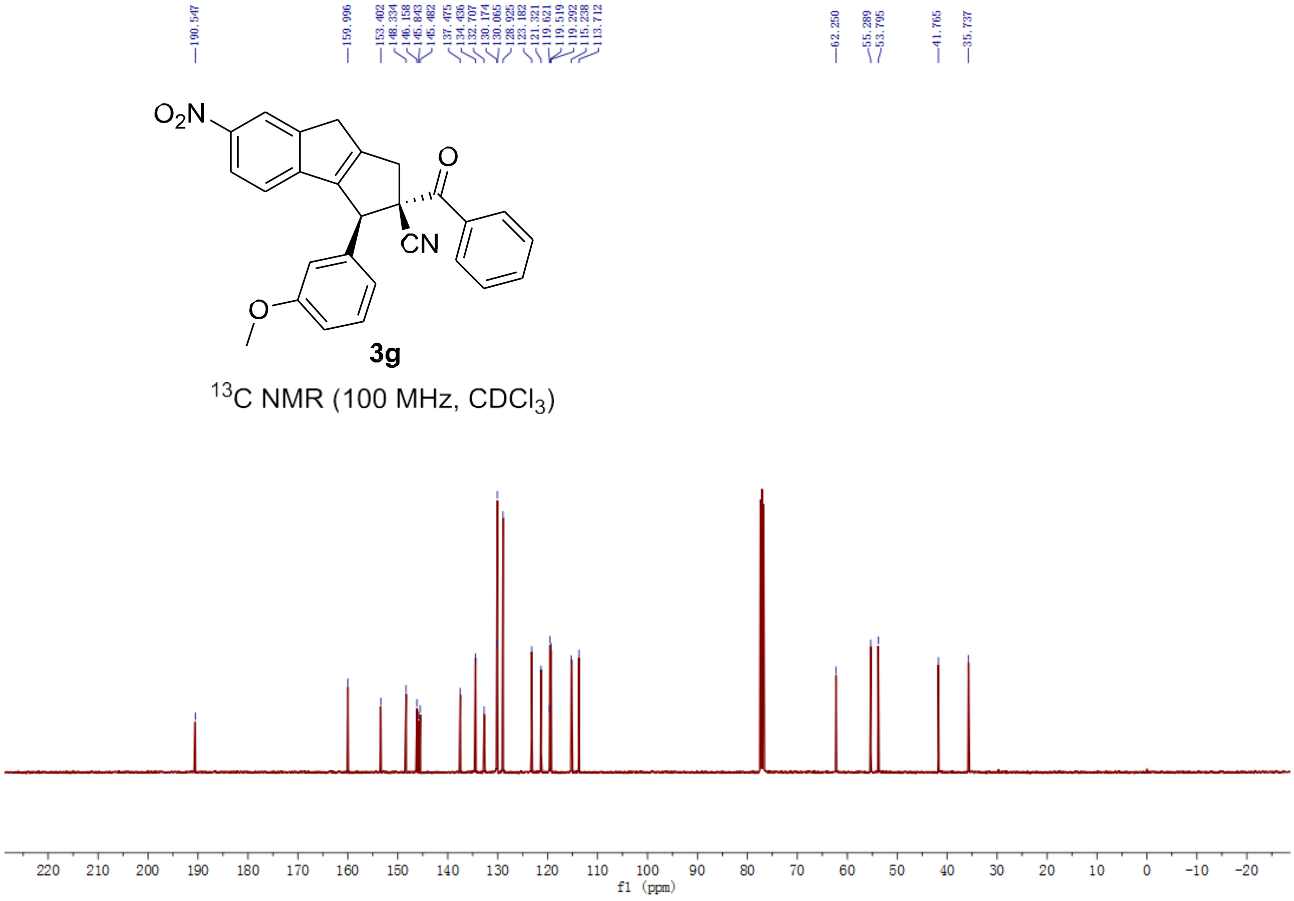
190.547
159.996 153.402 148.334 146.158 145.843 145.482 137.475 134.436 132.707 130.174 130.065 128.925 123.182 121.321 119.621 119.519 119.292 115.238 113.712
62.250 55.289 53.795 41.765 35.737
O2N
O
CN
O
3g
13C NMR (100 MHz, CDCl3)
220 210 200 190 180 170 160 150 140 130 120 110 100 90 80 70 60 50 40 30 20 10 0 -10 -20
f1 (ppm)

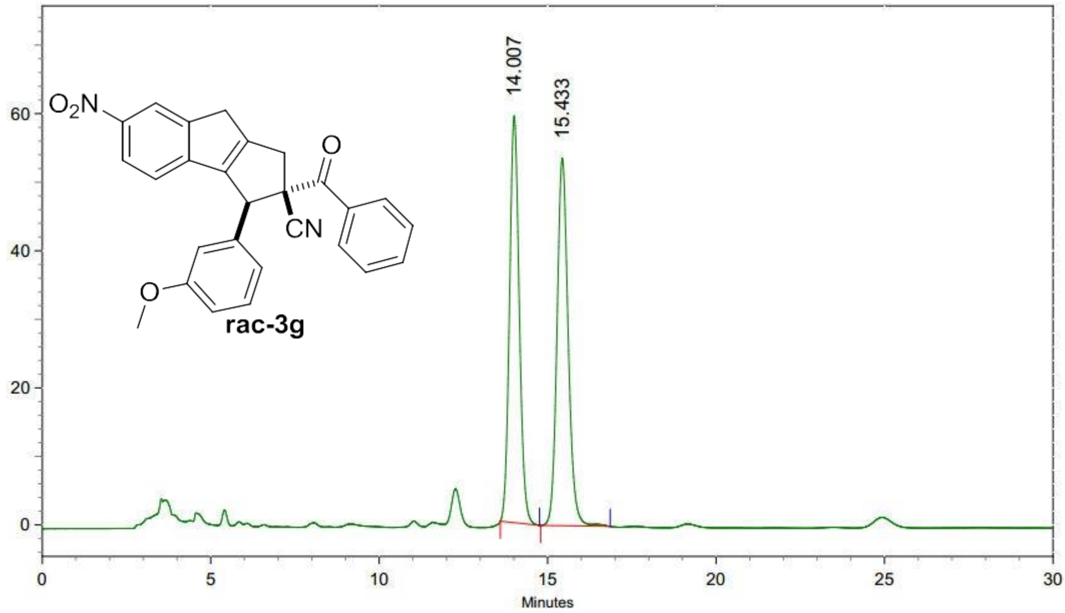

AREA PERCENT REPORT

Peak No.	Ret Time	Width	Height	Area	Area [%]
1	14.007	1.160	995546	19941547	49.0340
2	15.433	2.063	899527	20727274	50.9660

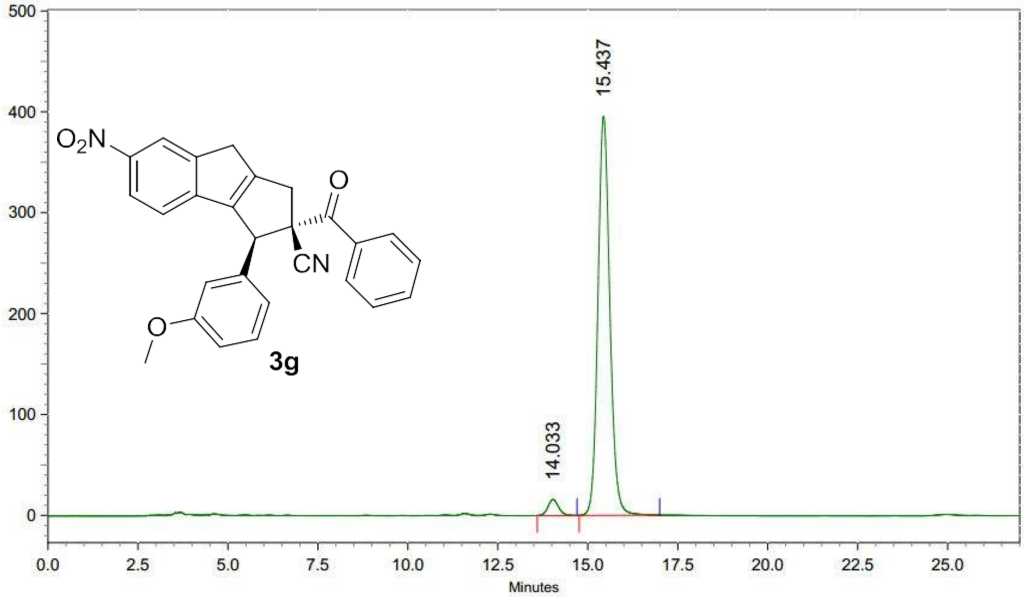

AREA PERCENT REPORT

Peak No.	Ret Time	Width	Height	Area	Area [%]
1	14.033	1.103	265650	5415212	3.4250
2	15.437	2.237	6635977	152694927	96.5750

Spectrum Plot Report

Name	CYC-20191124-8	Rack Pos.		Instrument	Instrument 1	Operator	
Inj. Vol. (ul)	10	Plate Pos.		IRM Status	Success		
Data File	CYC-20191124-8.d	Method (Acq)	TOF.m	Comment		Acq. Time (Local)	12/5/2019 4:54:21 PM (UTC+08:00)

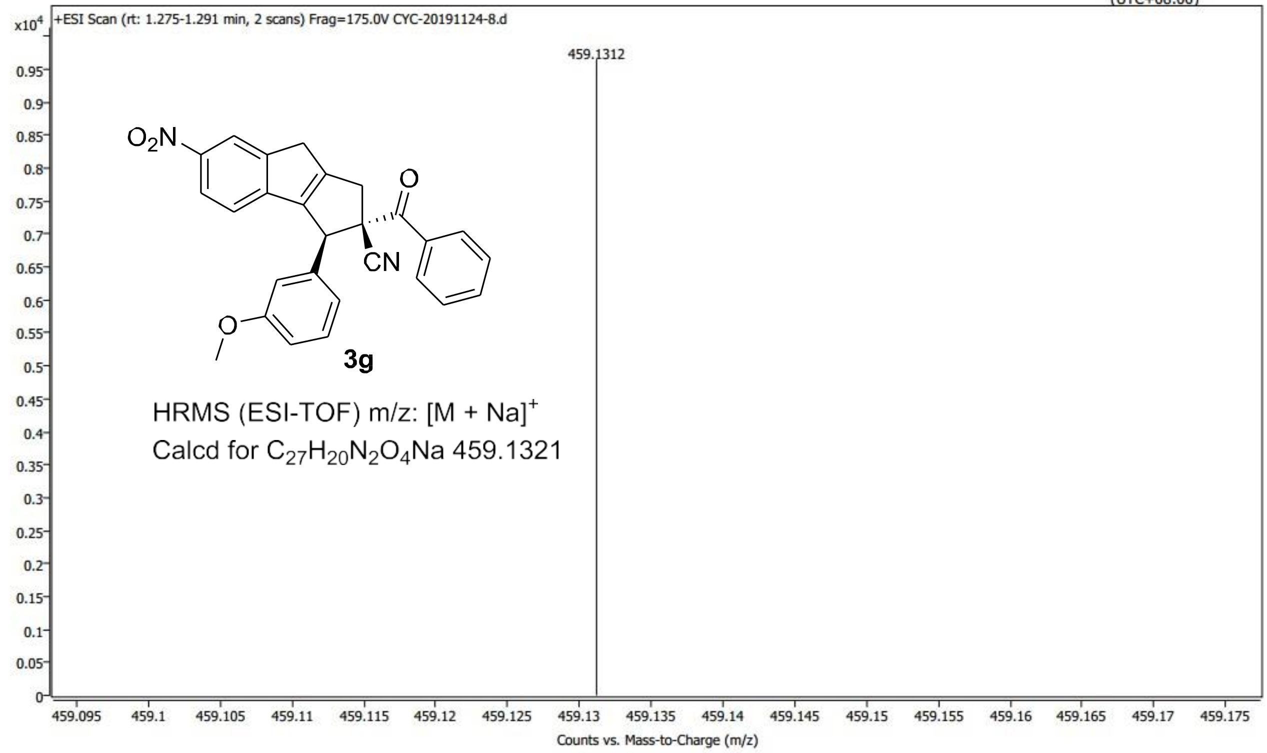

3g

HRMS (ESI-TOF) m/z: [M + Na]$^+$
Calcd for $C_{27}H_{20}N_2O_4Na$ 459.1321

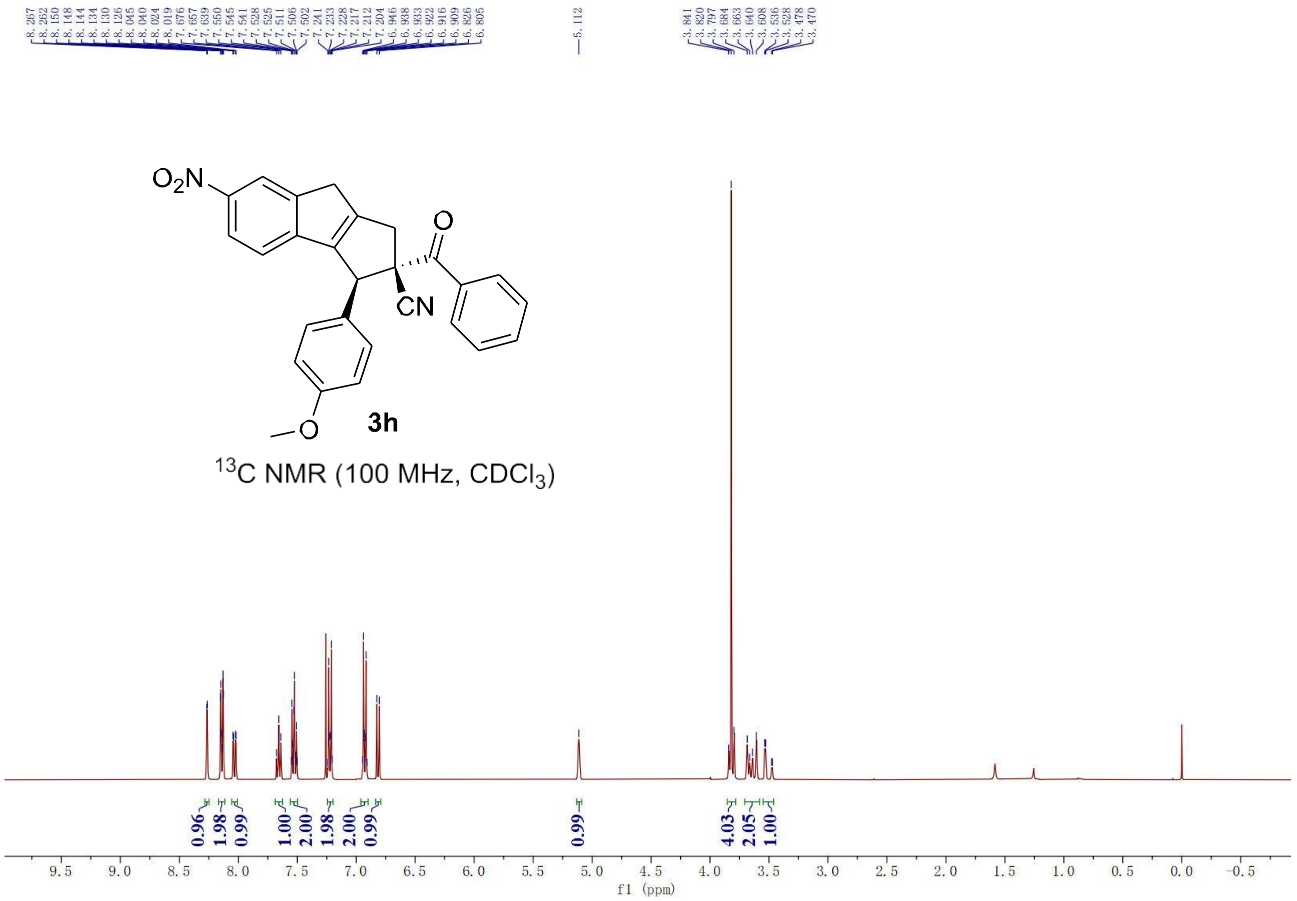

3h

^{13}C NMR (100 MHz, $CDCl_3$)

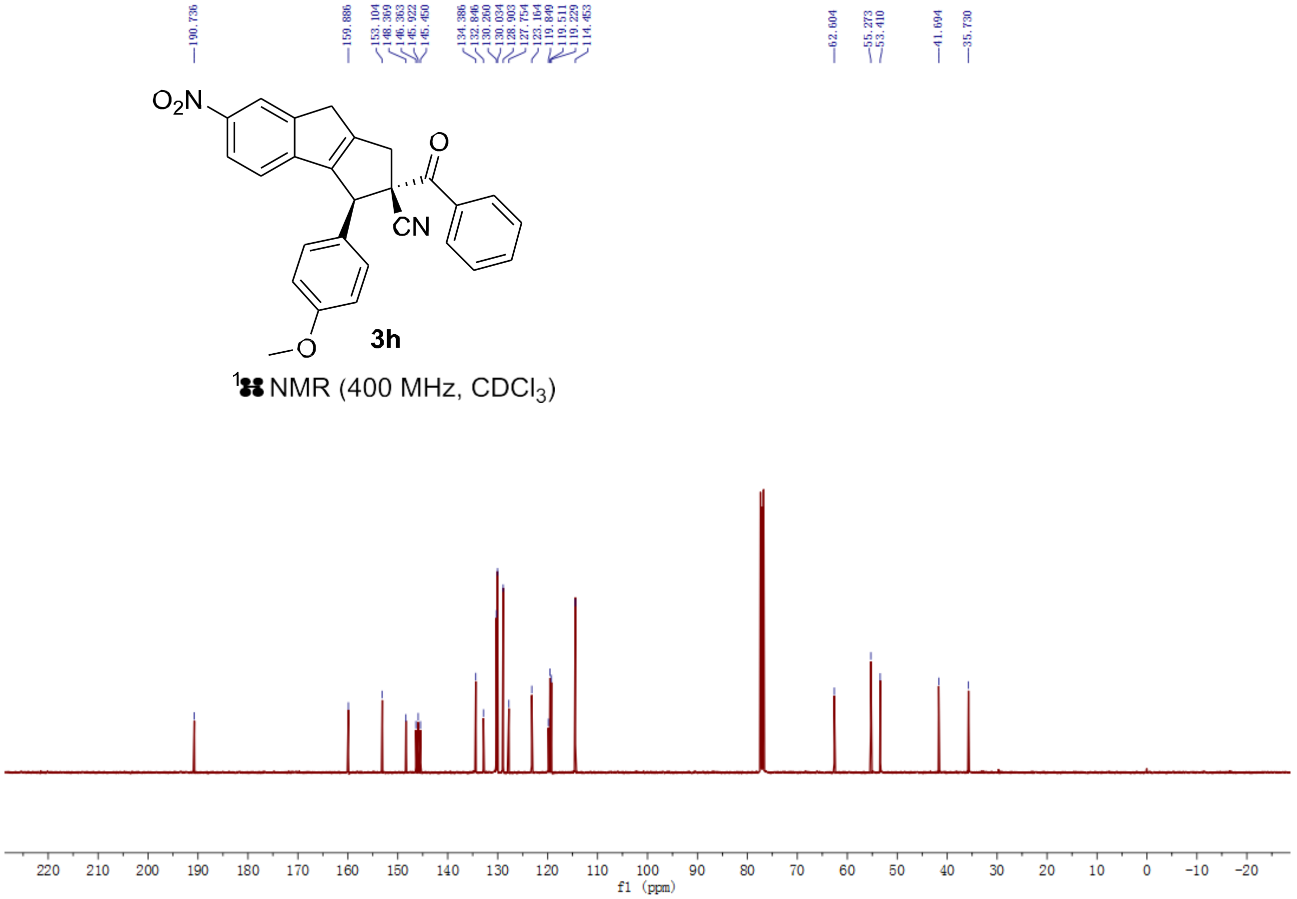

3h

^{1}[illegible] NMR (400 MHz, $CDCl_3$)

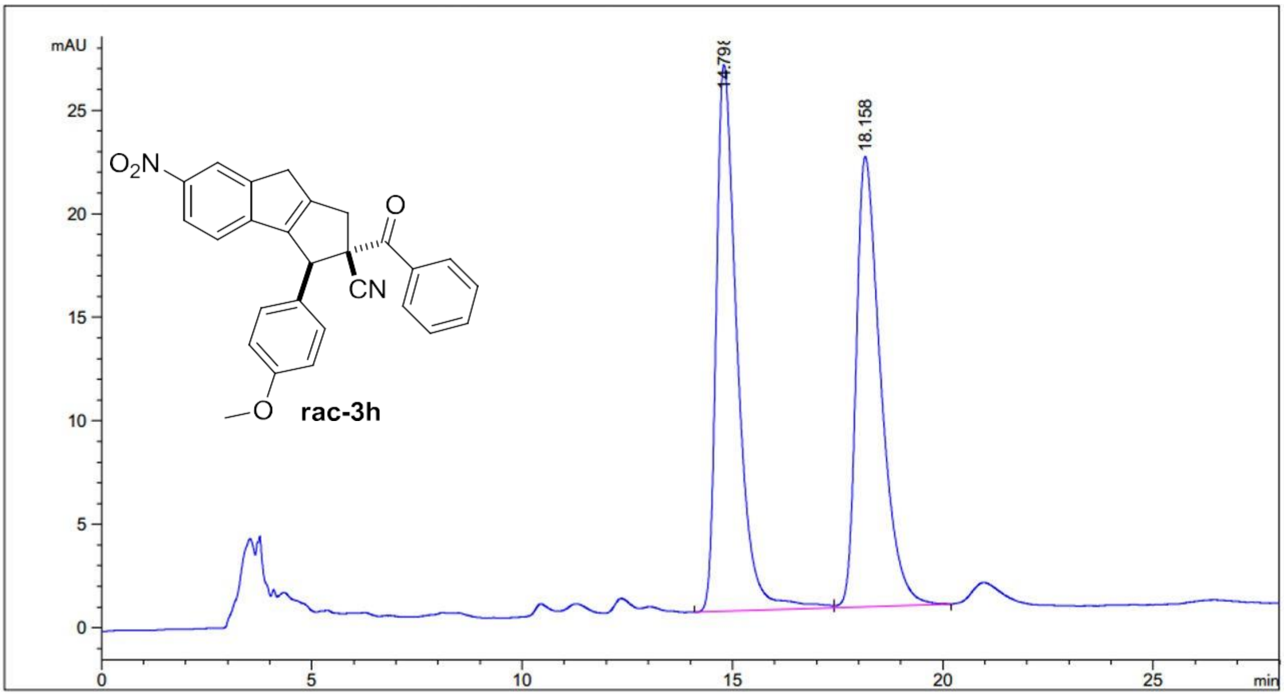

#	[min]		[min]	[mAU*s]	[mAU]	%
1	14.798	BV	0.5185	916.68500	26.41159	51.0173
2	18.158	VB	0.6032	880.12616	21.74638	48.9827

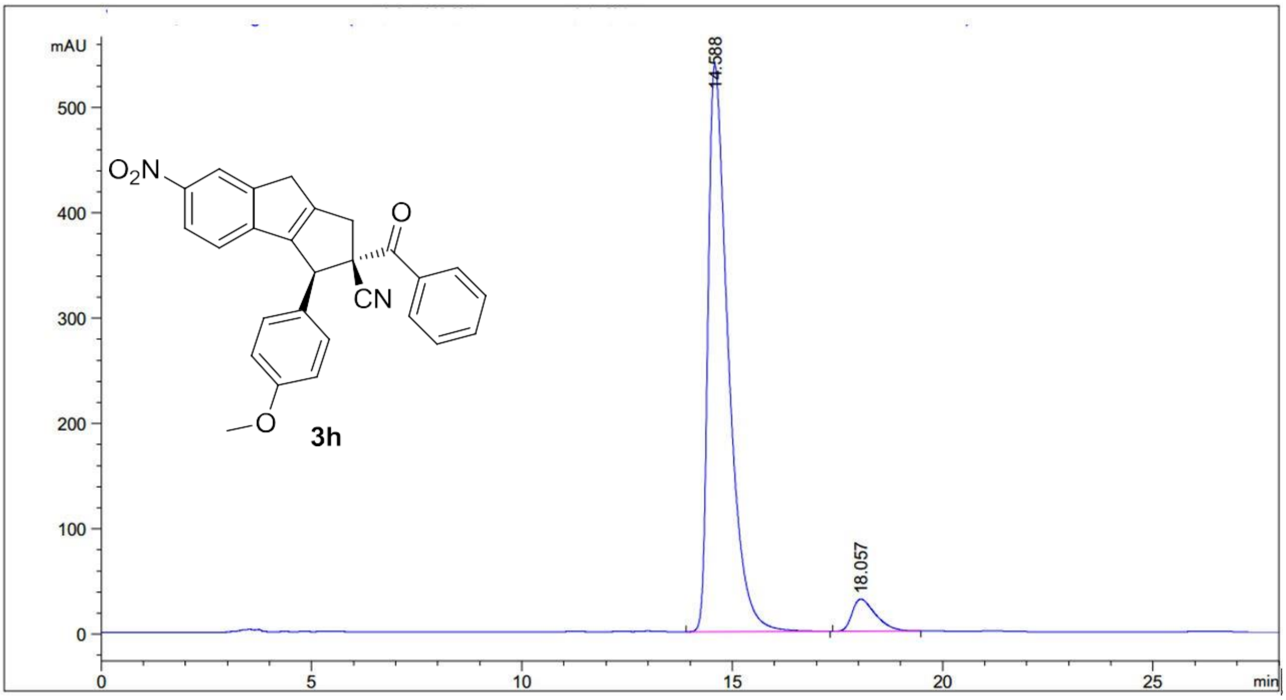

#	[min]		[min]	[mAU*s]	[mAU]	%
1	14.588	BB	0.4998	1.79586e4	538.46021	93.7801
2	18.057	BB	0.5876	1191.10168	30.62966	6.2199

Spectrum Plot Report

Name	CYC-20191018-60	Rack Pos.		Instrument	Instrument 1	Operator	
Inj. Vol. (ul)	10	Plate Pos.		IRM Status	Success		
Data File	CYC-20191018-60.d	Method (Acq)	TOF.m	Comment		Acq. Time (Local)	10/29/2019 3:55:22 PM (UTC+08:00)

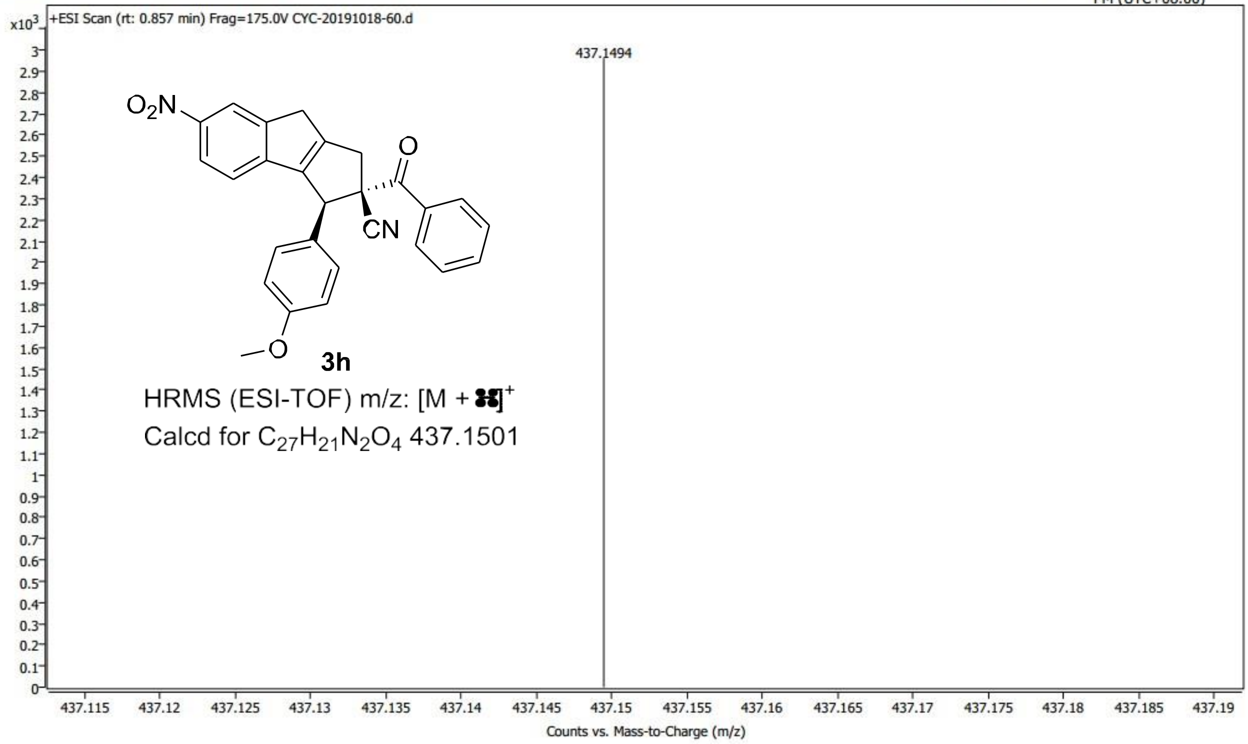

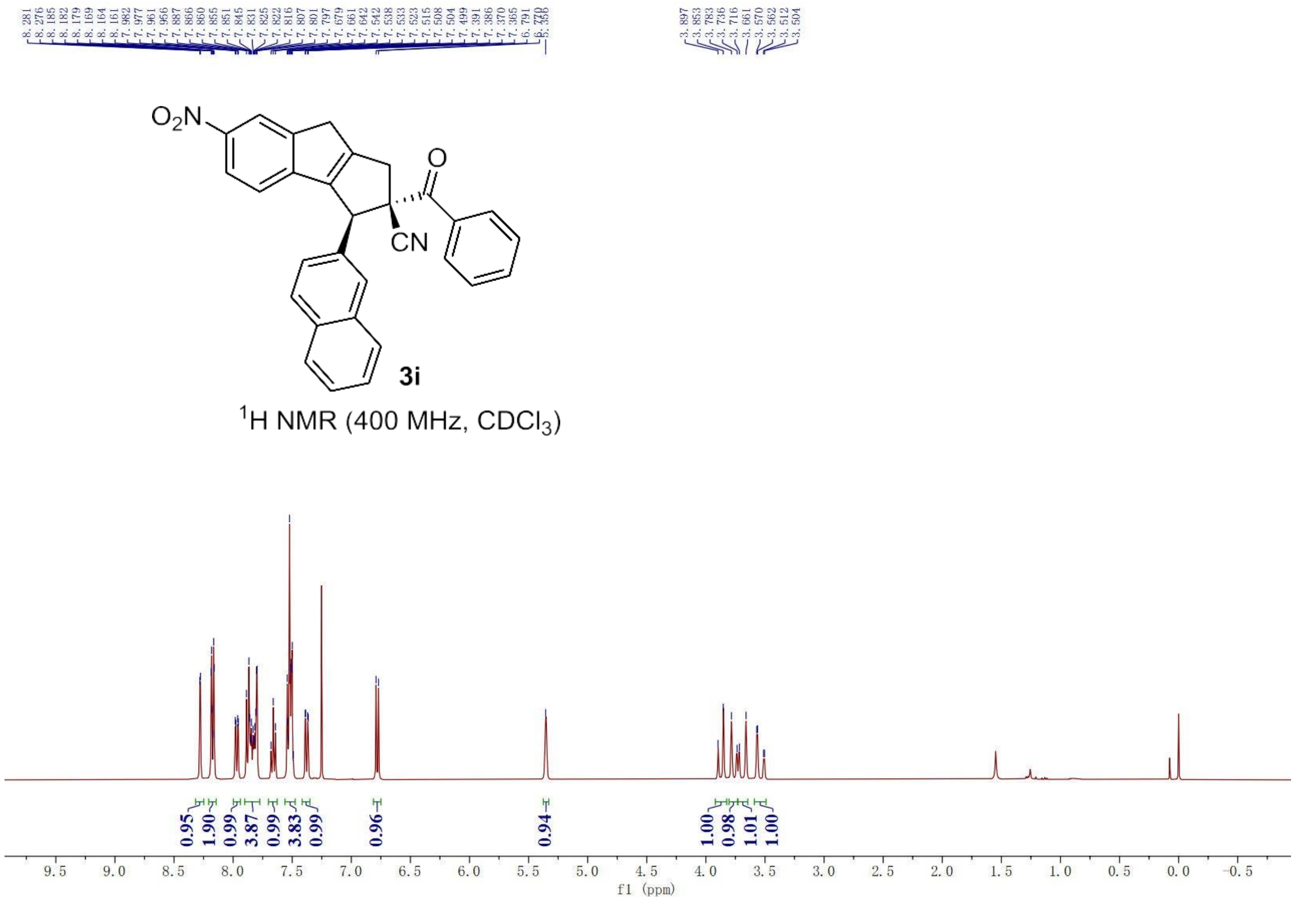
8.281 8.276 8.185 8.182 8.179 8.169 8.164 8.161 7.982 7.977 7.961 7.956 7.887 7.866 7.860 7.855 7.851 7.845 7.831 7.825 7.822 7.816 7.807 7.801 7.797 7.679 7.661 7.642 7.538 7.533 7.523 7.515 7.508 7.504 7.499 7.391 7.386 7.370 7.365 6.791 6.770 5.358
3.897 3.853 3.783 3.736 3.716 3.661 3.570 3.562 3.512 3.504
O_2N
CN
O
3i
^{1}H NMR (400 MHz, $CDCl_3$)
0.95 1.90 0.99 3.87 0.99 3.83 0.99 0.96 0.94 1.00 0.98 1.01 1.00
f1 (ppm)

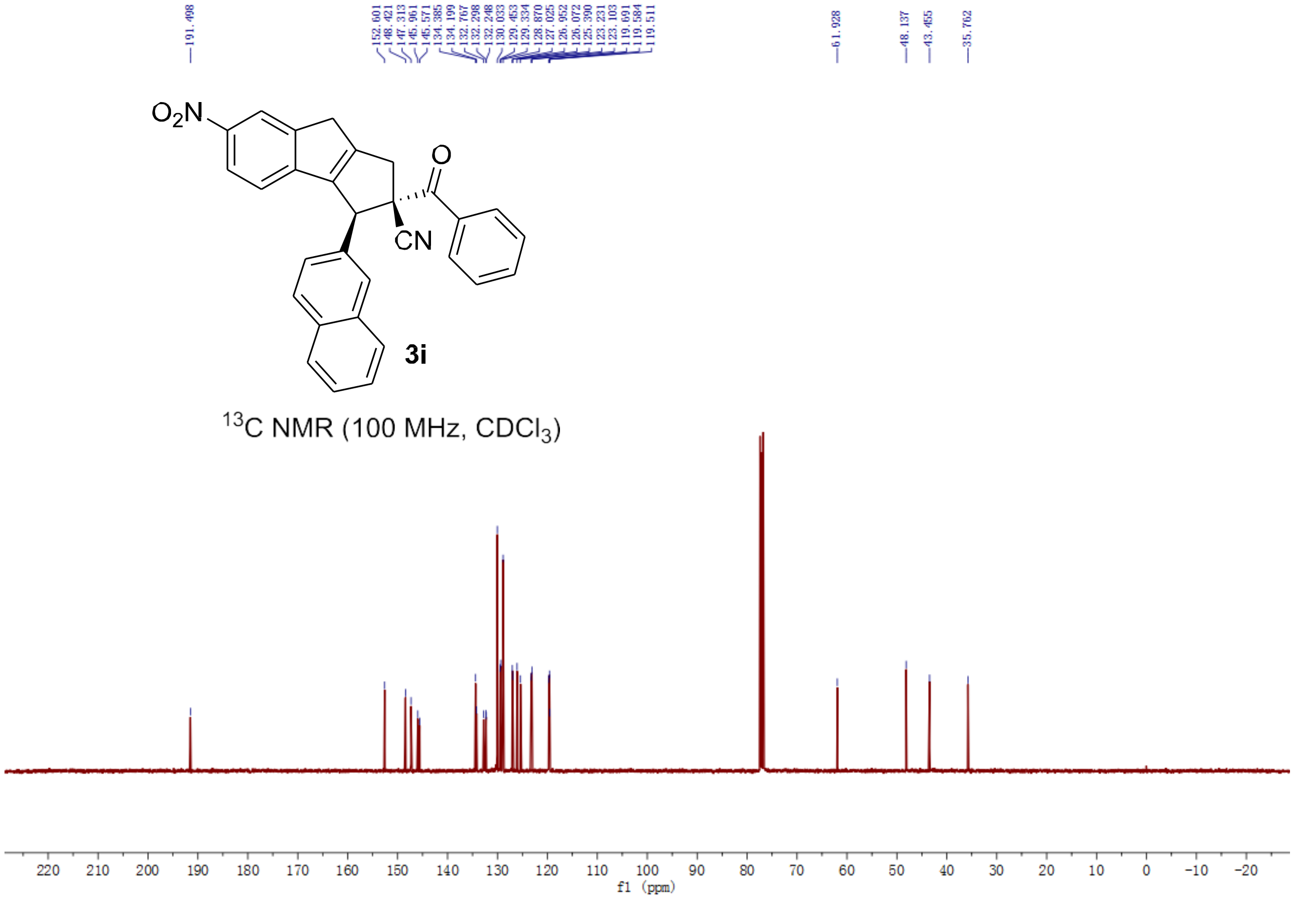
191.498
152.601 148.421 147.313 145.961 145.571 134.385 134.199 132.767 132.298 132.248 130.033 129.453 129.334 128.870 127.025 126.952 126.072 125.390 123.231 123.103 119.691 119.584 119.511
61.928
48.137 43.455 35.762
O_2N
CN
O
3i
^{13}C NMR (100 MHz, $CDCl_3$)
f1 (ppm)

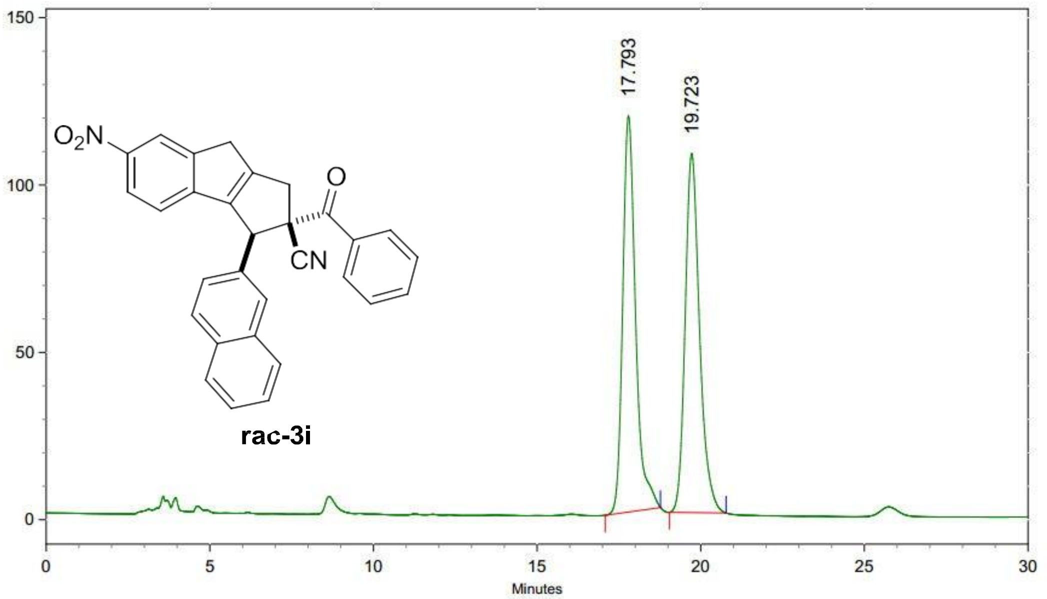

AREA PERCENT REPORT

Peak No.	Ret Time	Width	Height	Area	Area [%]
1	17.793	1.690	1985813	54130810	49.5316
2	19.723	1.727	1800208	55154706	50.4684

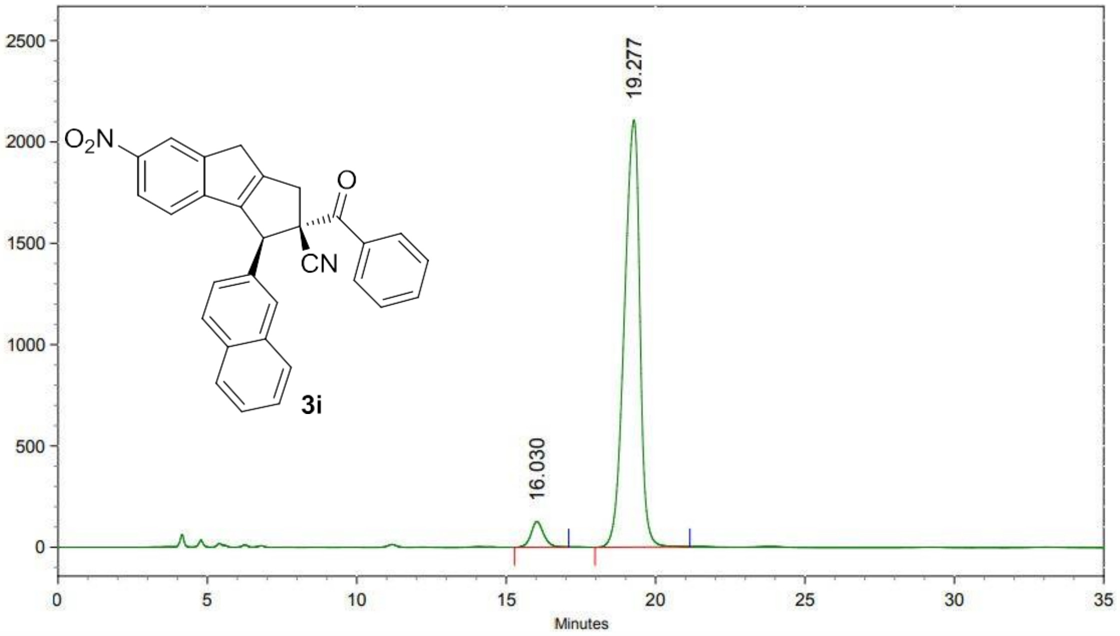

AREA PERCENT REPORT

Peak No.	Ret Time	Width	Height	Area	Area [%]
1	16.030	1.817	2130800	62356236	4.6631
2	19.277	3.173	35341117	1274867679	95.3369

Spectrum Plot Report

Agilent

Name	CYC-20191018-67	Rack Pos.		Instrument	Instrument 1	Operator	
Inj. Vol. (ul)	10	Plate Pos.		IRM Status	Success		
Data File	CYC-20191018-67.d	Method (Acq)	TOF.m	Comment		Acq. Time (Local)	10/29/2019 4:16:28 PM (UTC+08:00)

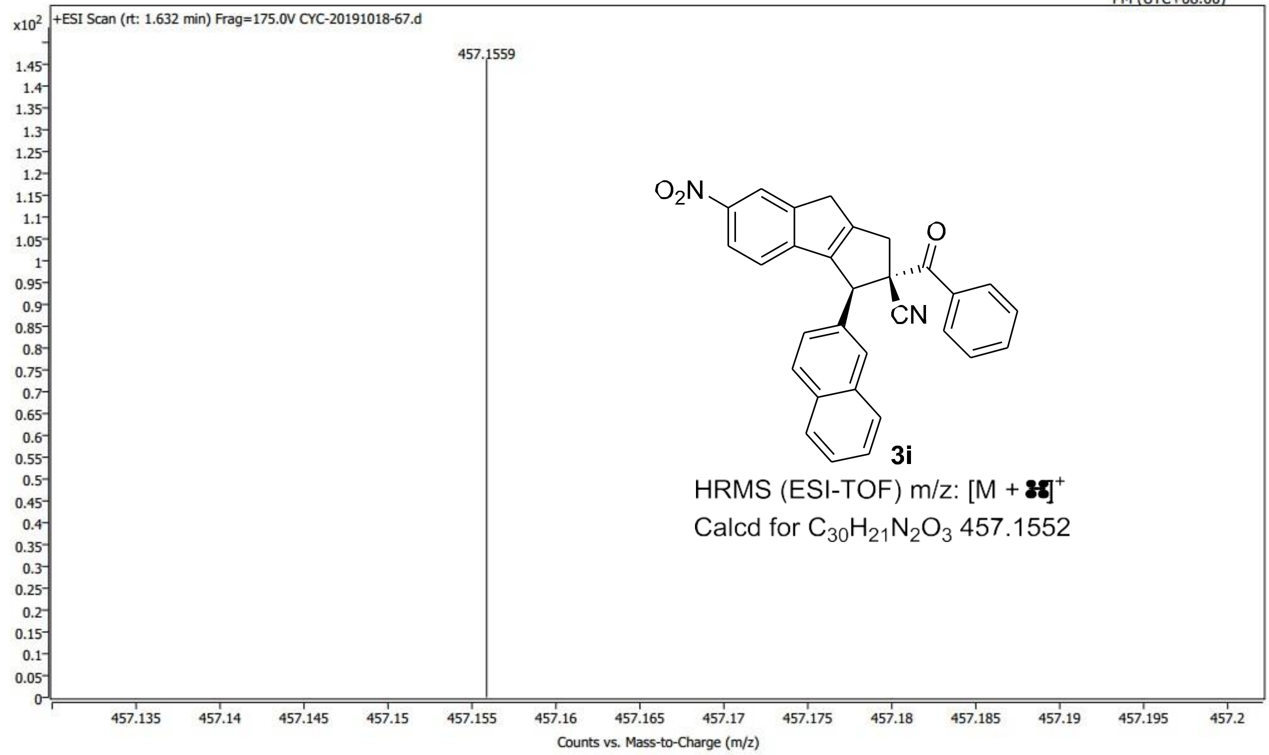

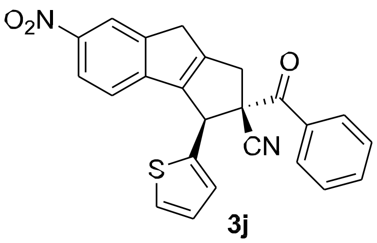

3j

^{1}H NMR (400 MHz, $CDCl_3$)

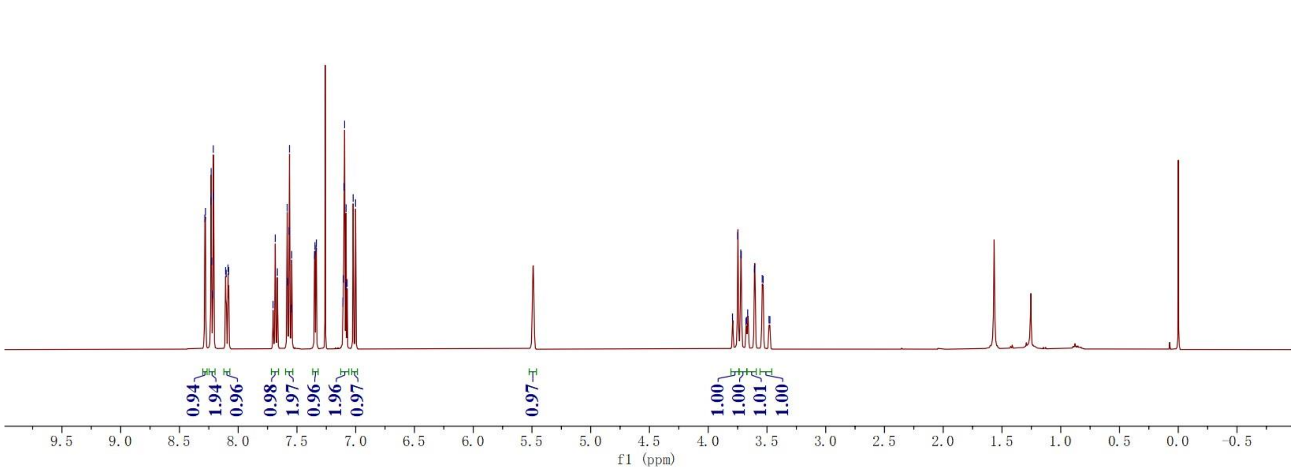

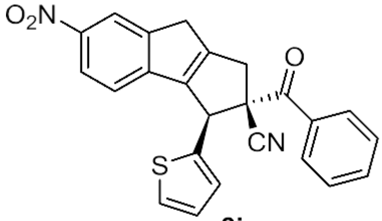

3j

^{13}C NMR (100 MHz, $CDCl_3$)

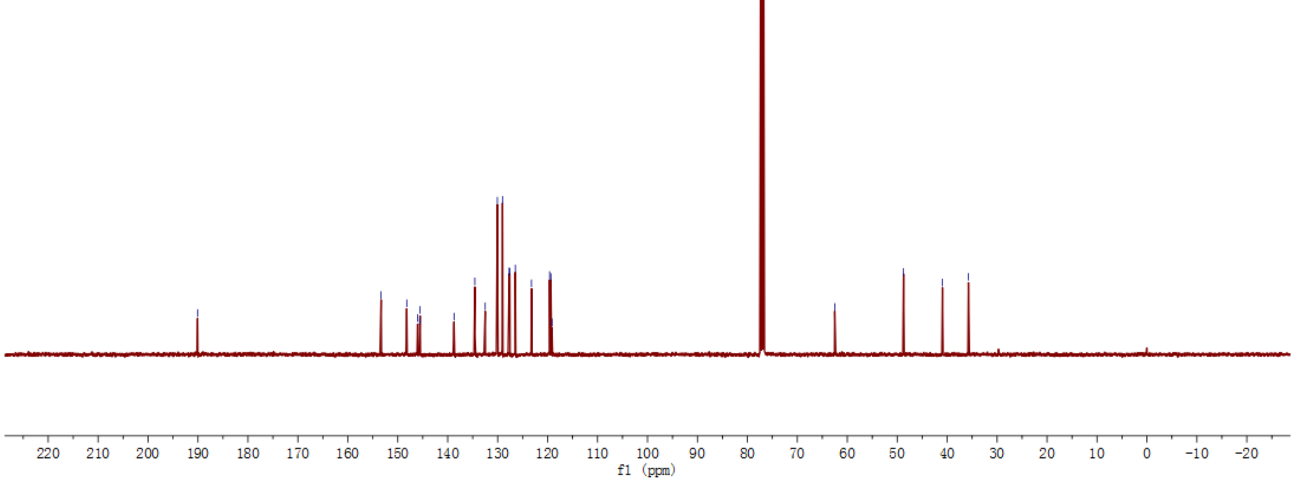

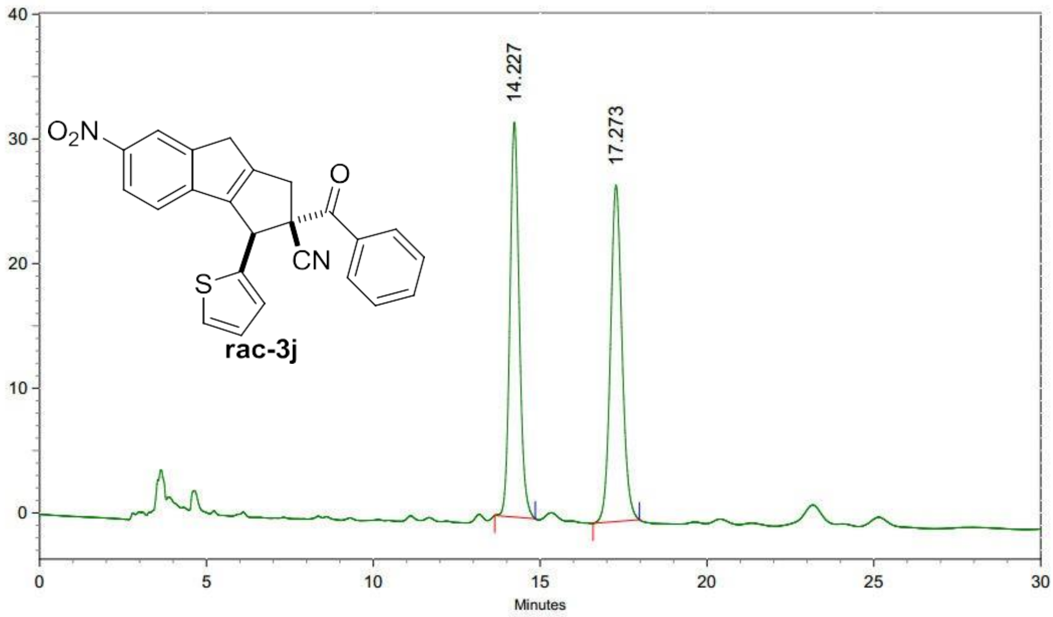

AREA PERCENT REPORT

Peak No.	Ret Time	Width	Height	Area	Area [%]
1	14.227	1.213	531413	10359157	48.7273
2	17.273	1.393	452753	10900289	51.2727

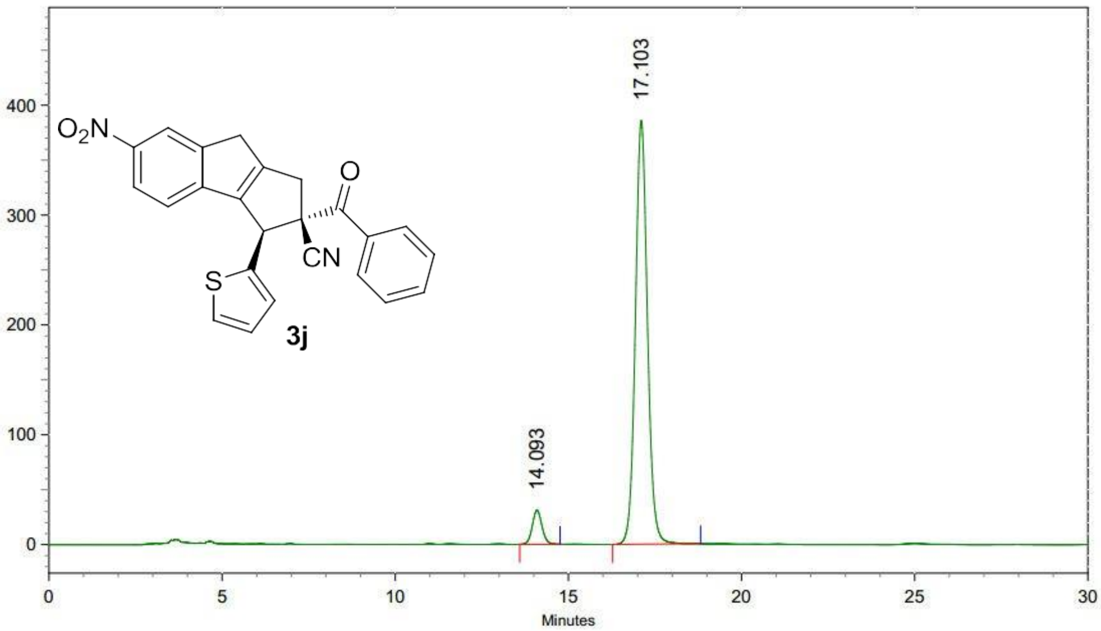

AREA PERCENT REPORT

Peak No.	Ret Time	Width	Height	Area	Area [%]
1	14.093	1.163	521715	10180607	6.1114
2	17.103	2.537	6470737	156403300	93.8886

Spectrum Plot Report

Name	CYC-20191025-13	Rack Pos.		Instrument	Instrument 1	Operator	
Inj. Vol. (ul)	10	Plate Pos.		IRM Status	Success		
Data File	CYC-20191025-13.d	Method (Acq)	TOF.m	Comment		Acq. Time (Local)	11/9/2019 11:16:18 AM (UTC+08:00)

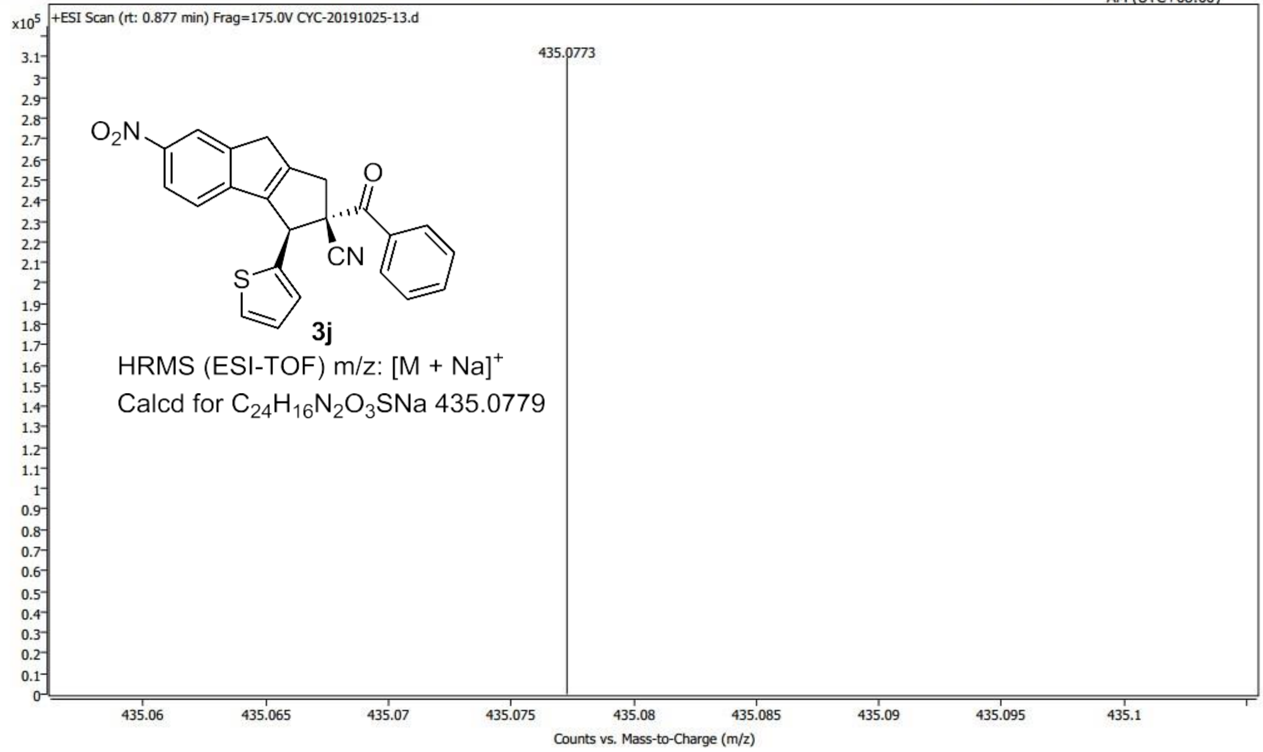

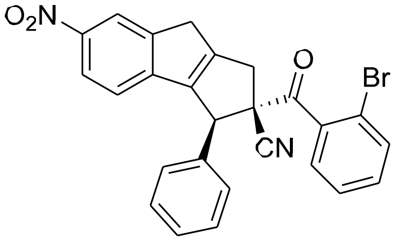

3k

^{1}H NMR (400 MHz, $CDCl_3$)

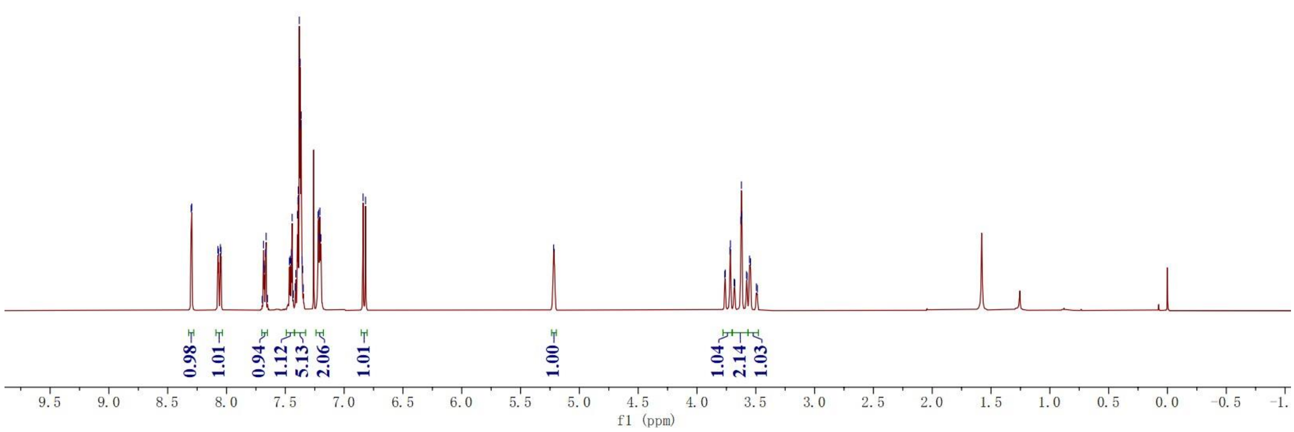

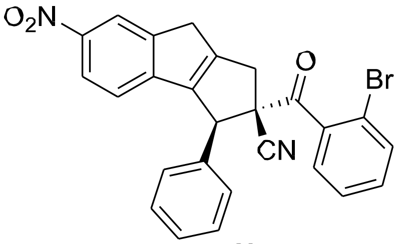

3k

^{13}C NMR (100 MHz, $CDCl_3$)

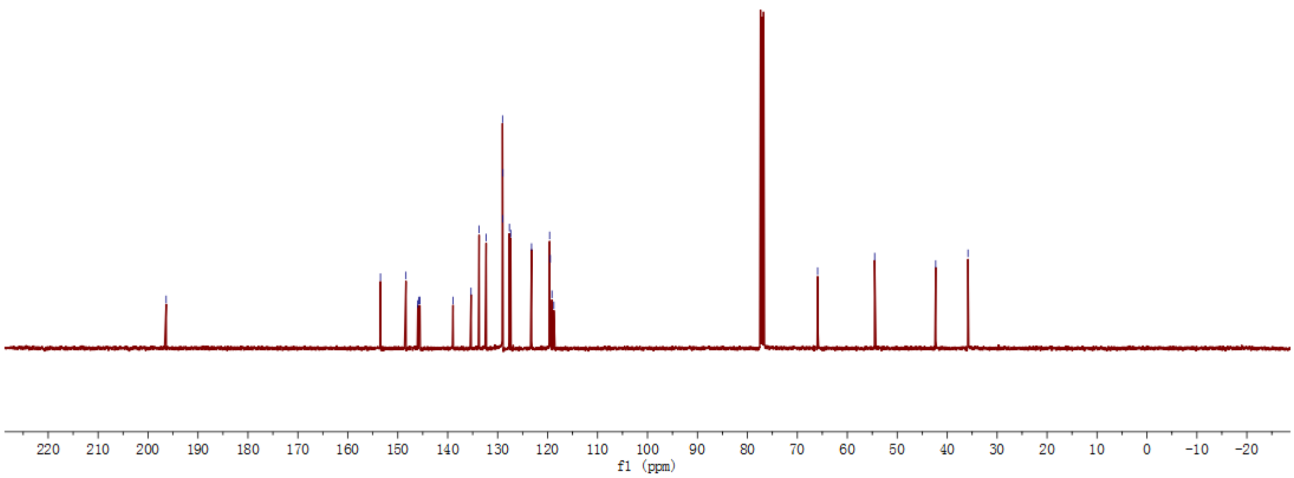

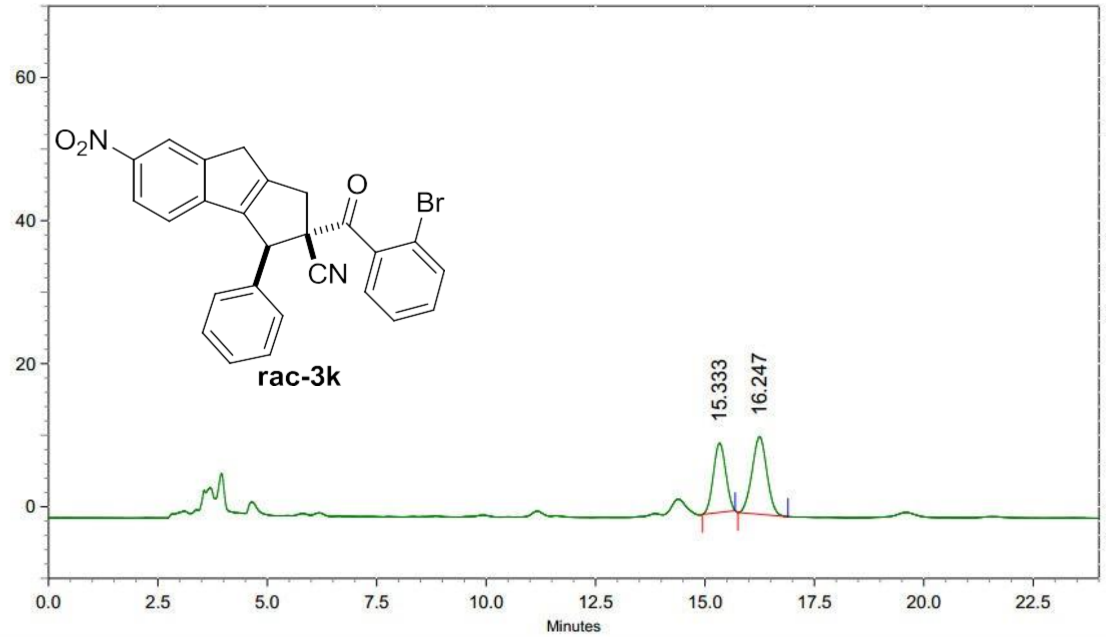

AREA PERCENT REPORT

Peak No.	Ret Time	Width	Height	Area	Area [%]
1	15.333	0.743	162762	3189159	42.6258
2	16.247	1.143	182088	4292591	57.3742

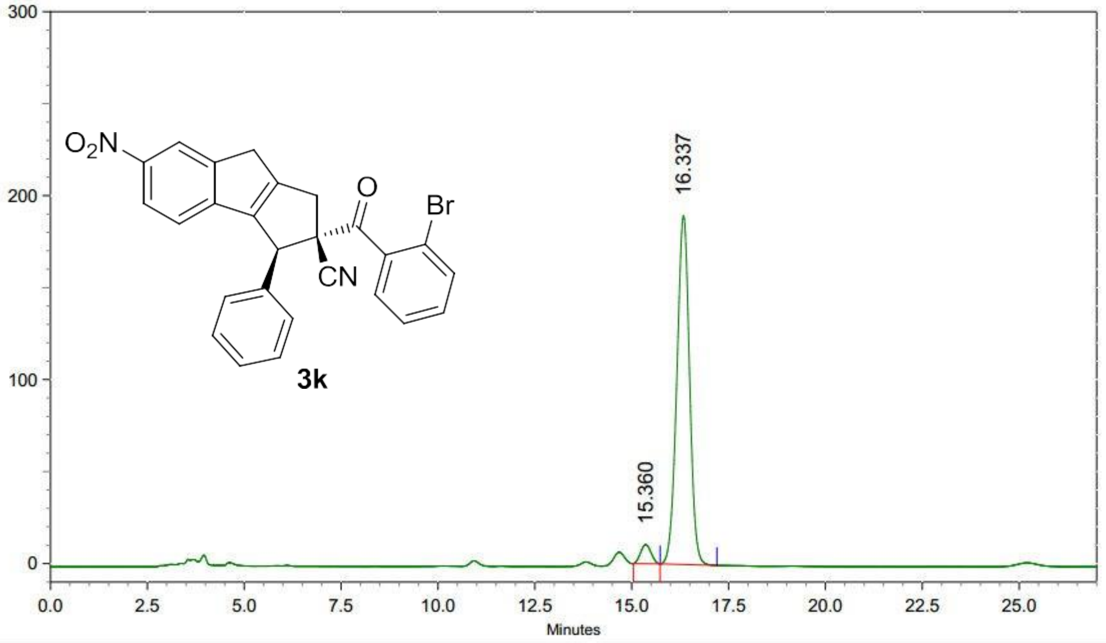

AREA PERCENT REPORT

Peak No.	Ret Time	Width	Height	Area	Area [%]
1	15.360	0.693	173993	3270873	4.3318
2	16.337	1.470	3182051	72237483	95.6682

Spectrum Plot Report

Name	CYC-20191124-4	Rack Pos.		Instrument	Instrument 1	Operator	
Inj. Vol. (ul)	10	Plate Pos.		IRM Status	Success		
Data File	CYC-20191124-4.d	Method (Acq)	TOF.m	Comment		Acq. Time (Local)	12/5/2019 4:42:18 PM (UTC+08:00)

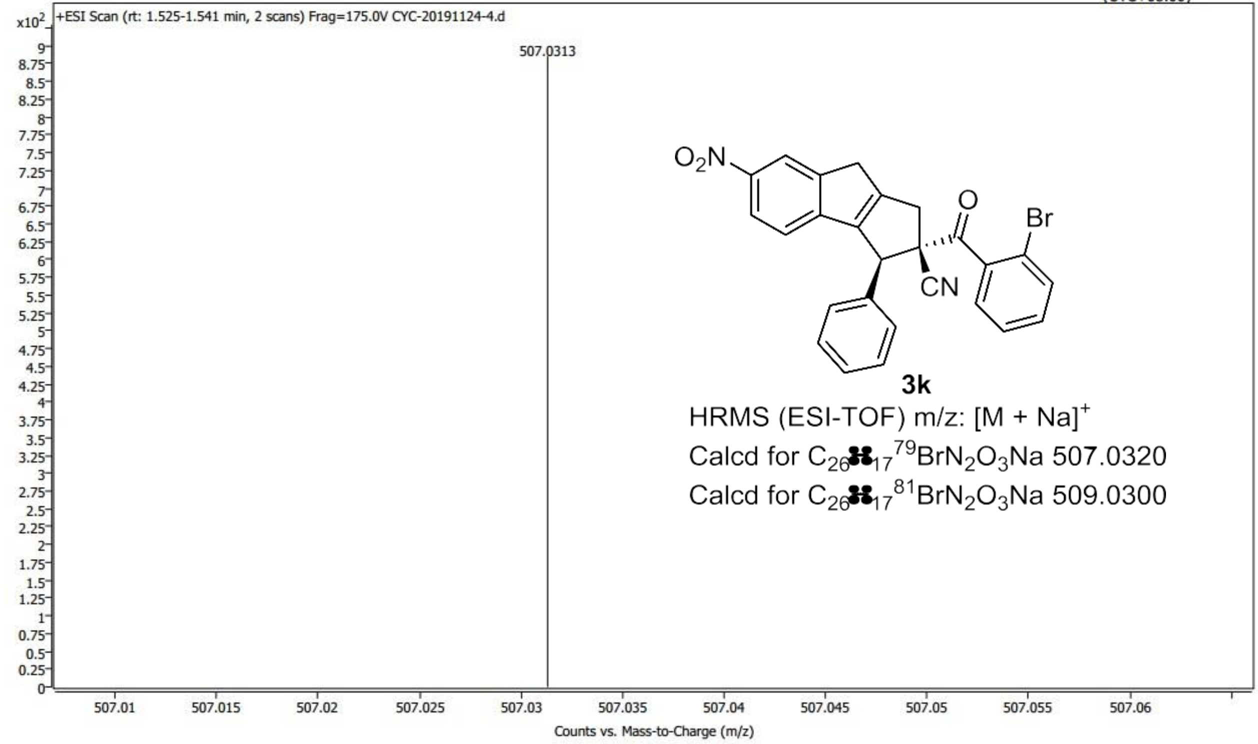

Spectrum Plot Report

Name	CYC-20191124-4	Rack Pos.		Instrument	Instrument 1	Operator	
Inj. Vol. (ul)	10	Plate Pos.		IRM Status	Success		
Data File	CYC-20191124-4.d	Method (Acq)	TOF.m	Comment		Acq. Time (Local)	12/5/2019 4:42:18 PM (UTC+08:00)

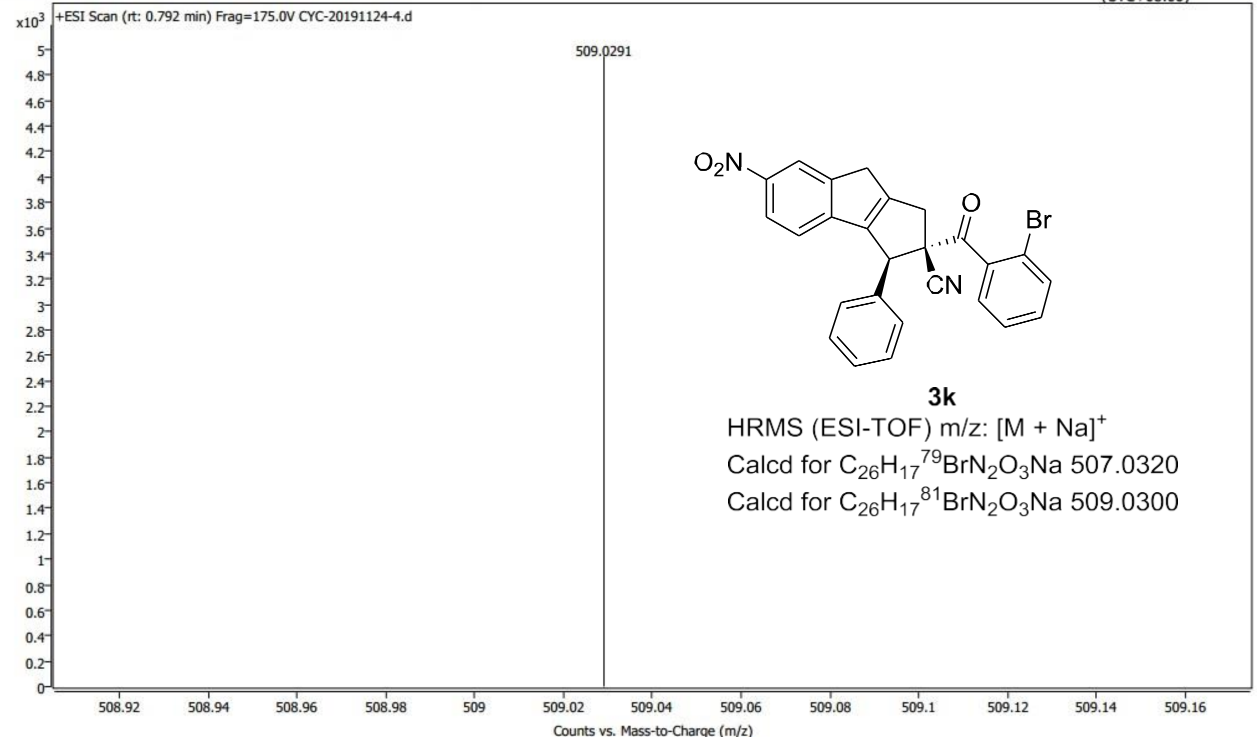

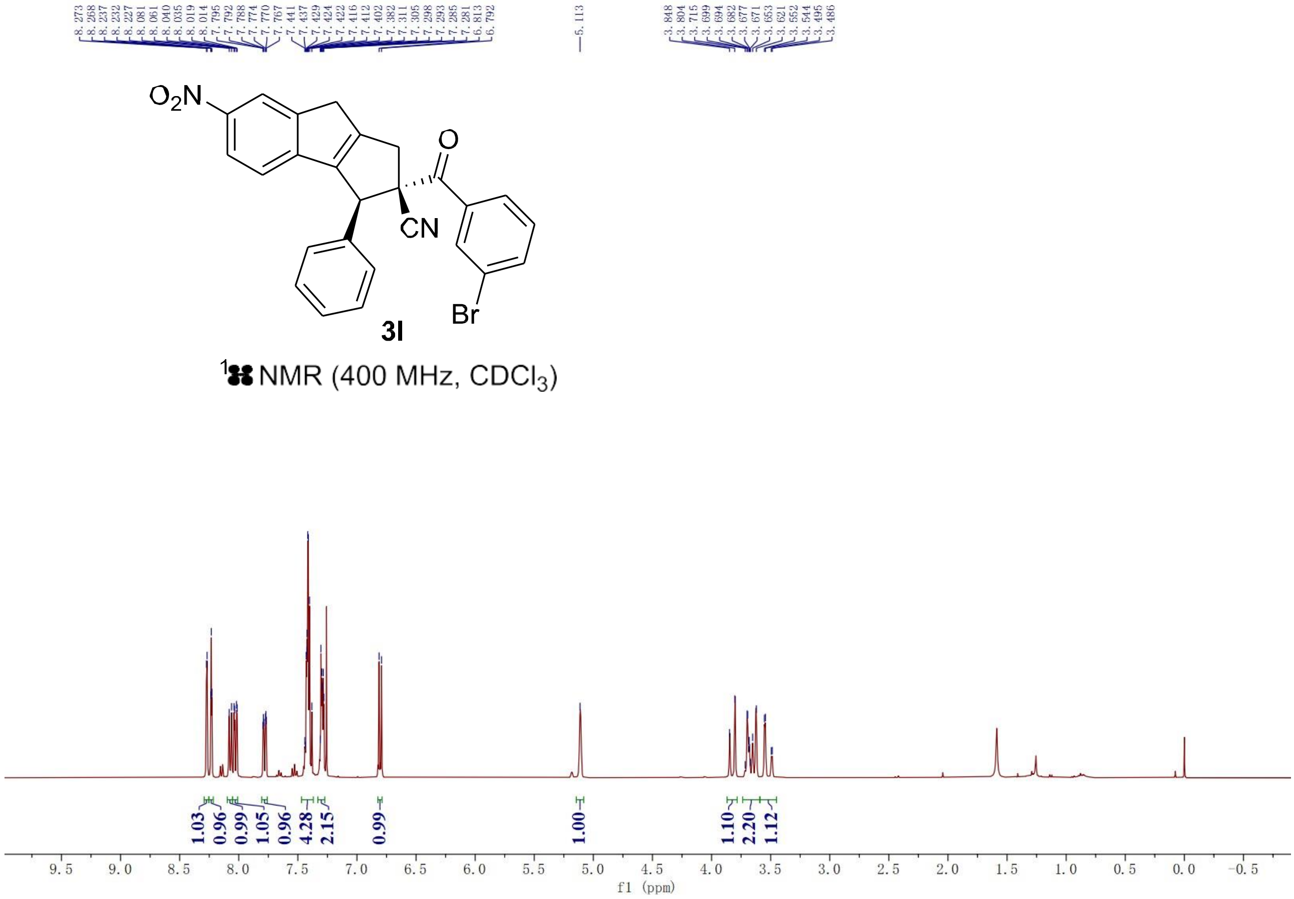

3l

[1]?? NMR (400 MHz, $CDCl_3$)

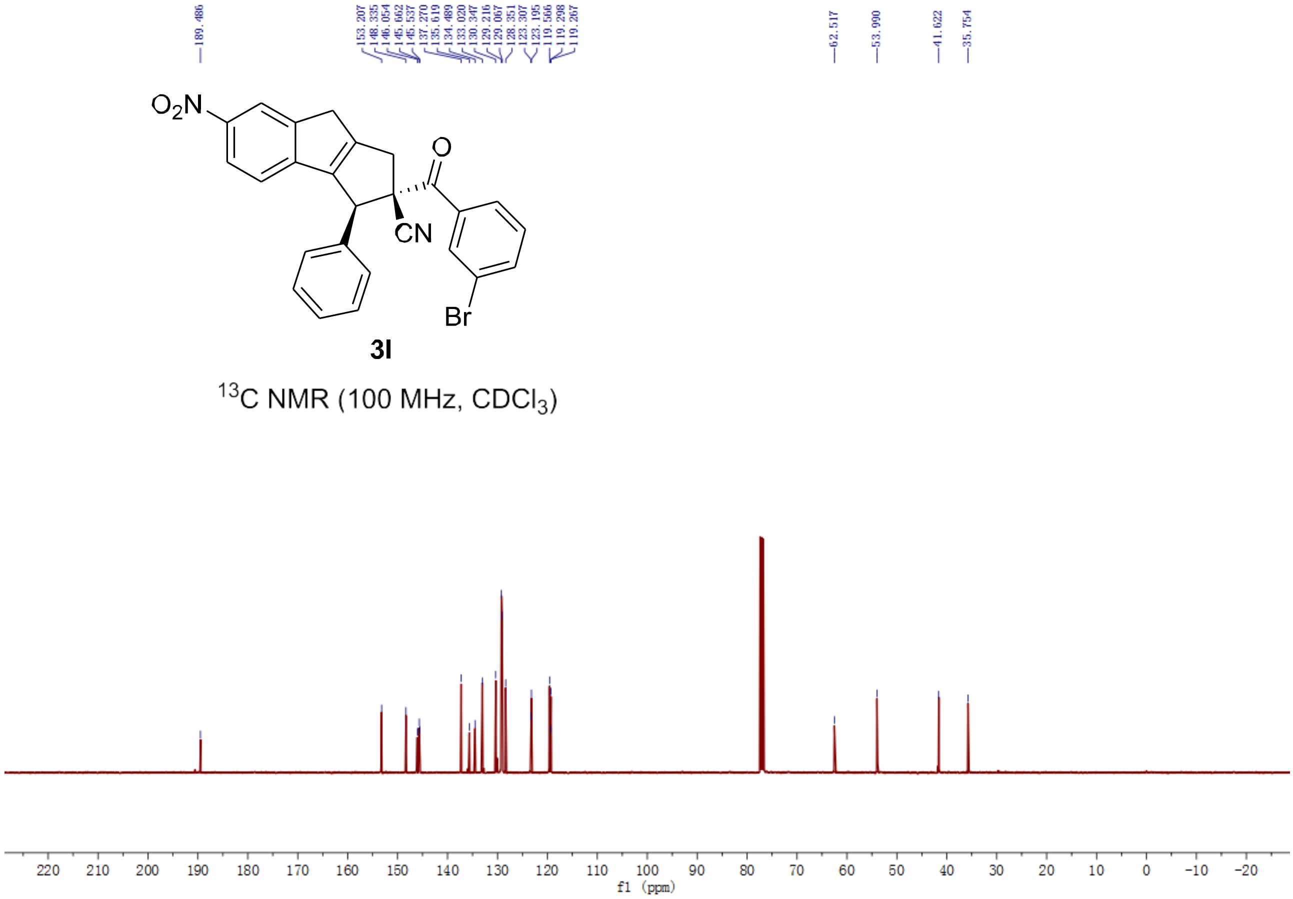

3l

^{13}C NMR (100 MHz, $CDCl_3$)

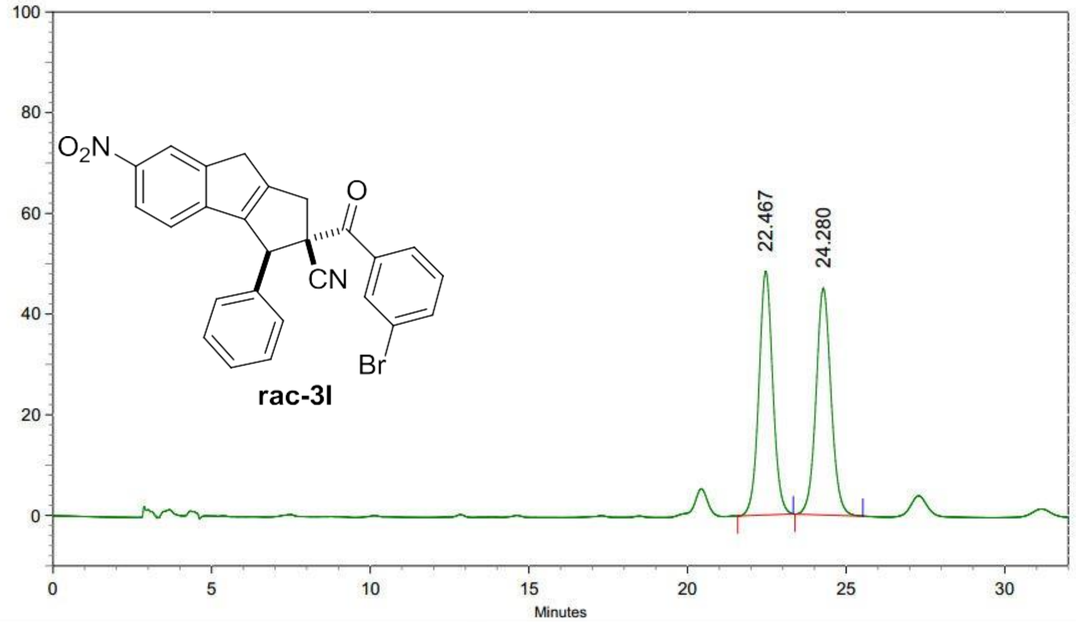

AREA PERCENT REPORT

Peak No.	Ret Time	Width	Height	Area	Area [%]
1	22.467	1.753	812614	24766805	49.8434
2	24.280	2.143	755892	24922454	50.1566

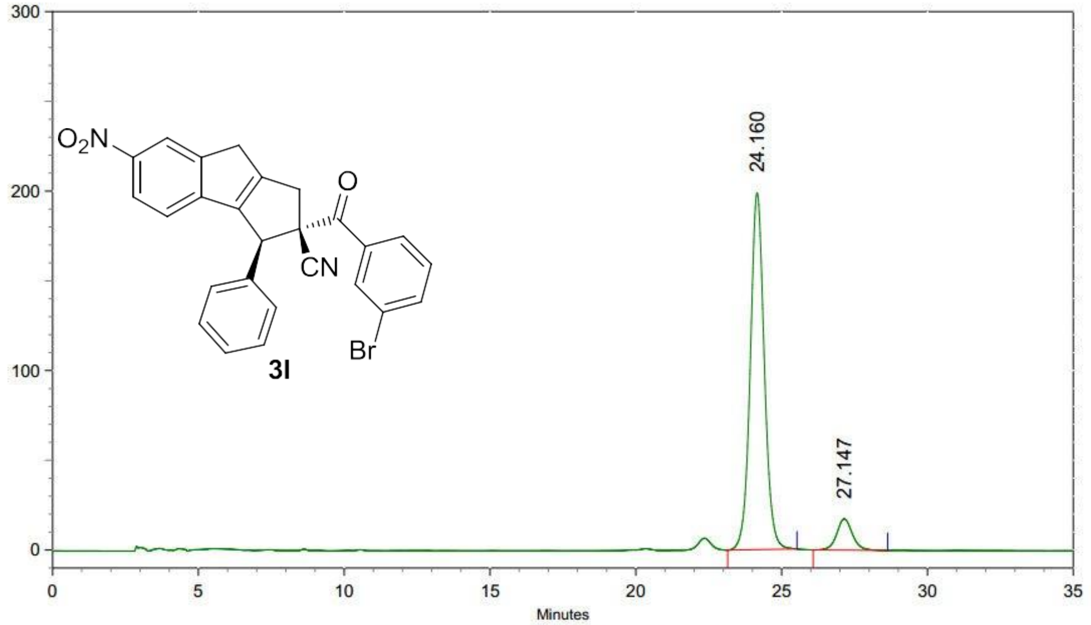

AREA PERCENT REPORT

Peak No.	Ret Time	Width	Height	Area	Area [%]
1	24.160	2.377	3335325	111500414	91.0943
2	27.147	2.557	292741	10900693	8.9057

Spectrum Plot Report

Name	CYC-20191103-11	Rack Pos.		Instrument	Instrument 1	Operator	
Inj. Vol. (ul)	10	Plate Pos.		IRM Status	Success		
Data File	CYC-20191103-11.d	Method (Acq)	TOF.m	Comment		Acq. Time (Local)	11/8/2019 10:50:35 AM (UTC+08:00)

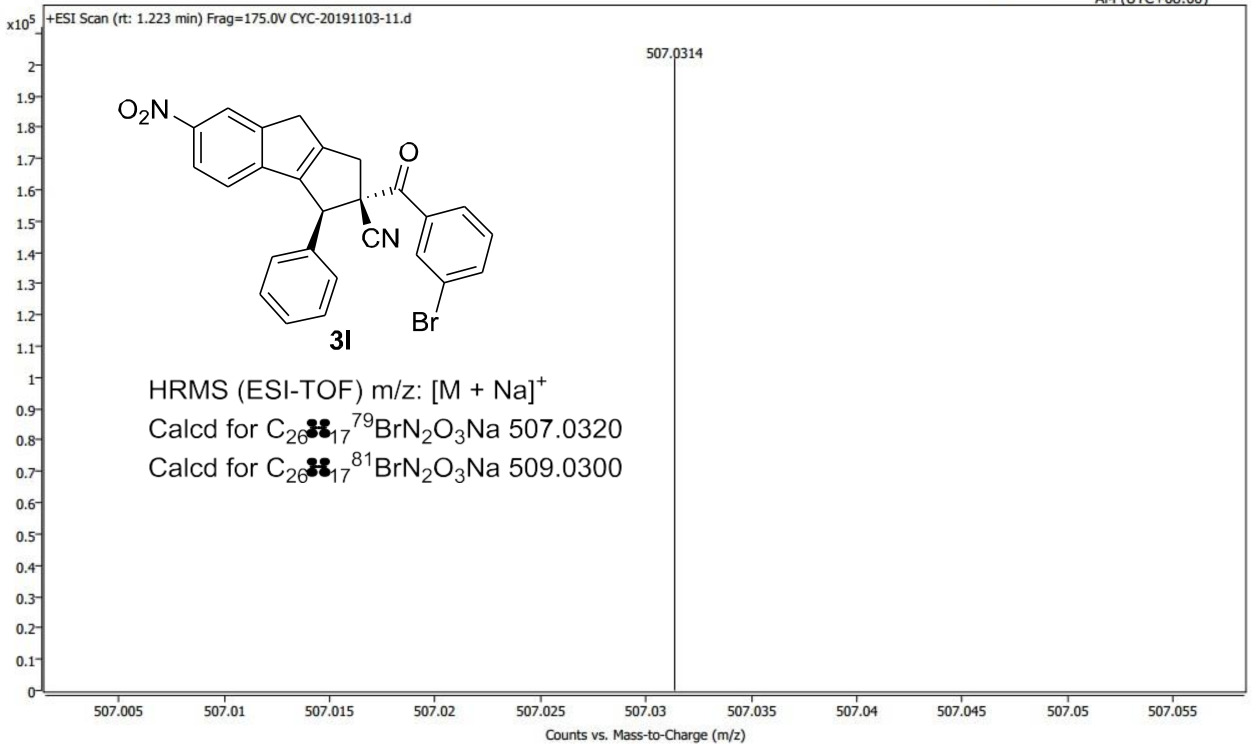

Spectrum Plot Report

Name	CYC-20191103-11	Rack Pos.		Instrument	Instrument 1	Operator	
Inj. Vol. (ul)	10	Plate Pos.		IRM Status	Success		
Data File	CYC-20191103-11.d	Method (Acq)	TOF.m	Comment		Acq. Time (Local)	11/8/2019 10:50:35 AM (UTC+08:00)

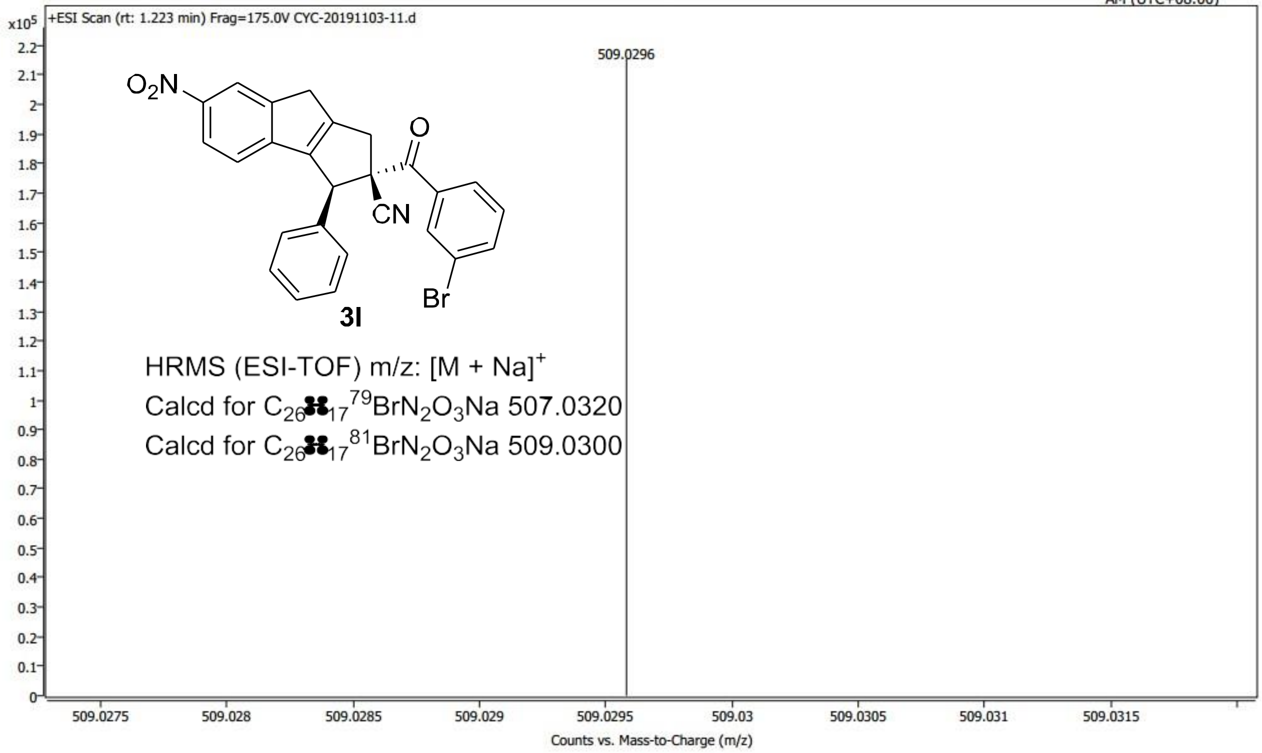

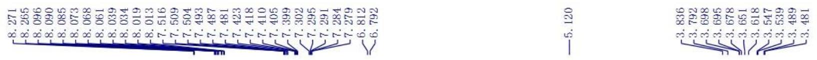

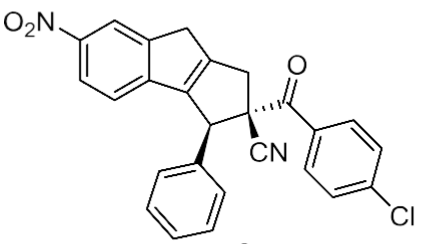

3m

^{1}H NMR (400 MHz, $CDCl_3$)

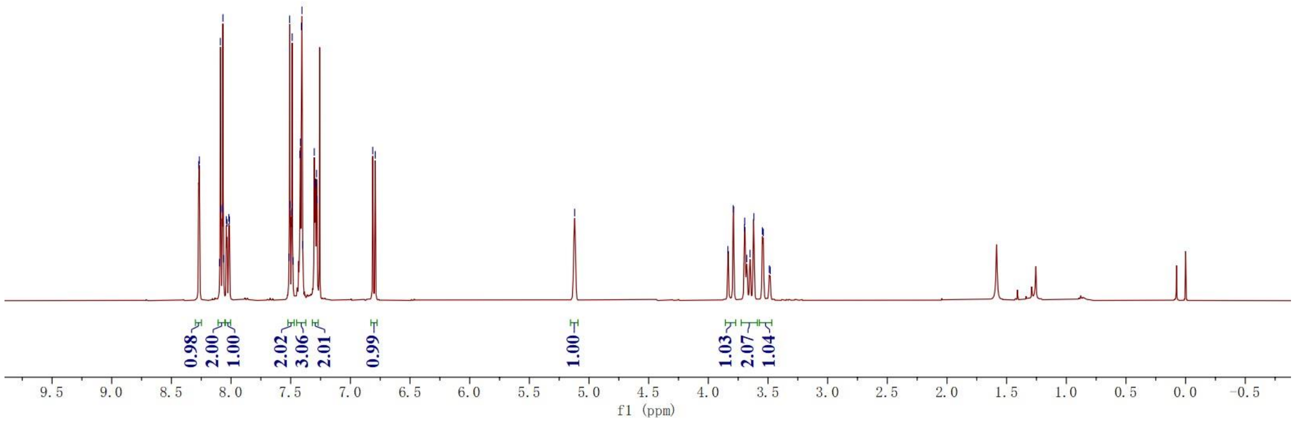

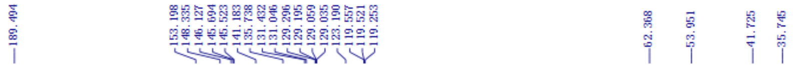

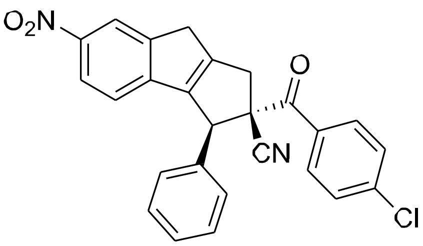

3m

^{13}C NMR (100 MHz, $CDCl_3$)

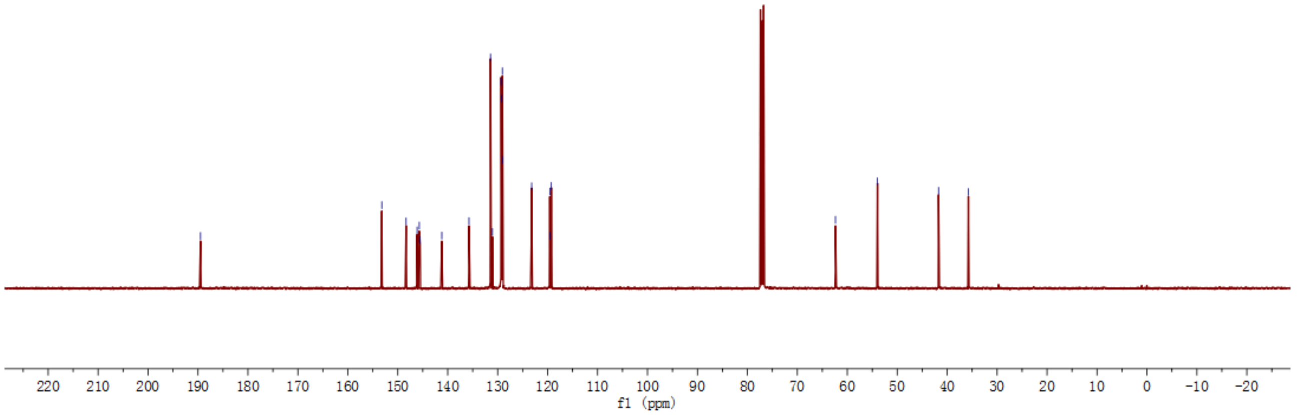

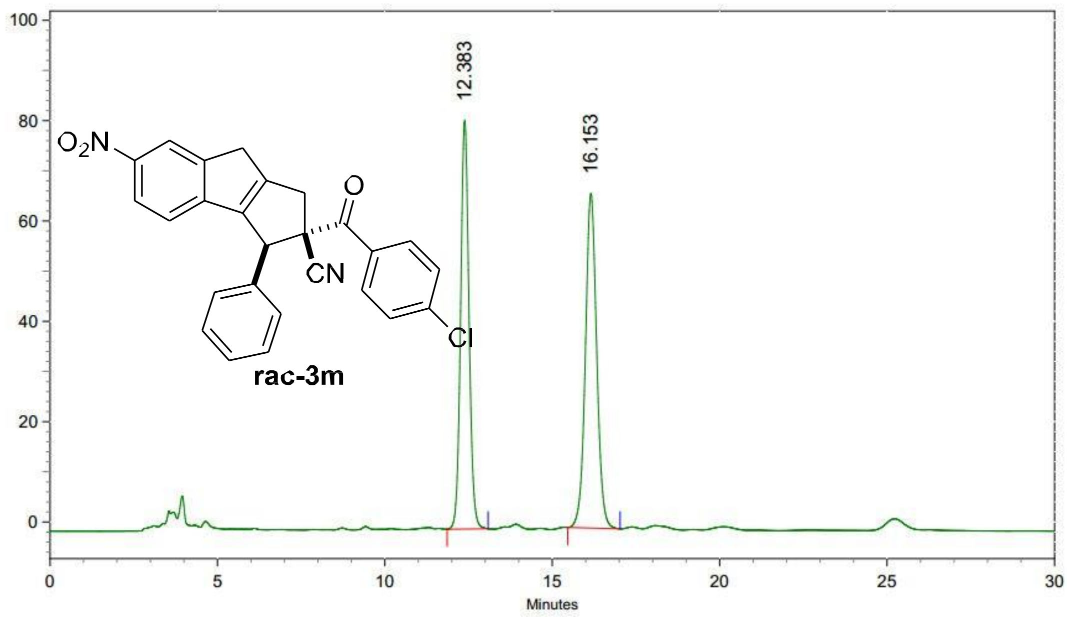

AREA PERCENT REPORT

Peak No.	Ret Time	Width	Height	Area	Area [%]
1	12.383	1.223	1366179	23725204	47.5559
2	16.153	1.567	1118453	26163836	52.4441

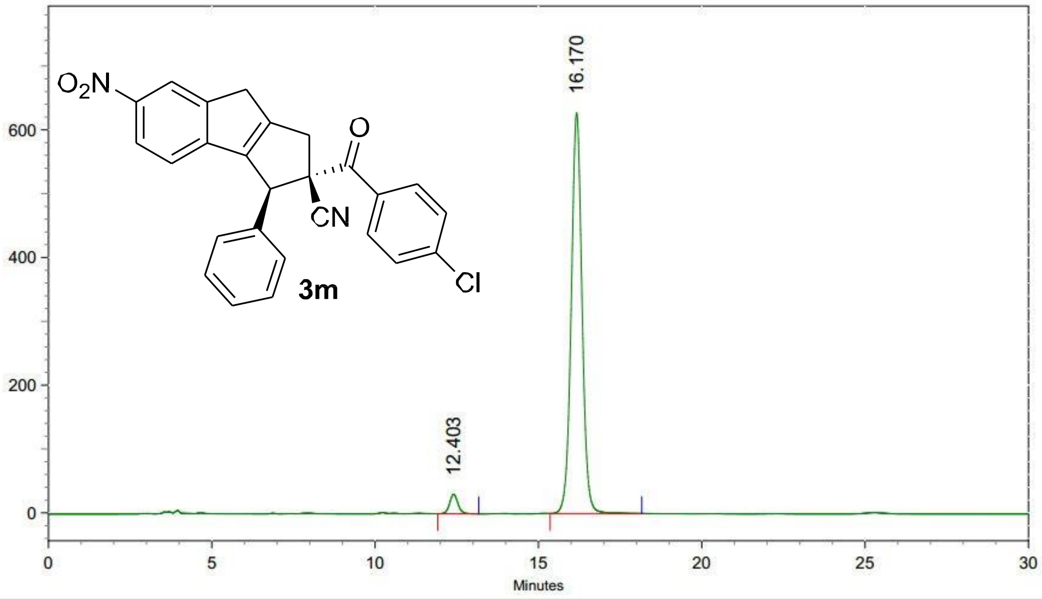

AREA PERCENT REPORT

Peak No.	Ret Time	Width	Height	Area	Area [%]
1	12.403	1.257	514824	9090498	3.6274
2	16.170	2.803	10529019	241519212	96.3727

Spectrum Plot Report

Name	CYC-20191103-6	Rack Pos.		Instrument	Instrument 1	Operator	
Inj. Vol. (ul)	10	Plate Pos.		IRM Status	Success		
Data File	CYC-20191103-6.d	Method (Acq)	TOF.m	Comment		Acq. Time (Local)	11/8/2019 10:35:35 AM (UTC+08:00)

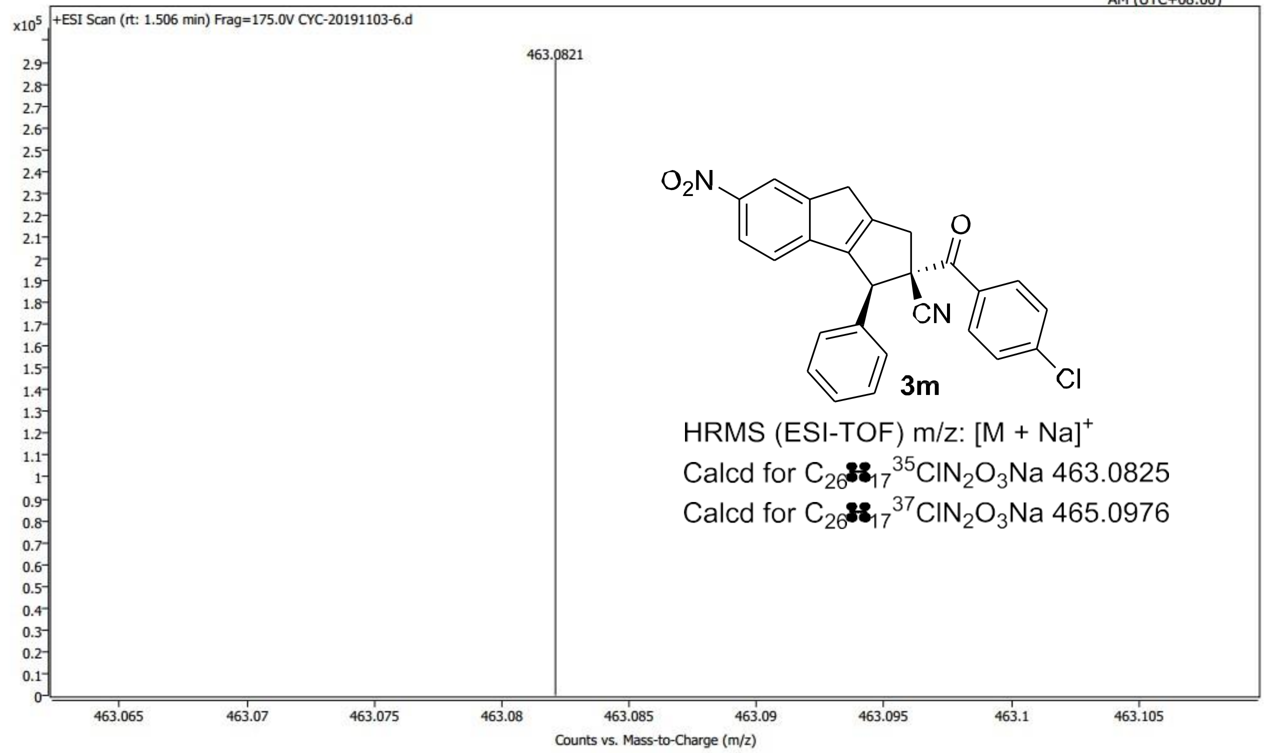

Spectrum Plot Report

Name	CYC-20191103-6	Rack Pos.		Instrument	Instrument 1	Operator	
Inj. Vol. (ul)	10	Plate Pos.		IRM Status	Success		
Data File	CYC-20191103-6.d	Method (Acq)	TOF.m	Comment		Acq. Time (Local)	11/8/2019 10:35:35 AM (UTC+08:00)

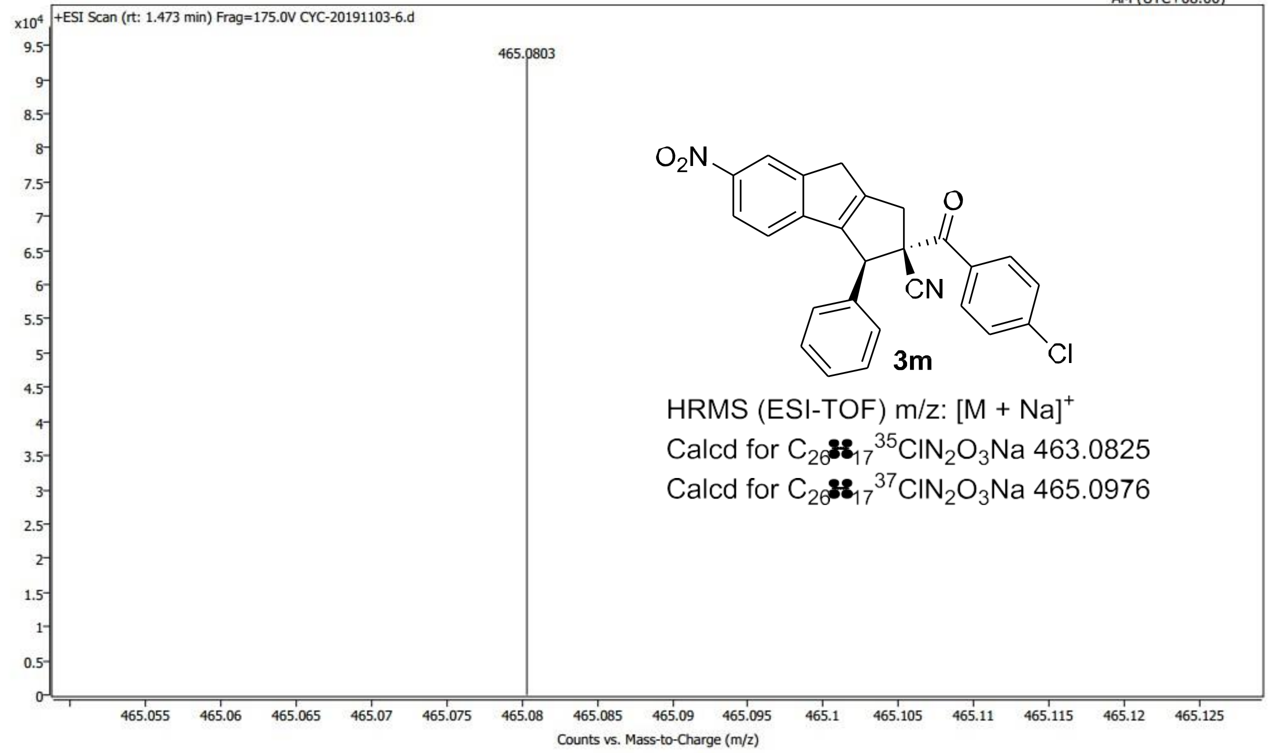

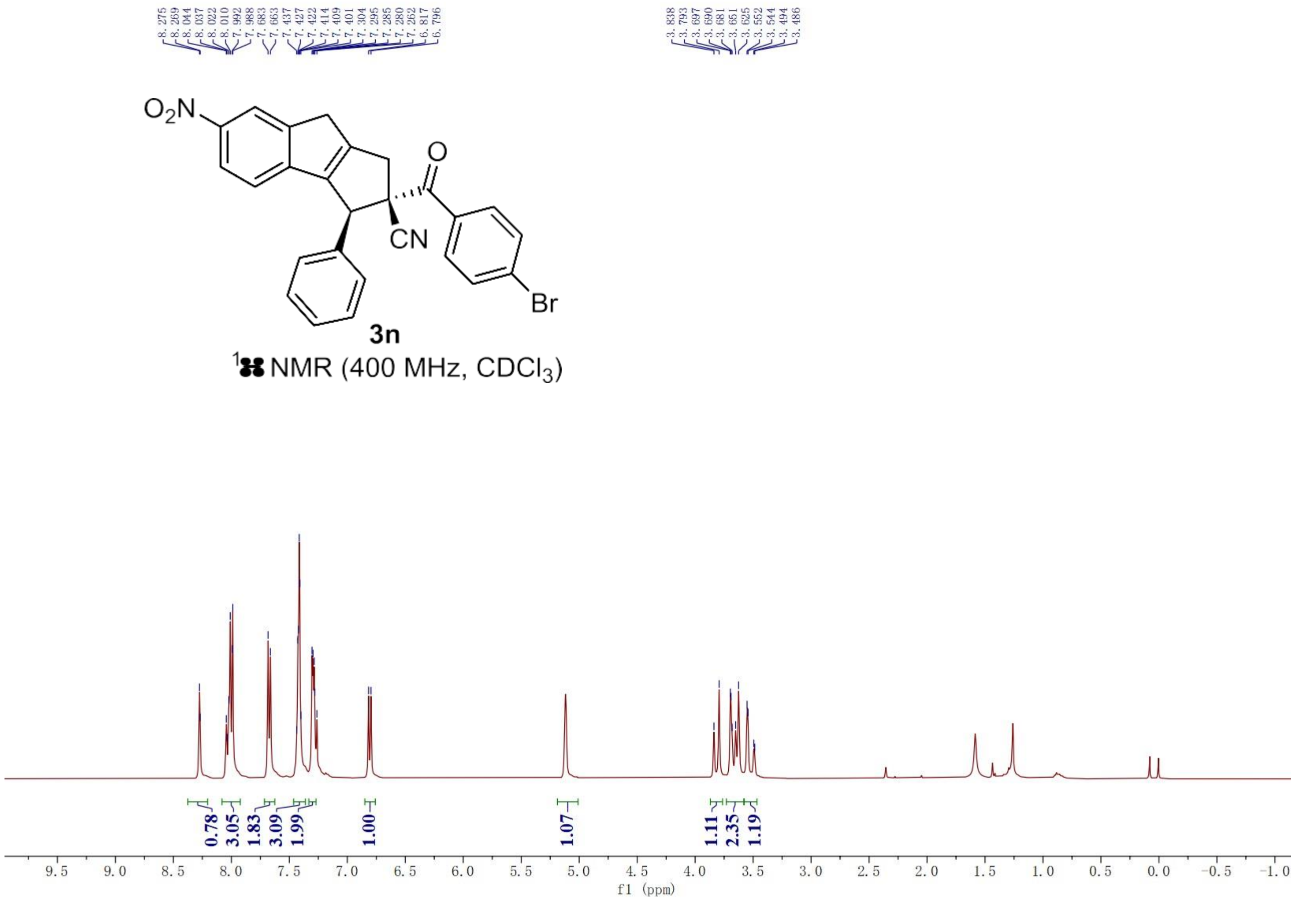

3n

^{1}H NMR (400 MHz, $CDCl_3$)

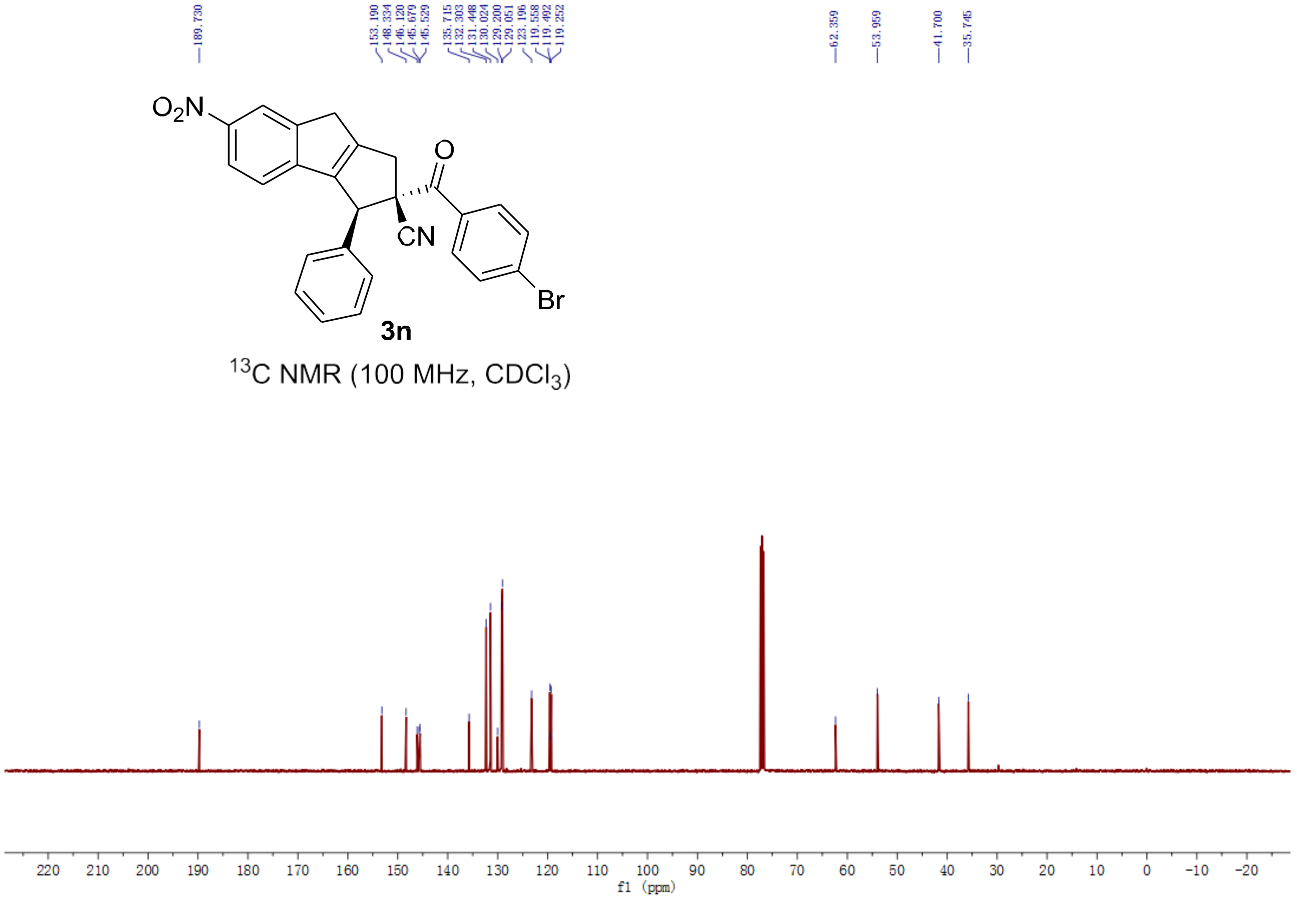

3n

^{13}C NMR (100 MHz, $CDCl_3$)

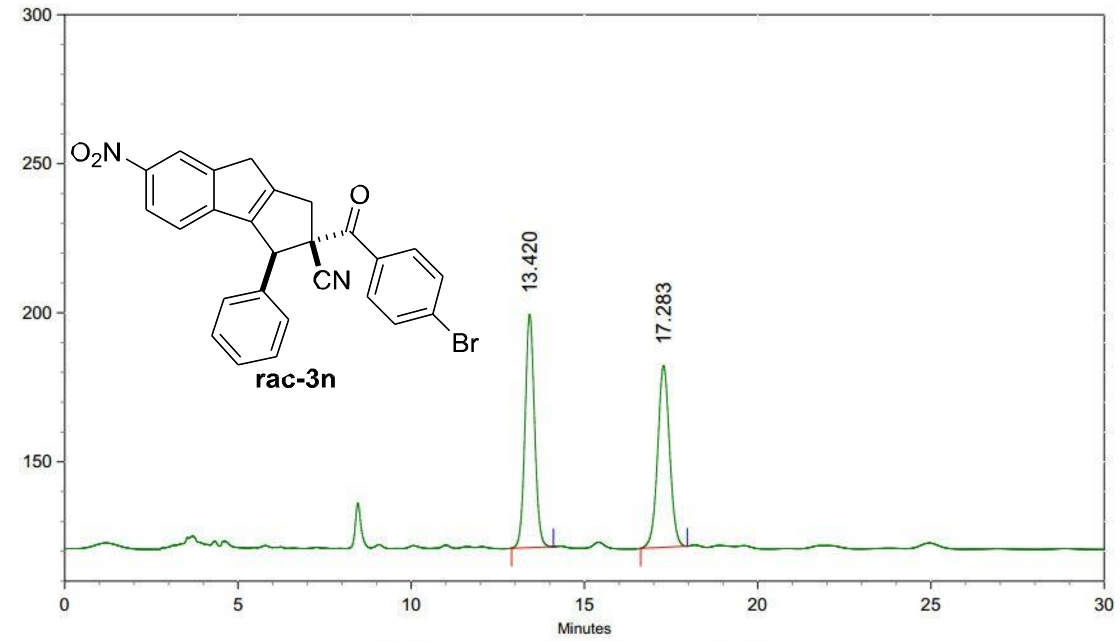

AREA PERCENT REPORT

Peak No.	Ret Time	Width	Height	Area	Area [%]
1	13.420	1.210	1313502	25019512	50.4931
2	17.283	1.343	1022344	24530872	49.5069

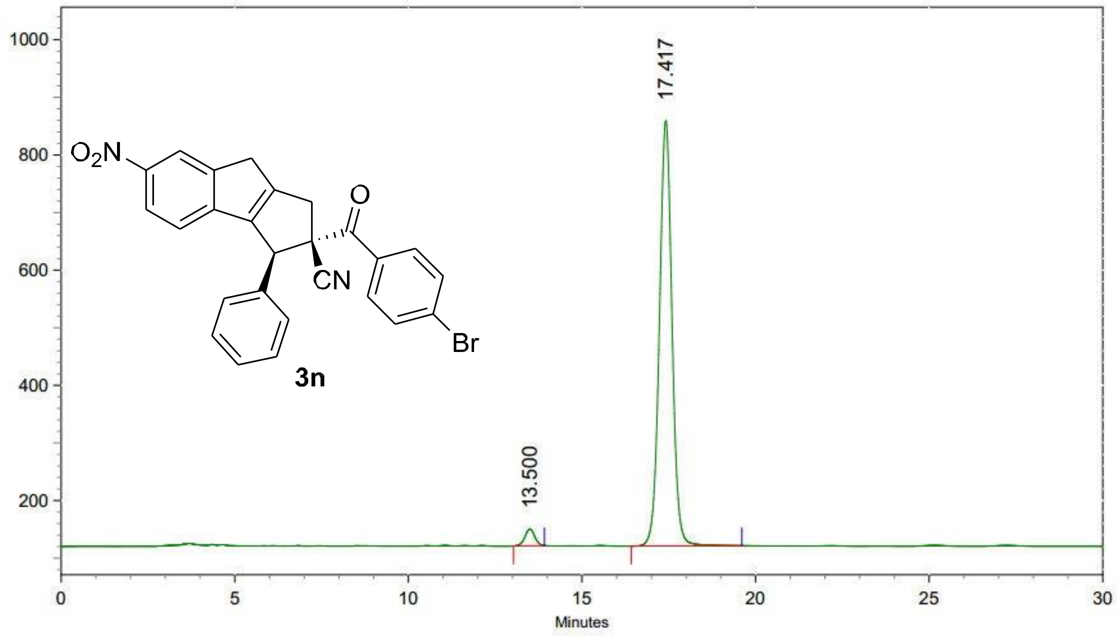

AREA PERCENT REPORT

Peak No.	Ret Time	Width	Height	Area	Area [%]
1	13.500	0.887	490356	9592131	2.9857
2	17.417	3.183	12390634	311678228	97.0143

Spectrum Plot Report

Agilent

Name	CYC-20191103-10	Rack Pos.		Instrument	Instrument 1	Operator	
Inj. Vol. (ul)	10	Plate Pos.		IRM Status	Success		
Data File	CYC-20191103-10.d	Method (Acq)	TOF.m	Comment		Acq. Time (Local)	11/8/2019 10:47:34 AM (UTC+08:00)

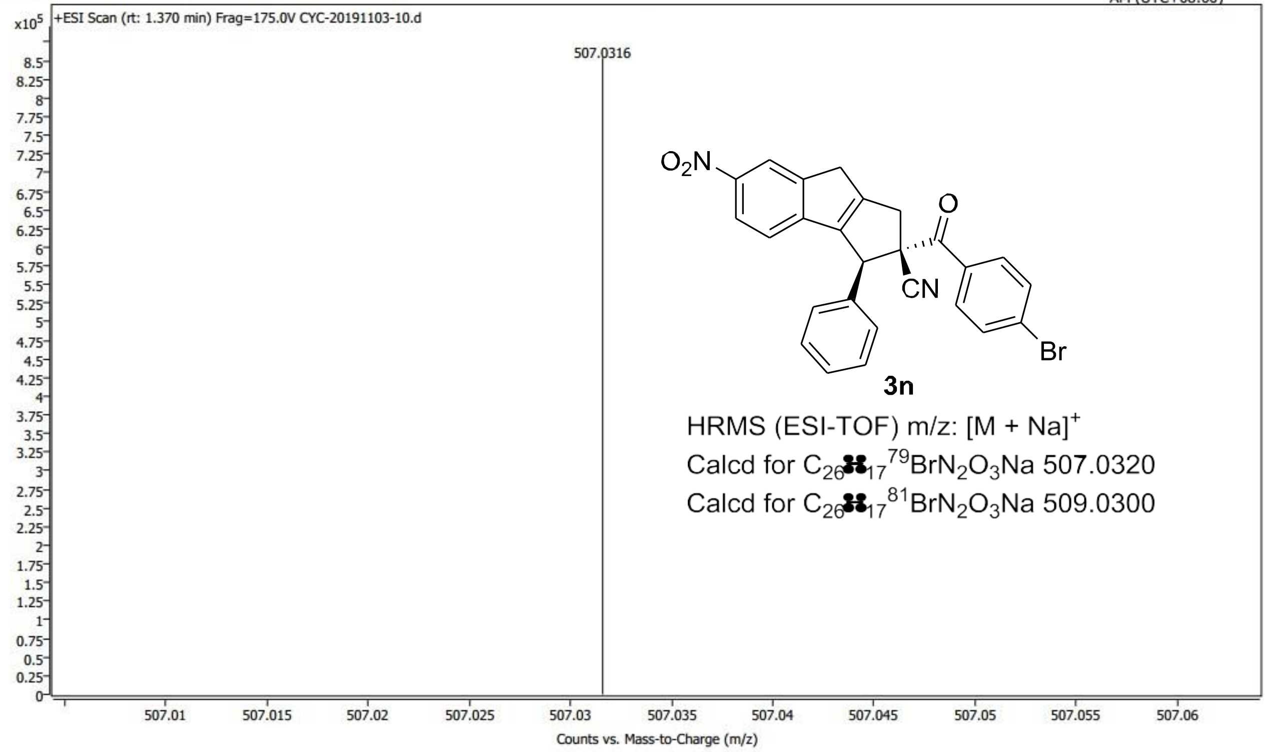

Spectrum Plot Report

Agilent

Name	CYC-20191103-10	Rack Pos.		Instrument	Instrument 1	Operator	
Inj. Vol. (ul)	10	Plate Pos.		IRM Status	Success		
Data File	CYC-20191103-10.d	Method (Acq)	TOF.m	Comment		Acq. Time (Local)	11/8/2019 10:47:34 AM (UTC+08:00)

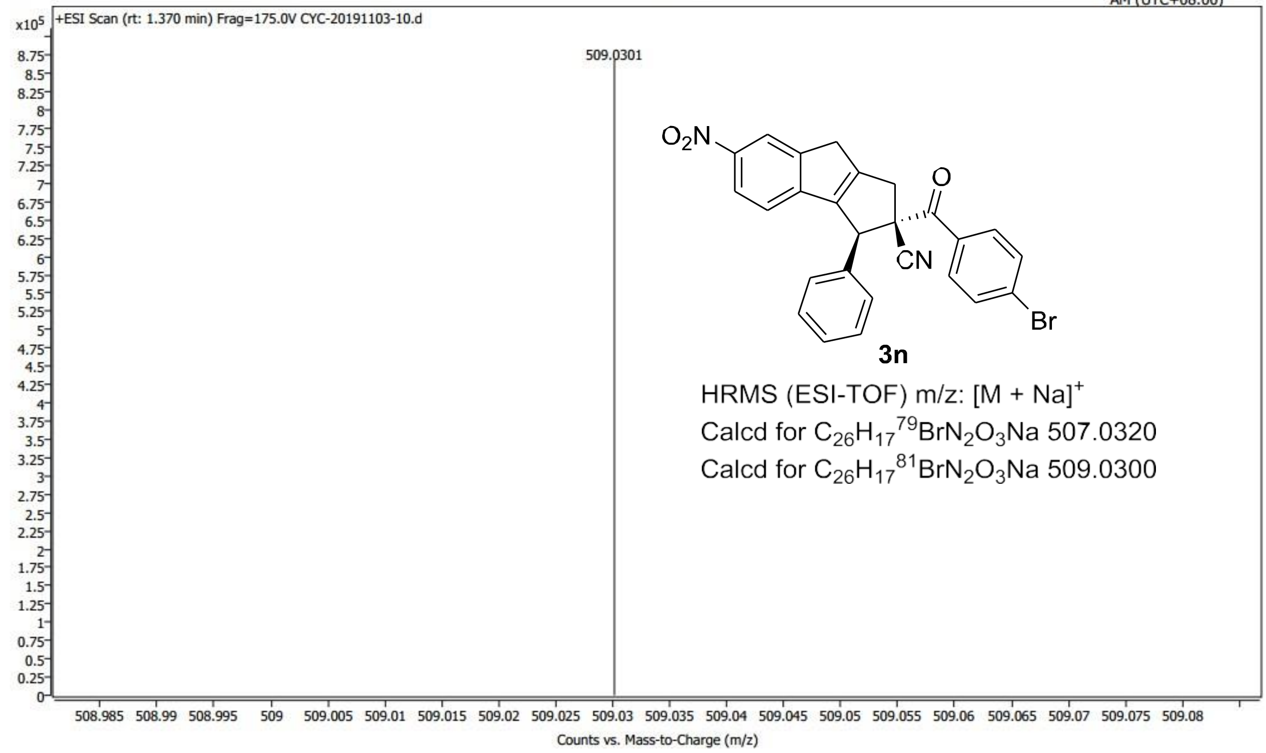

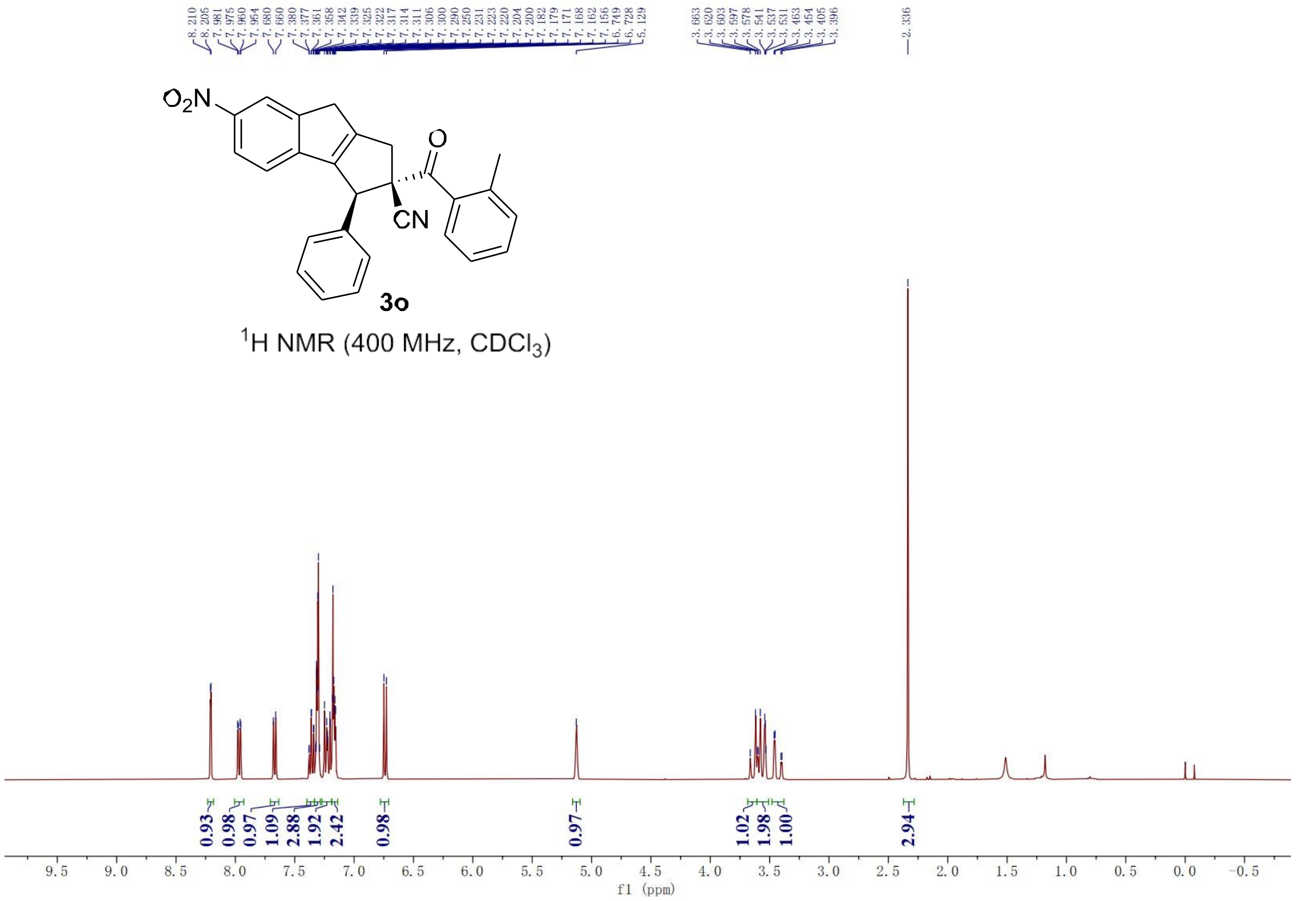
3o
^{1}H NMR (400 MHz, CDCl$_3$)
f1 (ppm)

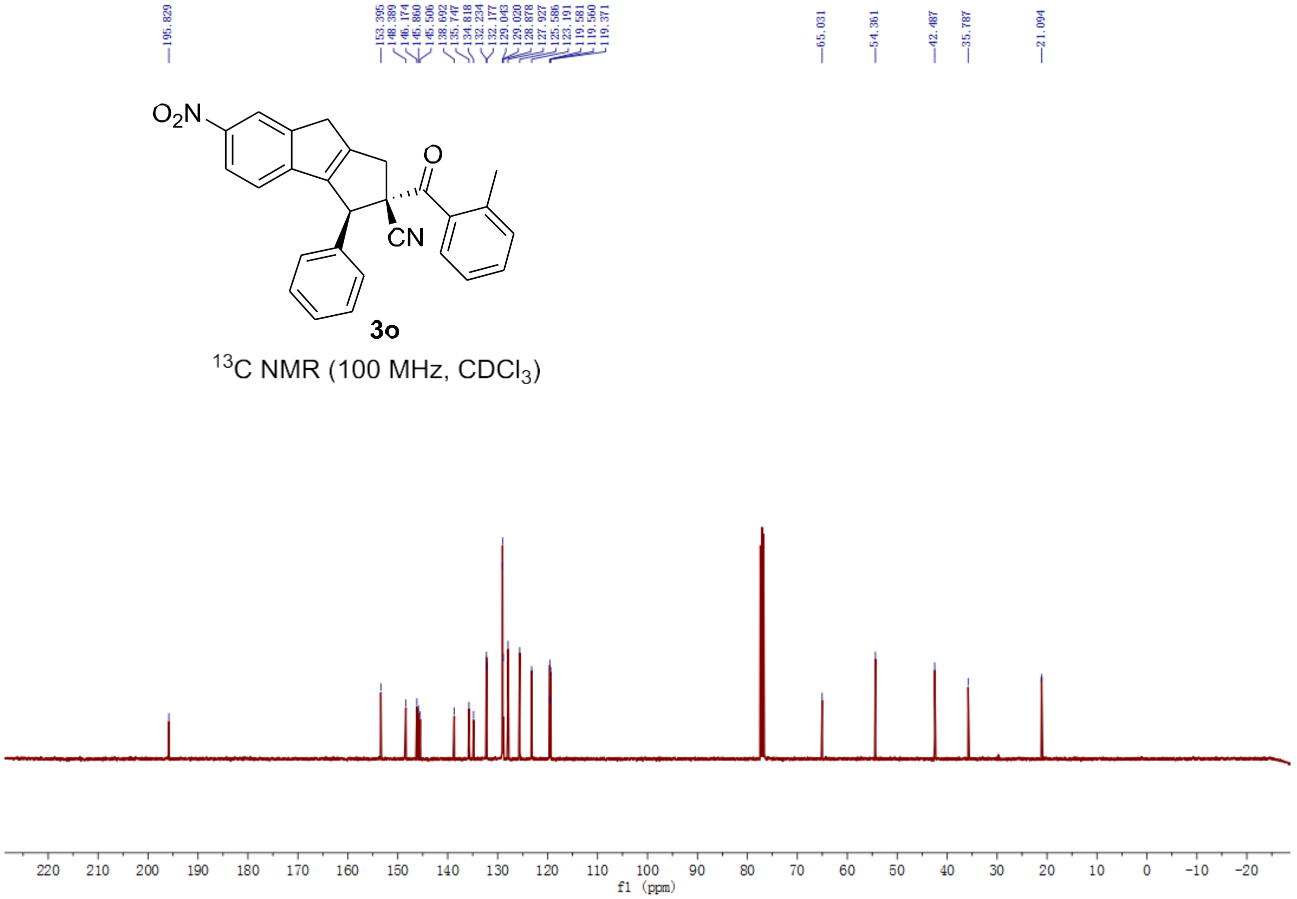
3o
^{13}C NMR (100 MHz, CDCl$_3$)
f1 (ppm)

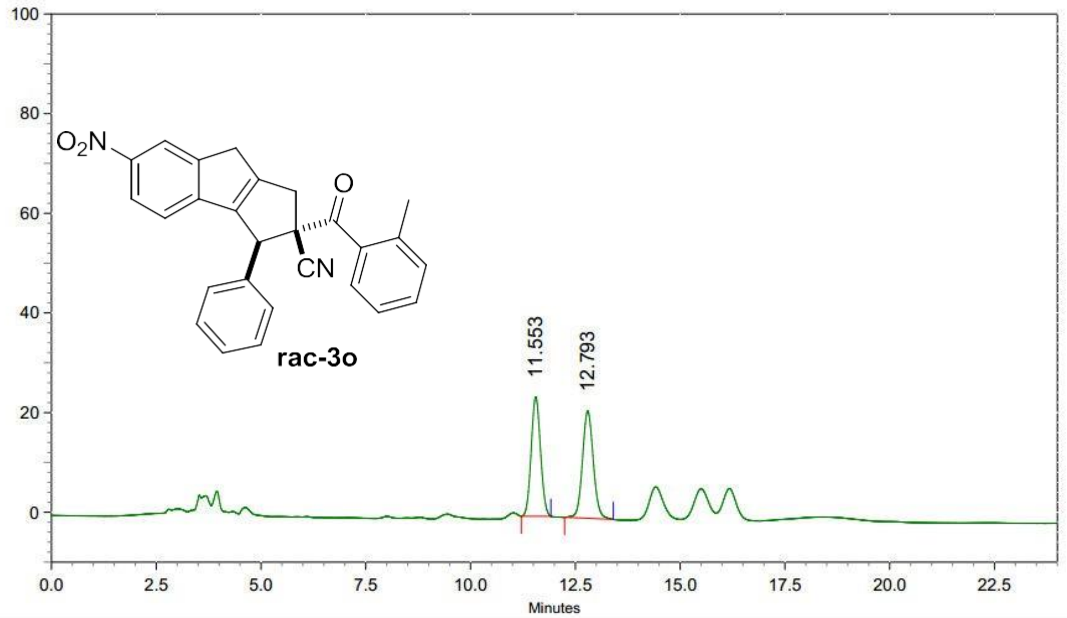

AREA PERCENT REPORT

Peak No.	Ret Time	Width	Height	Area	Area [%]
1	11.553	0.707	402004	6228146	48.9014
2	12.793	1.163	361638	6507974	51.0986

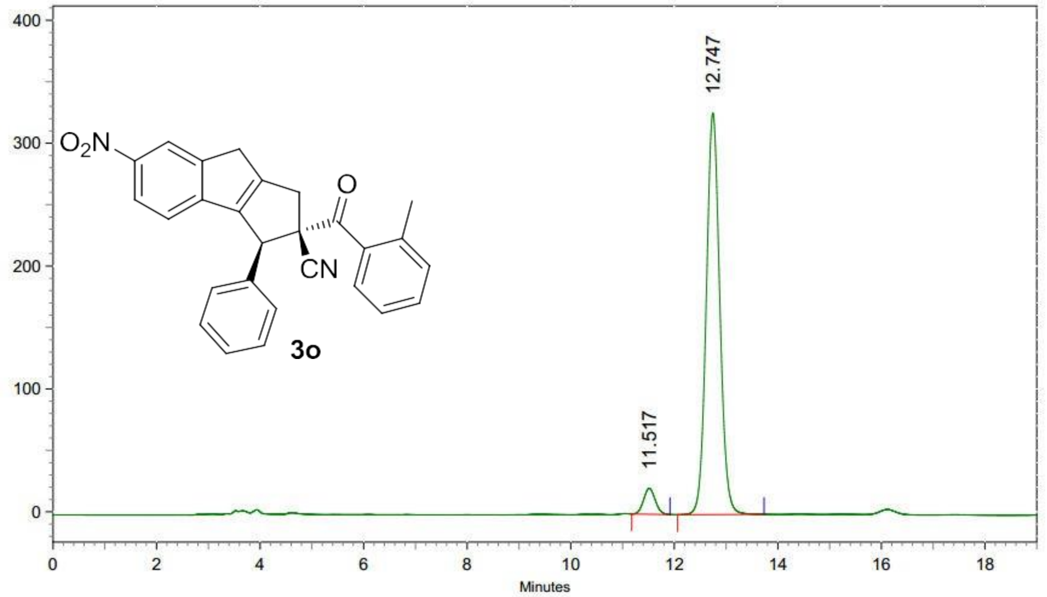

AREA PERCENT REPORT

Peak No.	Ret Time	Width	Height	Area	Area [%]
1	11.517	0.740	354759	5524992	5.2765
2	12.747	1.670	5480554	99183875	94.7235

Spectrum Plot Report

Agilent

Name	CYC-20191018-63	Rack Pos.		Instrument	Instrument 1	Operator	
Inj. Vol. (ul)	10	Plate Pos.		IRM Status	Success		
Data File	CYC-20191018-63.d	Method (Acq)	TOF.m	Comment		Acq. Time (Local)	10/29/2019 4:04:25 PM (UTC+08:00)

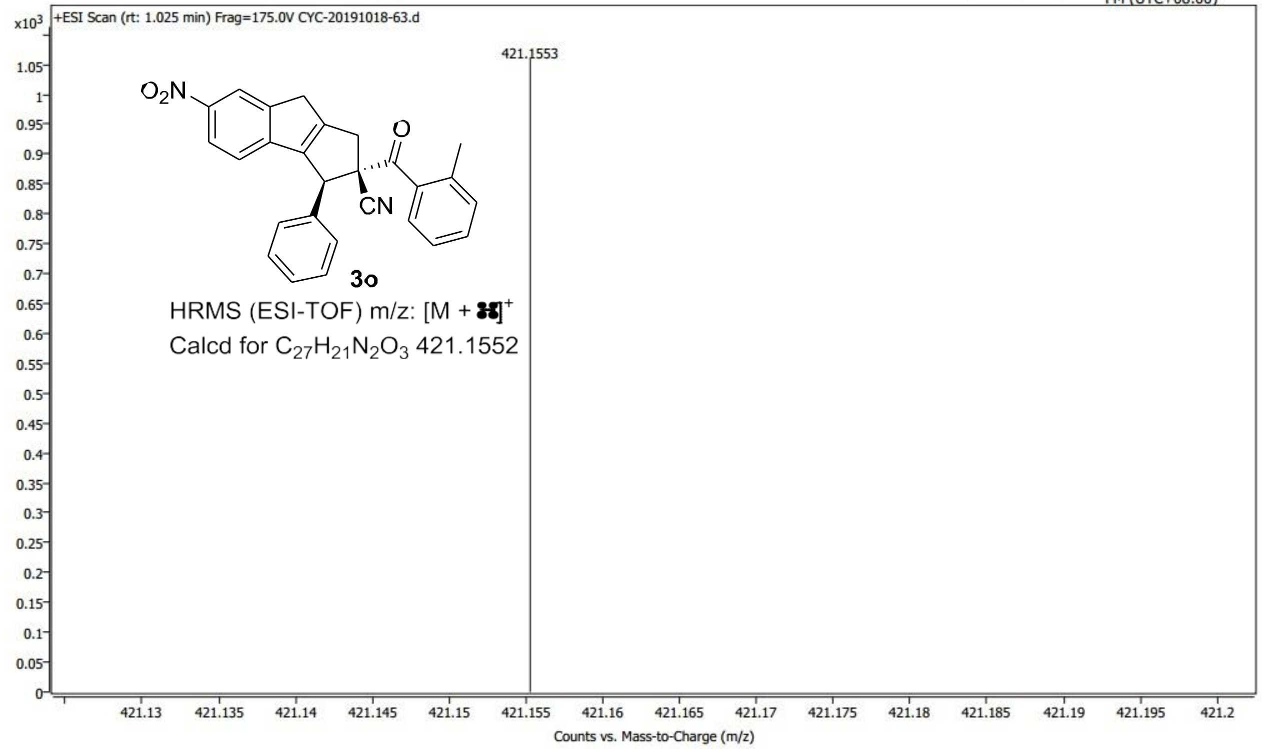

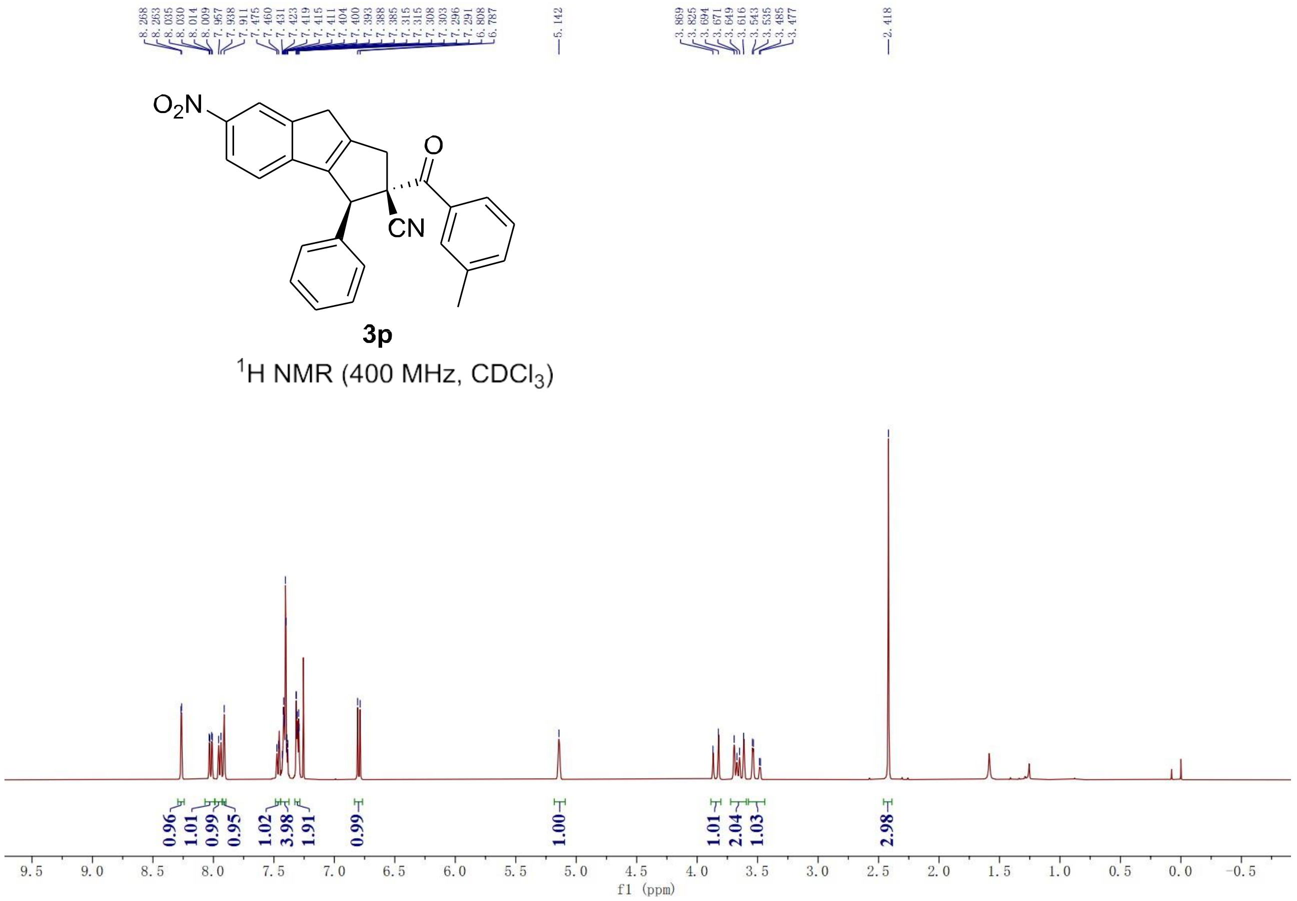

3p

^{1}H NMR (400 MHz, $CDCl_3$)

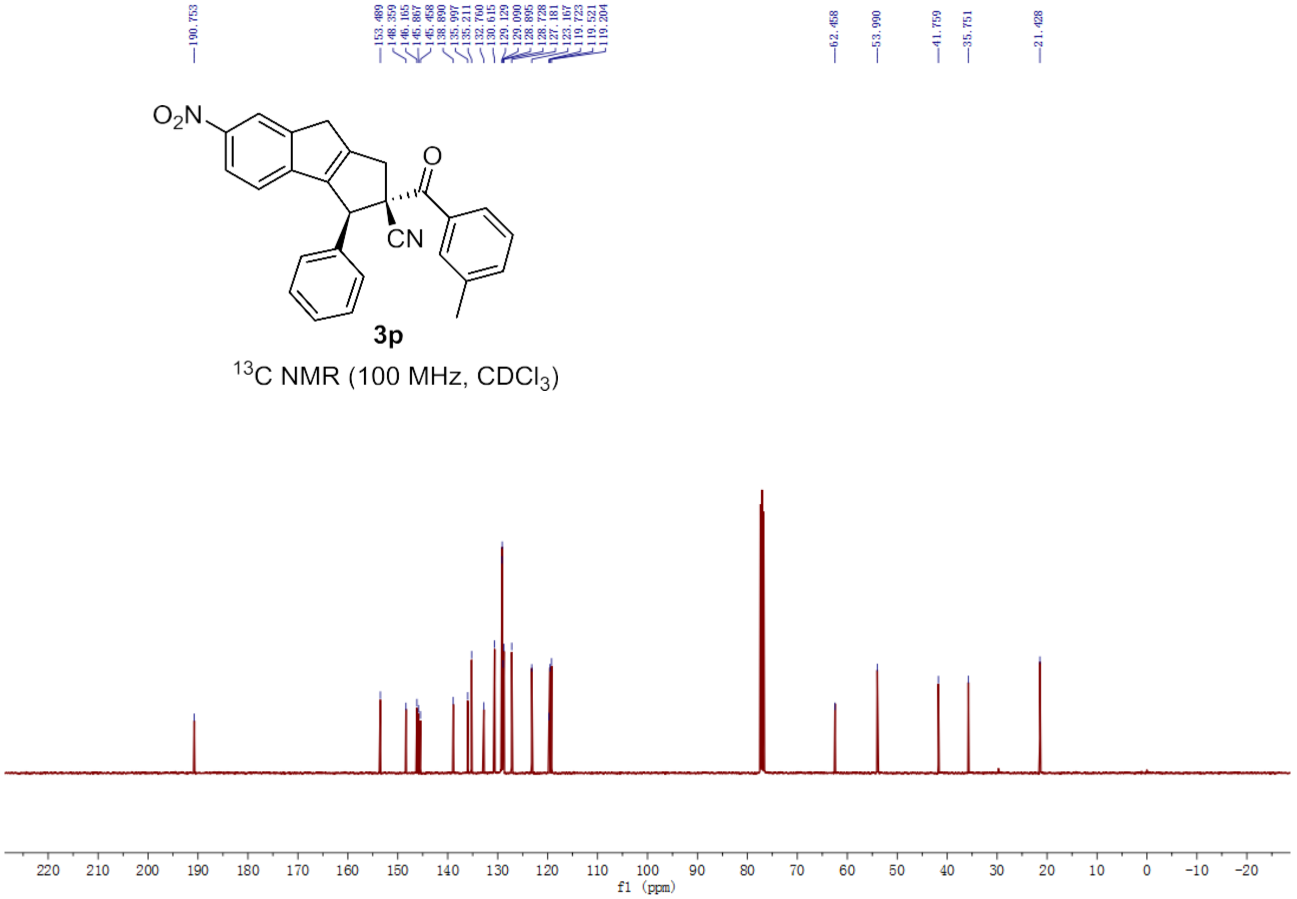

3p

^{13}C NMR (100 MHz, $CDCl_3$)

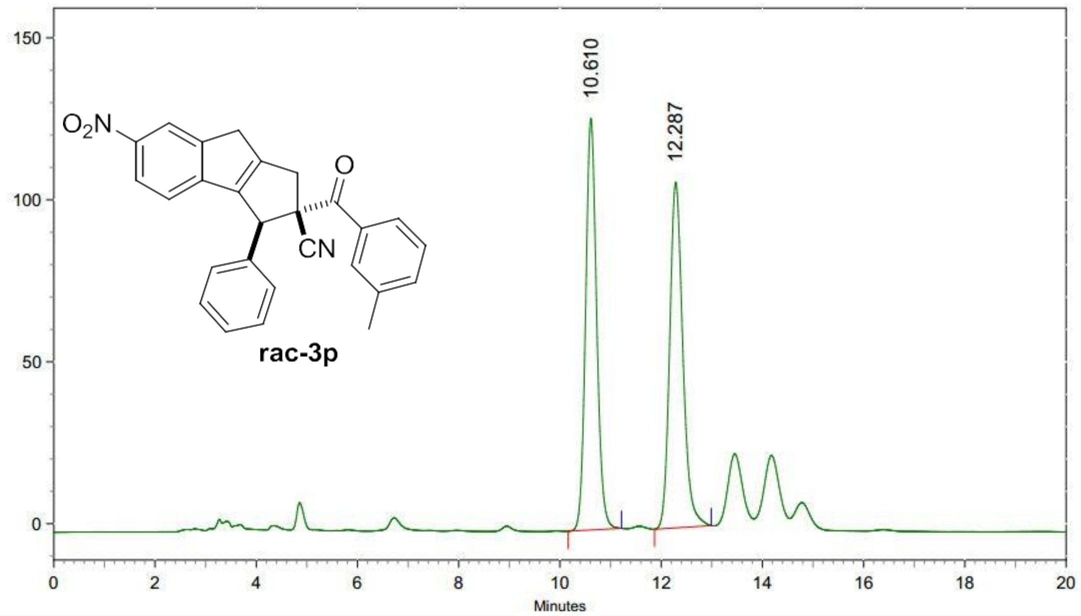

AREA PERCENT REPORT

Peak No.	Ret Time	Width	Height	Area	Area [%]
1	10.610	1.053	2131351	32150434	49.8933
2	12.287	1.120	1789656	32287971	50.1067

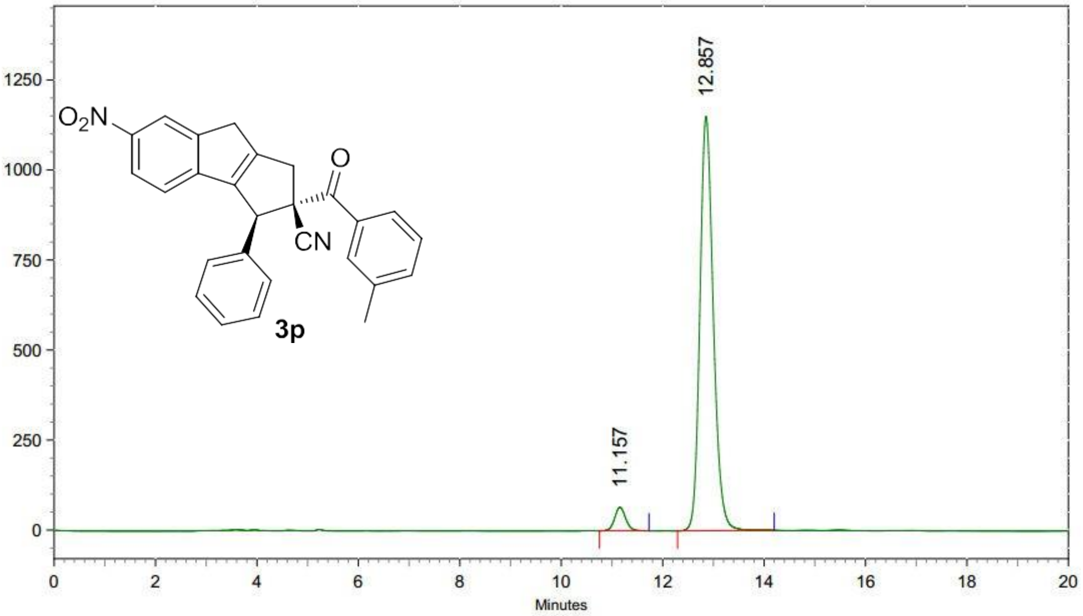

AREA PERCENT REPORT

Peak No.	Ret Time	Width	Height	Area	Area [%]
1	11.157	0.977	1099093	16538190	4.4579
2	12.857	1.907	19301059	354450217	95.5421

Spectrum Plot Report

Agilent

Name	CYC-20191018-59	Rack Pos.		Instrument	Instrument 1	Operator	
Inj. Vol. (ul)	10	Plate Pos.		IRM Status	Success		
Data File	CYC-20191018-59.d	Method (Acq)	TOF.m	Comment		Acq. Time (Local)	10/29/2019 3:52:21 PM (UTC+08:00)

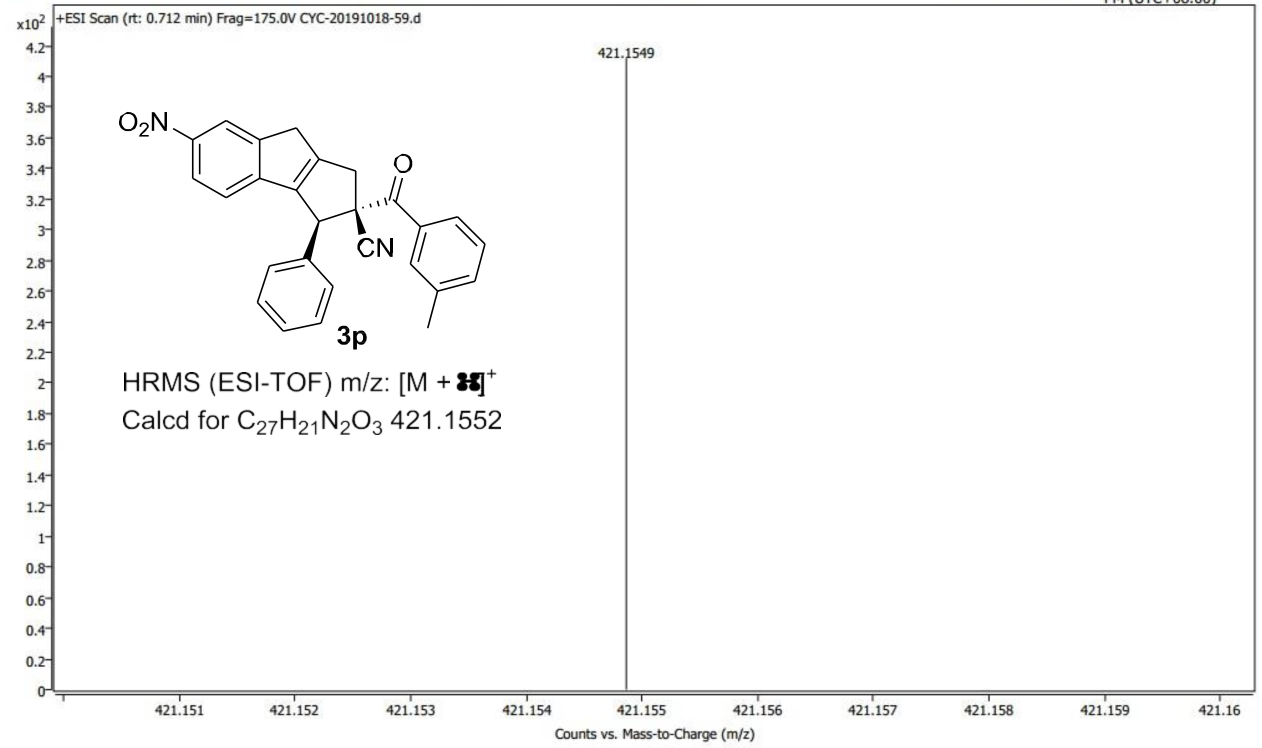

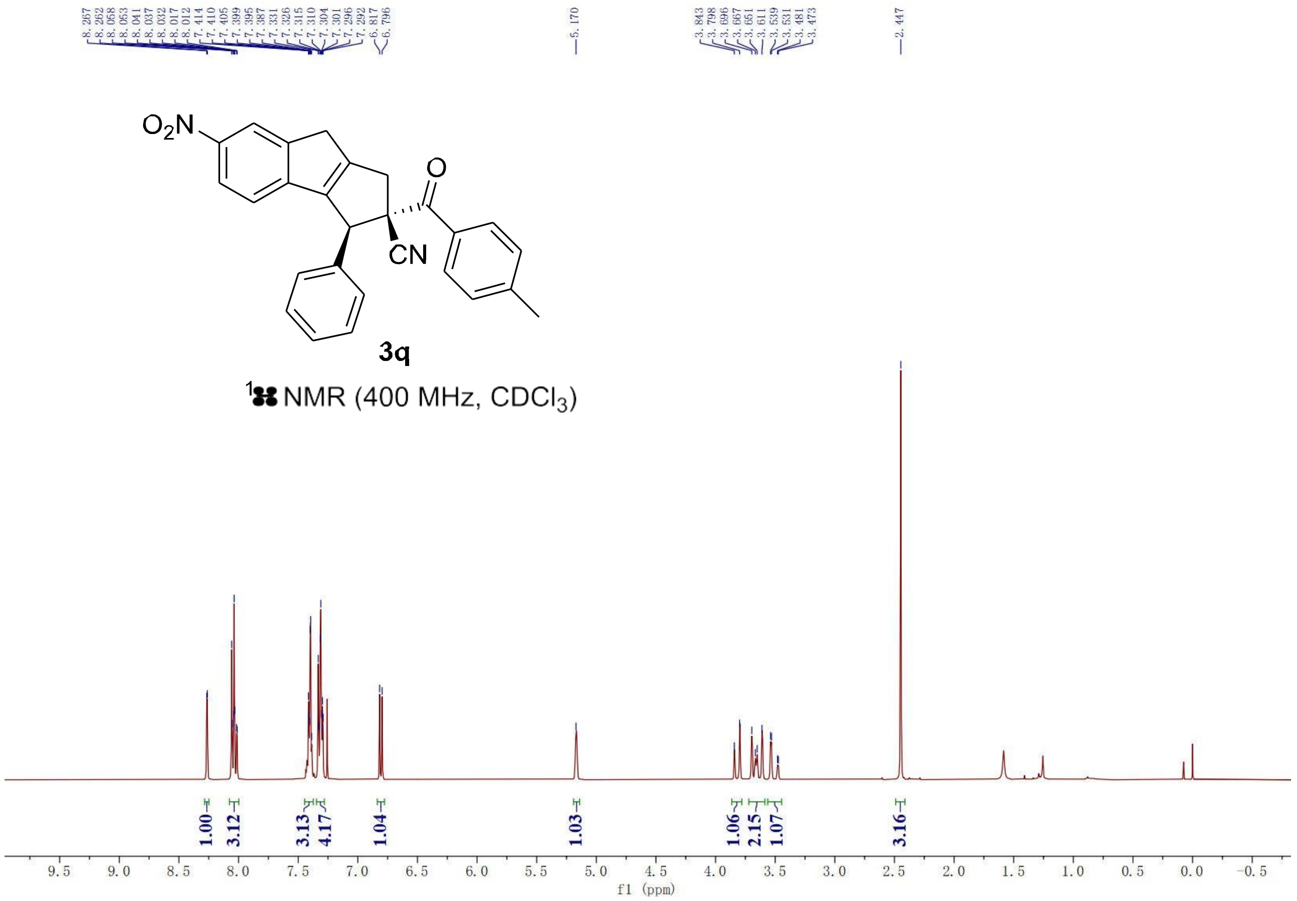
3q
1H NMR (400 MHz, CDCl3)
f1 (ppm)

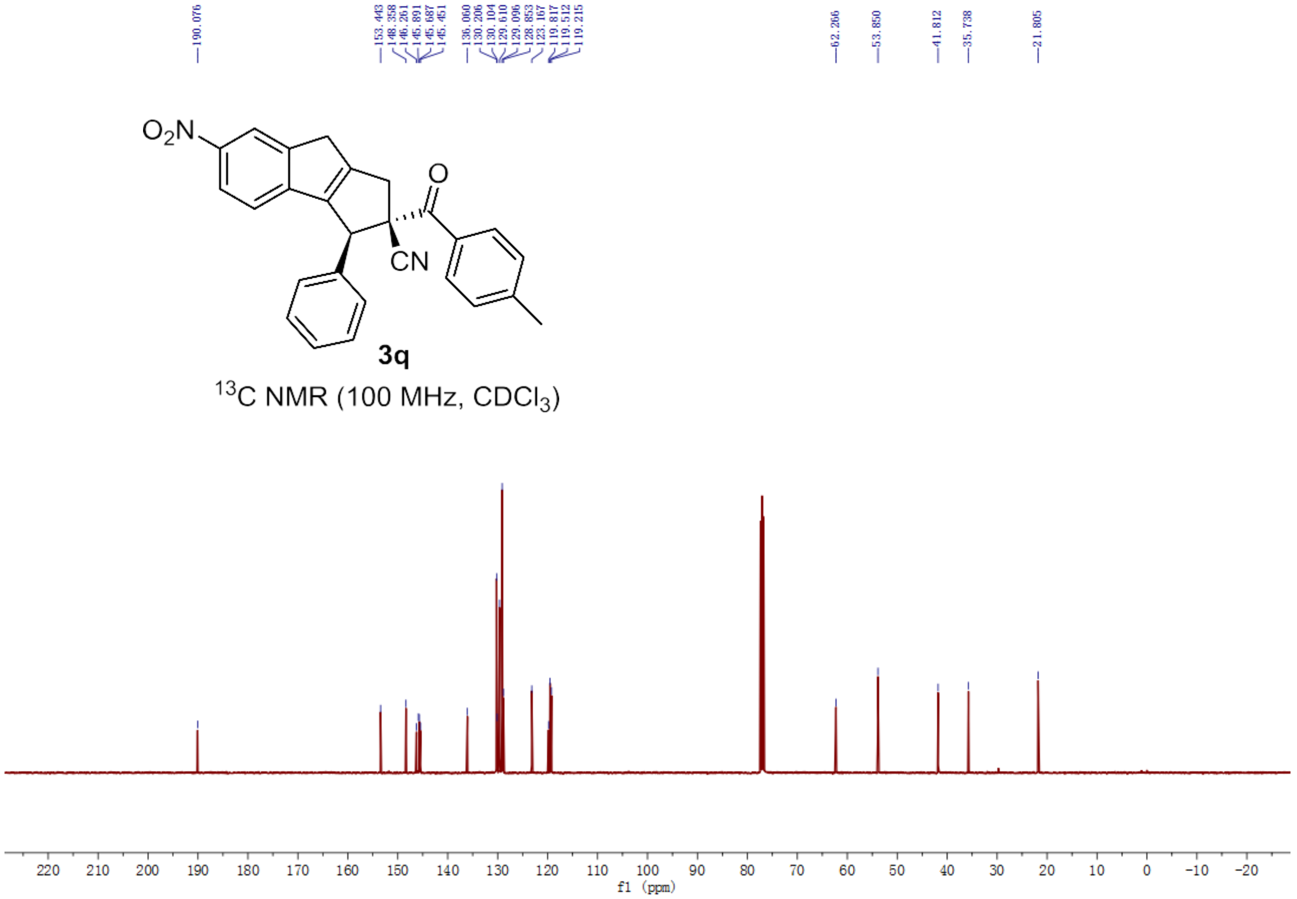
3q
13C NMR (100 MHz, CDCl3)
f1 (ppm)

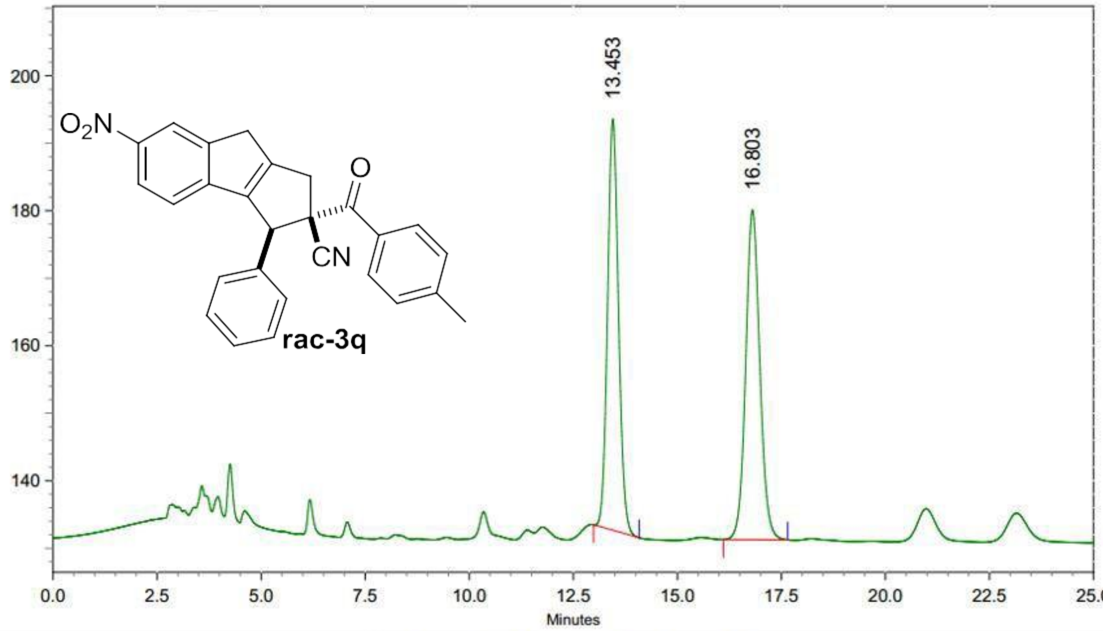

AREA PERCENT REPORT

Peak No.	Ret Time	Width	Height	Area	Area [%]
1	13.453	1.090	1021182	18628741	48.9803
2	16.803	1.540	819142	19404378	51.0197

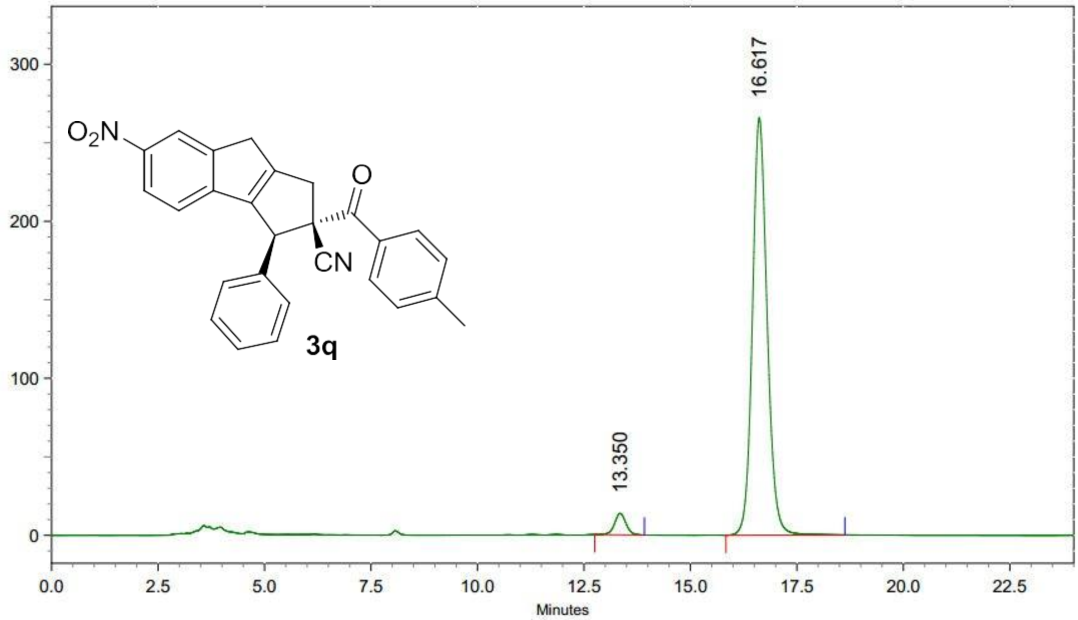

AREA PERCENT REPORT

Peak No.	Ret Time	Width	Height	Area	Area [%]
1	13.350	1.167	229248	4293777	3.8584
2	16.617	2.793	4460575	106989874	96.1416

Spectrum Plot Report

Agilent

Name	CYC-20191103-12	Rack Pos.		Instrument	Instrument 1	Operator	
Inj. Vol. (ul)	10	Plate Pos.		IRM Status	Success		
Data File	CYC-20191103-12.d	Method (Acq)	TOF.m	Comment		Acq. Time (Local)	11/8/2019 10:53:36 AM (UTC+08:00)

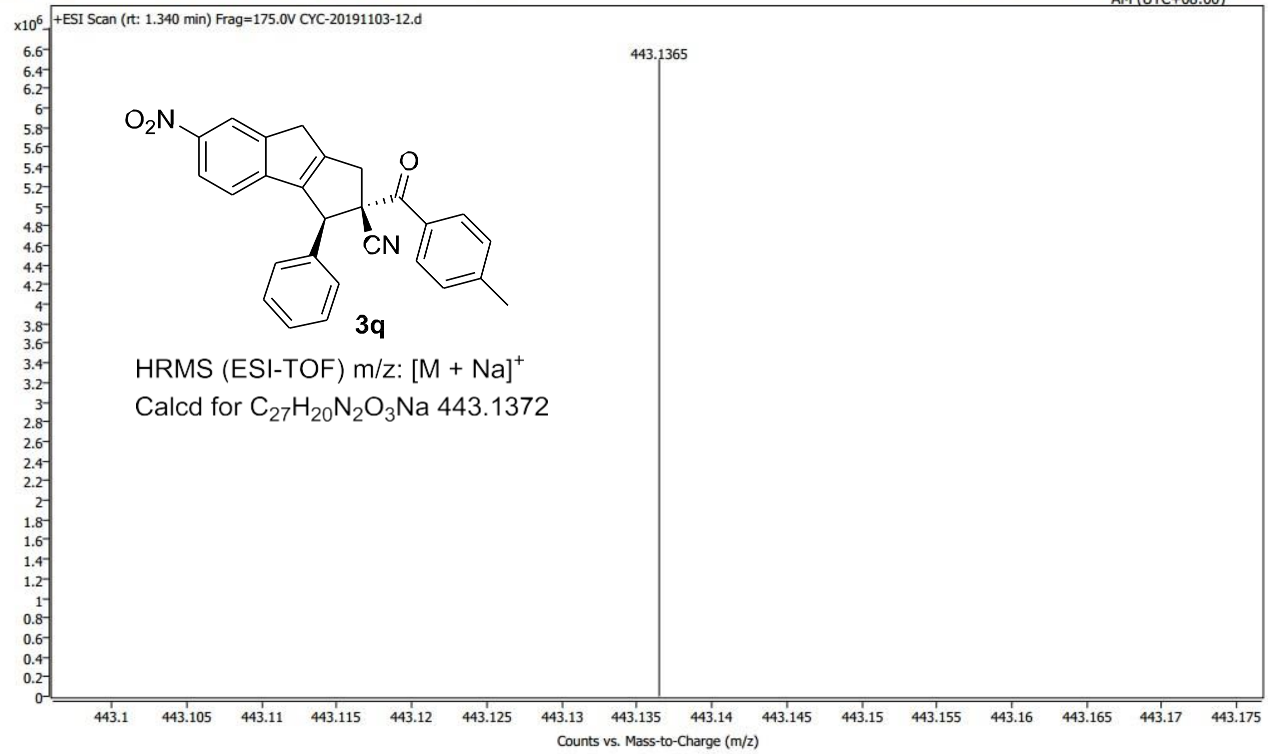

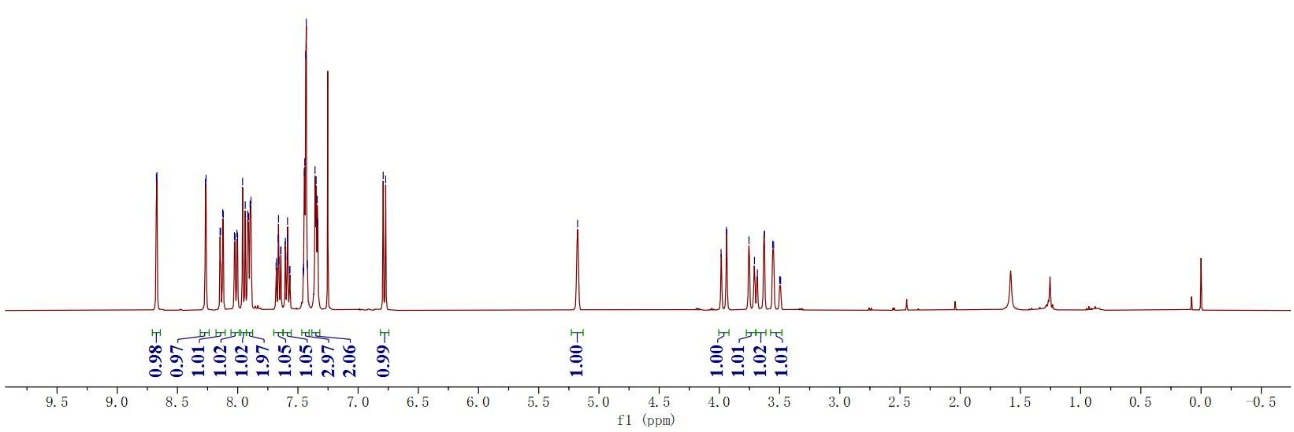

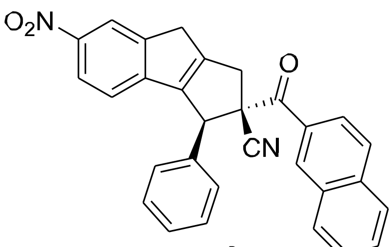

3r

^{1}H NMR (400 MHz, $CDCl_3$)

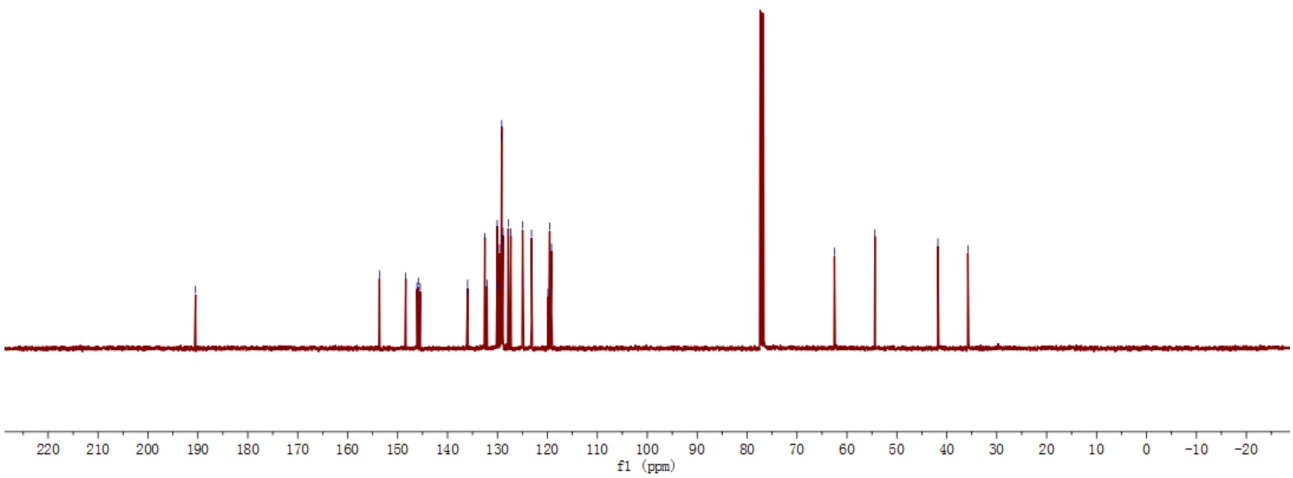

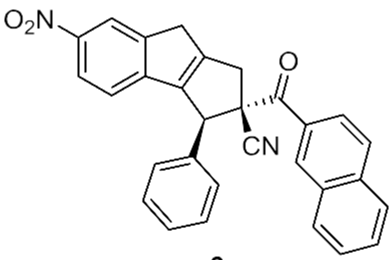

3r

^{13}C NMR (100 MHz, $CDCl_3$)

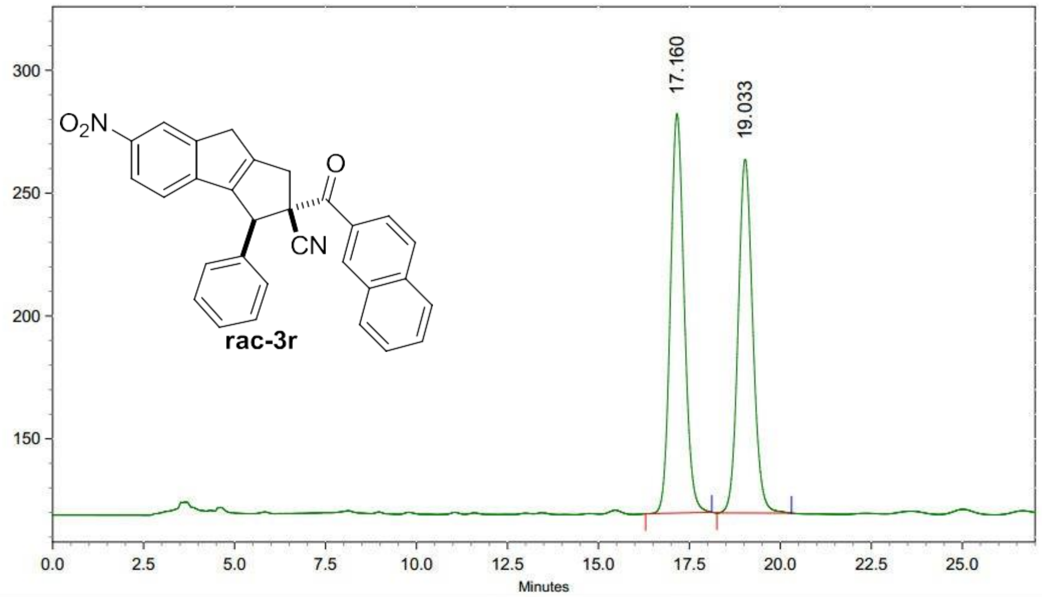

AREA PERCENT REPORT

Peak No.	Ret Time	Width	Height	Area	Area [%]
1	17.160	1.820	2727111	72005261	51.5611
2	19.033	2.050	2415633	67645165	48.4389

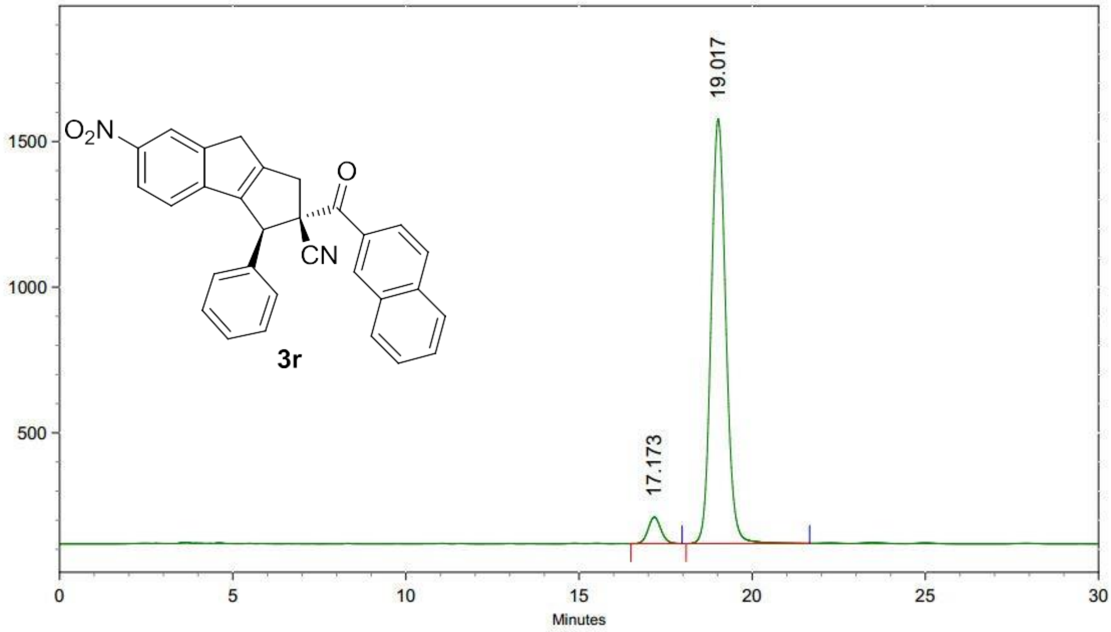

AREA PERCENT REPORT

Peak No.	Ret Time	Width	Height	Area	Area [%]
1	17.173	1.480	1527549	39200306	5.1541
2	19.017	3.563	24445602	721371191	94.8459

Spectrum Plot Report

Name	CYC-20191018-58	Rack Pos.		Instrument	Instrument 1	Operator	
Inj. Vol. (ul)	10	Plate Pos.		IRM Status	Success		
Data File	CYC-20191018-58.d	Method (Acq)	TOF.m	Comment		Acq. Time (Local)	10/29/2019 3:49:20 PM (UTC+08:00)

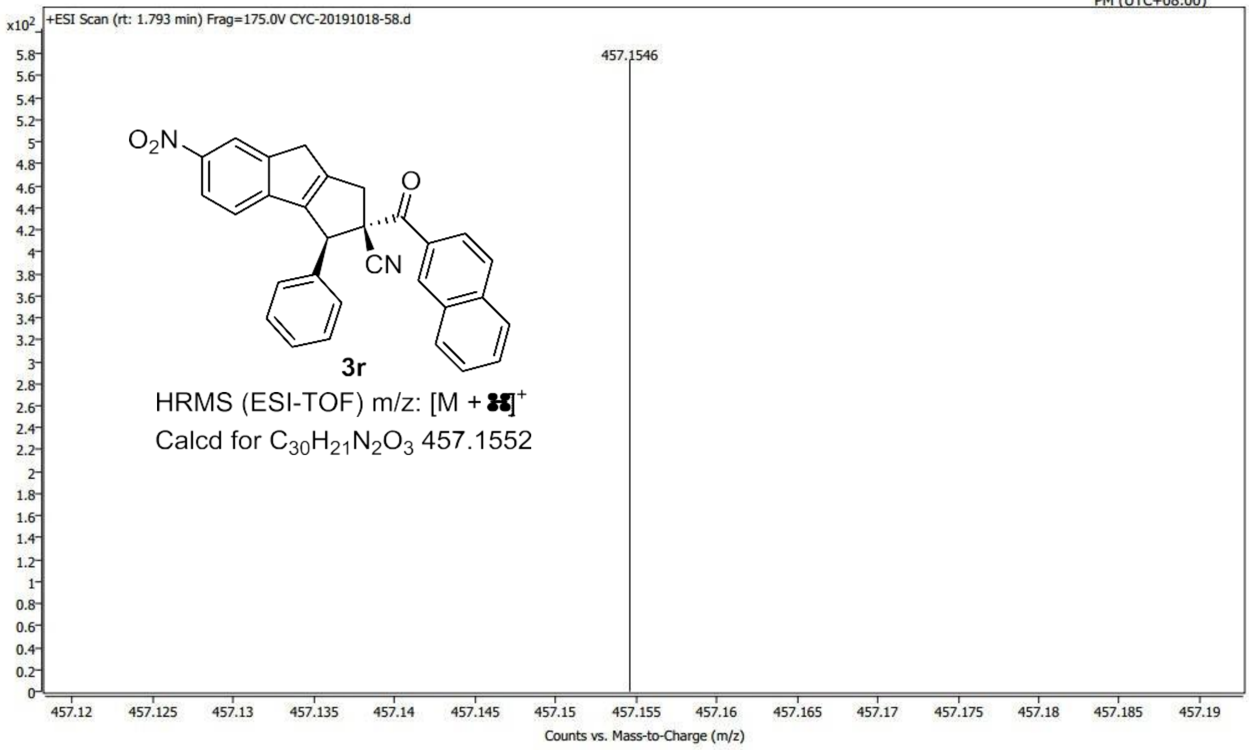

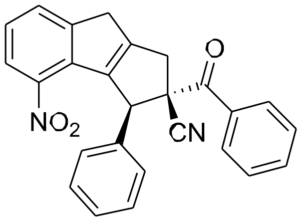

3s

^1H NMR (400 MHz, $CDCl_3$)

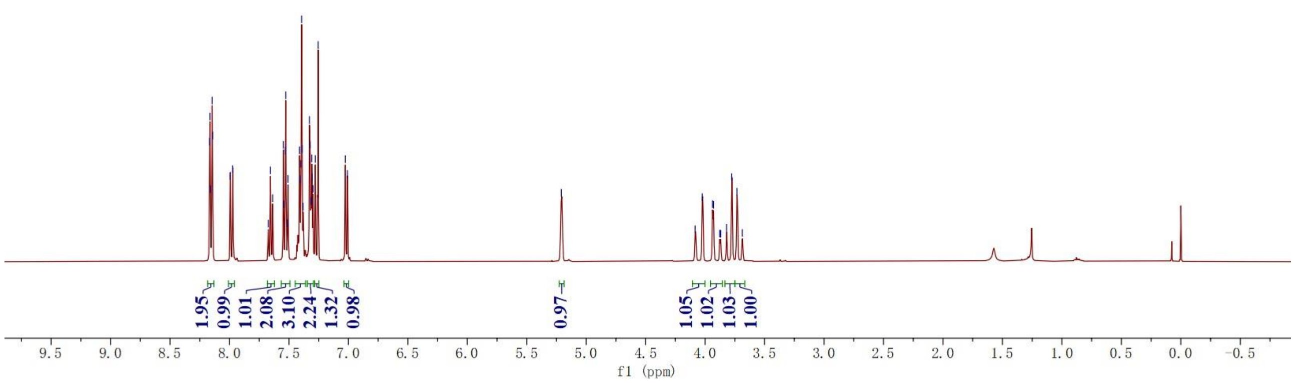

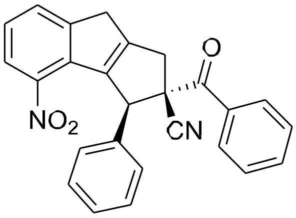

3s

^{13}C NMR (100 MHz, $CDCl_3$)

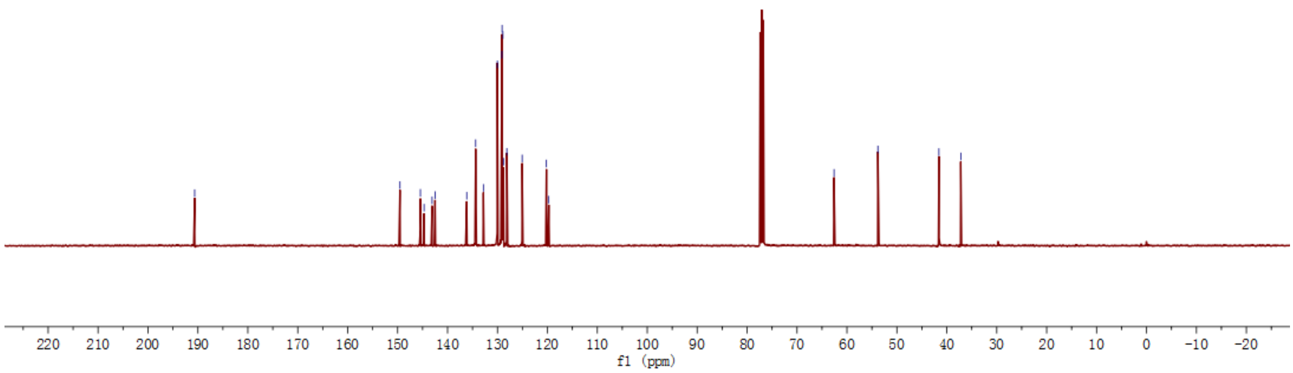

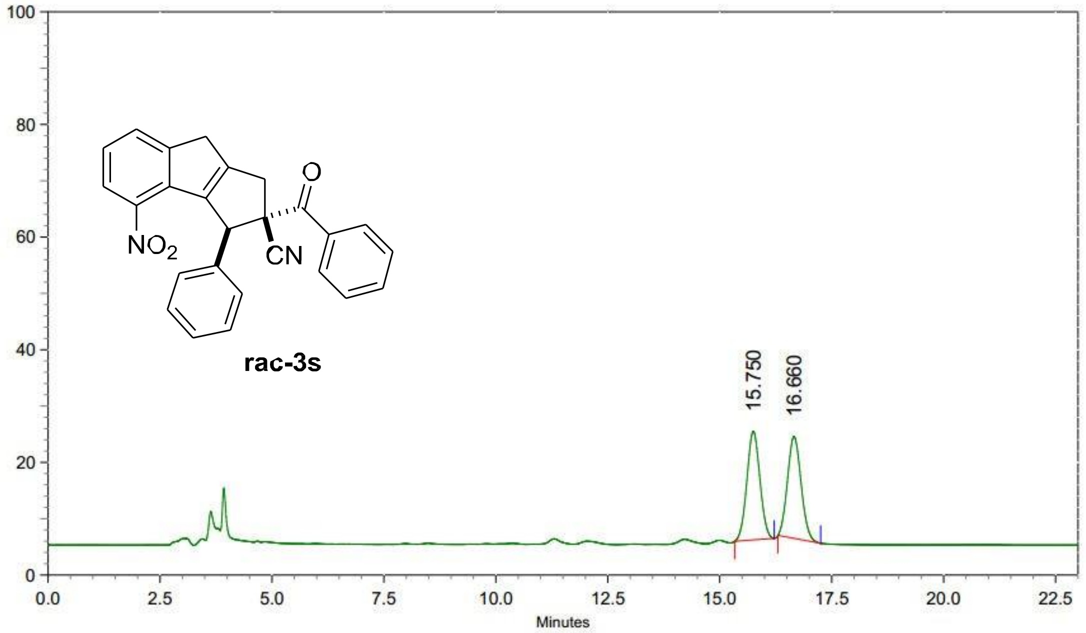

AREA PERCENT REPORT

Peak No.	Ret Time	Width	Height	Area	Area [%]
1	15.750	0.877	323440	6548981	50.5386
2	16.660	0.953	303604	6409392	49.4614

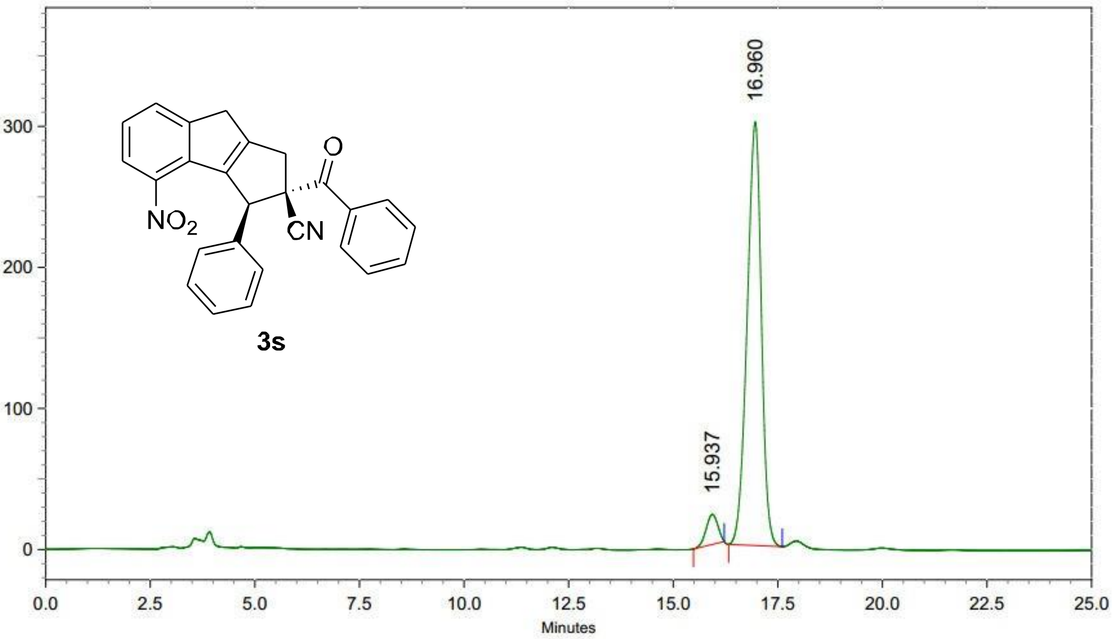

AREA PERCENT REPORT

Peak No.	Ret Time	Width	Height	Area	Area [%]
1	15.937	0.737	357278	6930989	5.4934
2	16.960	1.280	5037079	119237371	94.5066

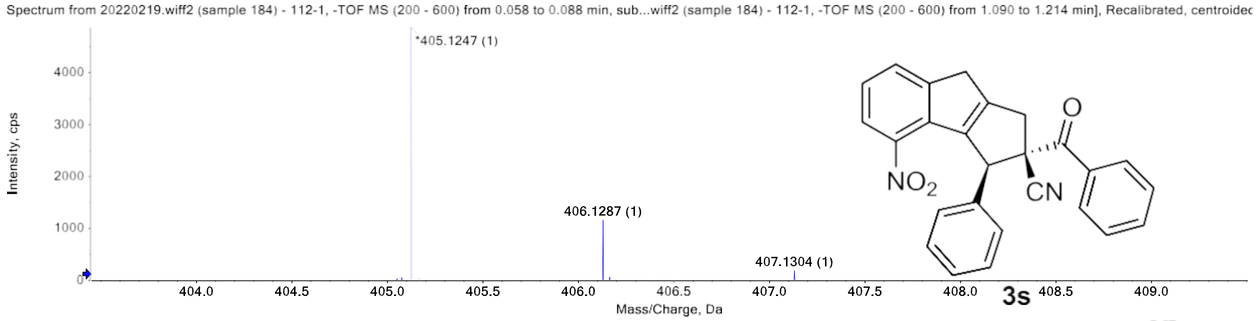

3s

HRMS (ESI-TOF) m/z: [M – [illegible]]$^-$

Calcd for $C_{26}H_{17}N_2O_3$ 405.1250

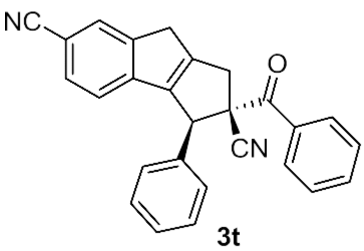

3t

^{13}C NMR (100 MHz, $CDCl_3$)

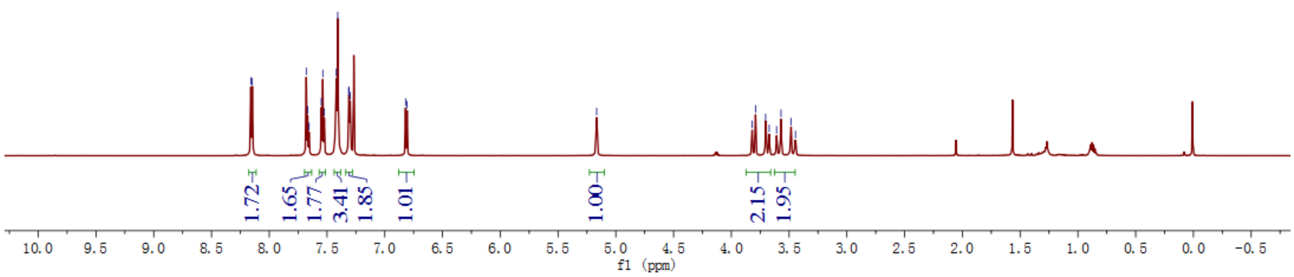

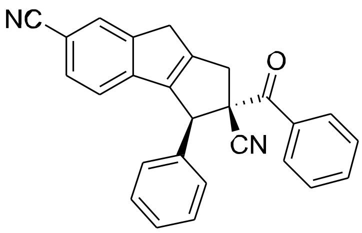

3t

^{1}H NMR (400 MHz, $CDCl_3$)

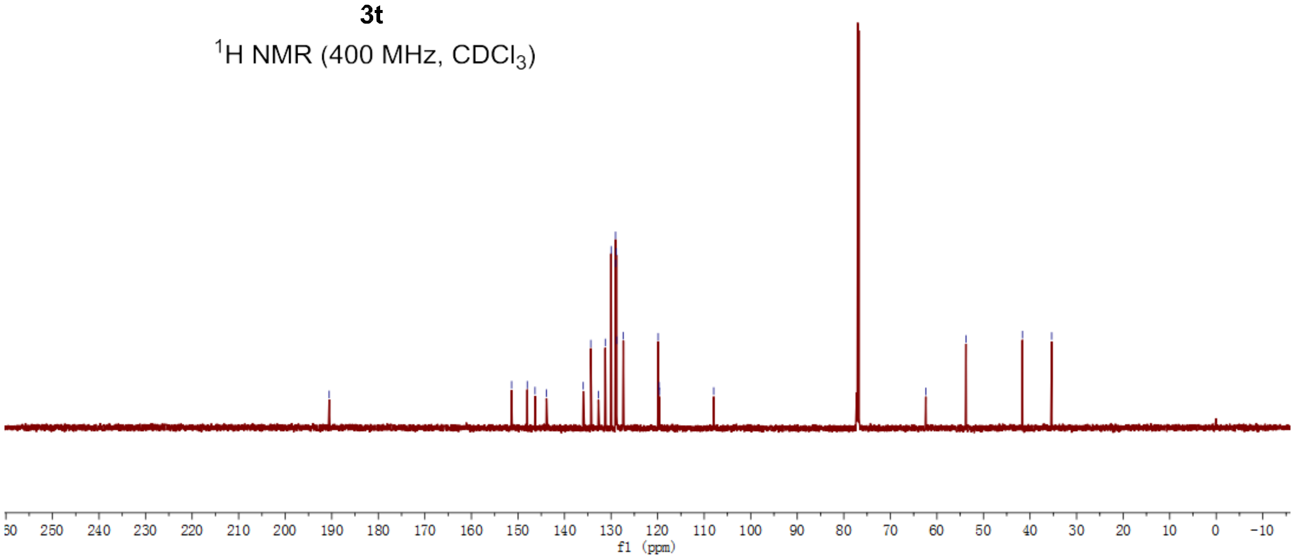

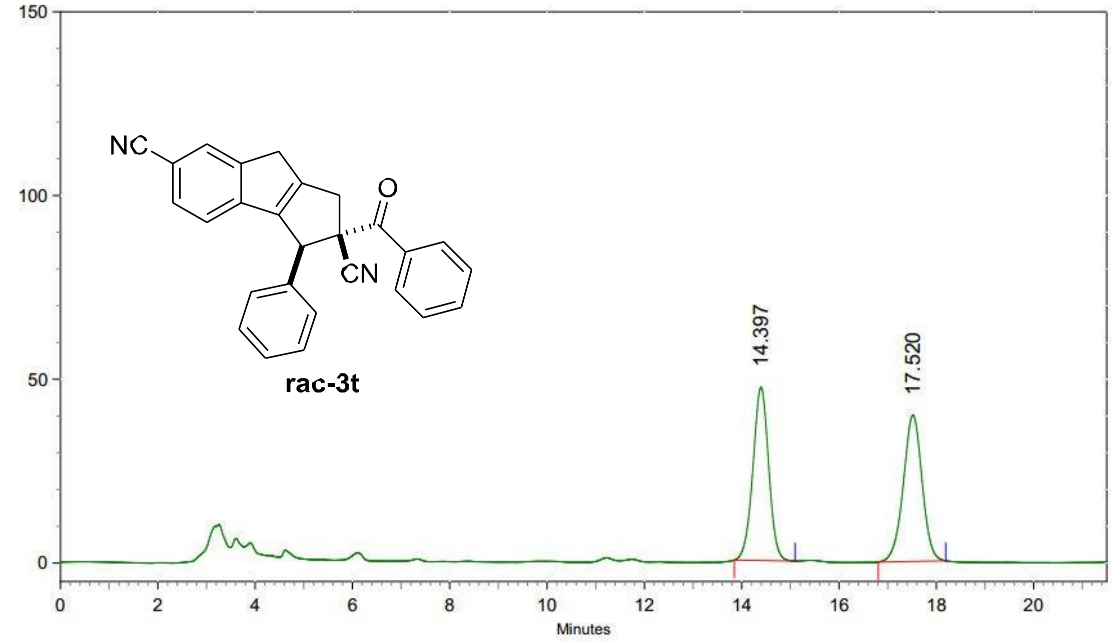

AREA PERCENT REPORT

Peak No.	Ret Time	Width	Height	Area	Area [%]
1	14.397	1.250	790811	17832981	49.8193
2	17.520	1.387	668040	17962342	50.1807

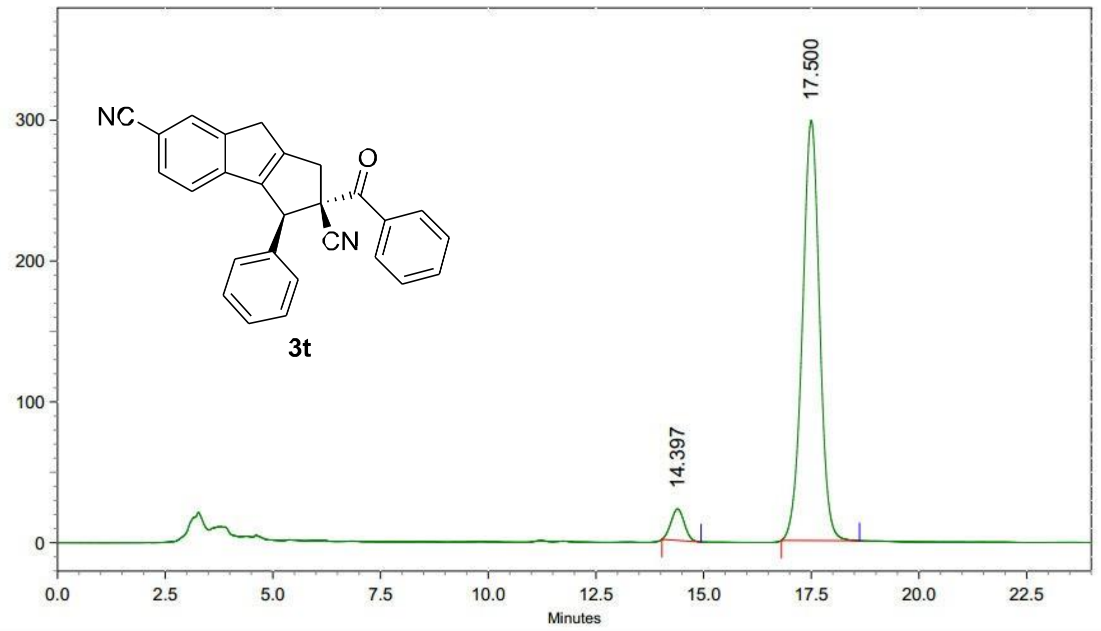

AREA PERCENT REPORT

Peak No.	Ret Time	Width	Height	Area	Area [%]
1	14.397	0.907	374621	8037502	5.4937
2	17.500	1.813	5003610	138265756	94.5063

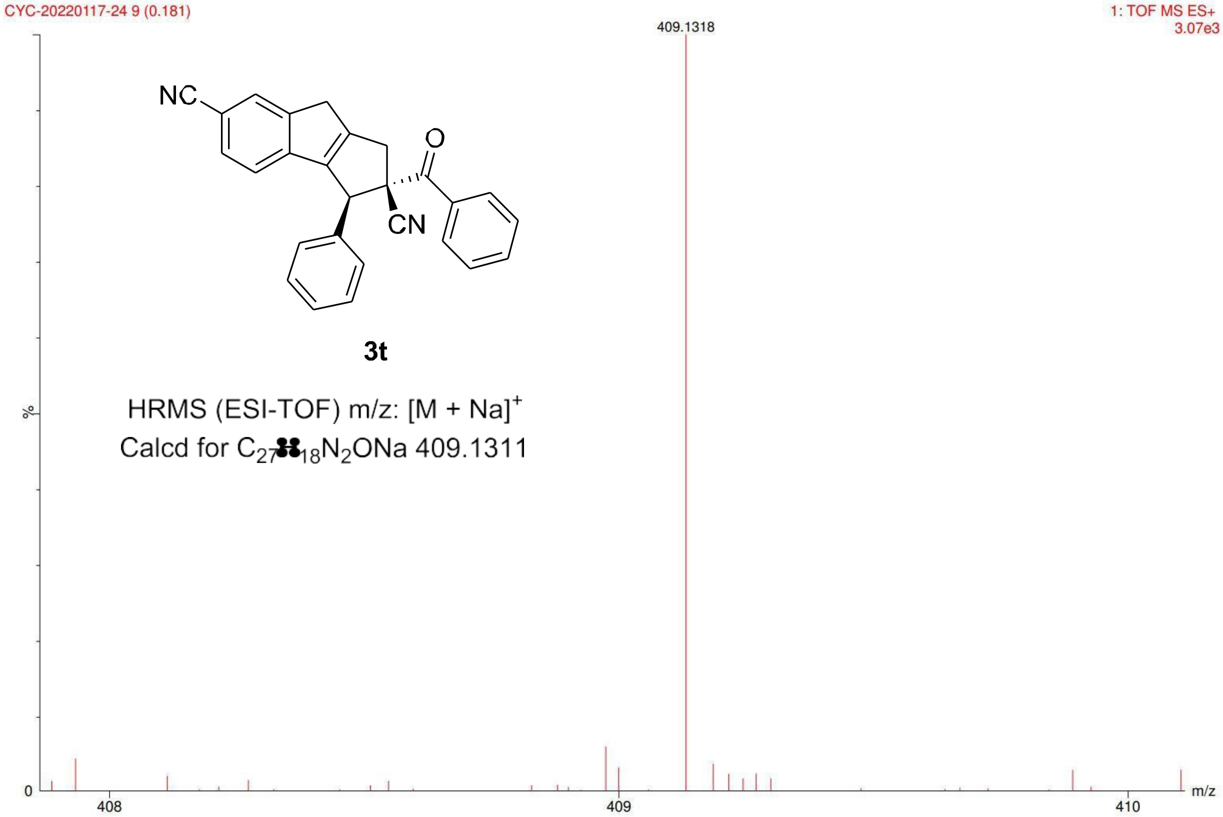
CYC-20220117-24 9 (0.181)
1: TOF MS ES+
3.07e3
409.1318
NC
O
CN
3t
HRMS (ESI-TOF) m/z: [M + Na]+
Calcd for C27[redacted]18N2ONa 409.1311
%
0
408
409
410
m/z

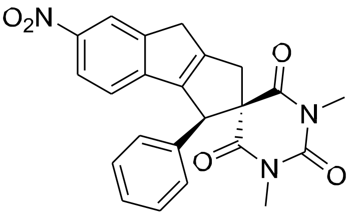

5a

^{1}H NMR (400 MHz, $CDCl_3$)

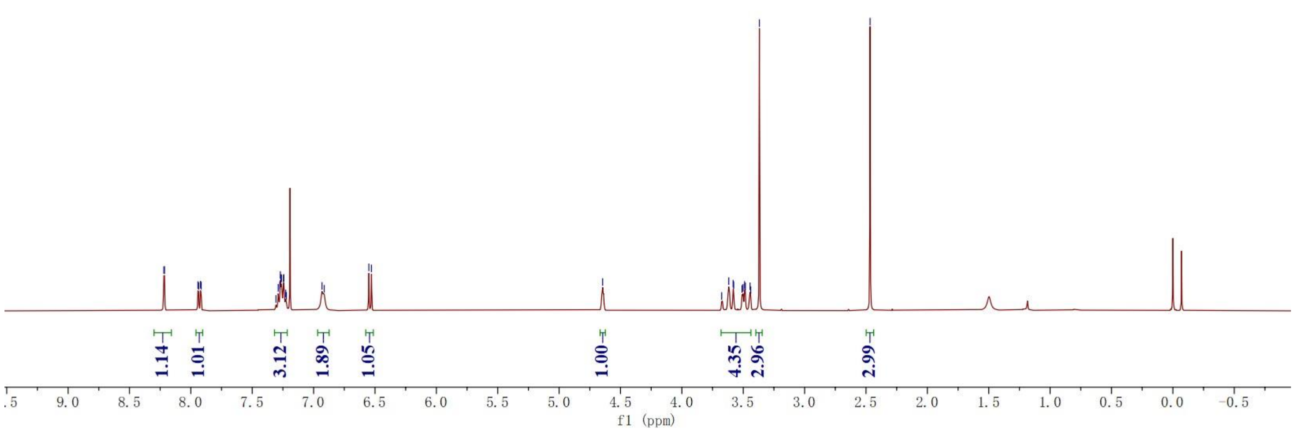

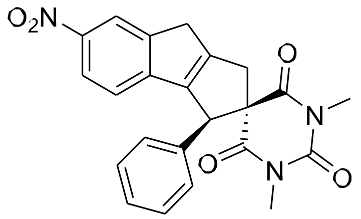

5a

^{13}C NMR (100 MHz, $CDCl_3$)

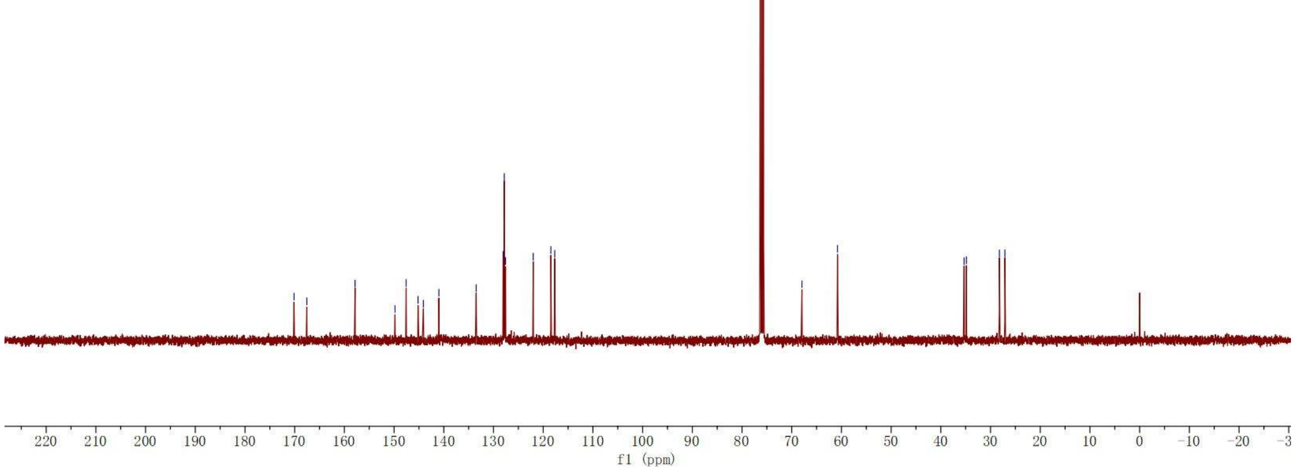

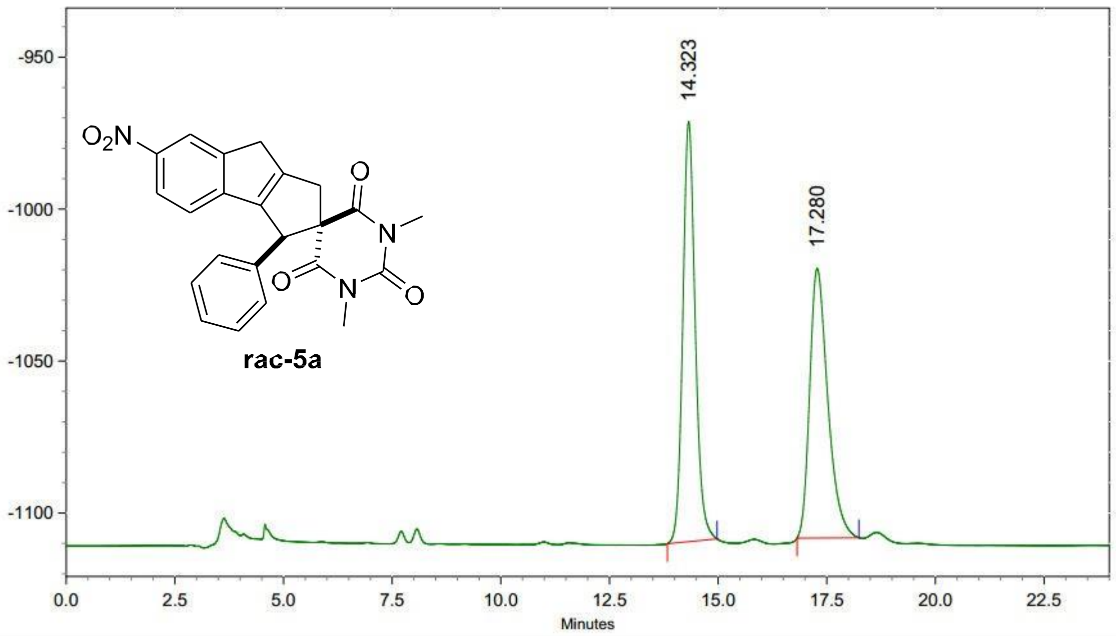

AREA PERCENT REPORT

Peak No.	Ret Time	Width	Height	Area	Area [%]
1	14.323	1.143	2318072	45990372	51.6594
2	17.280	1.423	1487545	43035705	48.3406

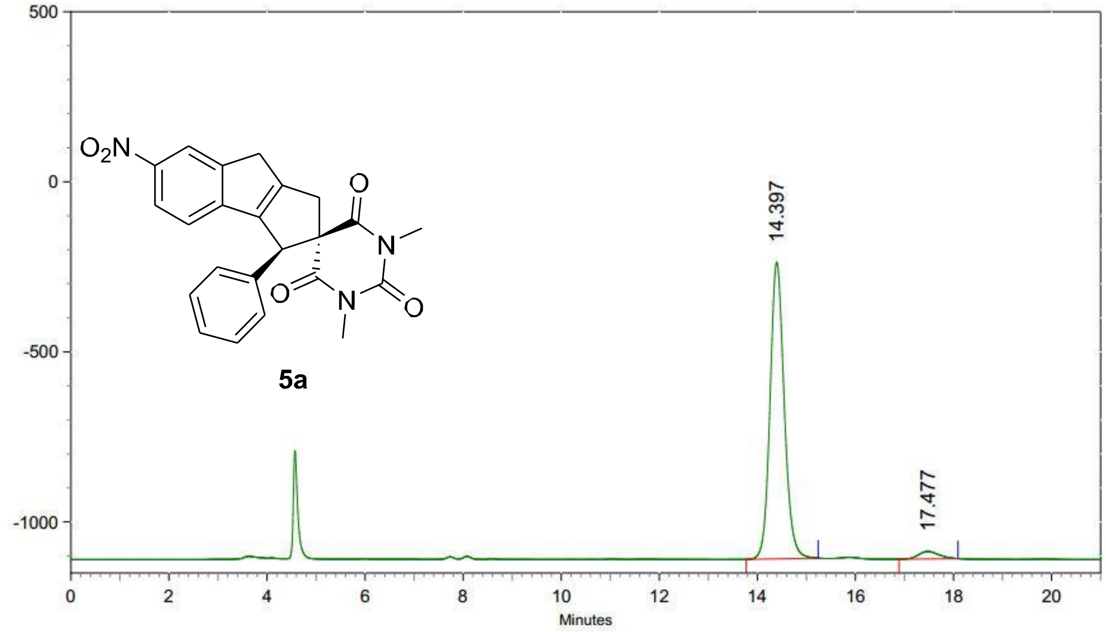

AREA PERCENT REPORT

Peak No.	Ret Time	Width	Height	Area	Area [%]
1	14.397	1.467	14625986	293562871	96.5107
2	17.477	1.203	369676	10613487	3.4893

Spectrum Plot Report

Name	CYC-20200824-25	Rack Pos.		Instrument	Instrument 1	Operator	
Inj. Vol. (ul)	10	Plate Pos.		IRM Status	Success		
Data File	CYC-20200824-25.d	Method (Acq)	TOF.m	Comment		Acq. Time (Local)	8/27/2020 2:37:37 PM (UTC+08:00)

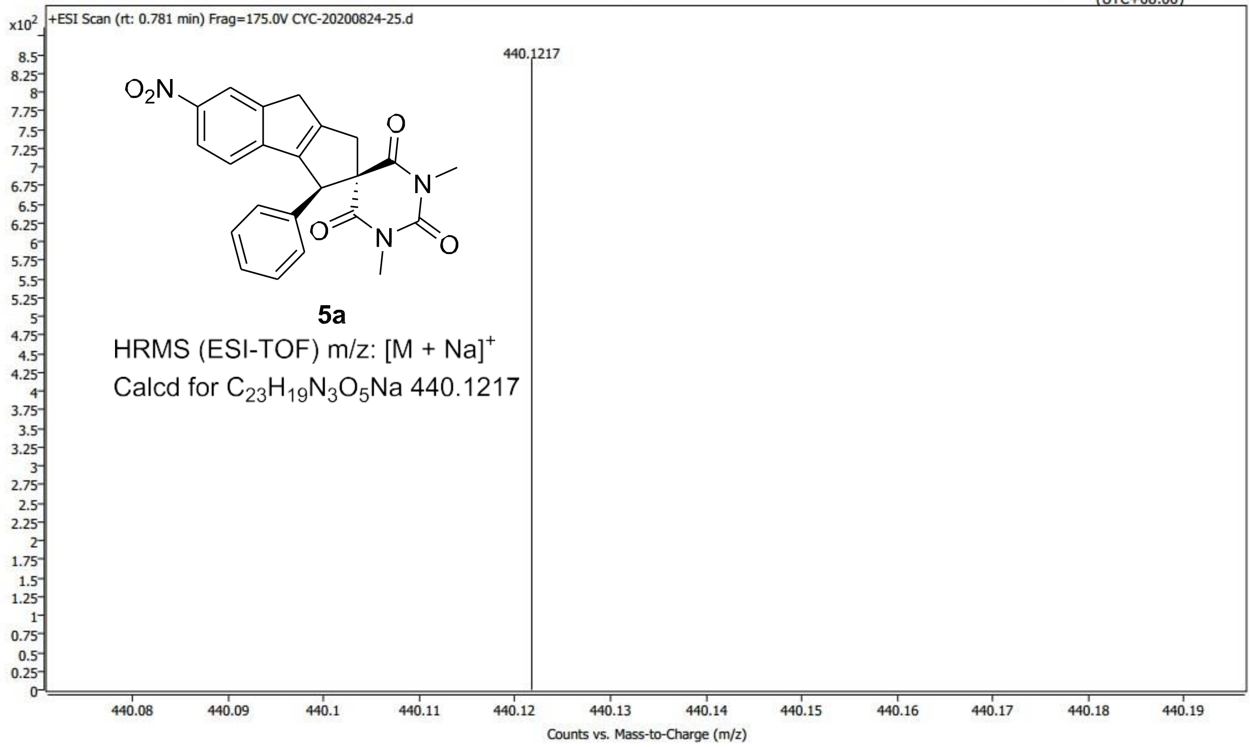

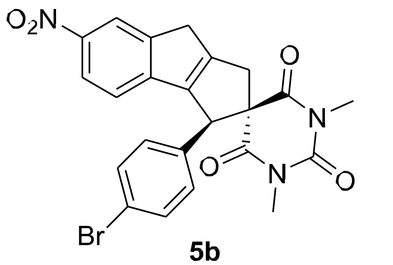

5b

^{1}H NMR (400 MHz, $CDCl_3$)

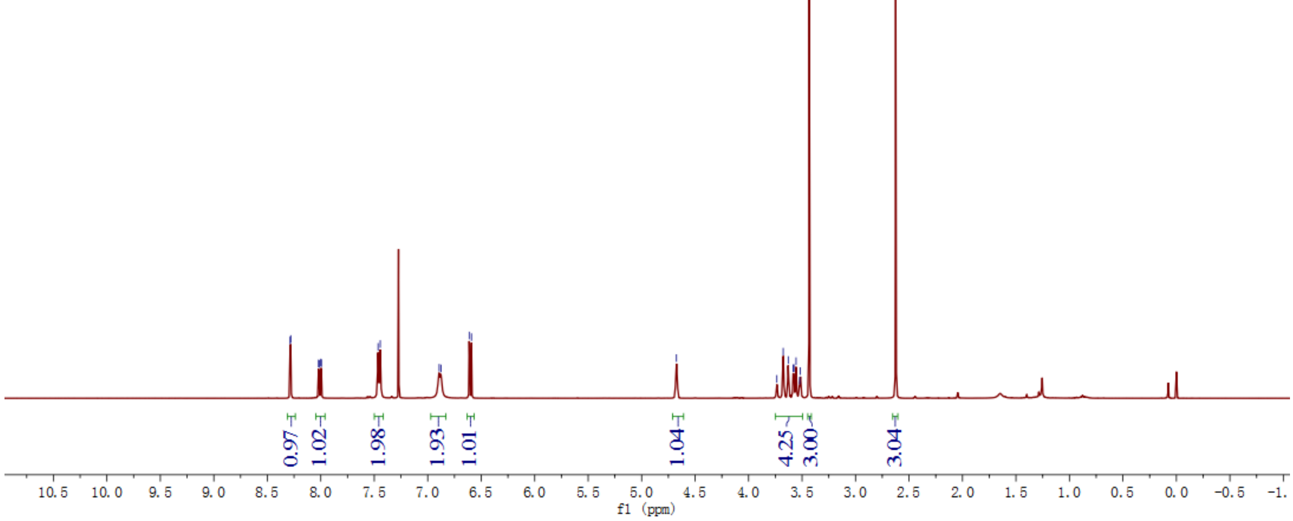

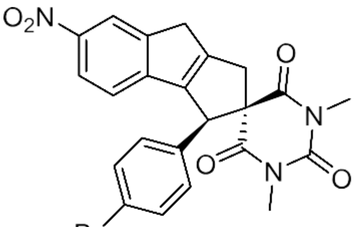

5b

^{13}C NMR (100 MHz, $CDCl_3$)

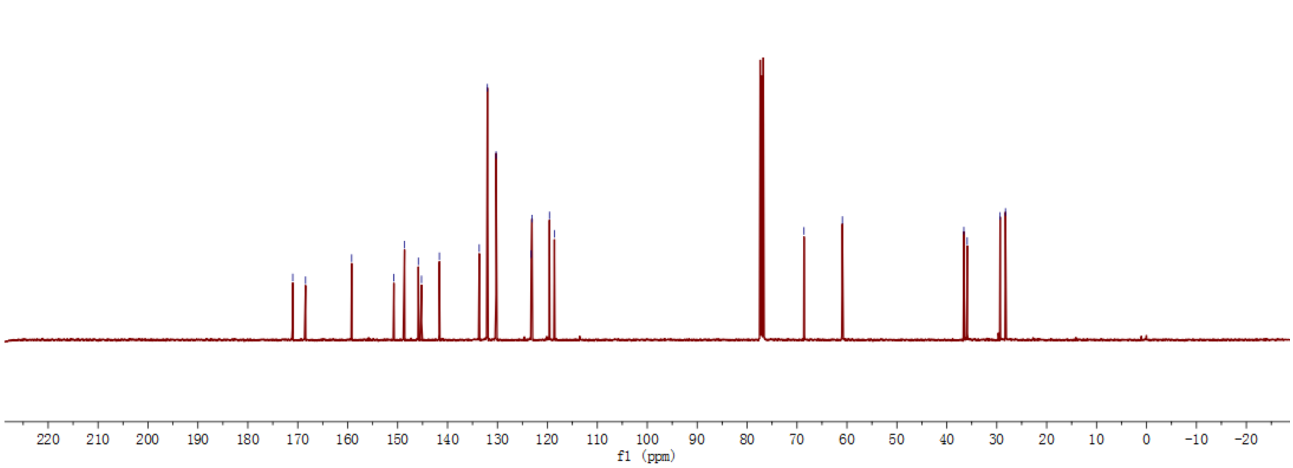

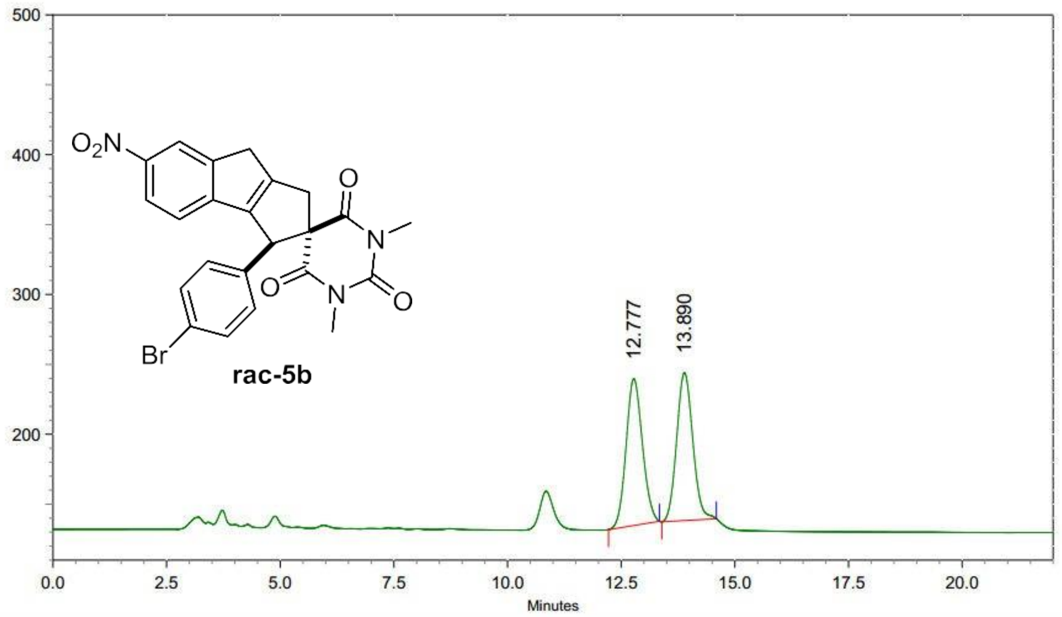

AREA PERCENT REPORT

Peak No.	Ret Time	Width	Height	Area	Area [%]
1	12.777	1.120	1762208	44032693	50.1633
2	13.890	1.200	1774725	43745957	49.8367

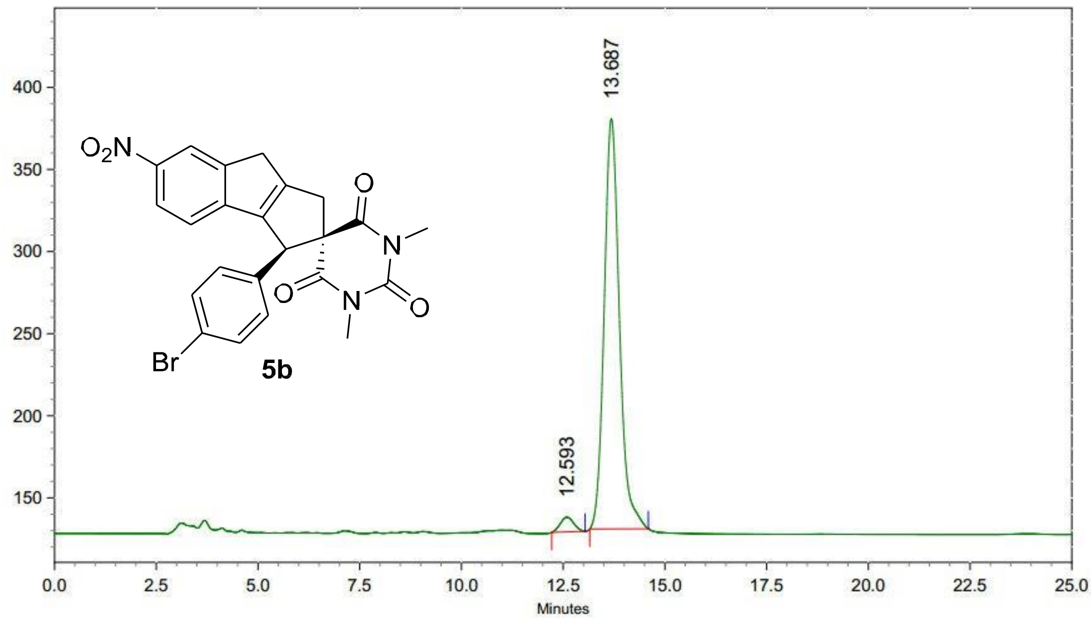

AREA PERCENT REPORT

Peak No.	Ret Time	Width	Height	Area	Area [%]
1	12.593	0.820	149833	3452028	3.0683
2	13.687	1.440	4190582	109055039	96.9317

Spectrum Plot Report

Name	CYC-20200824-26	Rack Pos.		Instrument	Instrument 1	Operator	
Inj. Vol. (ul)	10	Plate Pos.		IRM Status	Success		
Data File	CYC-20200824-26.d	Method (Acq)	TOF.m	Comment		Acq. Time (Local)	8/27/2020 2:40:36 PM (UTC+08:00)

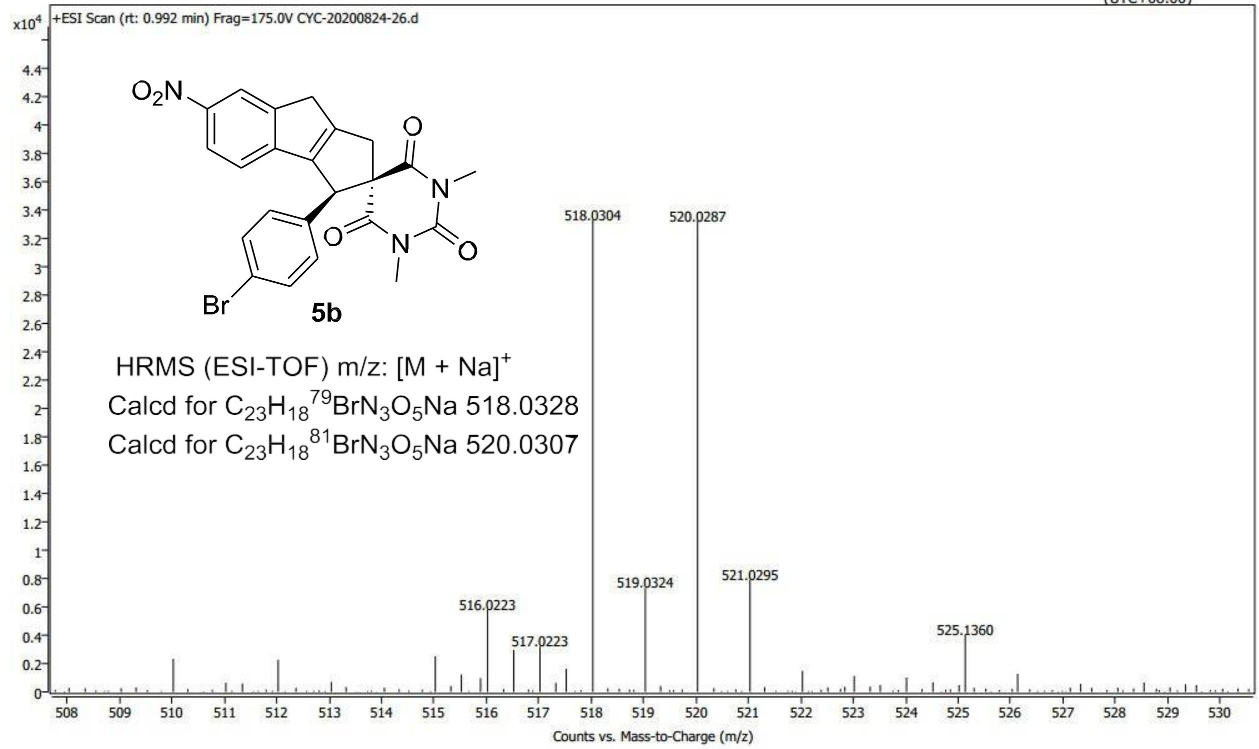

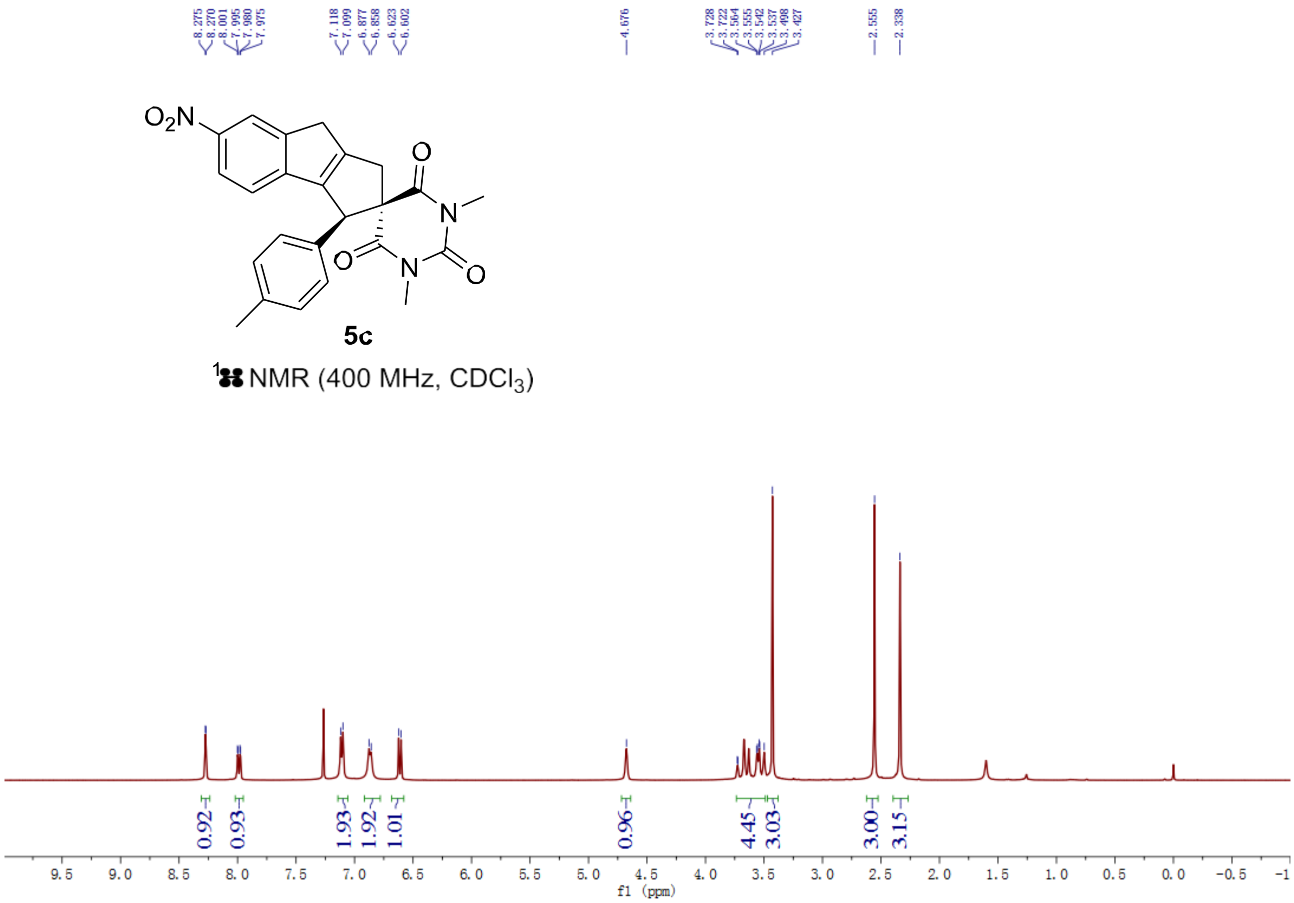

5c

^{1}H NMR (400 MHz, $CDCl_3$)

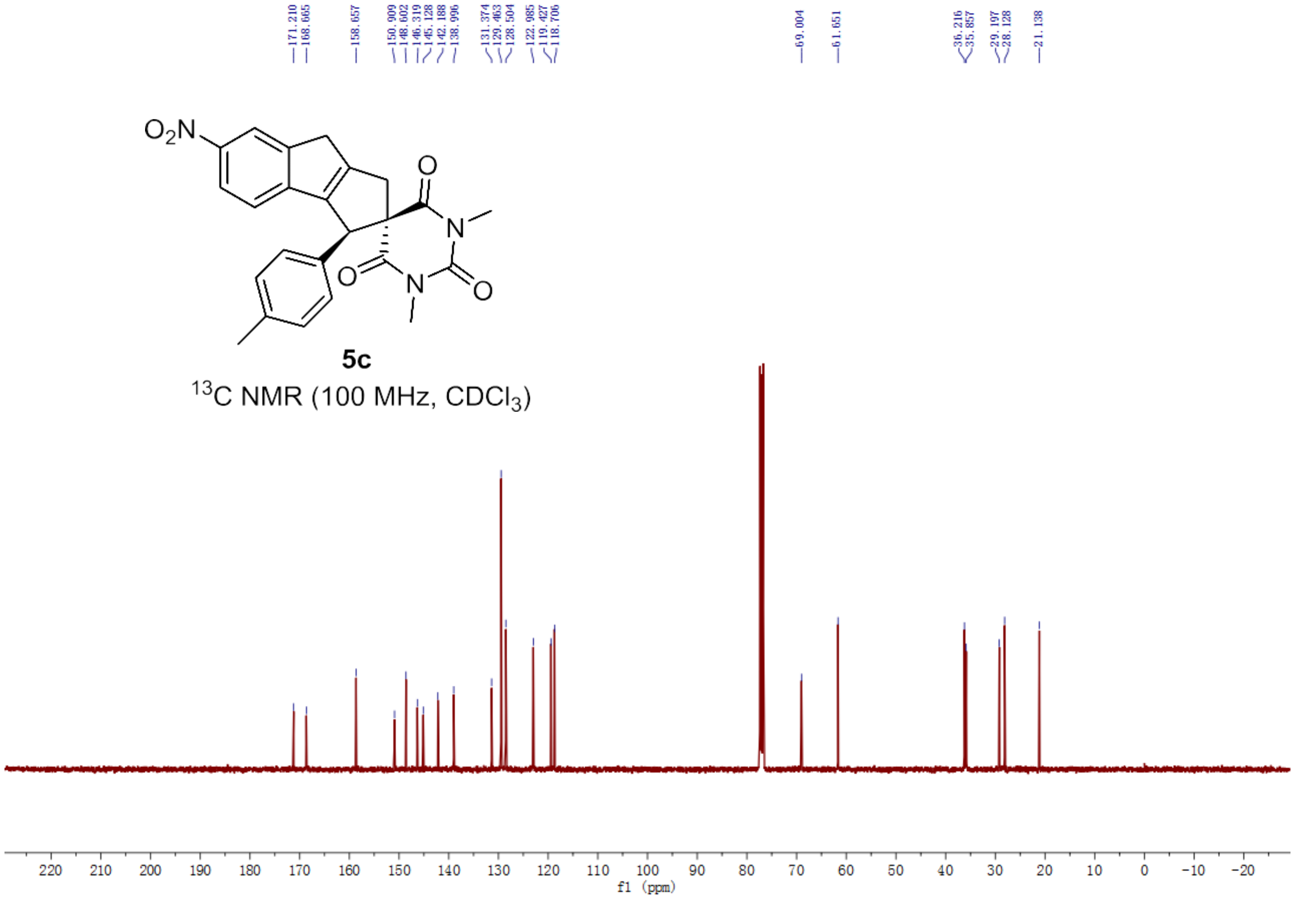

5c

^{13}C NMR (100 MHz, $CDCl_3$)

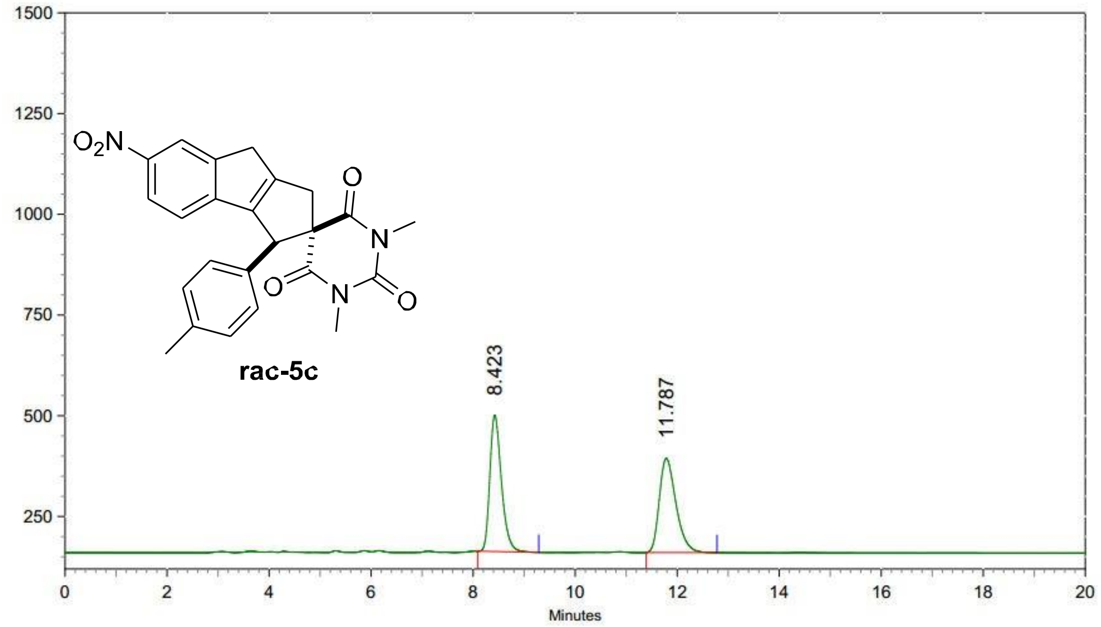

AREA PERCENT REPORT

Peak No.	Ret Time	Width	Height	Area	Area [%]
1	8.423	1.200	5678778	89672989	50.2062
2	11.787	1.387	3921789	88936385	49.7938

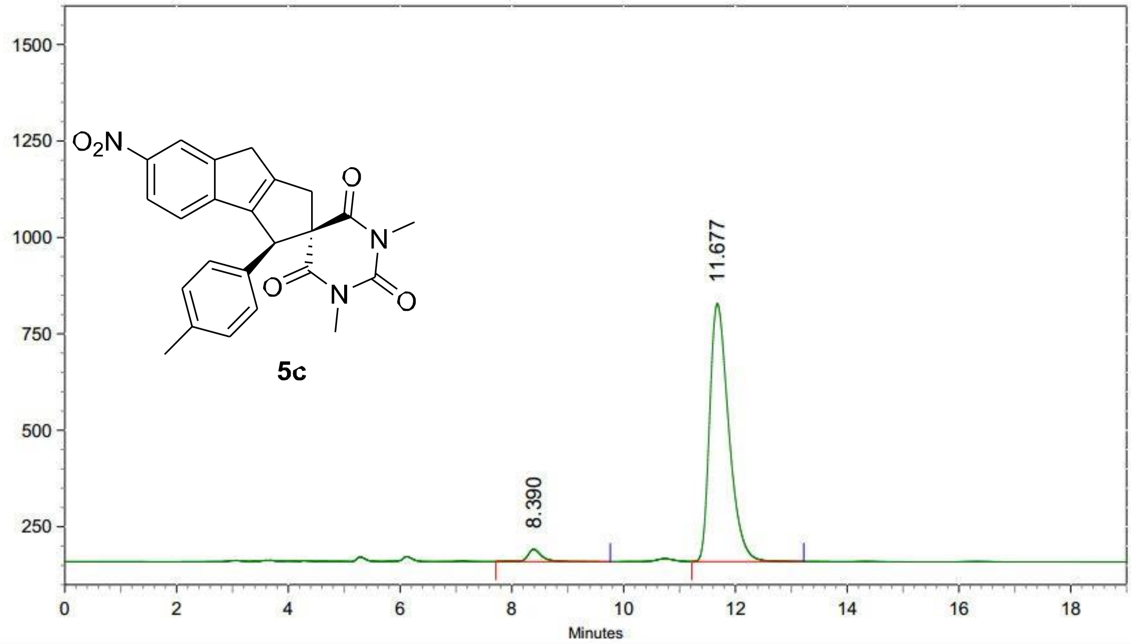

AREA PERCENT REPORT

Peak No.	Ret Time	Width	Height	Area	Area [%]
1	8.390	2.047	540551	9743247	3.5409
2	11.677	2.003	11214717	265421495	96.4591

Spectrum Plot Report

Name	CYC-20200824-29	Rack Pos.		Instrument	Instrument 1	Operator	
Inj. Vol. (ul)	10	Plate Pos.		IRM Status	Success		
Data File	CYC-20200824-29.d	Method (Acq)	TOF.m	Comment		Acq. Time (Local)	8/27/2020 2:49:36 PM (UTC+08:00)

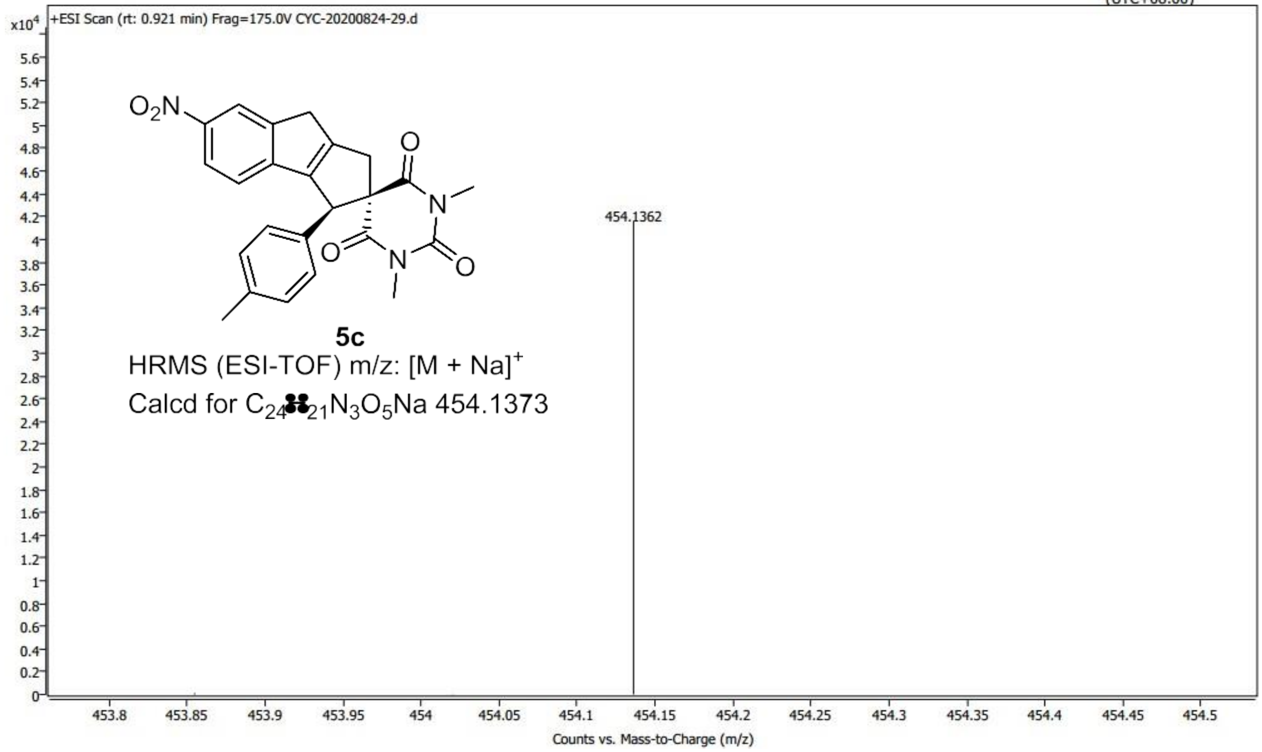

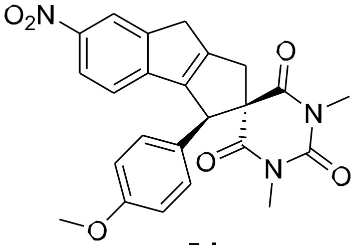

5d

^{1}H NMR (400 MHz, $CDCl_3$)

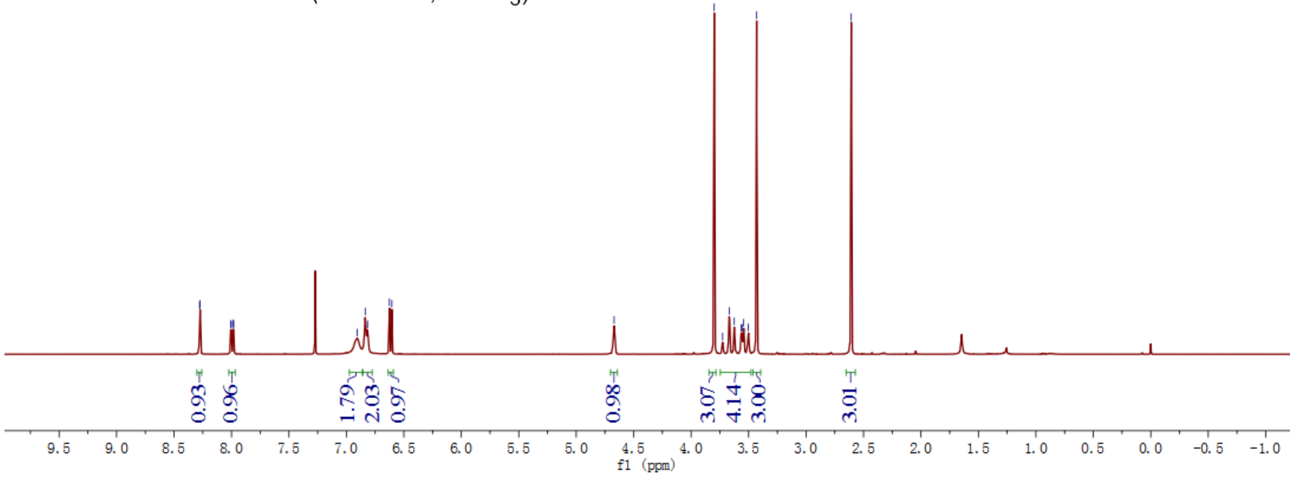

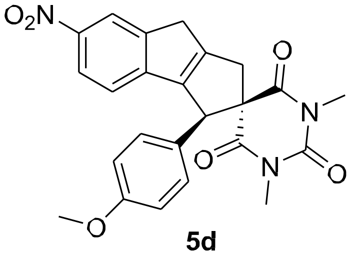

5d

^{13}C NMR (100 MHz, $CDCl_3$)

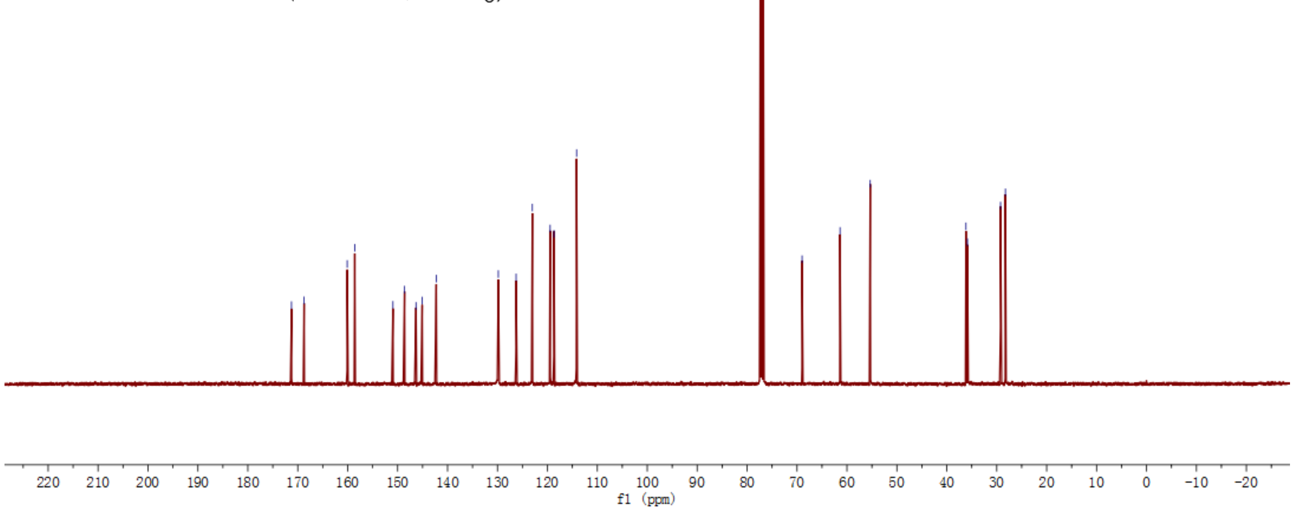

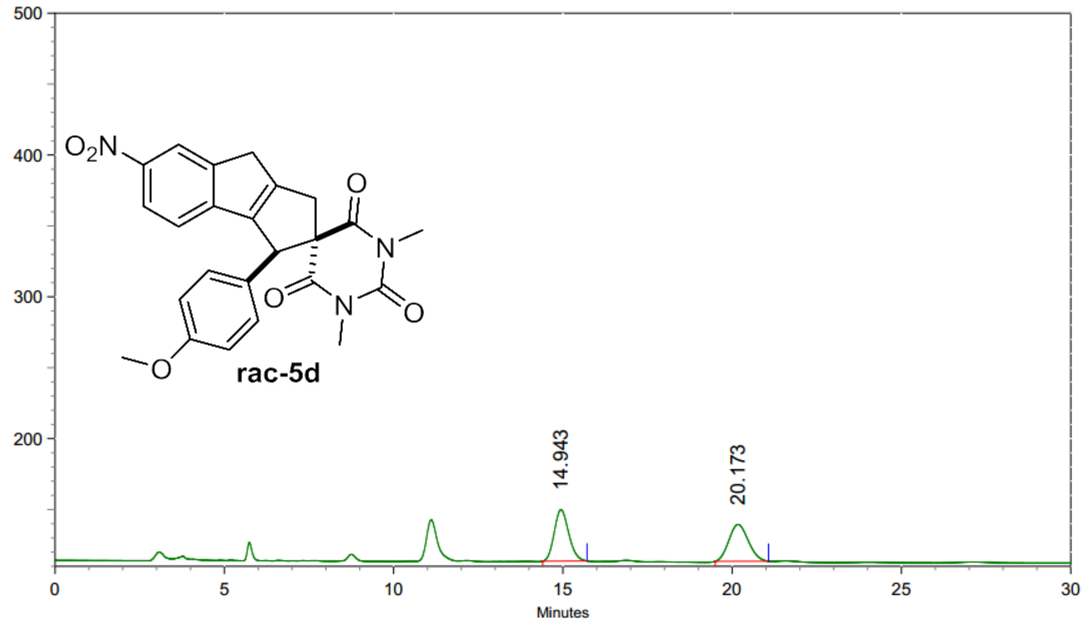

AREA PERCENT REPORT

Peak No.	Ret Time	Width	Height	Area	Area [%]
1	14.943	1.307	608903	18110113	51.0009
2	20.173	1.573	435975	17399257	48.9991

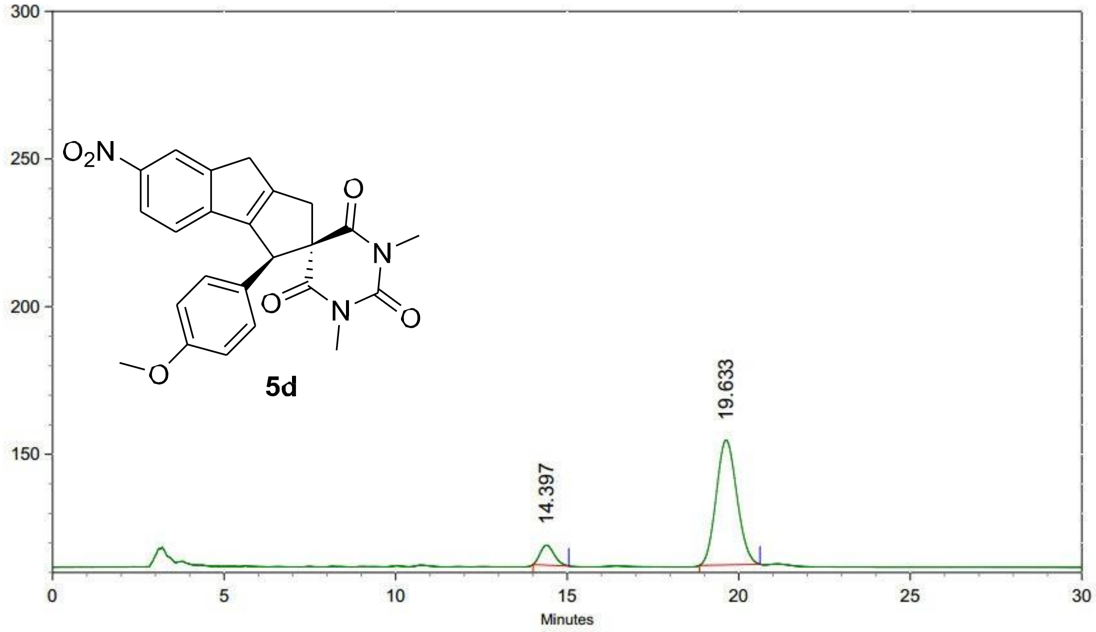

AREA PERCENT REPORT

Peak No.	Ret Time	Width	Height	Area	Area [%]
1	14.397	1.040	113070	3190962	9.6905
2	19.633	1.760	709381	29737678	90.3095

Spectrum Plot Report

Name	CYC-20200824-27	Rack Pos.		Instrument	Instrument 1	Operator	
Inj. Vol. (ul)	10	Plate Pos.		IRM Status	Success		
Data File	CYC-20200824-27.d	Method (Acq)	TOF.m	Comment		Acq. Time (Local)	8/27/2020 2:43:37 PM (UTC+08:00)

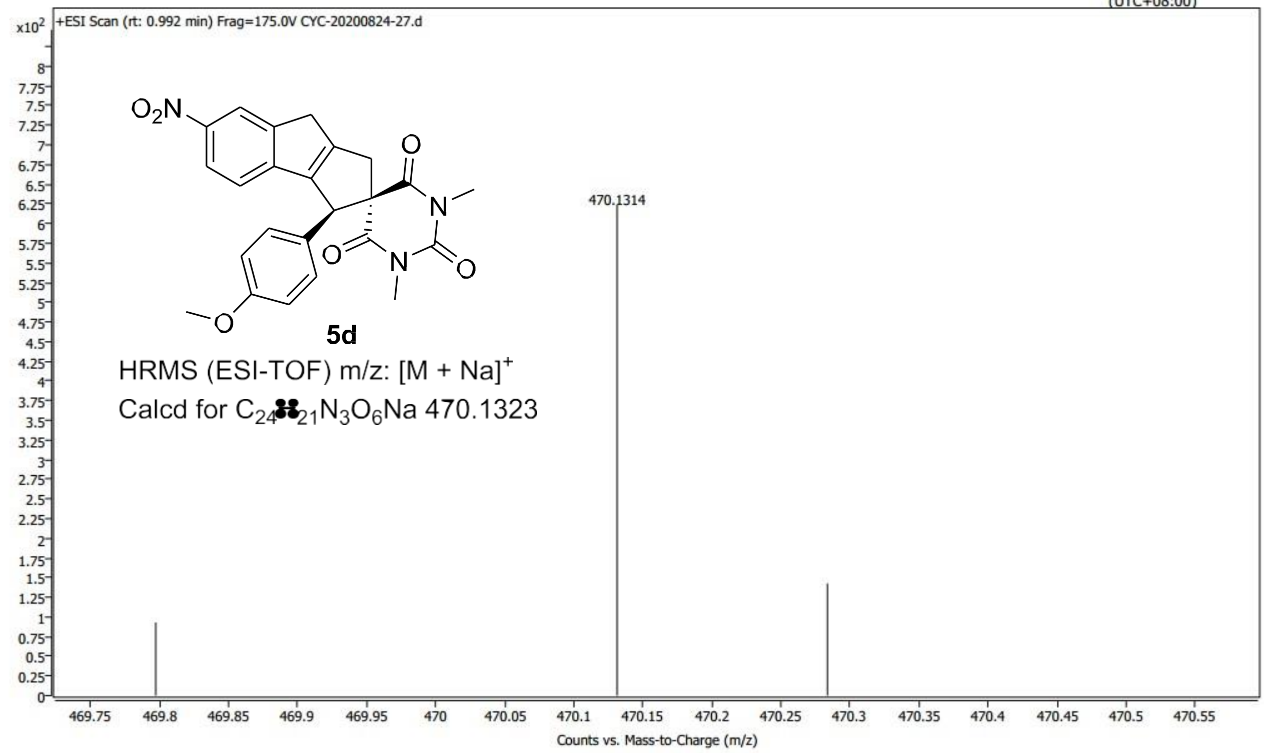

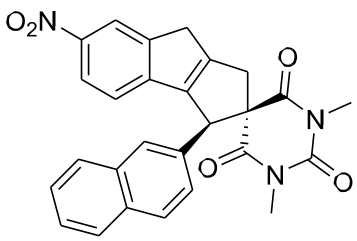

5e

^{1}H NMR (400 MHz, $CDCl_3$)

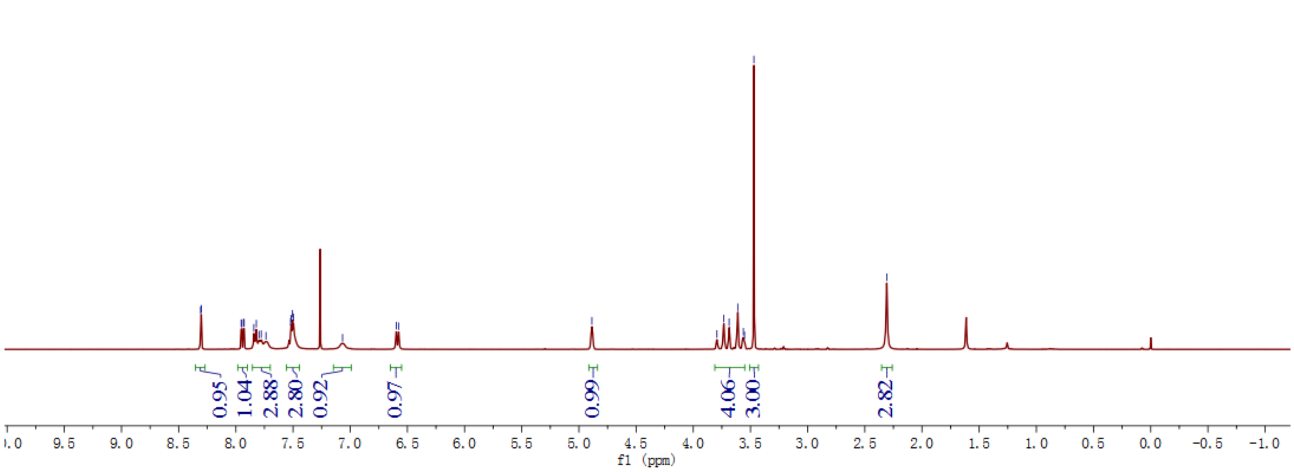

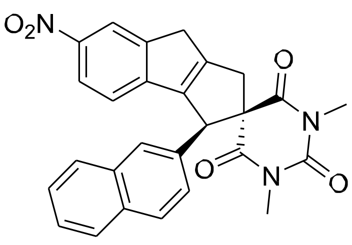

5e

^{13}C NMR (100 MHz, $CDCl_3$)

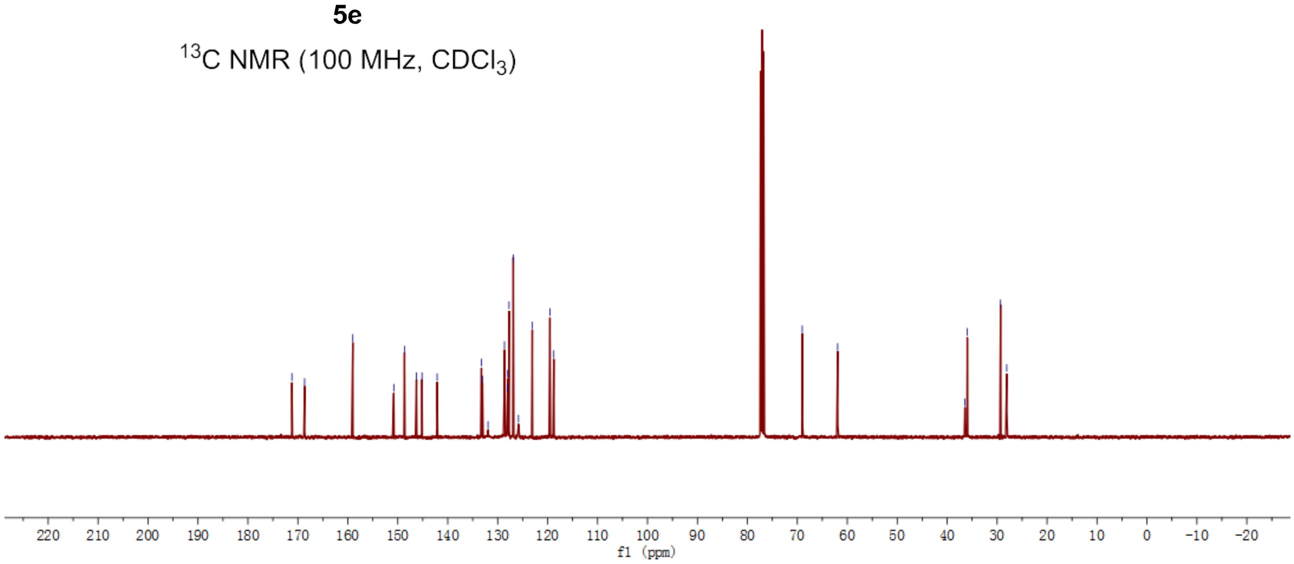

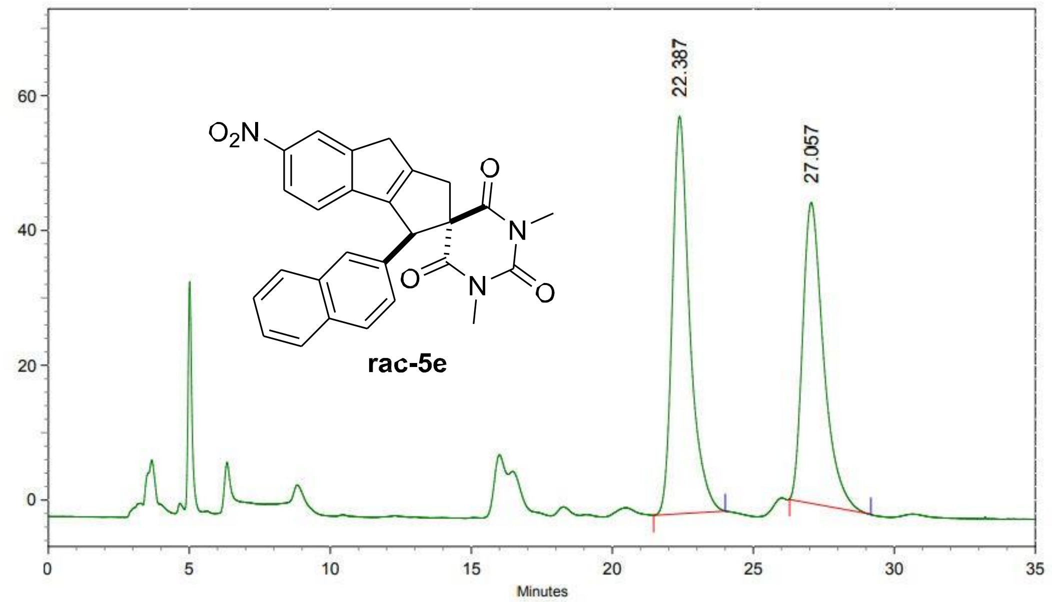

AREA PERCENT REPORT

Peak No.	Ret Time	Width	Height	Area	Area [%]
1	22.387	2.540	989286	42525262	52.4179
2	27.057	2.880	748518	38602032	47.5821

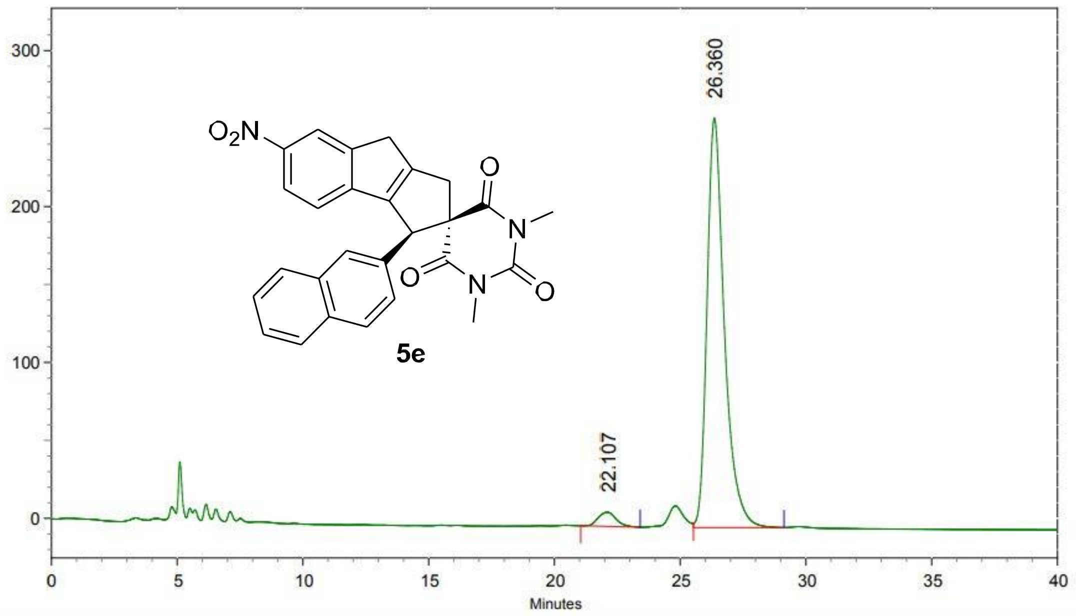

AREA PERCENT REPORT

Peak No.	Ret Time	Width	Height	Area	Area [%]
1	22.107	2.363	152557	7509188	3.4318
2	26.360	3.603	4406361	211301278	96.5682

Spectrum Plot Report

Agilent

Name	CYC-20200824-30	Rack Pos.		Instrument	Instrument 1	Operator	
Inj. Vol. (ul)	10	Plate Pos.		IRM Status	Success		
Data File	CYC-20200824-30.d	Method (Acq)	TOF.m	Comment		Acq. Time (Local)	8/27/2020 2:52:37 PM (UTC+08:00)

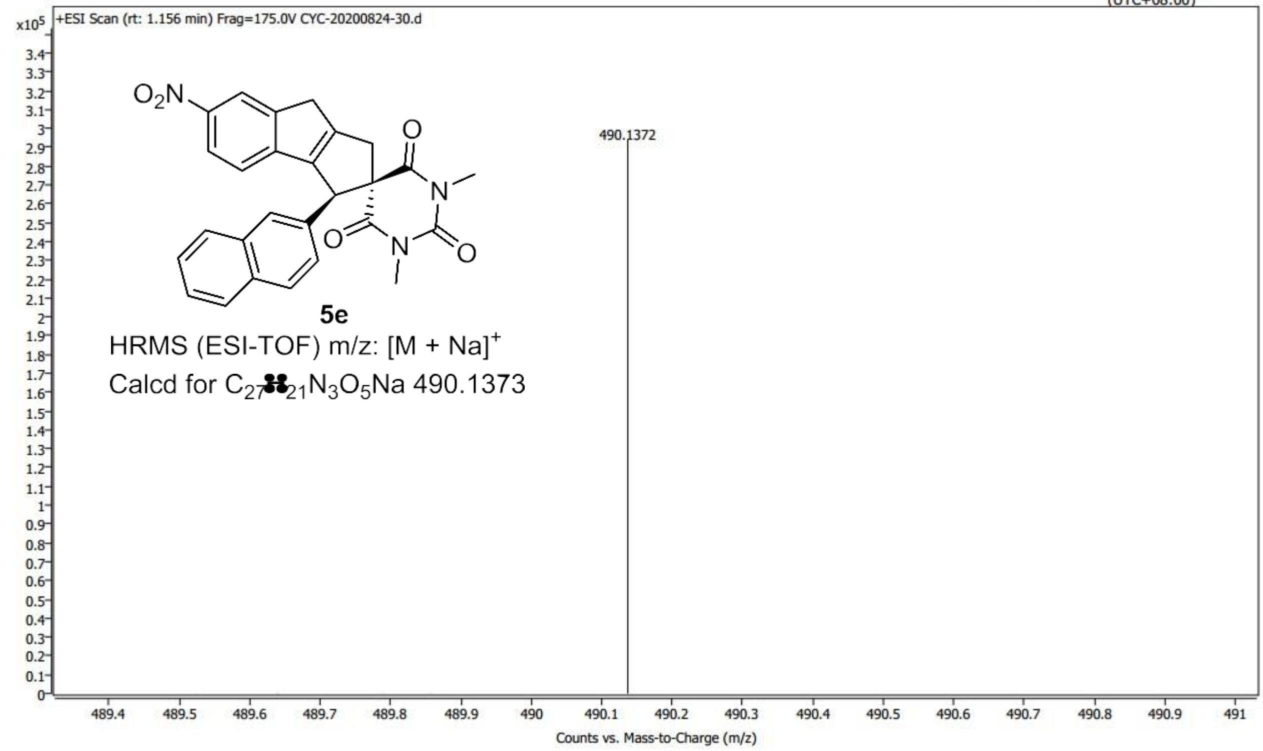

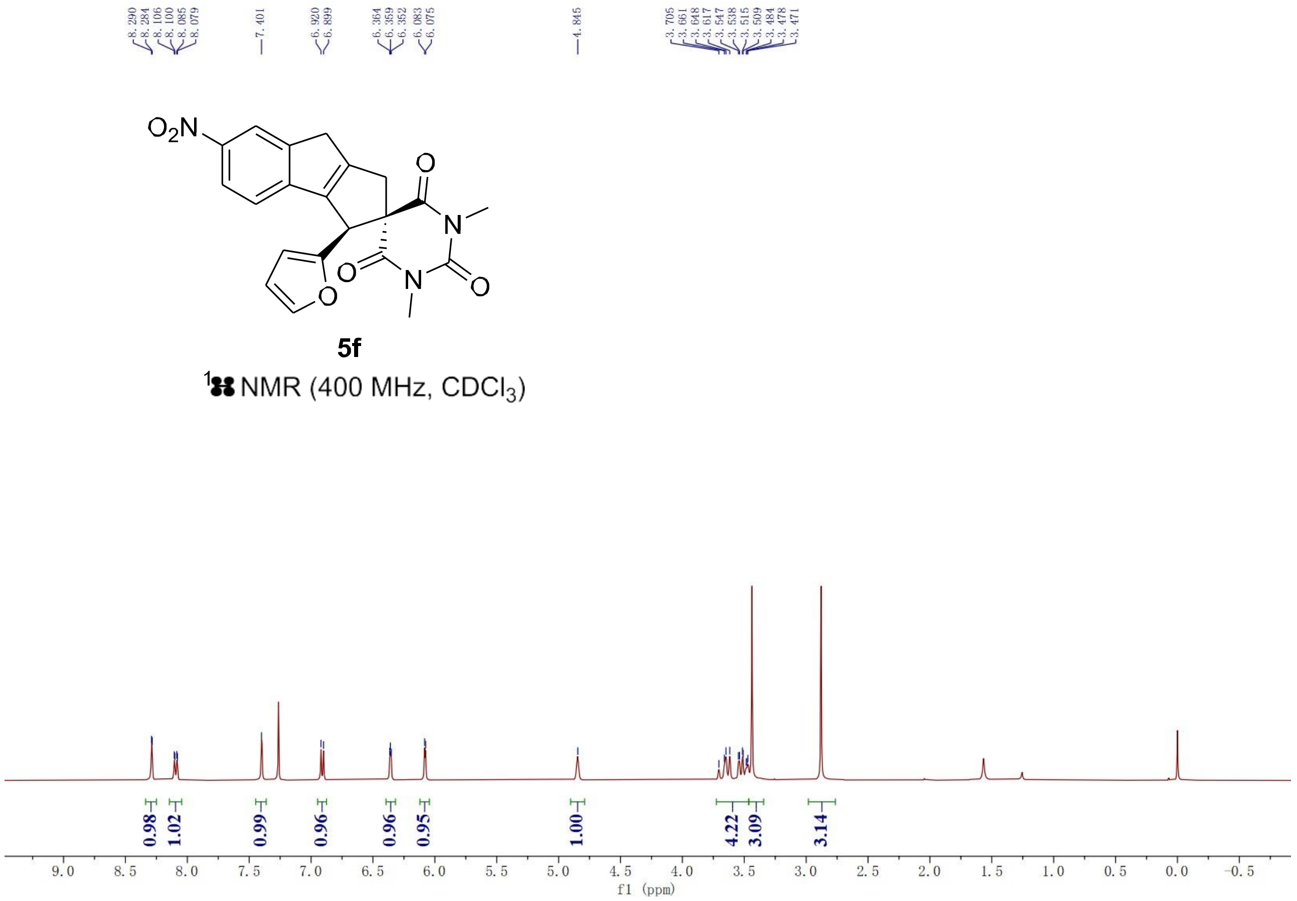

5f

^{1}H NMR (400 MHz, $CDCl_3$)

3

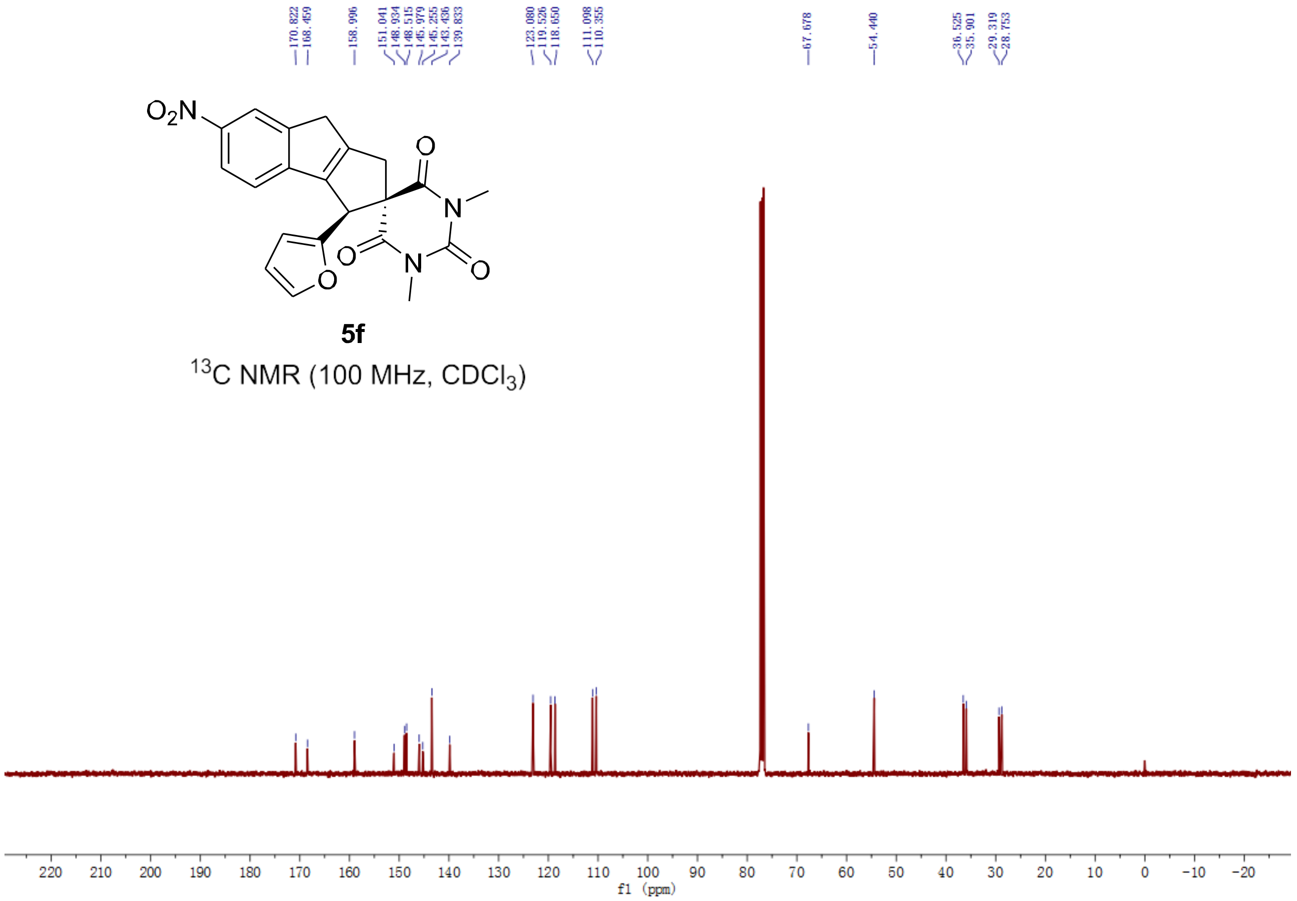

5f

^{13}C NMR (100 MHz, $CDCl_3$)

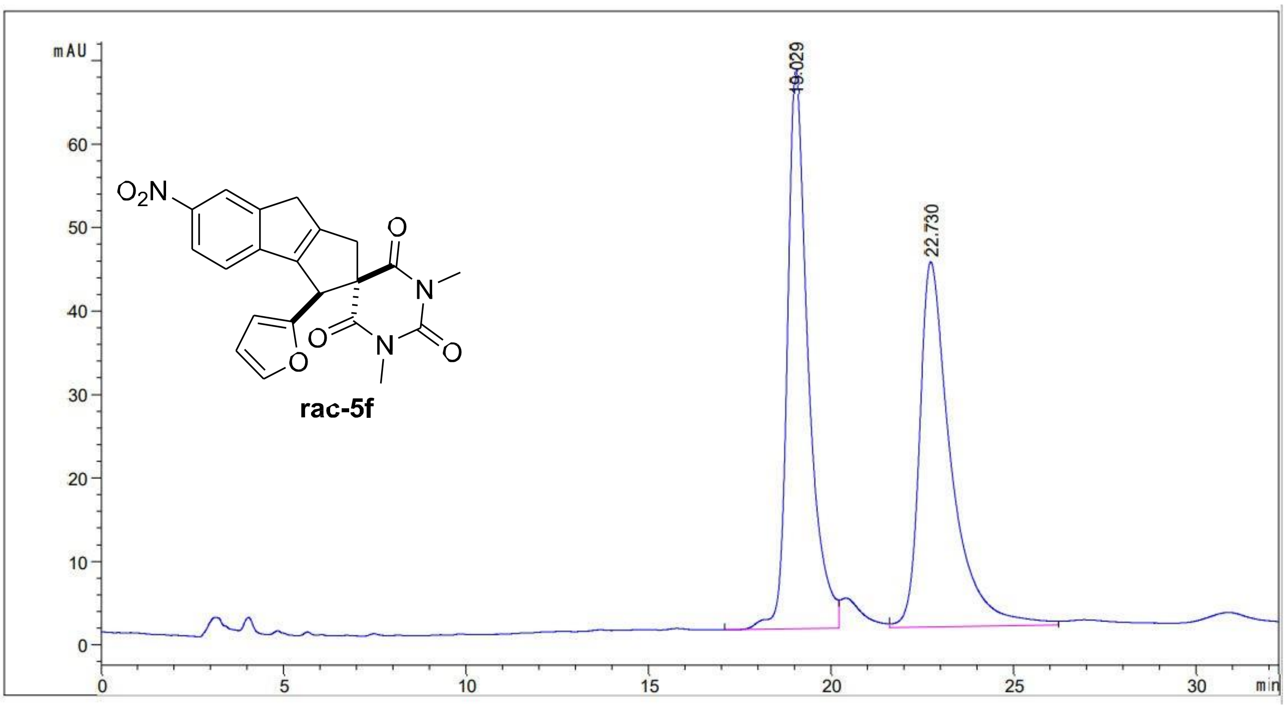

#	[min]		[min]	[mAU*s]	[mAU]	%
1	19.029	BV	0.5888	2676.49561	66.94044	50.5444
2	22.730	VV	0.8735	2618.83789	43.72339	49.4556

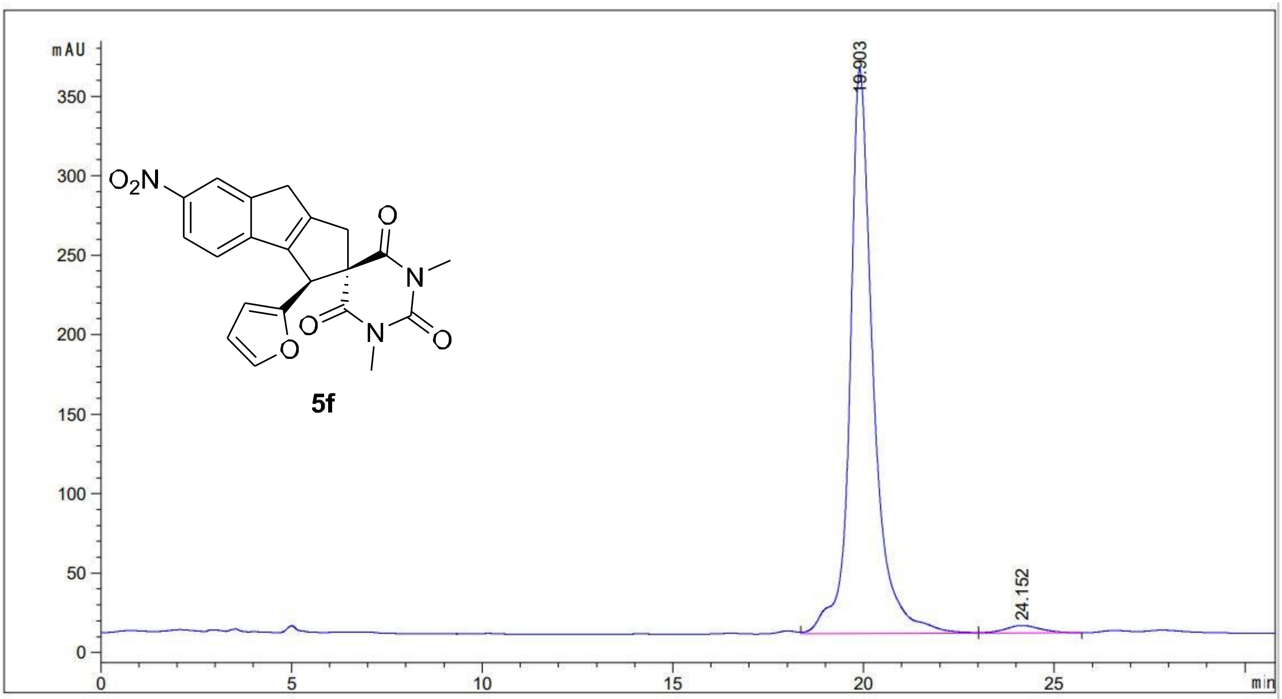

#	[min]		[min]	[mAU*s]	[mAU]	%
1	19.903	VV	0.6275	1.51928e4	354.93140	97.9882
2	24.152	VB	0.9718	311.91891	4.77469	2.0118

Spectrum Plot Report

Name	CYC-20200104-4	Rack Pos.		Instrument	Instrument 1	Operator	
Inj. Vol. (ul)	8	Plate Pos.		IRM Status	Success		
Data File	CYC-20200104-4.d	Method (Acq)	ZYJ-20201106.m	Comment		Acq. Time (Local)	1/6/2021 7:27:32 PM (UTC+08:00)

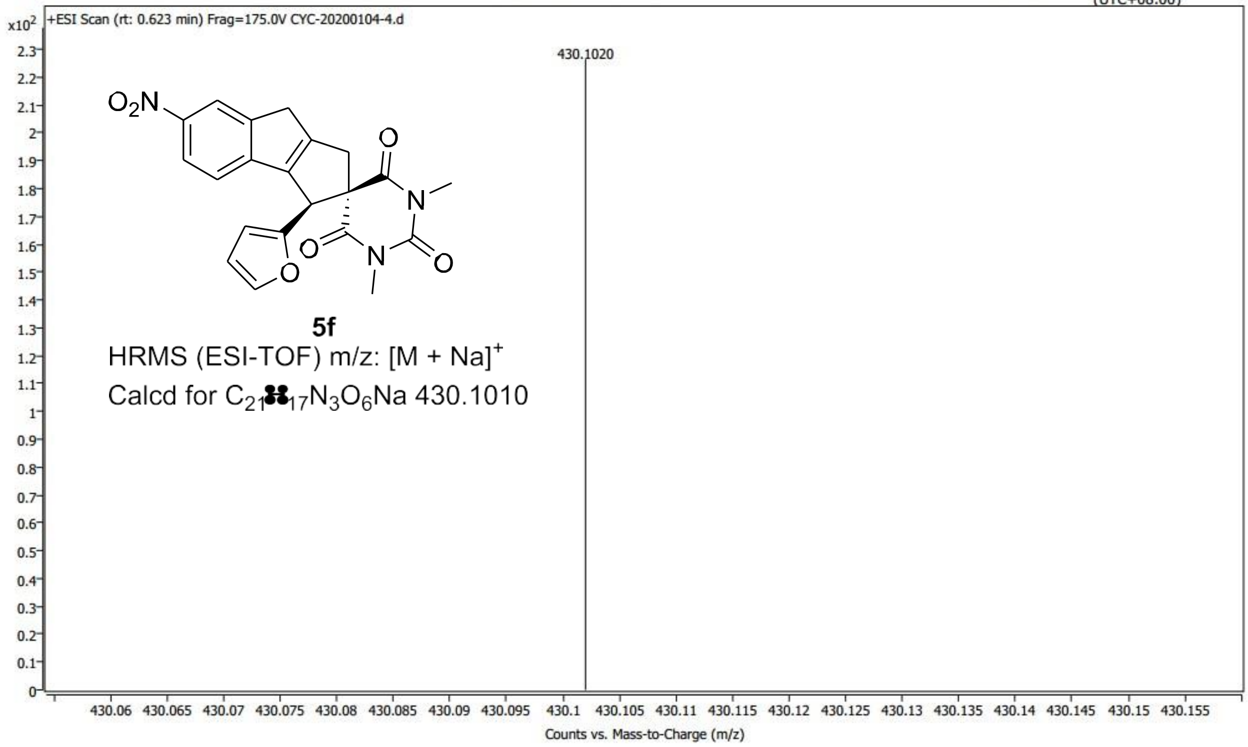

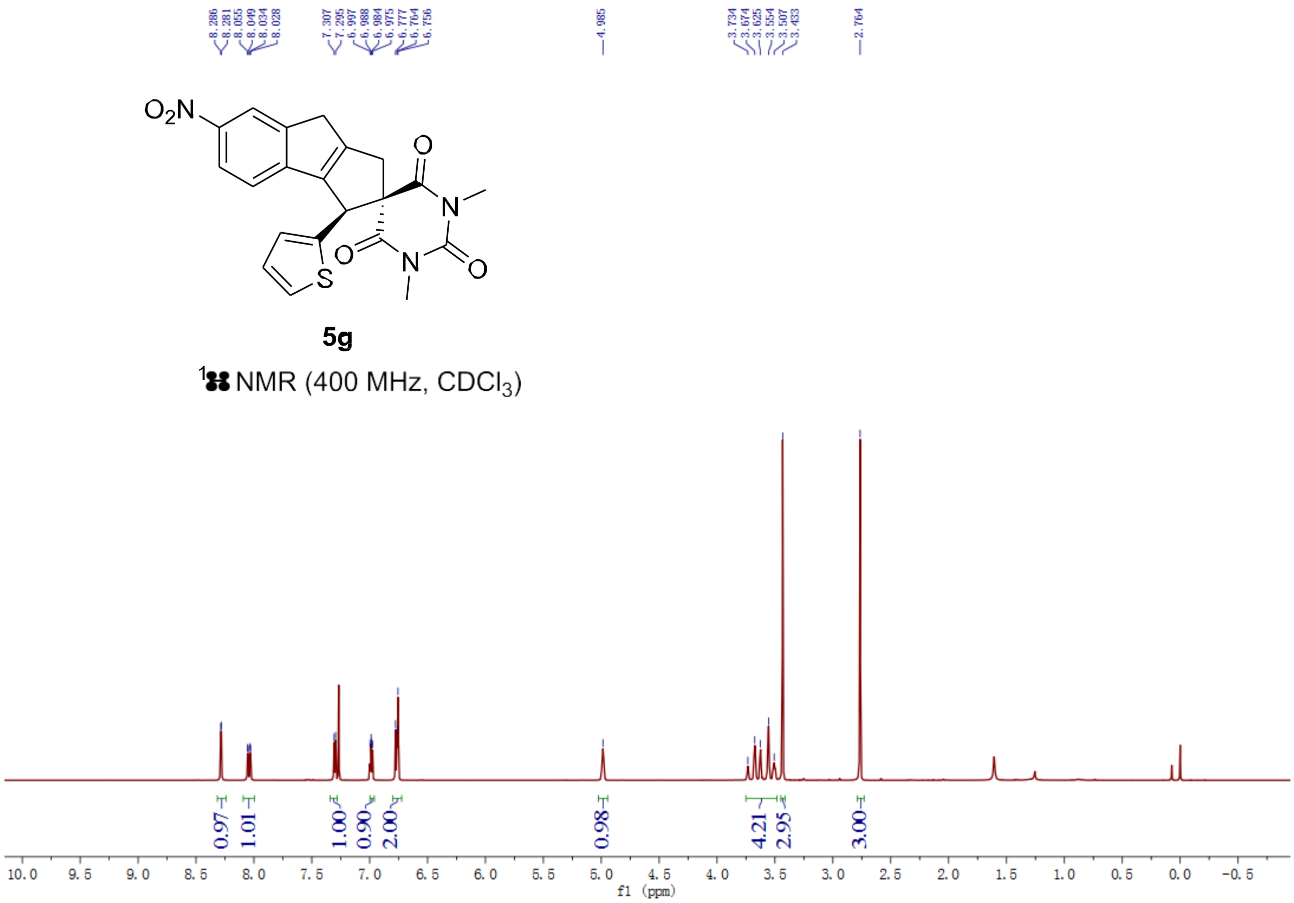
8.286
8.281
8.055
8.049
8.034
8.028
7.307
7.295
6.997
6.988
6.984
6.975
6.777
6.764
6.756
4.985
3.734
3.674
3.625
3.554
3.507
3.433
2.764
O_2N
O
N
O
N
O
S
5g
^{1}H NMR (400 MHz, $CDCl_3$)
0.97
1.01
1.00
0.90
2.00
0.98
4.21
2.95
3.00
10.0 9.5 9.0 8.5 8.0 7.5 7.0 6.5 6.0 5.5 5.0 4.5 4.0 3.5 3.0 2.5 2.0 1.5 1.0 0.5 0.0 -0.5
f1 (ppm)

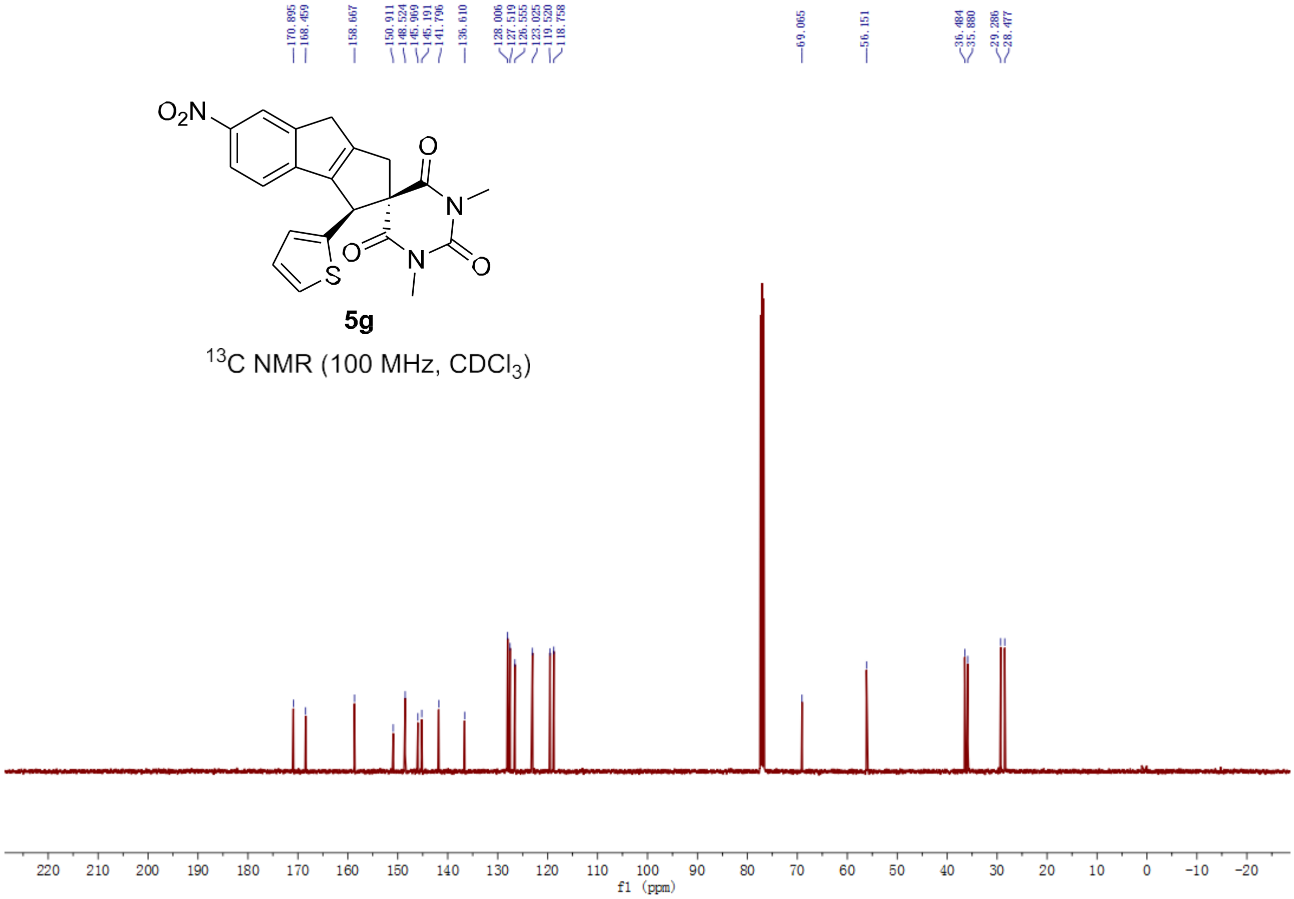
170.895
168.459
158.667
150.911
148.524
145.969
145.191
141.796
136.610
128.006
127.519
126.555
123.025
119.520
118.758
69.065
56.151
36.484
35.880
29.286
28.477
O_2N
O
N
O
N
O
S
5g
^{13}C NMR (100 MHz, $CDCl_3$)
220 210 200 190 180 170 160 150 140 130 120 110 100 90 80 70 60 50 40 30 20 10 0 -10 -20
f1 (ppm)
